# Development of the WHO Antenatal Care Recommendations Adaptation Toolkit: a standardised approach for countries

**DOI:** 10.1186/s12961-020-00554-4

**Published:** 2020-06-22

**Authors:** Maria Barreix, Theresa A. Lawrie, Nancy Kidula, Fatim Tall, Maurice Bucagu, Ram Chahar, Özge Tunçalp

**Affiliations:** 1 Department of Sexual and Reproductive Health and Research, , World Health Organization, 20 Avenue Appia, 1211 Geneva, Switzerland; 2 Evidence-based Medicine Consultancy Ltd, Bath, United Kingdom; 3 Reproductive, Maternal Health and Ageing Team, UHC Life Course Cluster, World Health Organization Regional Office for Africa (WHO-AFRO), Harare, Zimbabwe; 4 Reproductive, Maternal Health and Ageing Team, UHC Life Course Cluster, World Health Organization Regional Office for Africa (WHO-AFRO), Ouagadougou, Burkina Faso; 5 Department of Maternal, Newborn, Child & Adolescent Health & Ageing, World Health OrganizationCountry Office (WHO-CO), 20 Avenue Appia, 1211 Geneva, Switzerland; 6 Maternal & Reproductive Health Team, World Health Organization Country Office for India, New Delhi, India

**Keywords:** Antenatal care, policy-maker, implementation, evidence-based, policy-making, user-testing, toolkit, guidance, WHO guidelines, stakeholder engagement

## Abstract

**Background:**

Increasingly, WHO recommendations are defined by context-specific factors and WHO is developing strategies to ensure that recommendations are successfully adapted and implemented at country level. This manuscript describes the development of a toolkit to support governments to adapt the WHO recommendations on antenatal care (ANC) for a positive pregnancy experience for their context in a systematic manner.

**Methods:**

The toolkit was developed in three steps. It was created with input from methodologists and regional implementation experts (Step 1) followed by a user-testing phase (Step 2), implemented during country stakeholder meetings. User testing consisted of stakeholder interviews that were transcribed, and data was categorised according to the content analysis method. Suggestions for toolkit improvement and issues identified during the interviews were assessed as serious, moderately serious or minor/cosmetic.

**Results:**

A total of 22 stakeholders – comprising five Ministry of Health (MoH) consultants, four MoH policy-makers, and 13 advisors/implementers – from Burkina Faso, India, Rwanda and Zambia participated in user-testing interviews during stakeholder meetings held in each country between August 2018 and February 2019. Most stakeholders had a medical or nursing background and half were women. Overall, responses to the toolkit were positive, with all stakeholders finding it useful and desirable. User testing interviews highlighted four serious, four moderately serious and five minor/cosmetic issues to be managed. These were addressed in the final step (Step 3), an updated version of the WHO ANC Recommendations Adaptation Toolkit, comprised of two main components – a baseline assessment tool with spreadsheets for data entry and a Slidedoc^®^, a dual-purpose document for reading and presentation, outlining the qualitative data that shaped the women-centred perspective of the guidelines, accompanied by an instruction manual detailing the components’ use.

**Conclusions:**

The WHO ANC Recommendations Adaptation Toolkit was developed to support countries to systematically adapt the WHO ANC recommendations for country contexts. Using this approach, similar tools can be developed to support guideline implementation across different health domains and the continuum of care.

## Background

Improving health is key to reaching the United Nations Sustainable Development Goals. Assisting countries in overcoming common barriers to implementing WHO’s evidence-based guidelines across all health domains will be crucial in achieving the Sustainable Development Goals. To address the complex and diverse global healthcare needs, WHO recommendations are becoming increasingly context specific [1, 2] and context-specific recommendations require additional steps to interpret and apply [3]. Furthermore, passive approaches to dissemination of WHO guidelines, such as printing and distribution, have in the past been criticised for being insufficient [4].

As part of its efforts to improve maternal health and service quality, the WHO launched its comprehensive guideline on routine antenatal care (ANC) for pregnant women and adolescent girls, in November 2016 [1]. *The WHO recommendations on ANC for a posiive pregnancy experience* are subdivided into five different content categories (nutritional interventions, maternal and fetal assessment, preventive measures, physiological symptoms, and health systems) and seek to integrate service provision across health domains (malaria, tuberculosis, HIV, syphilis, etc.). The guideline includes 23 context-specific recommendations to be tailored to populations with, for example, certain nutritional needs, endemic infections or healthcare settings [5, 6].

Following publication of the ANC guidelines, WHO received requests from national governments for adaptation and implementation support, especially on how to contextualise and tailor the guideline content to local settings. In many countries, such support is critical to ensure WHO guideline uptake at national and subnational levels. In response to goverments' request, and given the complexity of the new 8-contact ANC model outlined by the new recommendations [5, 6], and the known barriers to implementing guidelines at the country level [7], WHO planned to develop tools to support the adaptation and implementation of the ANC recommendations.

This manuscript documents the process of developing a toolkit designed to assist national governments to systematically (1) adapt the ANC guideline to their contexts and (2) update their ANC policies according to the WHO ANC recommendations. The toolkit’s aim is to facilitate the design of country-specific packages for ANC health services, including essential clinical (i.e. blood pressure, weight and height measurement, etc.) and counselling practices (i.e. birth preparedness, labour companion, etc.), tailored to the individual country’s health system and context. It also aims to highlight country-specific factors that are likely to influence (positively or negatively) the implementation of the stakeholder-approved package as well as what should be considered during the country implementation. The described in this manuscript are part of a wider approach to assist countries in translating and tailoring WHO recommendations to national contexts and settings. The example detailed in this manuscript focuses on ANC; however, the methodologies outlined in this manuscript can be applied to any health domain.

## Methods

**Fig. 1:**
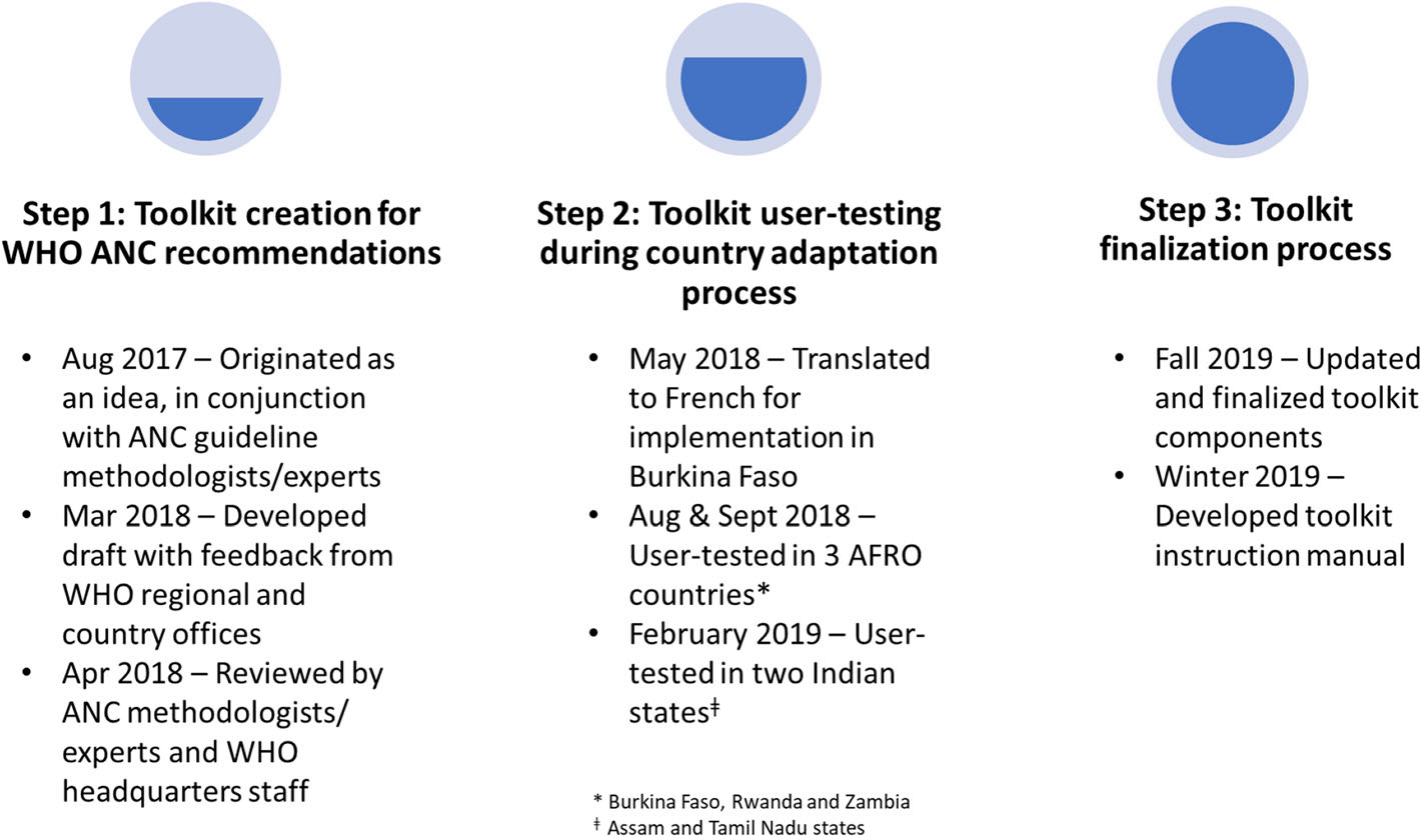
Toolkit development methodology and timeline

The WHO Antenatal Care Recommendations Adaptation Toolkit was created in a three-step process (Fig. 1). Firstly, in Step 1, the toolkit development team (hereafter referred to as ‘the team’) created the draft toolkit. Whilst in Step 2, the team user tested the toolkit in four countries, Burkina Faso, India (two states: Assam and Tamil Nadu), Rwanda and Zambia, during national (or state-level in India) adaptation processes of the WHO ANC recommendations which employed the toolkit. Finally, in Step 3, the team updated the toolkit, based on feedback from the user testing results, and developed an instruction manual describing its use for country adaptation of the WHO ANC recommendations.

### Step 1: Toolkit creation for WHO ANC recommendations

In response to government requests, the idea for the development of a WHO ANC toolkit focusing on policy-makers originated in August 2017 during a meeting in Norway with research methodologists and knowledge translation experts, who had worked on the development of the WHO ANC guideline.

Prior to the first drafting of the components, the team consulted with relevant experts during the WHO’s Sexual and Reproductive Health Department’s Scientific and Technical Advisory Group annual meeting in February 2018 for similar tools employed in knowledge translation efforts as well as in other health domains. In drafting the toolkit’s Baseline Assessment Tool (BAT) component, the team modelled it on similar tools developed by the United Kingdom’s National Institute for Health and Care Excellence (NICE) [8, 9]. Additionally, the team drew on the work of the WHO’s Evidence-Informed Policy Network (EVIPNet) for designing the toolkit's implentation and the stakeholder meetings' organisation [10].

The first outline and subsequent draft versions of the toolkit were developed by ÖT, TAL and MBa. The toolkit was then further refined in collaboration with WHO headquarters, regional and country office colleagues (including NK, FT and MBu) during a March 2018 meeting in Lusaka, Zambia. Subsequently, in April, a second version of the tool was reviewed by the methodologists and experts (from the original Norway meeting) and it was decided that a second informational component on the Qualitative Evidence Synthesis (QES), which underpinned the ANC recommendations would be added to the toolkit in the form of a Slidedoc^®^; thereafter, during user testing, the two components together were referred to as the WHO ANC policy-maker toolkit [11]. The toolkit was also shared with WHO headquarters staff with expertise in maternal and child health for further feedback.

### Step 2: Toolkit user-testing during country adaptation process

In parallel to the tool development process, WHO has been directly working with four countries, Burkina Faso, India (in two states: Assam and Tamil Nadu), Rwanda and Zambia, to update their ANC policies based on the 2016 WHO recommendations. To do so, the four countries employed the toolkit for conducting a situational analysis of current ANC service delivery, held stakeholder consultations, and updated and validated national ANC policy changes. Stakeholder meetings were organised by the Ministry of Health (MoH) in each of the four countries to assess and update the national ANC policy, where this toolkit was used to inform the proceeding. In preparation for use in Burkina Faso, the toolkit was translated into French. This country guideline adaptation process, where the toolkit was applied, created an opportunity to user-test the toolkit with the goal of improving it and making it more useful and user friendly. The toolkit user-testing process comprised a survey of the views and experiences of users through individual stakeholder interviews. The survey was modelled after user testing efforts carried out previously by Norwegian Public Health Institute [12, 13]. Those interviewed were exposed to all or part of the toolkit either prior to or during the stakeholder meetings. Potential user-testing stakeholders were identified by members of the team, WHO country staff, or the country consultant who supported the process, to ensure that all relevant users of the toolkit would be represented in the findings. Stakeholders who were invited to take part in the user-testing provided written informed consent.

The standardised interview guide used was adapted from a guide employed in user-testing of the DECIDE framework [14], and included open-ended questions on the usefulness, user-friendliness, credibility and desirability of the toolkit. User-testing interviews were conducted during coffee and lunch breaks, or immediately after concluding the stakeholder’s meetings, in a 30- to 60-min process. Each interview was conducted by an interviewer (MBa or TAL) and accompanied by an observer/notetaker (MBa, TAL, NK, FT or RC) with an in-depth knowledge of the toolkit. In addition to toolkit-related questions, interviewed stakeholders were invited to comment on the usefulness and value of the stakeholder meeting. The project was reviewed and approved by the UNDP/UNFPA/UNICEF/WHO/World Bank Special Programme of Research, Development and Research Training in Human Reproduction’s research project review panel.

All interviews were transcribed and checked (by TAL and MBa) and data from Burkina Faso were translated to English. Following this, TAL and MBa categorised and coded the data using content analysis methods, and employed a pre-defined framework. The framework grouped data according to problems identified, positive feedback, and suggestions for improvement. ‘Problems identified’ were further categorised into three groups, namely (1) serious issues, (2) moderately serious issues or big frustrations, and (3) minor or cosmetic issues. Categorisation into these three sub-groups was done subjectively by TAL and MBa (criteria were not developed, instead shared experience and understanding of the toolkit served as a basis for this step). Differences were resolved by discussion or by involving a third author (OT). Common themes with respect to problems and suggestions related to the different toolkit components were tabulated; thereafter, the team considered how best to address them. Participant comments and suggestions concerning the stakeholder meetings were also considered.

## Results

As a result of Step 1, the team created a draft version of the WHO ANC Recommendations Adaptation Toolkit. During Step 2, the team led the user testing of the draft version within the context of country implementation. The findings from the user testing process and the resulting updated version of the toolkit (Step 3), including a user’s manual describing how to employ it to inform country adaptation processes, are detailed in this section.

### Step 2: Toolkit user-testing during country adaptation process

For the user testing of the ANC toolkit, 22 stakeholders from Burkina Faso (*n* = 4), India (*n* = 7), Rwanda (*n* = 6) and Zambia (*n* = 5) participated and provided feedback. User-testing stakeholders fell into three categories, namely consultants who had completed the situational analyses and draft country reports (*n* = 5), MoH policy-makers (*n* = 4), and advisors and other stakeholders (*n* = 13). Most stakeholders (17/22) had a medical background, i.e. either held a medical degree or a nursing or midwifery qualification. There was equal distribution between male and female stakeholders across the sample. Most interviews were conducted in English (*n* = 17), the remaining five were conducted in French (four in Burkina Faso and an interview in Rwanda, which was conducted in English and interpreted to French for the participant).

### Baseline Assessment Tool (BAT) user-testing results

#### User feedback from the consultants

The country consultants were the stakeholders with the greatest exposure to the toolkit, having received it at least 6 weeks prior to the stakeholder meetings and having used it to compile the draft country reports for discussion at the meetings. All five consultants stated that they were familiar with these types of toolkits. However, most expressed that the BAT component of the toolkit was more detailed than usual, and their first impressions suggested that it was rather intimidating. For example, comments included “*A lot of sheets!*”; “*...at first, I thought I wouldn’t master the guideline in any way, it would take a long time...*”; “*I thought what have I gotten myself into!* [laugh] *I found it voluminous and complex*.”

Only one of the consultants stated positively that their first impression was a “*wow impression*”. For the others, their responses suggested that it was only after working with the BAT that they appreciated that the level of detail required facilitated a comprehensive understanding of the existing situation of ANC service provision.

- “*…as I went on I realised there was more to the tool...*”
- “*First it was overwhelming, then understandable that each* [item in the BAT] *shows the total picture*.”
- “[It is] *comprehensive and good; the recommendations tab gives an idea of how we are performing*.”
- “[It is] *an informative excel sheet with so many areas that were helpful to understand what is happening with the programmes running*.”
- “*The* [Excel] *sheets were very useful and helped make the report*.”

The time taken for consultants to complete the situational analysis ranged from 2 to 4 weeks. Consultants explained that this was because:

- “*It took me one week to understand it fully* [before I could start to complete it]. *One week is necessary*.”
- “*...to fill it out I really needed reliable sources*.”
- “*It took time to coordinate with MoH people*.”
- “*Finding people to sit with and ask questions took time, because they were mostly out of town*.”

Consistent with their initial impressions, three of the five consultants found the BAT quite onerous to complete due to the level of detail required, whereas the other two did not, for example, stating that the spreadsheet had “*...good linkages, is easy to use, and flows logically*”. However, all consultants unanimously agreed that it was a useful tool, partly due to its detail:

- “*It help*[*sic*] *to clearly inform what needs to be done so as not to miss anything*.”
- “*It is probably something that should be done every so often so that you are improving on service delivery*.”

#### User feedback from the policy-makers

In addition to receiving the draft report compiled by each country consultant, the four MoH policy-makers received the complete toolkit prior to their respective country stakeholder meetings. Most said that they had not read the BAT in detail before the meeting, therefore their exposure to the materials was less than that of the consultants. Initial impressions of the BAT were positive. One policy-maker noted that “*They were requesting much information!*” All four considered the BAT to be useful because of the detailed outputs it provided:

- “*All the information is useful because it goes deeper than the data that the MoH currently has on ANC services*.”
- “*I expected a few questions regarding key points which were missing* [from our existing policy] *according to the new guideline, but this one goes deep*.”
- “*At first, I didn’t think it was necessary, but in the end I realised it was necessary*.”
- “*It gives the big picture or overview of the programme and touched on many areas not usually thought about, for example, human resources*.”

In general, the MoH policy-makers found the toolkit to be a desirable aid for decision-making:

- “*There were some recommendations that I was not aware of* [and it was helpful for these].”
- “*It encompasses many things. We can use it, employ it at different levels of the health system*.”
- “*This tool has components of things we are doing and components of things that we are not doing. It’s a reminder that if there’s evidence those things work, we jack ourselves up and... implement the areas we are not doing*.”

#### User feedback from advisors and other stakeholders

Most of these stakeholders commented only on the ‘Output’ sheets of the BAT; however, for the Rwanda stakeholder meeting, the toolkit was included with the other pre-meeting documents. Therefore, three of the advisor-type stakeholders in Rwanda also provided feedback on the full BAT. Comments from this group of stakeholders were consistent with the other two groups with respect to the need for clear instructions on how to complete the BAT and, in particular, the meeting group work components (the country-specific ANC package (output 1) and the SWOT analysis (output 2)).

What was notable from this group, in particular, were concerns expressed about the meeting group work outputs not adequately capturing implementer issues:

- “[In our group] *we were not aware of the real challenges on the ground*.”
- “[There were] *a lot of ground level issues missing. Data was good to see but challenges and difficulties at ground level need to be discussed at central level*.”
- “*Some information that was not captured by the tool...why, what is the cause?*”

Feedback from this group helped to identify certain country-specific interventions that were missing from the BAT (e.g. malaria counselling). This group also had issues with certain terminology used in the toolkit, when it differed from what was used in their settings.

Issues identified with the draft version of the BAT are tabulated in Table 1, alongside the actions taken by the team to address them. Where stakeholders offered suggestions on how to improve the BAT, the team gave these due consideration during the toolkit revision.

**Table 1:** Issues identified and suggestions offered by stakeholders of the user-testing study

Issues and Suggestions	Seriousness	Toolkit Development Team Actions
**Baseline Assessment Tool (BAT)**		
Clearer instructions are needed to complete the BAT	XXX	We have written an instruction manual (Additional file 3) on how to use the toolkit.
The purpose of the Recommendations spreadsheet and Outputs sheets were difficult to understand	XXX	We have revised the heading of this sheet to ‘Recommendations mapping’ to make its purpose clearer. Similarly, we have changed the headings of the Output sheets to be descriptive rather than numerical; for Output 1 (country-specific ANC package), we have removed the row requiring input on ‘Interpersonal support’ from each of the 8 contacts and, instead, inserted a single input column for these data to avoid repetition; for Output 2 (SWOT analysis), we have modified it from focusing on innovations to focusing on new and updated recommendations, which the Ministry of Health will have to coordinate. Additionally, we added a column for ‘ongoing implementation and research efforts’ and have highlighted that the SWOT analysis relates to any new and updated recommendations.
In the French version, the links (conditional formatting) between the Situational analysis sheet and the Recommendations sheet are not functional	XXX	We have fixed this technical issue.
The BAT does not capture ground level issues, e.g. related to minority populations or field workers	XXX	We have added a column to the Population statistics section (Item 2.8) of the situational analysis tool tab to provide for regional or population variations in the indicators; we have also added item 3.5 as follows: “*Please describe any equity issues affecting health service coverage and quality*”. Additionally, we have revised the SWOT analysis (Output 2) to better capture ground level issues.
The formatting was frustrating and needs to be improved	XX	We have modified the formatting to make it more user friendly; however, we realize that Excel is not the best medium for the BAT and we are planning to convert it to a website/HTML format.
Recommendations should be linked to an implementation plan	XX	Implementation guidance is the next step in the process; issues related to implementation are likely to be country specific and how to address them will depend on the toolkit outputs, which require finalisation after the stakeholder meetings.
Some interventions and outcomes are missing from the Recommendations sheet	X	We have added these where relevant.
The BAT would be better as a word processing document than a spreadsheet	X	We understand that word documents are helpful to some people; however, as they do not facilitate analysis, we believe that a spreadsheet format is justified. The planned HTML version of the toolkit will be more user friendly.
Certain terminology is considered not widely used	X	We have clarified the problematic terms used or have provided alternative terms, e.g. community health worker or accredited social health activist.
**Slide document**		
The logic models are quite complicated	XX	We have revised the logic model graphics to make them more accessible to users.
Slides had a lot of information	XX	In the new instruction manual, we have suggested that the meeting organisers share the Slidedoc^®^ (as a pdf booklet) with stakeholders prior to the meeting so they can have time to review the full contents of the Slidedoc^®^ prior to the meeting’s presentation.
Need to improve the French translation	X	We hired a professional translator to edit and improve the French version.
Tailor pictures to setting or make the pictures more diverse	X	Organisers can substitute the pictures in the Slidedoc^®^ as necessary to represent the national context, or conversely make the pictures more diverse to represent a global audience; we have modified the Slidedoc^®^ in accordance with the latter.
**Stakeholder meeting**^**a**^		
The meeting was too short	NA	Organisers should consider this suggestion, depending on the time and resources available.
Groups were too big	NA	Organisers could consider smaller groups, e.g. a group size of 10 instead of 20. This would mean that there would be four groups instead of two; as the feedback session would take longer (while each group presents their results) this would have implications for the meeting duration. If smaller groups are preferred, ensure that a selection of different stakeholders are represented within each group.
Have another room available for group work	NA	Organisers should consider this suggestion, as having adequate space for group work is important to facilitate open discussions.
Grass roots issues should be discussed	NA	Organisers should ensure that implementers and service users are among the invited stakeholders and that their voices are heard in the discussions. Suggestions for representatives to be invited are detailed in the instruction manual.
Include implementation case studies and experiences from other countries	NA	We have now included the experiences of two country case studies in this paper (Boxes 1 and 2) and organisers might wish to refer to these in their presentations.
Presentations could be shortened	NA	Organisers might prefer to keep presentations brief to maximise the time for group work.

*NA* not applicable, *XXX* serious, *XX* moderately serious/big frustrations, *X* minor/cosmetic

^a^ The team collated and reviewed these suggestions but did not make judgements about their seriousness

Overall, user testing of the BAT identified four issues that the team assessed as serious (Table 1). One related to the need for clear instructions on how to complete each sheet. Another concerned a technical problem with the conditional formatting linkage between different sheet parameters in the French version. Formatting in general, was a frequent source of user frustrations. Comprising many columns and rows, headings were sometimes lost when scrolling across sheets, making the viewing and understanding of some sections challenging.

The ‘Recommendations’ sheet was a particular source of confusion, and its purpose was often misinterpreted, leading to incomplete or inappropriate data collection. Another problem that the team assessed as serious was the apparent failure of the BAT to identify prevailing equity issues in some countries.

### Feedback on qualitative evidence synthesis slide document

Overall, meeting stakeholders trusted the information in the Slidedoc^®^, found it easy to understand and visually appealing from the graphics and pictures. For example, a graphic depicting an experience of a fictional teenage pregnancy resonated with stakeholders as true and humanising the ANC experience. Most concerns related to the amount of information on the slides and the size of the font, although two stakeholders commented that the logic models at the end of the Slidedoc^®^ were rather complicated. Especially in India, stakeholders wanted more pictures representing the local population and examples representing the country context. Thus, comments from a variety of stakeholders included:

- “[It] *is easy to understand because it has information and images to convey the evidence...*”
- “*I did like the caricatures which present stories and make it easy to understand...it helps people understand why WHO has issued these new recommendations*.”
- “*There was lots of information and the slides had too much information. If you’re not sitting close you cannot read it*.”

In assessing the feedback, the team agreed that the issue with the logic models and the amount of information presented in the Slidedoc^®^ was moderately serious; other comments and suggestions were minor or cosmetic. Table 1 shows the respective actions taken to address them.

### Feedback on national stakeholder meetings where the toolkit was used

In general, stakeholder liked the meetings and appreciated being included in them. As it was a Ministry-led and WHO-supported process, stakeholders found the toolkit, presentations and process trustworthy and credible. They especially appreciated the representation of diverse stakeholders and felt that this was key to the success of the meeting:

- “*We got different views coming from programme managers, health providers, partners – I mean, I think we got information from all categories of stakeholders, which is why it was really helpful – especially from those providing services*.”
- “[The stakeholder meeting] *will definitely help to implement the model here, because they tried to involve many stakeholders, especially people who are implementing or supporting the Ministry to do ANC*.”
- “*So having everyone together, like, you had midwives, you had doctors, policy-makers working together, it was positive*.”

In addition, evidence of government support through the presence of the Health Ministers and other senior members of the MoH was important to stakeholders and gave them confidence in the process:

- “*The fact that* [he] *was leading and taking ownership, and he is a senior person in government, that helps, because it also shows that there is commitment*.”
- “[It was good]*, seeing the involvement of the higher authorities, even the Minister, joining the team, showing everyone that* [improving ANC] *is supported by the country*.”

Conversely, for meetings where stakeholders felt that representation was limited, stakeholders were more likely to be sceptical of achieving a successful outcome:

- “[We] *need more representation from grass roots level*.”
- “[We needed] *more comments from basic officers, implementers*.”
- “*I was a bit scared regarding implementation because the Stakeholders were not truly familiar with the recommendations*.”

Whilst the group interactions were highly praised and appreciated by stakeholders, sometimes they did not feel comfortable to voice their opinions:

- “*Not all reps* [supervisors and district health personnel] *participated actively in discussions*.”
- “[There should have been] *more interaction and stimulation for them* [the quieter ones] *to give their ideas*.”

The size of the participant groups for the group work sessions consisted of about 20 stakeholders in most instances. This, as well as the space that was available to conduct group work, was raised by some as a barrier to effective discussions:

- “*The discussion groups were very big and so, often only a few people were talking*.”
- “*Groups were very big, it would have been better to split into four. It also would have been good to get another room for the group work*.”

Suggestions offered by stakeholders on how to improve the stakeholder meetings are also summarised in Table 1.

### Naming the toolkit

The toolkit was provisionally named the ‘WHO ANC policy-maker toolkit’. Stakeholders were asked what they thought about the name and could offer suggestions. It was noted that the toolkit might not be used exclusively by policy-makers, therefore, this term was not favoured. Whilst several stakeholders favoured inclusion of the term ‘positive pregnancy’ in the name, others favoured a simpler title that clearly described its purpose. Hence, after considering all participant comments and suggestions, the final name of the WHO ANC Recommendations Adaptation Toolkit came about.

#### Step 3: Toolkit updating process

The WHO ANC Recommendations Adaptation Toolkit, accompanying the WHO ANC guideline, comprises two main components: a BAT with spreadsheets for data entry and information (Additional file 1) and a Slidedoc^®^, a dual-purpose document for reading and presentation, outlining the qualitative data which helped shaped the guideline’s woman-centred perspective (Additional file 2) The toolkit components, as well as their modifications based on user testing, are outlined in Table 2.

**Table 2:** WHO antenatal care (ANC) Recommendations Adaptation Toolkit components

Component	Description
1. Baseline Assessment Tool (BAT)	The BAT consists of a Microsoft Excel file which includes sub-components a–c.
a. Situational Analysis Tool	The situational analysis tool tab serves as a data collection guide and is loosely adapted from the WHO health system building blocks [15, 16]. Questions (soliciting mostly dichotomous yes/no or numerical responses) are grouped as follows: (1) Leadership and governance (2) Health information systems (3) Services delivery (4) Health workforce (5) Financing (6) Access to essential medicines (7) Patient and population engagement (8) Existing ANC model. Information from section (b) of the tab sets population parameters establishing which context-specific recommendations should be applied or not. The entirety of the results from this exercise are then summarised in a narrative report, which also highlights challenges and promising initiatives supporting the implementation of the country’s current ANC model.
b. Recommendations Mapping Exercise	This tab assists users to map the country’s existing ANC policies to the WHO ANC recommendations. It also provides users with a link between the population parameters and the recommendations that apply (or not) to the country setting. Users are asked to compare how current activities (and related policies) align with each of the 49 ANC recommendations, who performs said activity (main ANC provider or other), and whether a national policy change or update is necessary. Additionally, the Ministry of Health programme manager responsible for said change should be identified, as well as a timeline for the update to take place.
c. Country-specific ANC package (Output 1) and Strengths, Weaknesses, Opportunities and Threats (SWOT) analysis for implementation of new or update ANC recommendations (Output 2)	The country-specific ANC package (Output 1) is an output in which all recommended ANC activities to be implemented during each of the eight contacts are outlined. The activities are broken down into three categories that emerged from a scoping review [17] on what women want from ANC care, namely information, medical interventions and interpersonal support. This output also outlines which healthcare cadre provides each activity, the level of the health system at which the intervention will be provided, and the phasing, if necessary, to scale up that activity (i.e. the country may decide to phase the implementation of the ultrasound recommendation to procure equipment, train staff, etc.) Output 2 consists of a SWOT analysis. In the version that was user tested, the SWOT analysis focused on innovations that could assist the country’s implementation of the ANC package. Innovations were drawn from the health system-related recommendations in the ANC Guideline (section E) as well as existing national initiatives or local pilot programmes with potential for scale up. However, based on stakeholder feedback, we have modified the SWOT analysis to respond to new and updated recommendations that the country will implement.
2. Qualitative Evidence Synthesis (QES) Slidedoc^®^	This PowerPoint, built using Duarte Slidedoc^®^ format, details how the QES were employed, not only to inform the ANC recommendations (regarding values and acceptability) but also in shaping the entire guideline development process and explaining the focus on a ‘positive pregnancy experience’. An abbreviated version of the Slidedoc^®^ was used during the stakeholder meetings, after a brief presentation on the overall guideline, to set the tone of the meeting.
Supplementary materials: a. Implementation considerations b. Remarks section from each recommendation c. National ANC guideline template d. Draft agenda for stakeholder meeting e. Draft group work materials for stakeholder meeting	Following user testing feedback, all supplementary materials were moved to the instruction manual (Additional file 3). These materials contain the implementation considerations and the remarks section from each recommendation, which were extracted verbatim from the WHO ANC Recommendations [1]. They aim to help toolkit users develop Outputs 1 and 2. The draft template is aimed at guiding countries in their development of an updated ANC policy (after completing the mapping), which should align with the longer-term national reproductive health strategy. The supplementary materials (draft agenda and draft group work materials) aim to help the MoH organise and carry out the stakeholder meeting.

Furthermore, the toolkit is accompanied by a user-friendly instruction manual (Additional file 3) to guide stakeholders through updating their national ANC policies using the toolkit. It includes detailed instructions on the process to complete the different components of the BAT (Additional file 1). Table 3 outlines the process to use this toolkit effectively as part of the country adaptation and implementation process, whereby, following the use of the toolkit in the initial stakeholder meeting, countries then finalise their updated integrated ANC package and national ANC policy. Next, countries develop an implementation plan (including revised ANC facility registries and user cards and well as coordination for all necessary resources, i.e. staffing, equipment and materials) and a related budget.

**Table 3:** Process for employing the WHO Antenatal Care (ANC) Recommendations Adaptation Toolkit

Activity	Toolkit component used	Timeline
Introductory meetings held with the Ministry of Health (MoH) and WHO country office staff to ensure government support and set up an integrated MoH team (with representatives from TB, HIV, malaria, adolescent, safe motherhood, nutrition, etc. programmes) to lead the activities.	None	Approximately 1.5-hour meeting
Local consultant hired to work with the MoH team lead to support the following activities: 1) Completion of the Baseline Assessment Tool (BAT) to produce a situational analysis report of current ANC health service provision, 2) Conduct a mapping of national ANC Guidelines in comparison with WHO 2016 ANC recommendations, highlighting necessary changes and/or updates 3) Produce a draft integrated country-specific package of all ANC interventions, detailing what activities will take place at each contact, by whom and at what health system level 4) Participate in and document stakeholder meeting discussions 5) Finalise outputs 1 and 2 6) Draft updated national ANC guideline for validation, based on outputs 1 and 2 7) Participate and document validation meeting 8) Finalise updated national ANC guideline	BAT	65 working days (~ 3.25 months)
MoH held an ANC stakeholders meeting to update national ANC policies based on WHO 2016 ANC recommendations. Activities included: · Presentation of Qualitative Evidence Synthesis (QES) Slidedoc^®^ · Presentation of WHO 2016 ANC recommendations · Review of situational analysis report · Review of Outputs 1 and 2 during group work · Presentation of group work results in plenary discussions	QES Slidedoc^®^, Outputs 1 and 2, Group work material	2 days
MoH team and consultant finalised meeting outputs, including the situational analysis, the proposed country-specific ANC package (Output 1) and SWOT analysis (Output 2), and the country report. An updated national ANC policy and implementation plan, including phasing for national scale up, were also drafted.	Situational analysis report, Outputs 1 and 2	2 months
MoH held validation meeting(s) for stakeholders to approve the proposed country-specific ANC package, updated national ANC guidelines and implementation plan.	Situational analysis report, Outputs 1 and 2	2 days
MoH updated ANC-related facility-based tools, such as paper registers and mother’s case notes, and validated them in a further stakeholder meeting(s).	Output 1	3 days

## Discussion

The WHO ANC Recommendations Adaptation Toolkit was developed to support countries adapt and implement the 2016 WHO ANC recommendations in a systematic and transparent way. This iterative 3-step approach, which was well received by a variety of stakeholders, could be replicated in other healthcare domains to support effective guideline adaptation and implementation. In alignment with Straus et al.’s knowledge translation framework, the toolkit seeks to both assess barriers in knowledge use and adapt knowledge (from the WHO ANC guideline) to local context [18–20]. To receive feedback on the draft toolkit, four countries (Burkina Faso, India, Rwanda and Zambia) employed it and carried out its user testing. The user-testing process involved diverse stakeholders and was extremely valuable in the development and improvement of the toolkit. Interviews highlighted a range of issues, from minor to serious, that could be addressed by the development team before releasing the toolkit for global use.

The development process was iterative and, while the resulting version of the toolkit is presented with this manuscript (Additional files 1, 2, and 3), the team anticipates that further changes will be made to the toolkit as it is employed in other countries and the team receives more feedback from users. For example, under the guidance of WHO regional office, other countries have used this toolkit in their national processes. Boxes 1 and 2 provide examples of this process in Uganda and Sierra Leone, respectively. While user testing data was not systematically collected in Uganda and Sierra Leone, a number of lessons learnt were incorporated, such as the need to update clinical and woman-held tools and facilities registers as well as indicators to align with ANC recommendations.

The creation of an instructor’s manual for toolkit use (Additional file 3) was a direct response to feedback from user testing. Additionally, some sheets in the BAT were originally included as reference material only. However, during teleconferences with consultants to support their completion of the BAT prior to the stakeholder meetings, it became apparent to the team that users were sometimes confused regarding the purpose of these informational sheets. Therefore, in revising the toolkit, seven of these informational sheets were moved to the instruction manual. All sheets in the BAT now require action, whilst all informational material can now be found in the manual. This aims to make the toolkit more user friendly.

It is important to note that prior to and during stakeholder meetings, various stakeholders voiced concern that the situational analysis process had been based on a desk review only, and believed that primary data collection (whether quantitative or qualitative) would be more helpful for identifying relevant barriers to ANC provision. While this is a valid concern, the team did not modify the toolkit to include primary data collection, mainly due to the resource and time implications of this endeavour; the BAT was designed with the ultimate goal of being simple and of minimal cost. In addition, a varied stakeholder representation (professional associations, community-based organisations, women’s groups, etc.) could supplement the information collected in the BAT. However, depending on resources and time availability, countries may want to conduct primary data collection.

In general, stakeholders highly valued relatable references and imagery in the QES Slidedoc^®^. For example, the presentation included the logic models for women who attend none, partial or full ANC services and this aimed to help stakeholders consider and identify local factors affecting utilisation and provision of ANC services [17]. Whilst the full Slidedoc^®^ comprises 52 slides in total, a smaller slide deck of 25 slides was used for the stakeholder meeting presentation. User-testing suggested that the accessibility of this document would be enhanced by the insertion of photographic images that reflect local populations and culture. Therefore, consultants and organisers should be encouraged to source these and insert them into the Slidedoc^®^, which is editable as well as local qualitative data to inform and support local adaptation. Sharing the full Slidedoc^®^ with stakeholders before the stakeholder meeting would also be helpful.

As anticipated, stakeholder meetings were more productive when a diverse group of stakeholders, particularly providers and service users, were represented and encouraged to contribute their opinions and experiences to the meeting discussions. Suggestions provided during the user-testing which aimed at optimising stakeholder engagement are also included in the instruction manual.

To assist other countries interested in adapting and implementing WHO’s ANC recommendations and, ultimately, to increase the impact of the recommendations at country level to improve health outcomes, next steps include making the toolkit available to accompany the WHO ANC guideline, developing an online version of the BAT, and modifying it for use at sub-national decision-making and health system levels.

The toolkit’s development is similarly linked to broader efforts to support healthcare providers in implementing the ANC recommendations. WHO has been exploring other ways to improve knowledge translation through digital health and innovation, including digital reference modules. For this, WHO has created the WHO digital ANC Module for healthcare workers, which provides decision support and longitudinal client record systems. The Module represents a digital health intervention in line with WHO guidance [2]. Further, the integrated ANC package (output 1) will allow for the customisation of the digital Module to different country settings.

Box 1 Adaptation of WHO ANC Recommendations 2016 in Uganda In August 2018, the Ugandan Ministry of Health (MoH) requested technical assistance from the WHO Country Office (WCO) to adapt the WHO ANC recommendations for a positive pregnancy experience and derive national guidelines and tools to support the implementation of this new approach. NK supported the country in applying the draft toolkit. The MoH led the completion of the BAT and convened a 5-day stakeholder workshop comprising a variety of stakeholders. During the workshop, the baseline assessment results were disseminated, discussed and validated. Stakeholders were oriented on WHO ANC recommendations and in working groups, reviewed and discussed them in detail. Using the situation analysis data, the stakeholders identified the recommendations relevant to Uganda and defined the country’s minimum essential package of ANC interventions. These were mapped by contact, cadre and level of care, and the time period for implementation was determined, namely short, medium or long term. The stakeholders outlined activities and interventions to be provided during outreach services and at community and household levels and defined key messages to be provided by the Village Health Teams. Subsequently, the ANC client take-home card, TB screening, and birth and emergency preparedness plan tools were reviewed and revised to reflect the updated country-specific ANC package. These were piloted in selected sites and have been scaled up. Finalisation of the training materials, which will include management of common complications of pregnancy (i.e. malaria, prevention of mother-to-child transmission, elimination of HIV and syphilis) and preconception care is ongoing.

The country team noted that the toolkit and the approach for adapting the new recommendations was very useful and recommended that a similar approach be used for other guidelines. Discussing the individual recommendations and explaining the evidence behind them not only facilitated the uptake of new recommendations but also the removal of harmful local practices that are not recommended.

Box 2 Adaptation of WHO ANC Recommendations 2016 in Sierra Leone In April 2018, the Ministry of Health (MoH) requested WHO technical and financial support for adapting the new antenatal care (ANC) guidelines for Sierra Leone. Following the experience in Burkina Faso, the draft toolkit was used to support the adaptation process in Sierra Leone. FT shared the toolkit and oriented WHO Country Office (WCO) staff on the process through teleconferences. The Baseline Assessment Tool (BAT) detailing the current status of ANC service provision was completed. In May 2018, a stakeholder workshop was organised and included 32 representatives from a diverse group of partners. During the meeting, the situational analysis was validated and the challenges and opportunities for the adaptation and implementation of each recommendation were discussed. An integrated ANC package for the Sierra Leonean context was developed detailing where interventions could be provided – facility (fixed or outreach) or community level. The national Maternity Record Card was reviewed and harmonized with the new ANC package. Stakeholders identified the following next steps – a national validation and adoption meeting; print and dissemination; development of training materials and job aides; development and implementation of a communication plan for dissemination among clinicians; advocacy to develop a plan to make available all necessary items to allow implementation of the new ANC package (equipment, supplies, drugs, commodities, competencies, etc.); and planning for supervision, monitoring and evaluation.

In Sierra Leone, a consultant was not hired, the MoH and WHO regional office completed the situational analysis making the effort less expensive and enabling local MoH and regional WHO staff to better understand the tool; however, it was time consuming.

Stakeholders found the tool to be very useful; it allowed them to go through each of the recommendations in detail, including the implementation considerations. It also raised awareness about the lack of data on indicators required for decision-making. Overall, the process highlighted the need to involve partners early on, to ensure their commitment and engagement in supporting the roll out.

## Conclusions

WHO is committed to providing technical support to ensure countries achieve effective implementation of guidelines. The WHO ANC Recommendations Adaptation Toolkit is a successful example of the organisation’s new approach to active dissemination for adopting new clinical and health systems recommendations, focused on quality of services. The toolkit was employed to support four countries adapt and prepare to implement the 2016 WHO ANC recommendations. User-testing and stakeholder engagement made a valuable contribution to the development process of the toolkit, leading to the production of a more user-friendly and effective product, accompanied by an instruction manual. Furthermore, this toolkit and the approach to its development is informing an overall adaptation and implementation strategy for guidelines across the maternal health continuum. Such tools can be replicated across health domains for effective guideline adaptation and implementation.

## Supplementary information

**Additional file 1.** Baseline Assessment Tool (BAT).

**Additional file 2. **Qualitative Evidence Synthesis (QES) Slidedoc^®^.

**Additional file 3.** Instruction Manual for the WHO Antenatal Care Recommendations Adaptation Toolkit.

## Data Availability

All data related to the study have been reported in this manuscript and relevant associated material are included in the supplementary files.

## Abbreviations

ANC: Antenatal care
BAT: Baseline Assessment Tool
MoH: Ministry of Health
