# Supplementary material for: Development of the WHO Antenatal Care Recommendations Adaptation Toolkit: a standardised approach for countries
Source: Health Res Policy Syst. 2020 Jun 22;18:70. doi: 10.1186/s12961-020-00554-4 (PMC7310220; doi:10.1186/s12961-020-00554-4)
Supplement: Supplementary file 2 — Additional file 2. Qualitative Evidence Synthesis (QES) Slidedoc®. [file 12961_2020_554_MOESM2_ESM.pptx]

## Slide 1
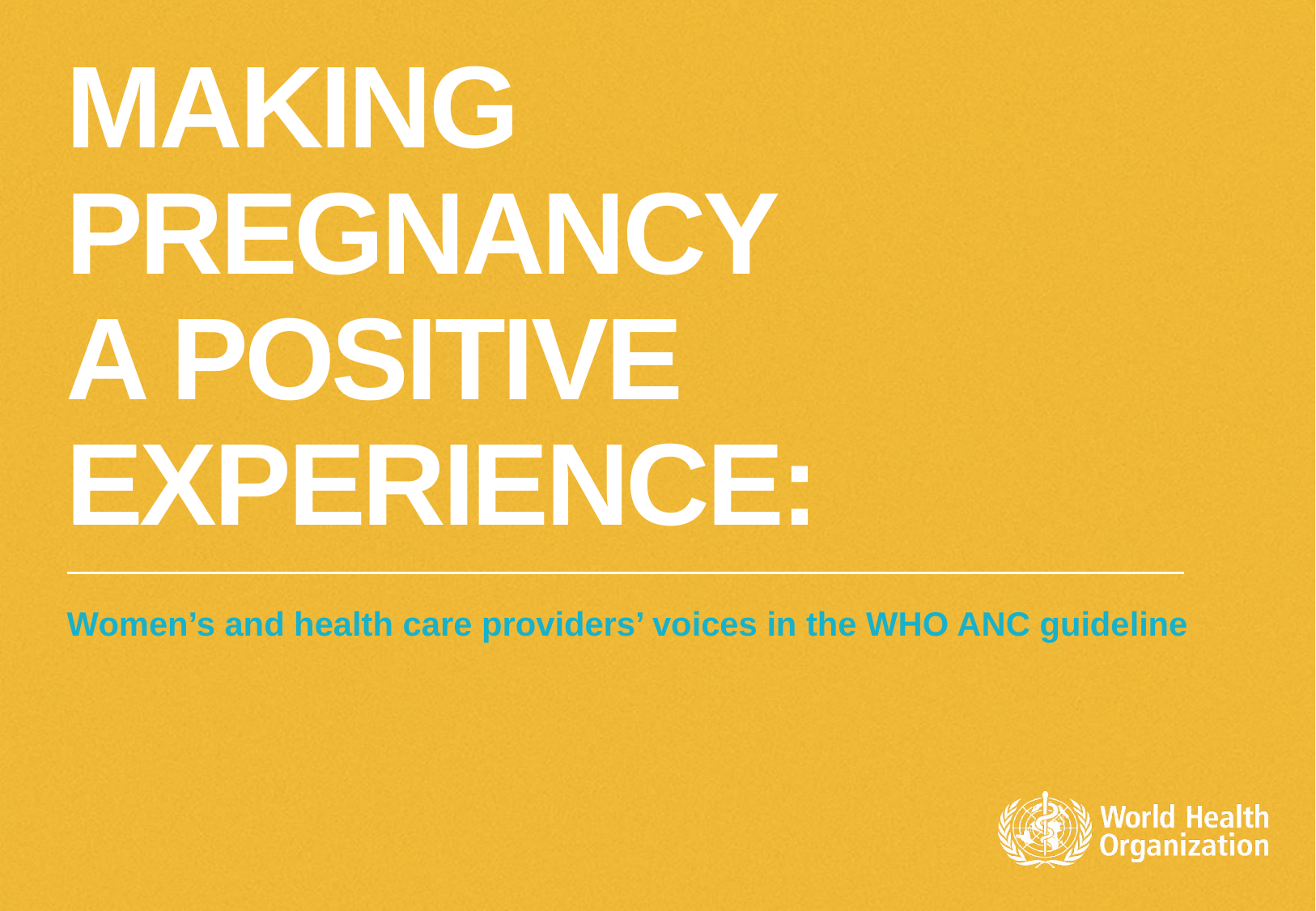

# MAKING pregnancy a positive experience:
Women’s and health care providers’ voices in the WHO ANC guideline

## Slide 2
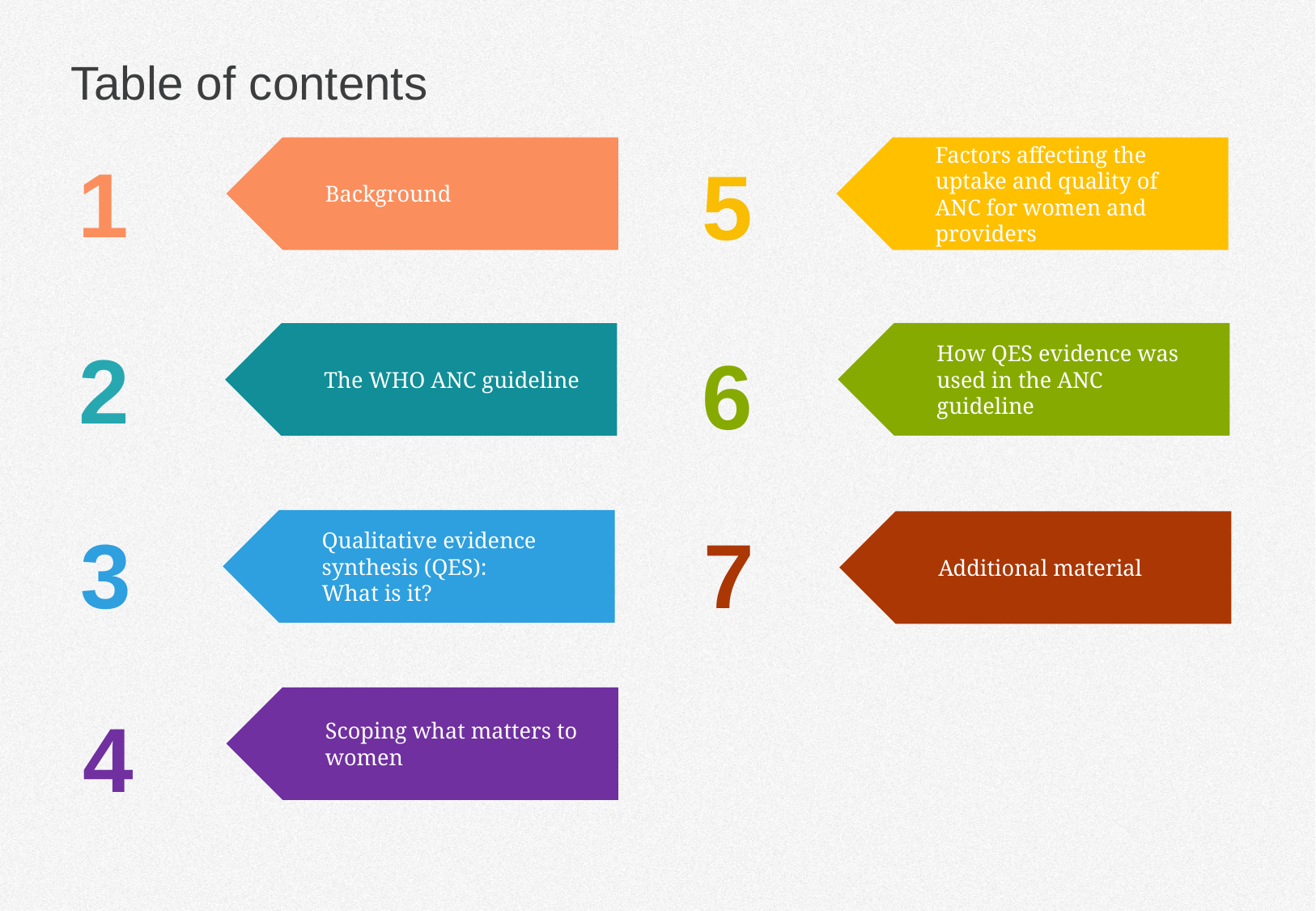

Table of contents
1
5
Background
Factors affecting the uptake and quality of ANC for women and providers
2
How QES evidence was used in the ANC guideline
6
The WHO ANC guideline
3
7
Qualitative evidence synthesis (QES):
What is it?
Additional material
4
Scoping what matters to women

## Slide 3
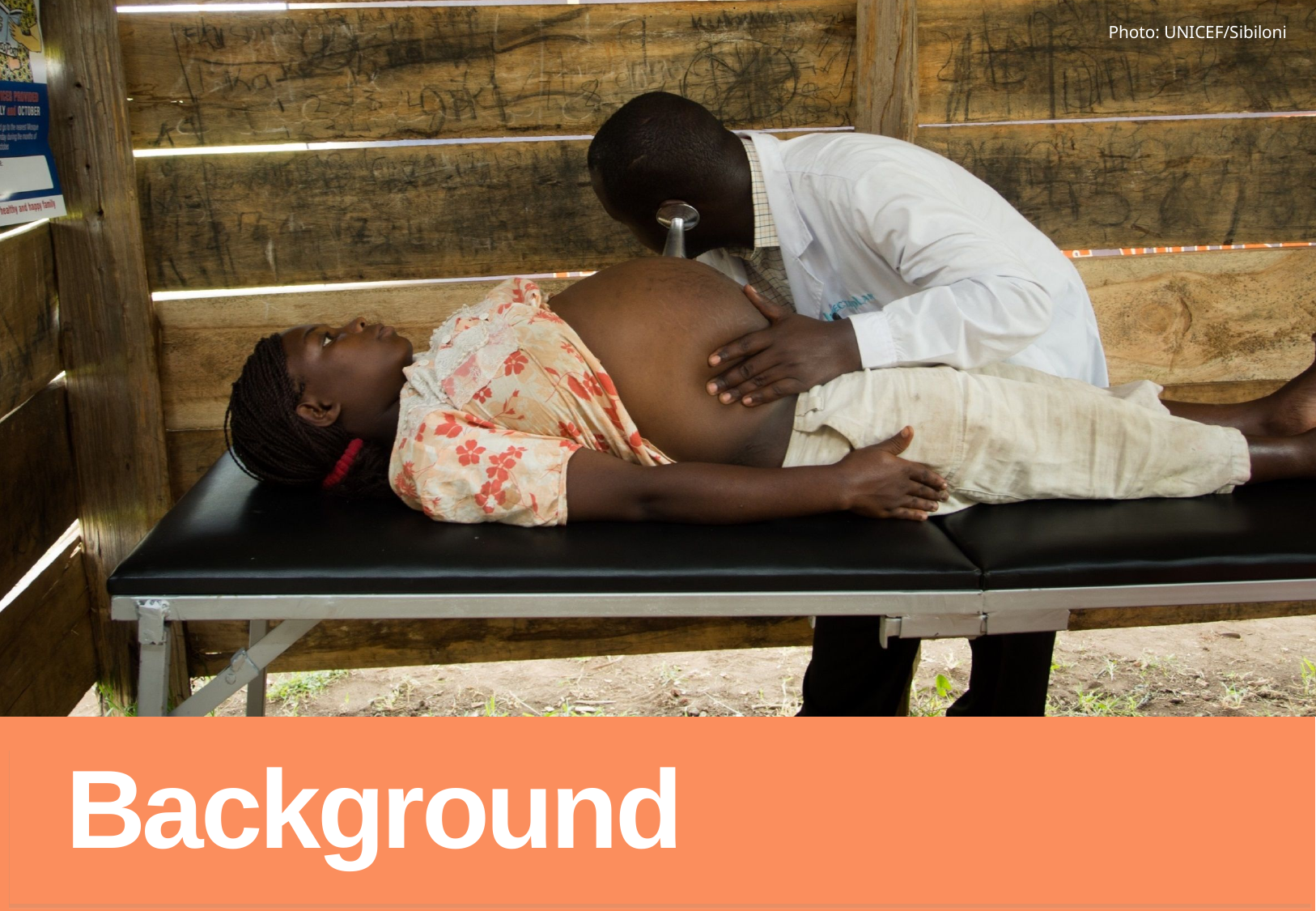

Photo: UNICEF/Sibiloni
Photo source XXXXXXX XXXXXX
# Background

## Slide 4
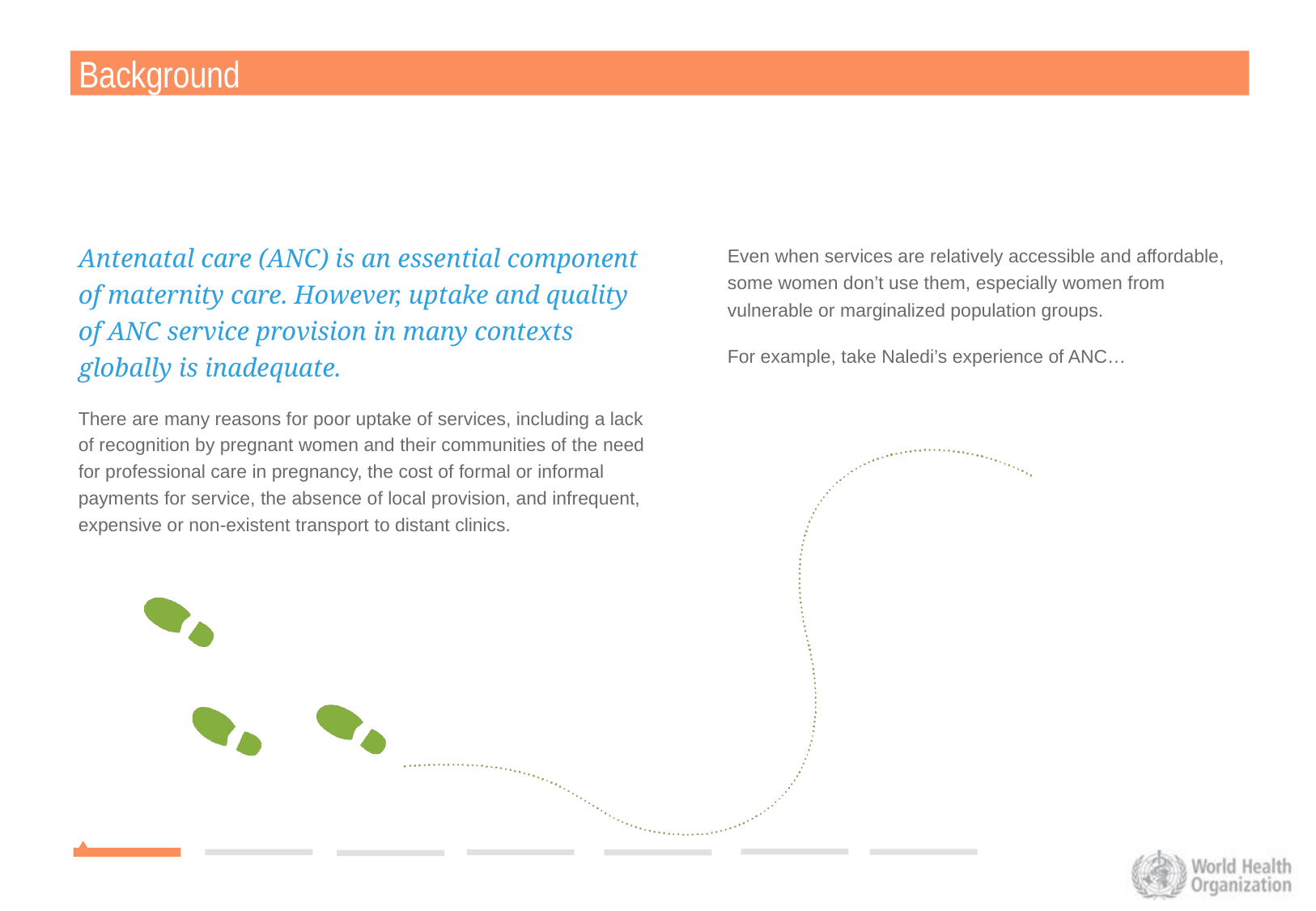

#
 Background
Antenatal care (ANC) is an essential component of maternity care. However, uptake and quality of ANC service provision in many contexts globally is inadequate.
There are many reasons for poor uptake of services, including a lack of recognition by pregnant women and their communities of the need for professional care in pregnancy, the cost of formal or informal payments for service, the absence of local provision, and infrequent, expensive or non-existent transport to distant clinics.
Even when services are relatively accessible and affordable, some women don’t use them, especially women from vulnerable or marginalized population groups.
For example, take Naledi’s experience of ANC…

## Slide 5
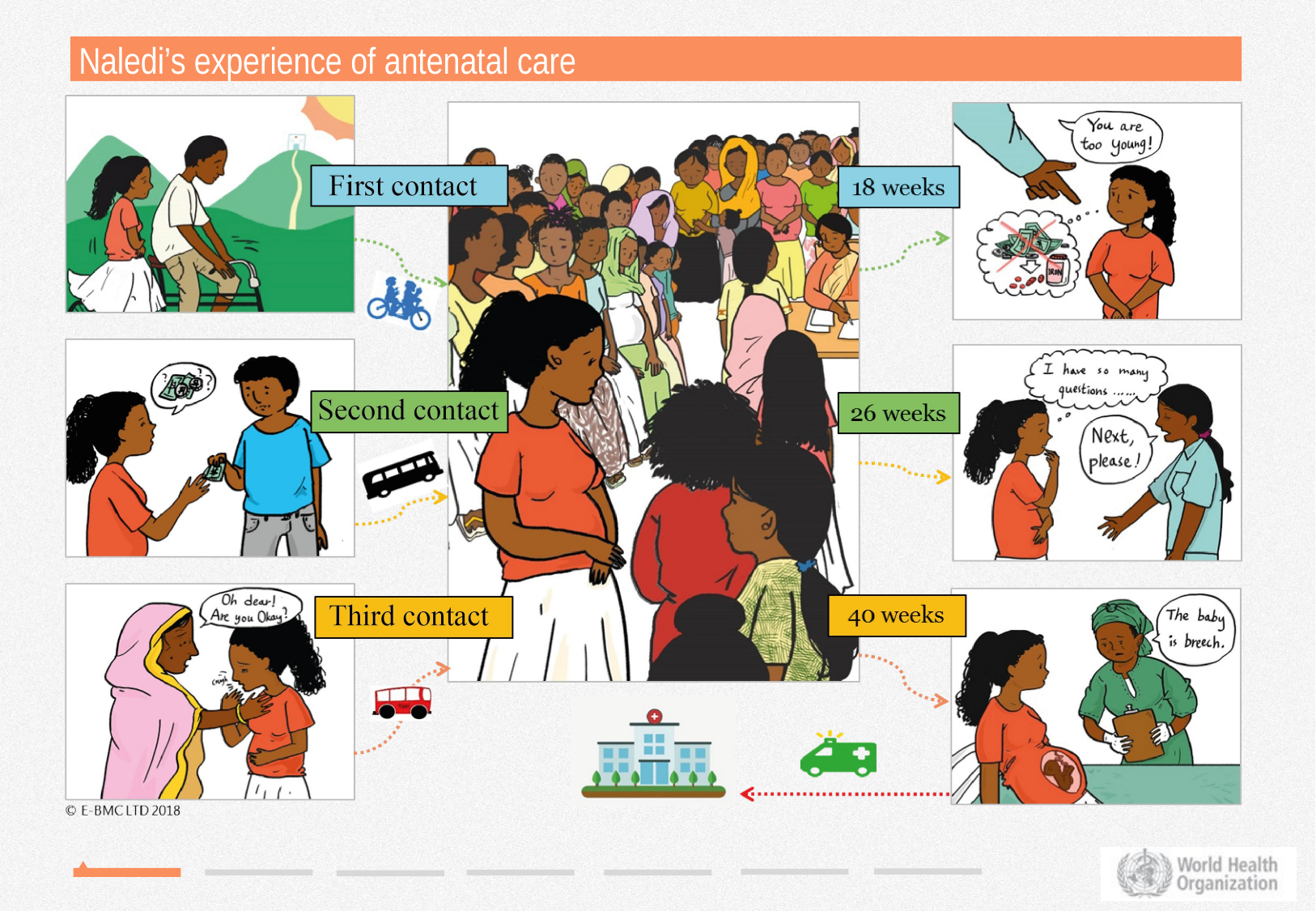

Naledi’s experience of antenatal care

## Slide 6
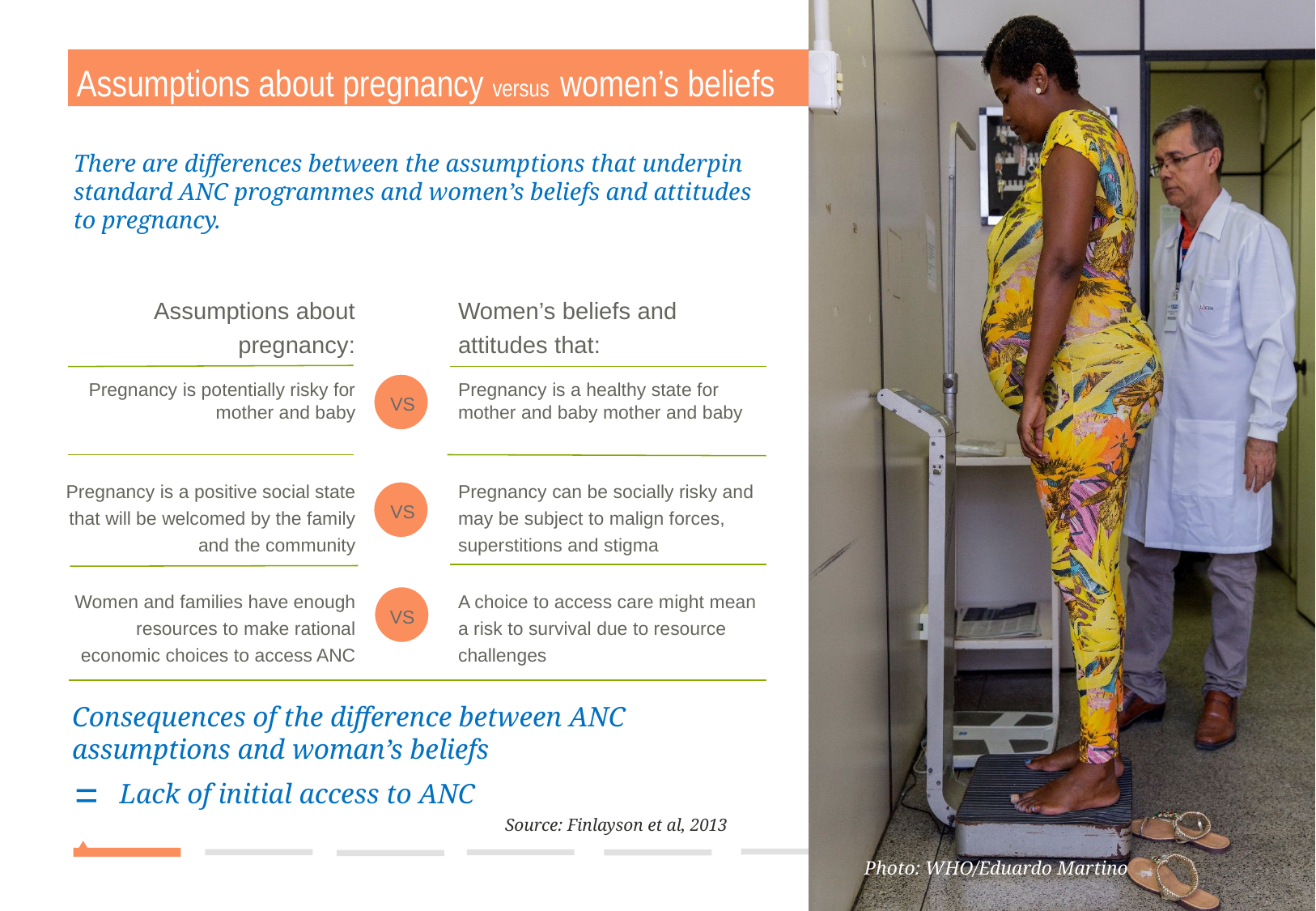

*
 Assumptions about pregnancy versus women’s beliefs
There are differences between the assumptions that underpin standard ANC programmes and women’s beliefs and attitudes to pregnancy.
Assumptions about pregnancy:
Pregnancy is potentially risky for mother and baby
Pregnancy is a positive social state that will be welcomed by the family and the community
Women and families have enough resources to make rational economic choices to access ANC choices to access ANC
Women’s beliefs and attitudes that:
Pregnancy is a healthy state for mother and baby mother and baby
Pregnancy can be socially risky and may be subject to malign forces, superstitions and stigma
A choice to access care might mean a risk to survival due to resource challenges
VS
VS
VS
Consequences of the difference between ANC assumptions and woman’s beliefs
=
Lack of initial access to ANC
Source: Finlayson et al, 2013
Photo: Flickr
Photo: WHO/Eduardo Martino
Photo: Flickr
Photo : © Adobe Stock
Photo: Dominic Chavez / World Bank

## Slide 7
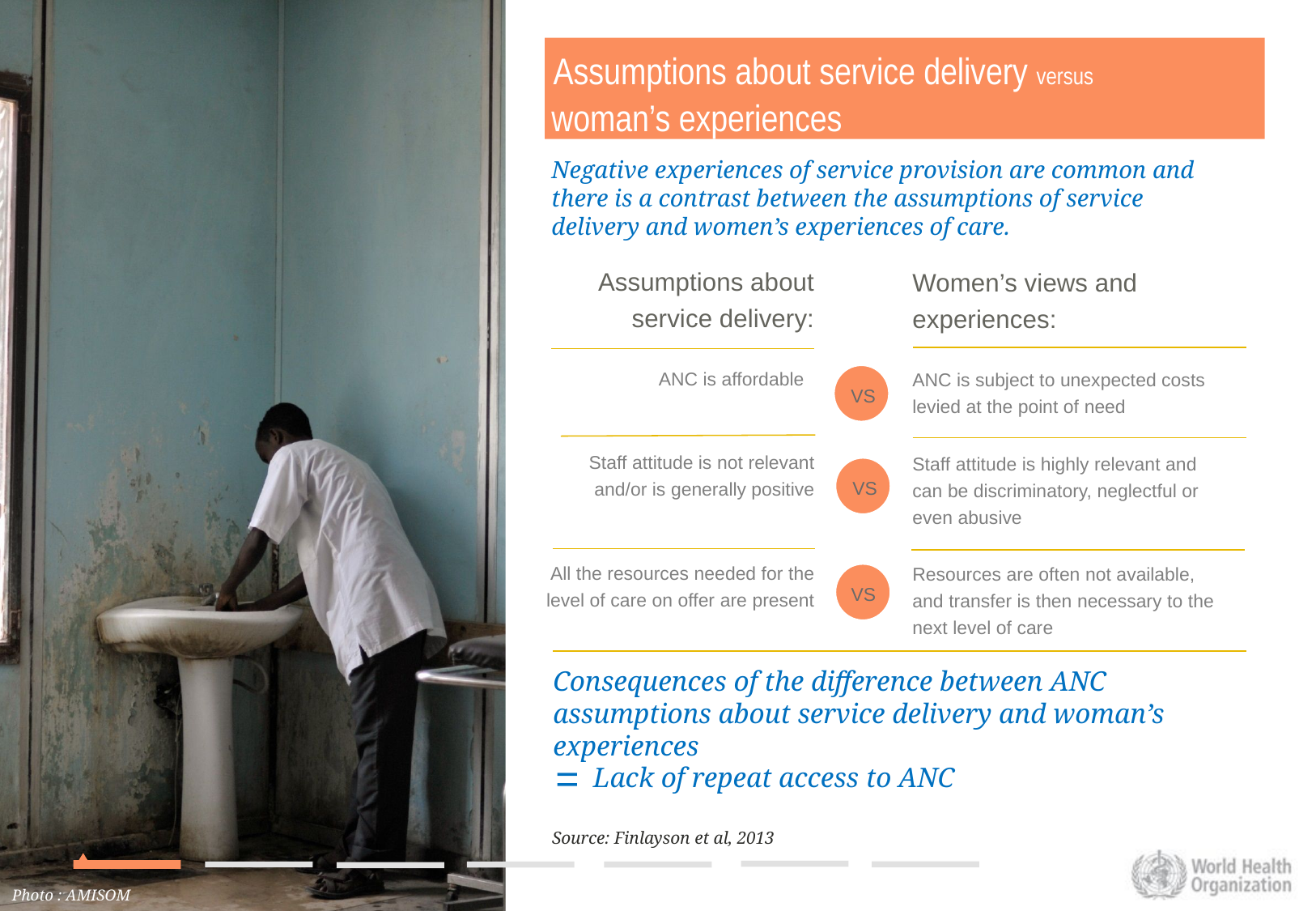

Assumptions about service delivery versus
iwoman’s experiences
Negative experiences of service provision are common and there is a contrast between the assumptions of service delivery and women’s experiences of care.
Assumptions about service delivery:
The story of he ANC is affordable f you
The Staff attitude is not relevant always and/or is generally positive and
All the resources needed for the level of care on offer are present
Women’s views and experiences: about
ANC is subject to unexpected costs levied at the point of need
Staff attitude is highly relevant and can be discriminatory, neglectful or even abusive
Resources are often not available, and transfer is then necessary to the next level of care
VS
VS
VS
Consequences of the difference between ANC assumptions about service delivery and woman’s experiences
=
Lack of repeat access to ANC
Source: Finlayson et al, 2013
Photo : AMISOM

## Slide 8
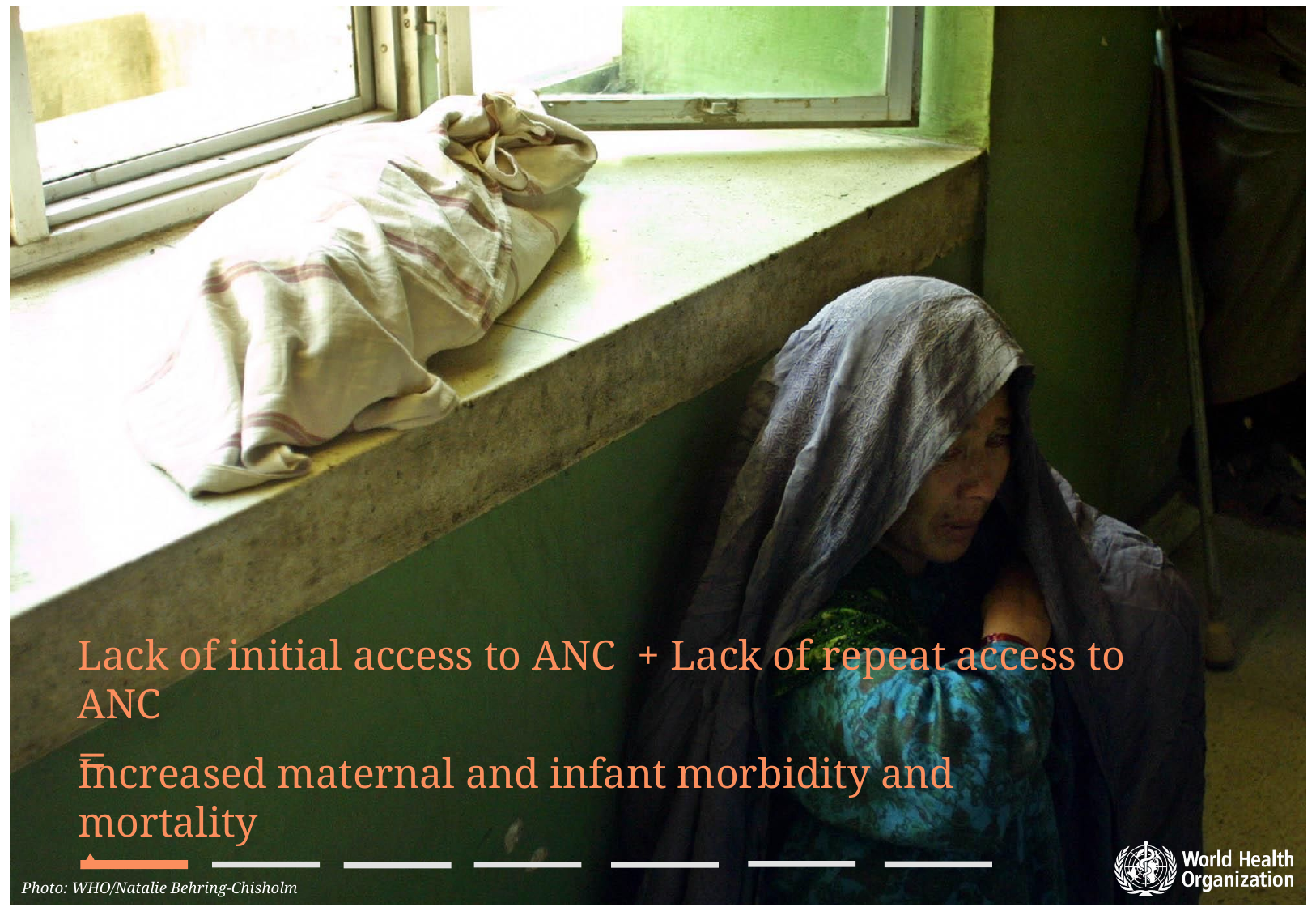

Lack of initial access to ANC + Lack of repeat access to ANC
=
Increased maternal and infant morbidity and mortality
Photo: WHO/Natalie Behring-Chisholm

## Slide 9
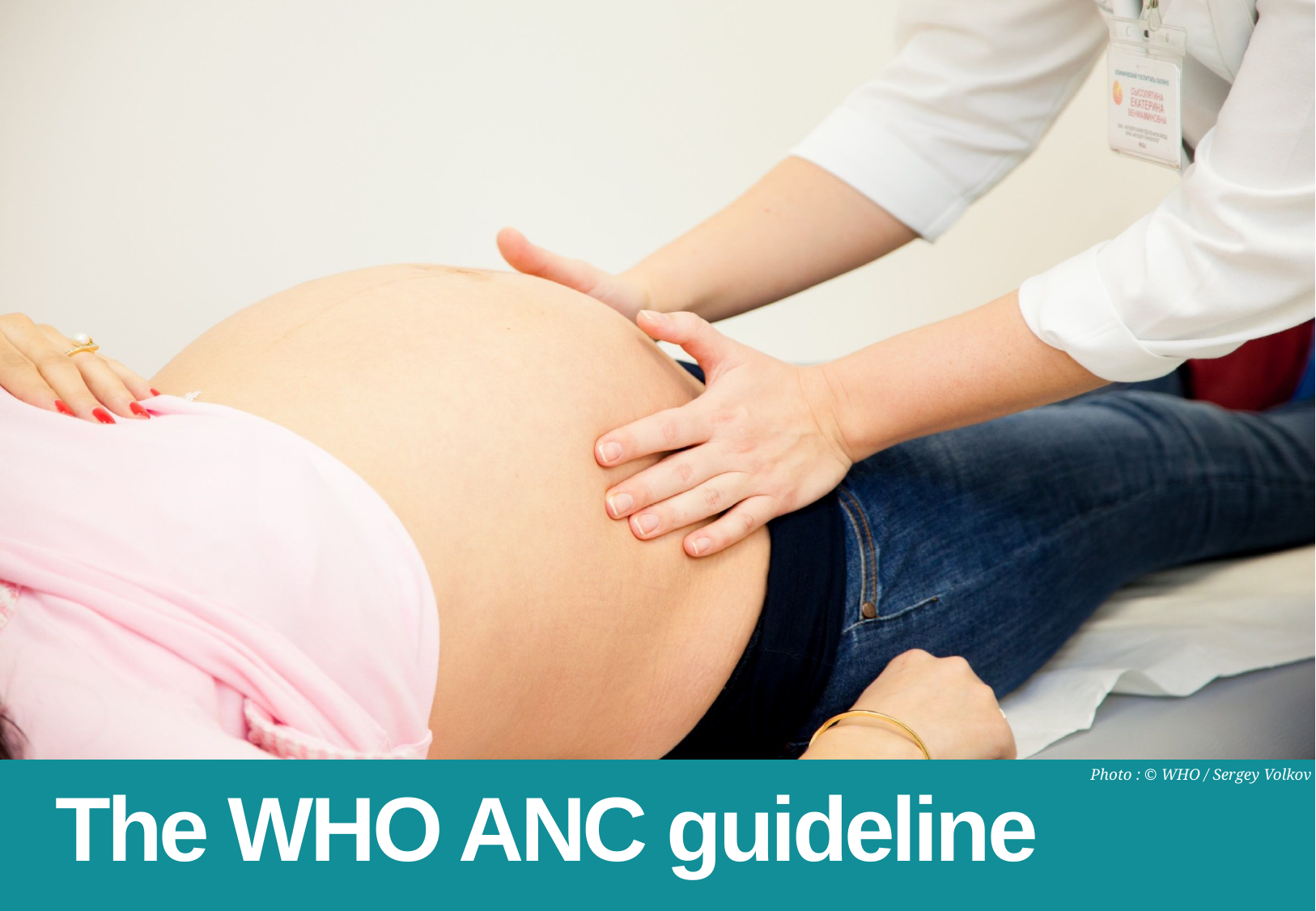

#
 The WHO ANC guideline
Photo : © WHO / Sergey Volkov

## Slide 10
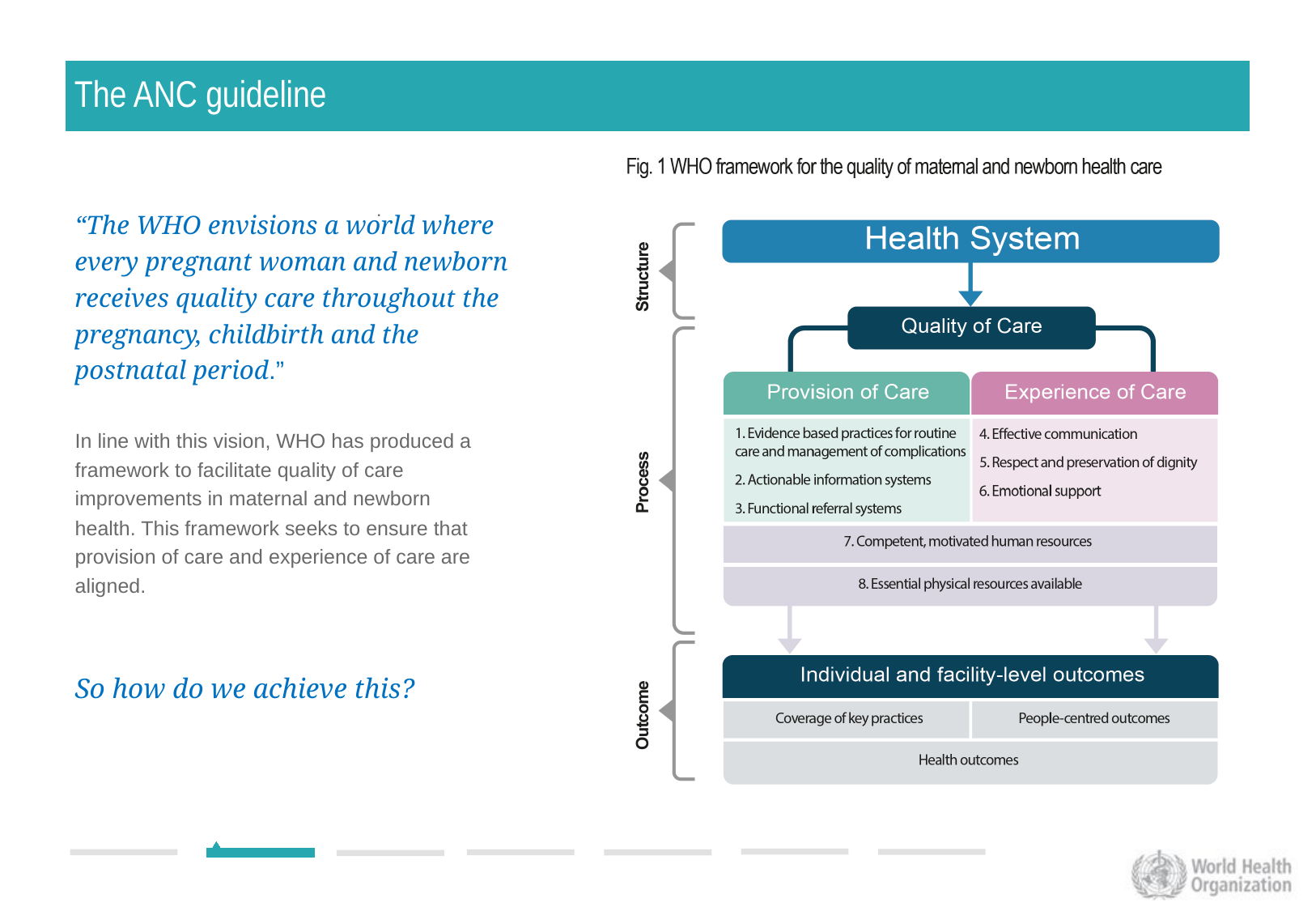

# The ANC guideline
.
“The WHO envisions a world where every pregnant woman and newborn receives quality care throughout the pregnancy, childbirth and the postnatal period.”
In line with this vision, WHO has produced a framework to facilitate quality of care improvements in maternal and newborn health. This framework seeks to ensure that provision of care and experience of care are aligned.
So how do we achieve this?

## Slide 11
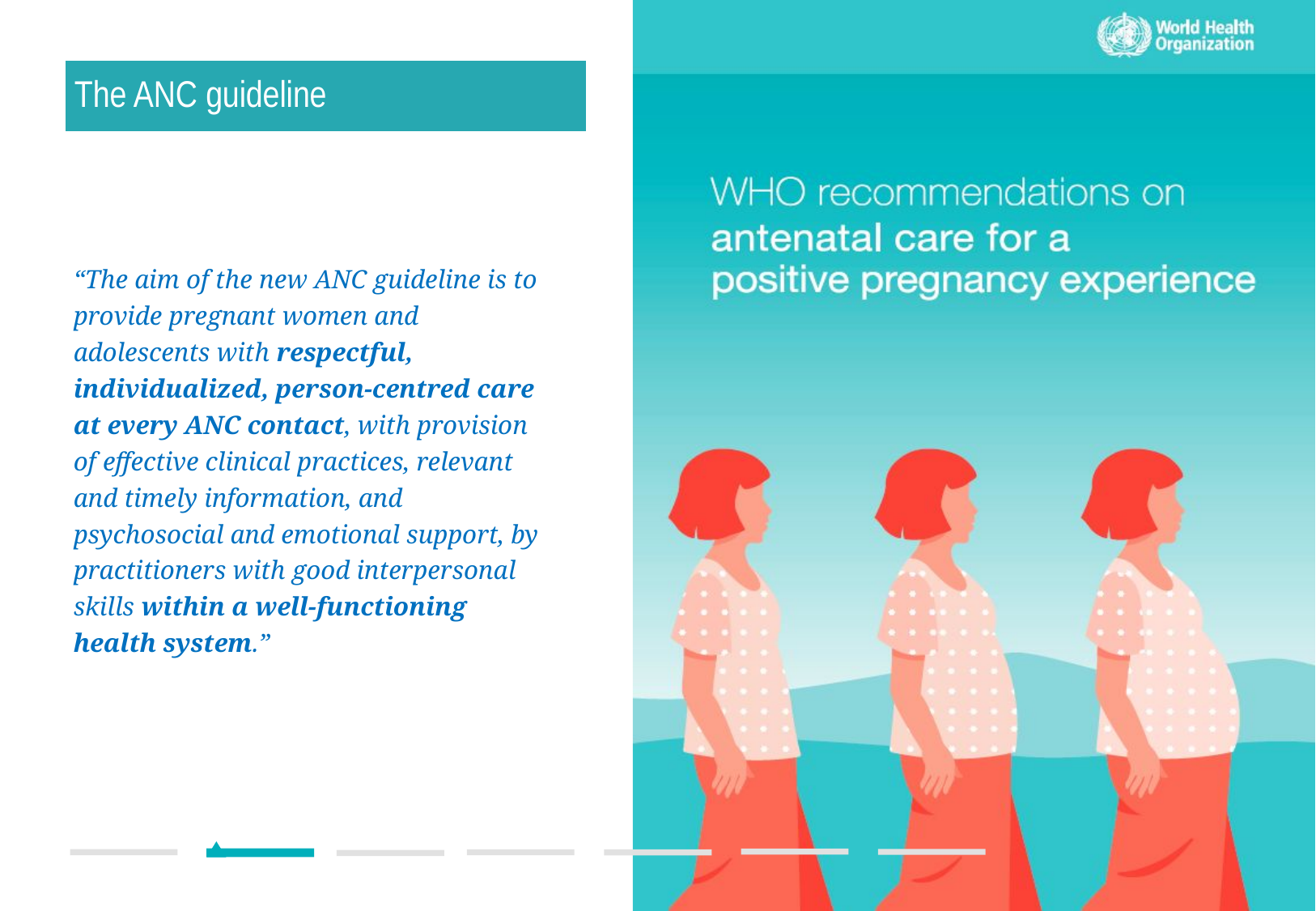

# The ANC guideline
“The aim of the new ANC guideline is to provide pregnant women and adolescents with respectful, individualized, person-centred care at every ANC contact, with provision of effective clinical practices, relevant and timely information, and psychosocial and emotional support, by practitioners with good interpersonal skills within a well-functioning health system.”

## Slide 12
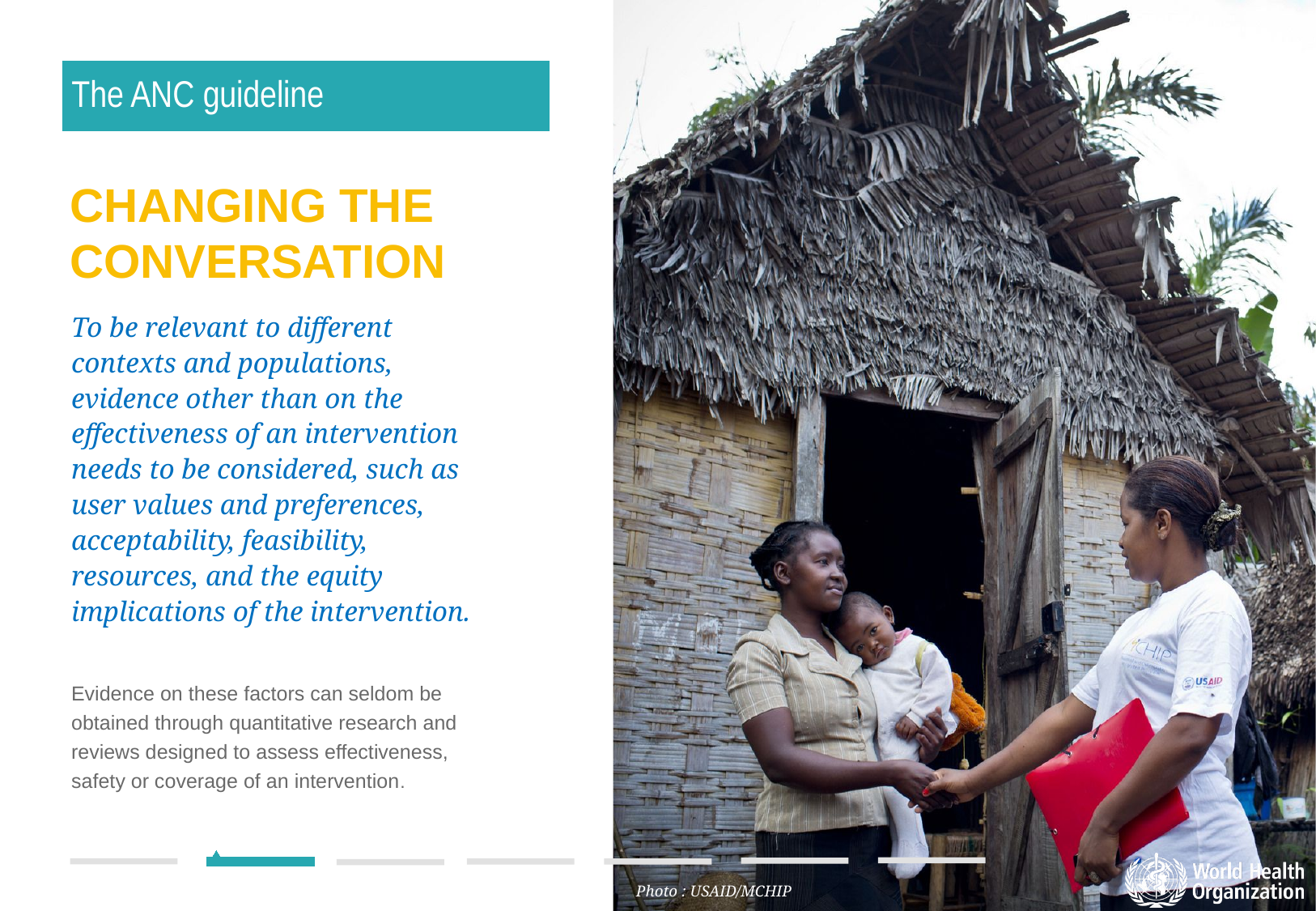

# The ANC guideline
CHANGING THE
CONVERSATION
To be relevant to different contexts and populations, evidence other than on the effectiveness of an intervention needs to be considered, such as user values and preferences, acceptability, feasibility, resources, and the equity implications of the intervention.
Evidence on these factors can seldom be obtained through quantitative research and reviews designed to assess effectiveness, safety or coverage of an intervention.
Photo : USAID/MCHIP

## Slide 13
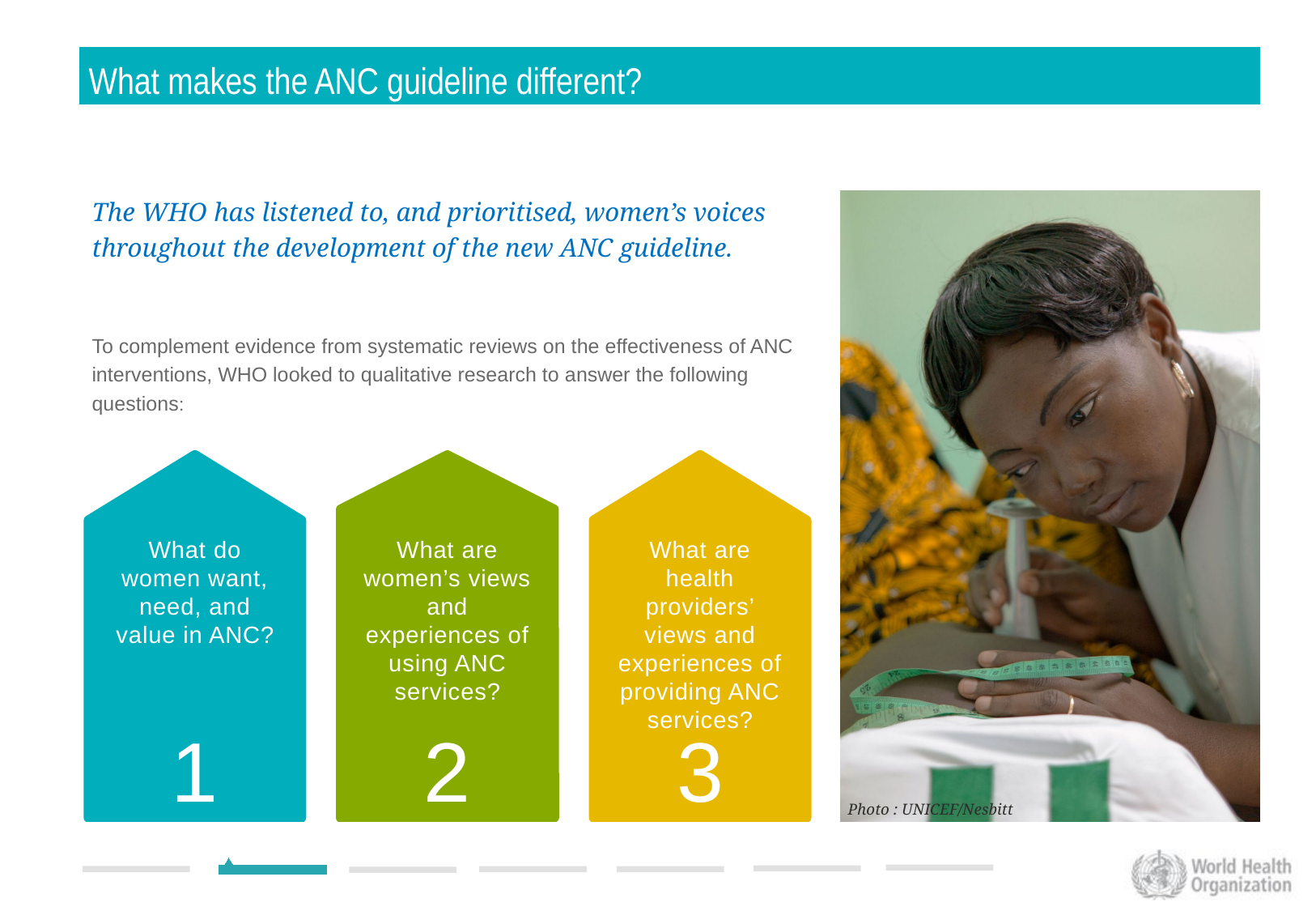

What makes the ANC guideline different?
The WHO has listened to, and prioritised, women’s voices throughout the development of the new ANC guideline.
To complement evidence from systematic reviews on the effectiveness of ANC interventions, WHO looked to qualitative research to answer the following questions:
What do women want, need, and value in ANC?
What are women’s views and experiences of using ANC services?
What are health providers’ views and experiences of providing ANC services?
1
2
3
Photo : UNICEF/Nesbitt

## Slide 14
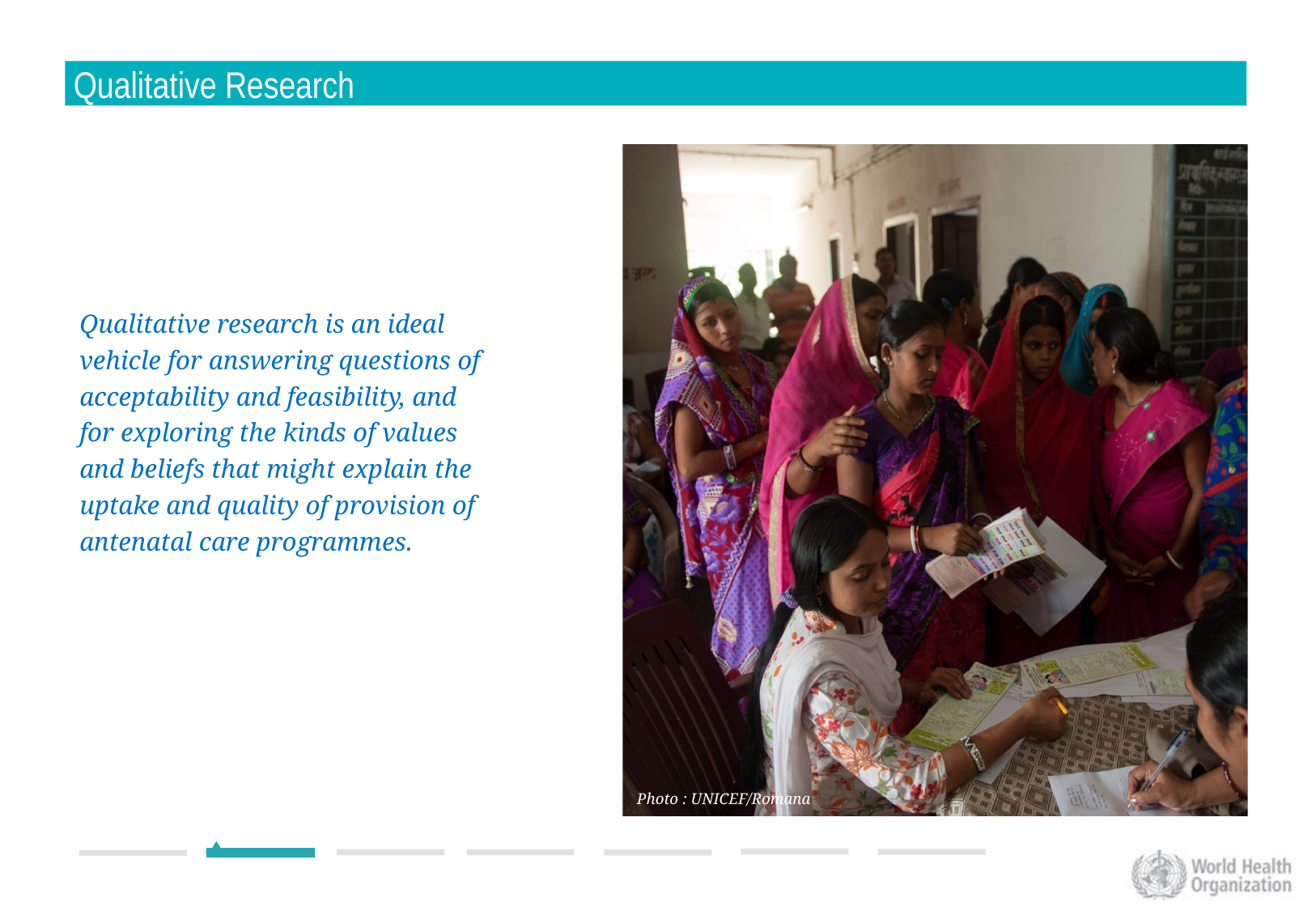

Qualitative Research
Qualitative research is an ideal vehicle for answering questions of acceptability and feasibility, and for exploring the kinds of values and beliefs that might explain the uptake and quality of provision of antenatal care programmes.
Photo : UNICEF/Romana
Photo XXXXXX XXXXXX

## Slide 15
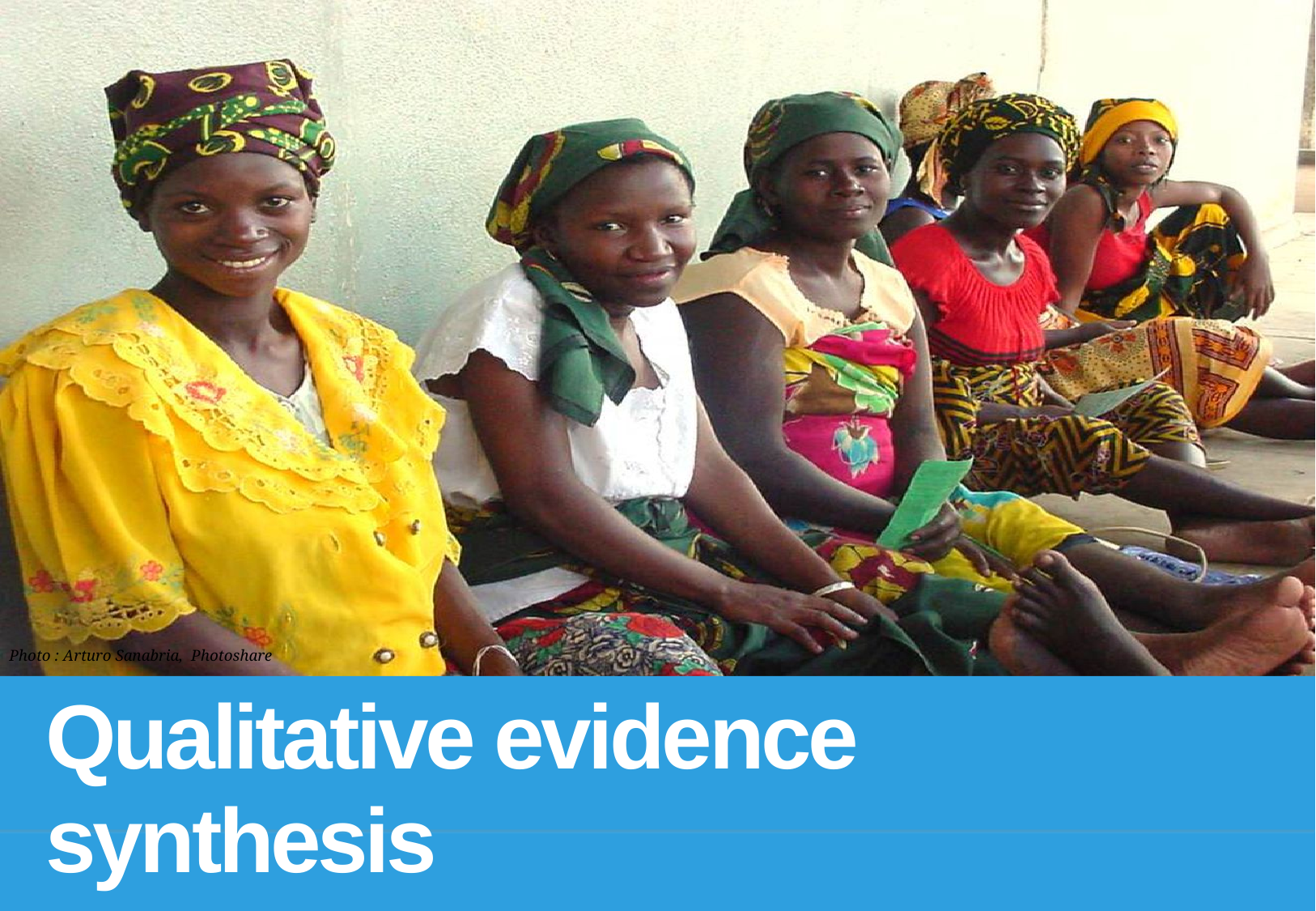

Photo : Arturo Sanabria, Photoshare
# Qualitative evidence synthesis

## Slide 16
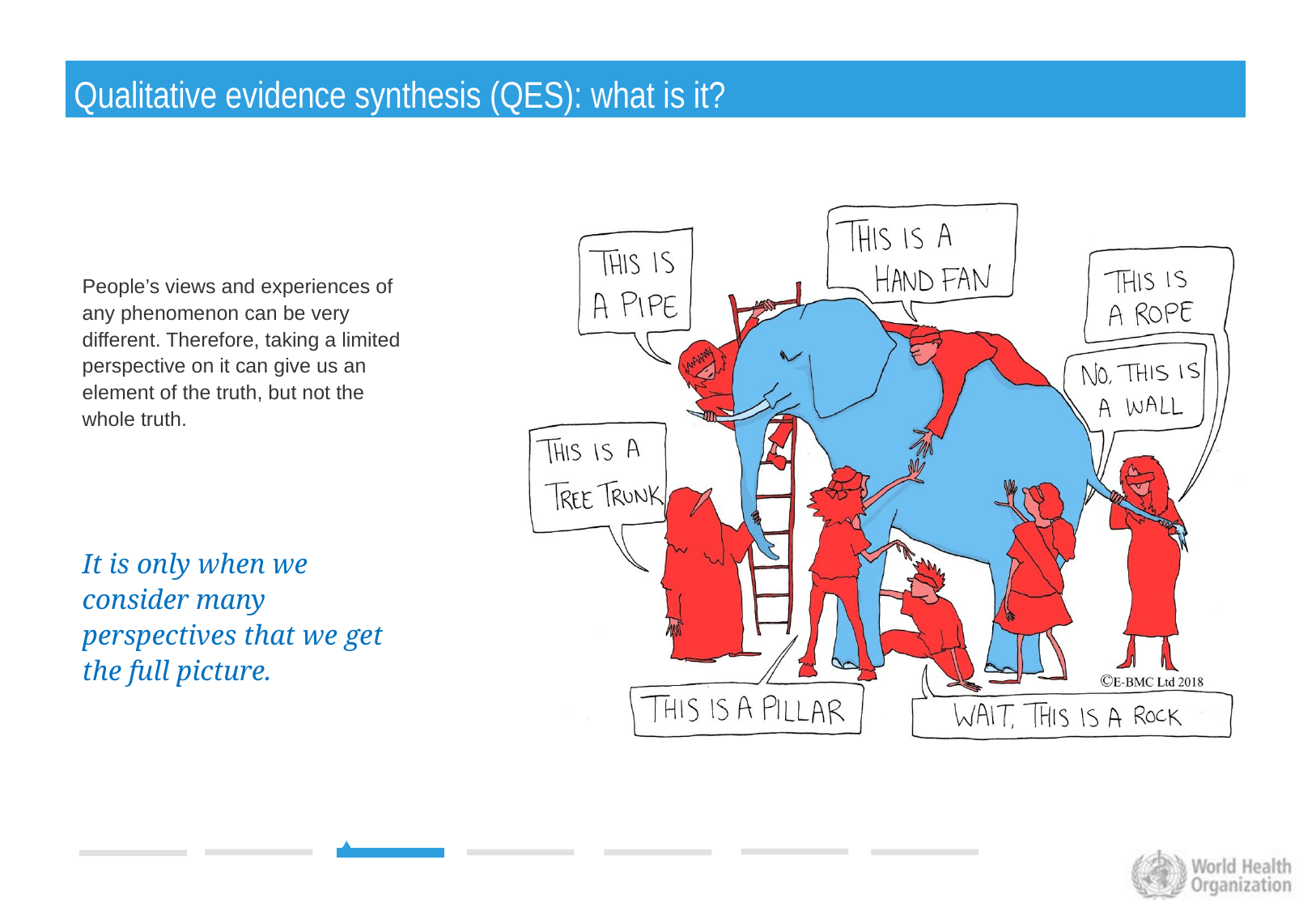

Qualitative evidence synthesis (QES): what is it?
People’s views and experiences of any phenomenon can be very different. Therefore, taking a limited perspective on it can give us an element of the truth, but not the whole truth.
It is only when we consider many perspectives that we get the full picture.

## Slide 17
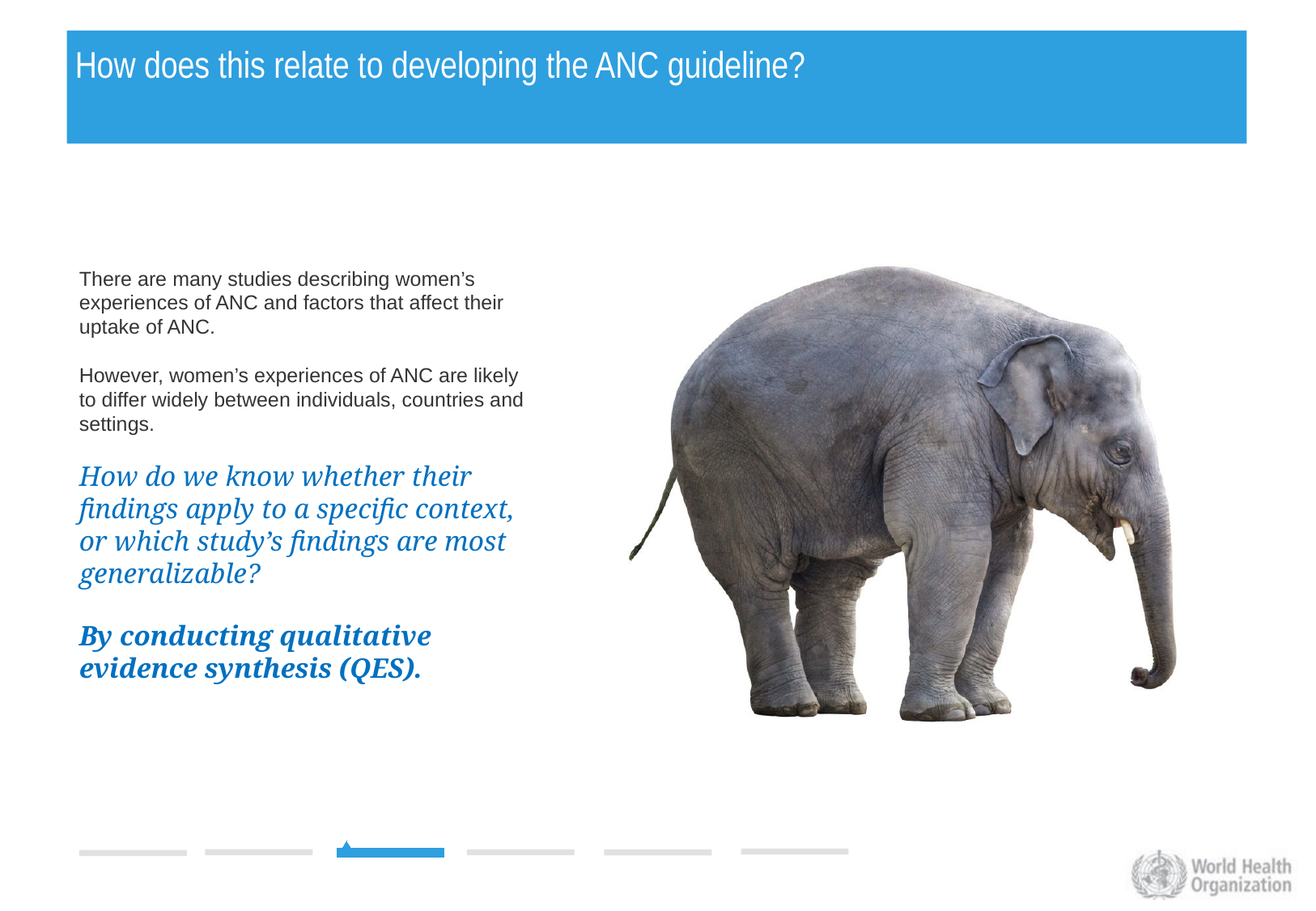

How does this relate to developing the ANC guideline?
There are many studies describing women’s experiences of ANC and factors that affect their uptake of ANC.
However, women’s experiences of ANC are likely to differ widely between individuals, countries and settings.
How do we know whether their findings apply to a specific context, or which study’s findings are most generalizable?
By conducting qualitative evidence synthesis (QES).

## Slide 18
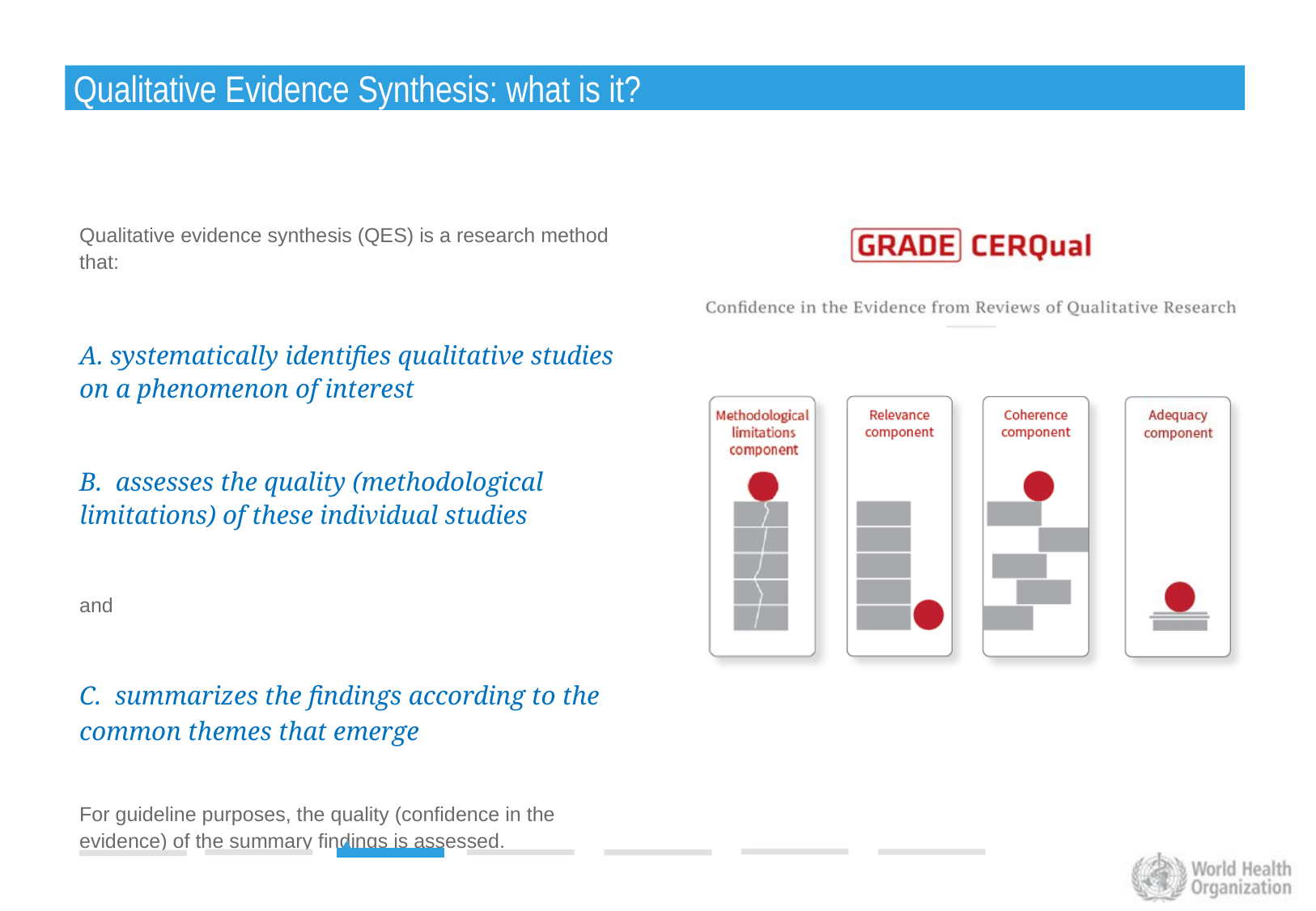

Qualitative Evidence Synthesis: what is it?
www.cerqual.org
Qualitative evidence synthesis (QES) is a research method that:
A. systematically identifies qualitative studies on a phenomenon of interest
B. assesses the quality (methodological limitations) of these individual studies
and
C. summarizes the findings according to the common themes that emerge
For guideline purposes, the quality (confidence in the evidence) of the summary findings is assessed.

## Slide 19
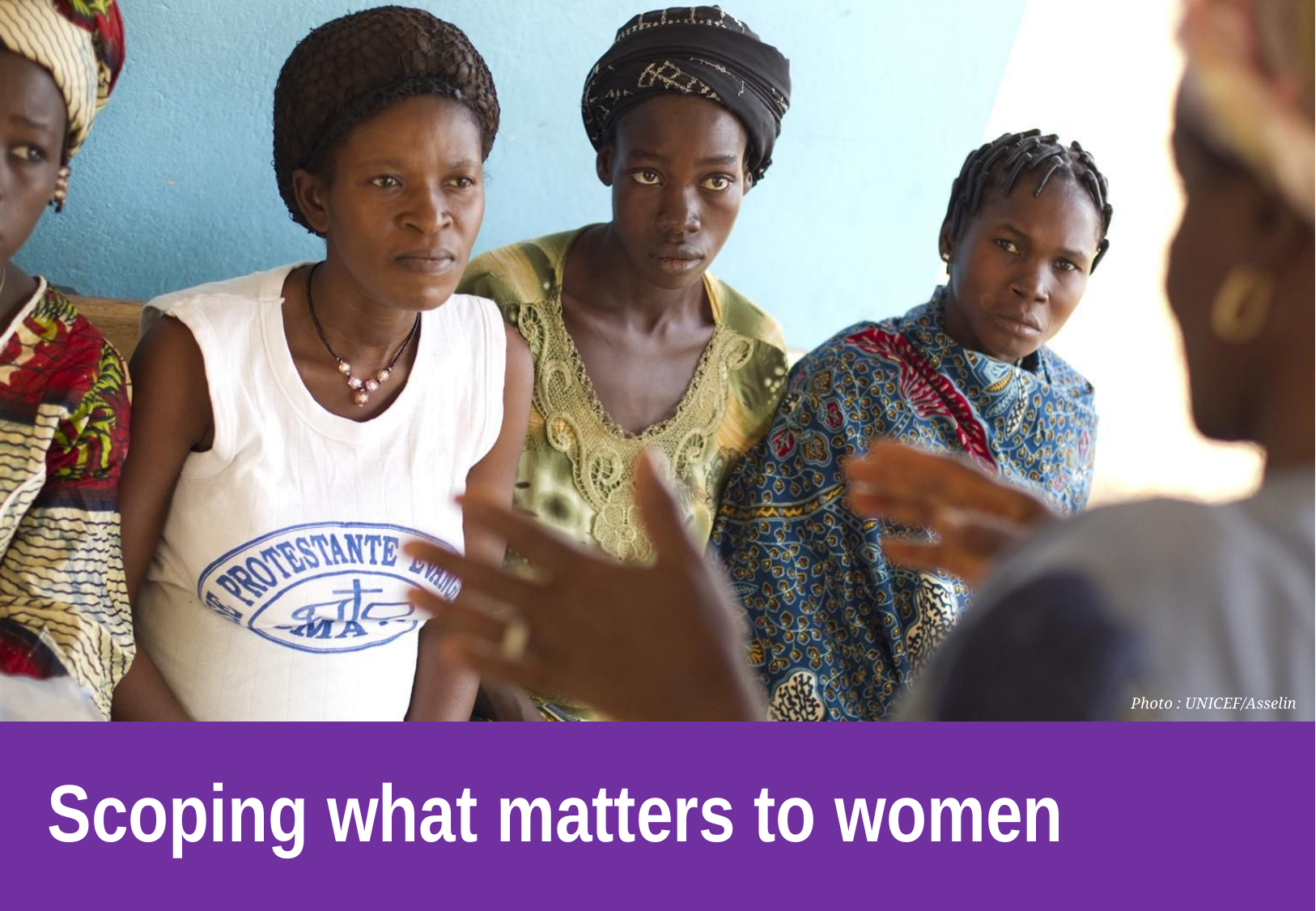

Photo : UNICEF/Asselin
Scoping what matters to women

## Slide 20
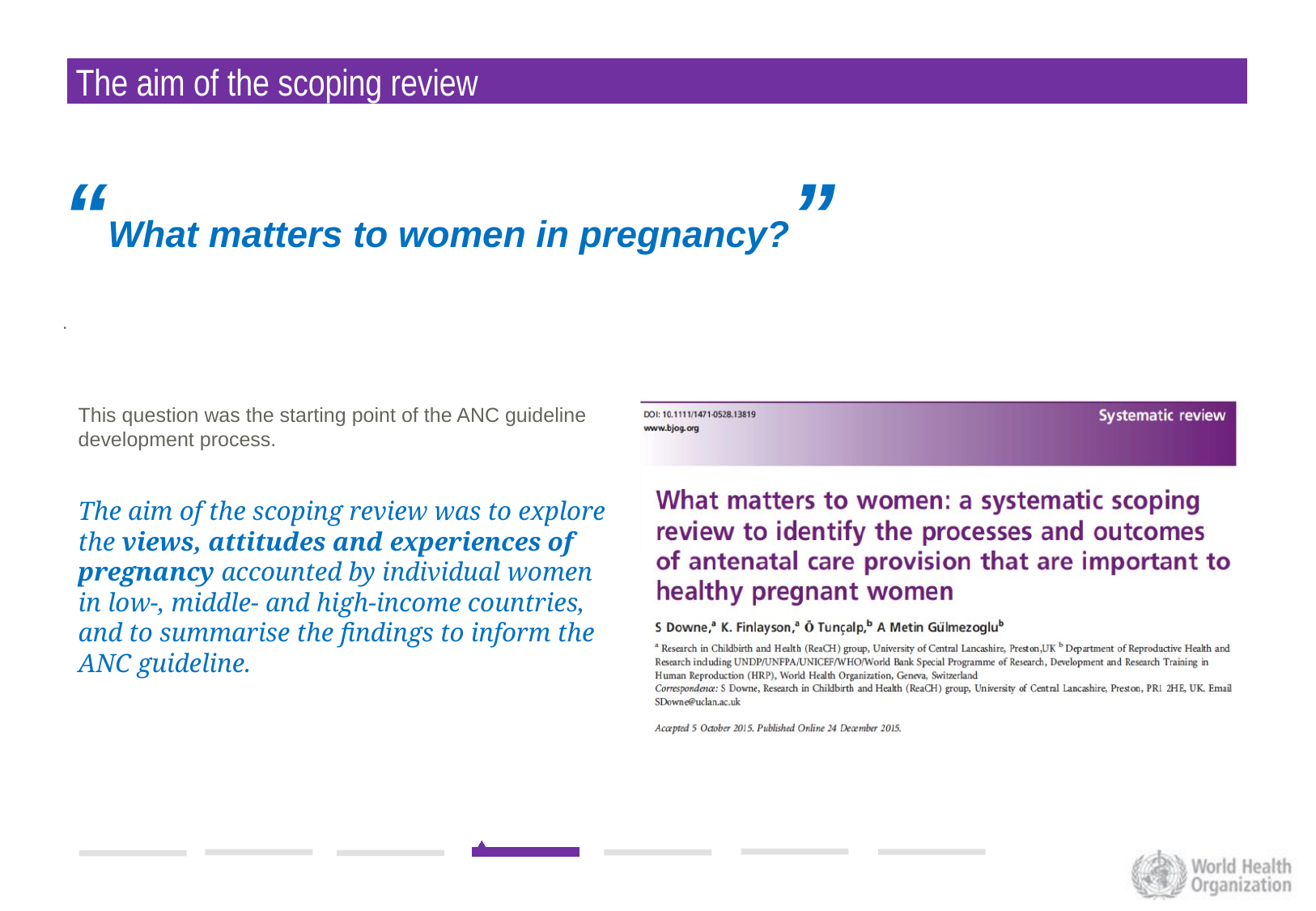

The aim of the scoping review
“What matters to women in pregnancy?”
.
This question was the starting point of the ANC guideline development process.
The aim of the scoping review was to explore the views, attitudes and experiences of pregnancy accounted by individual women in low-, middle- and high-income countries, and to summarise the findings to inform the ANC guideline.
“
Women around the world want ANC staff and services to help them achieve a positive pregnancy experience.
”

## Slide 21
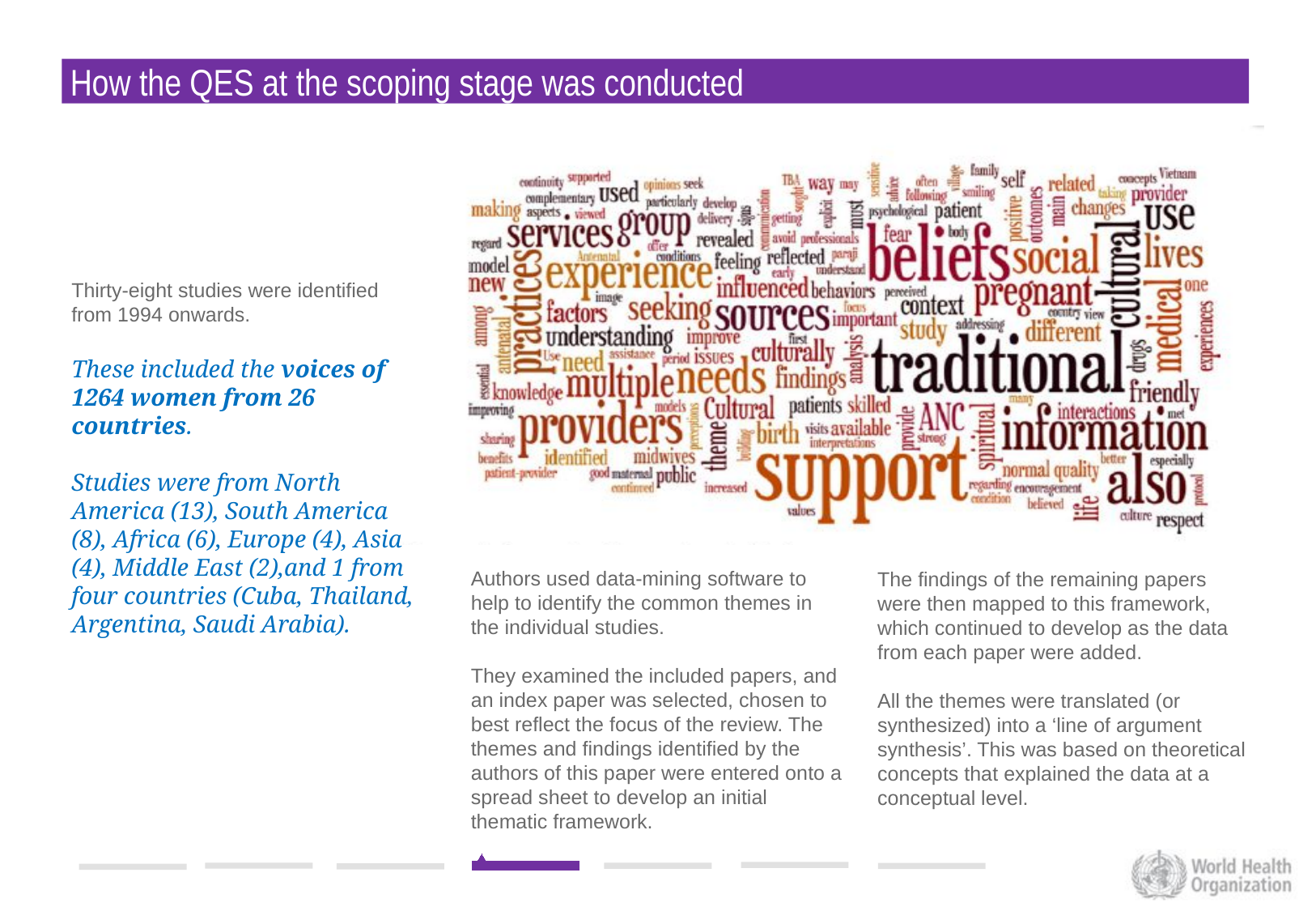

How the QES at the scoping stage was conducted
Thirty-eight studies were identified from 1994 onwards.
These included the voices of 1264 women from 26 countries.
Studies were from North America (13), South America (8), Africa (6), Europe (4), Asia (4), Middle East (2),and 1 from four countries (Cuba, Thailand, Argentina, Saudi Arabia).
Authors used data-mining software to help to identify the common themes in the individual studies.
They examined the included papers, and an index paper was selected, chosen to best reflect the focus of the review. The themes and findings identified by the authors of this paper were entered onto a spread sheet to develop an initial thematic framework.
The findings of the remaining papers were then mapped to this framework, which continued to develop as the data from each paper were added.
All the themes were translated (or synthesized) into a ‘line of argument synthesis’. This was based on theoretical concepts that explained the data at a conceptual level.

## Slide 22
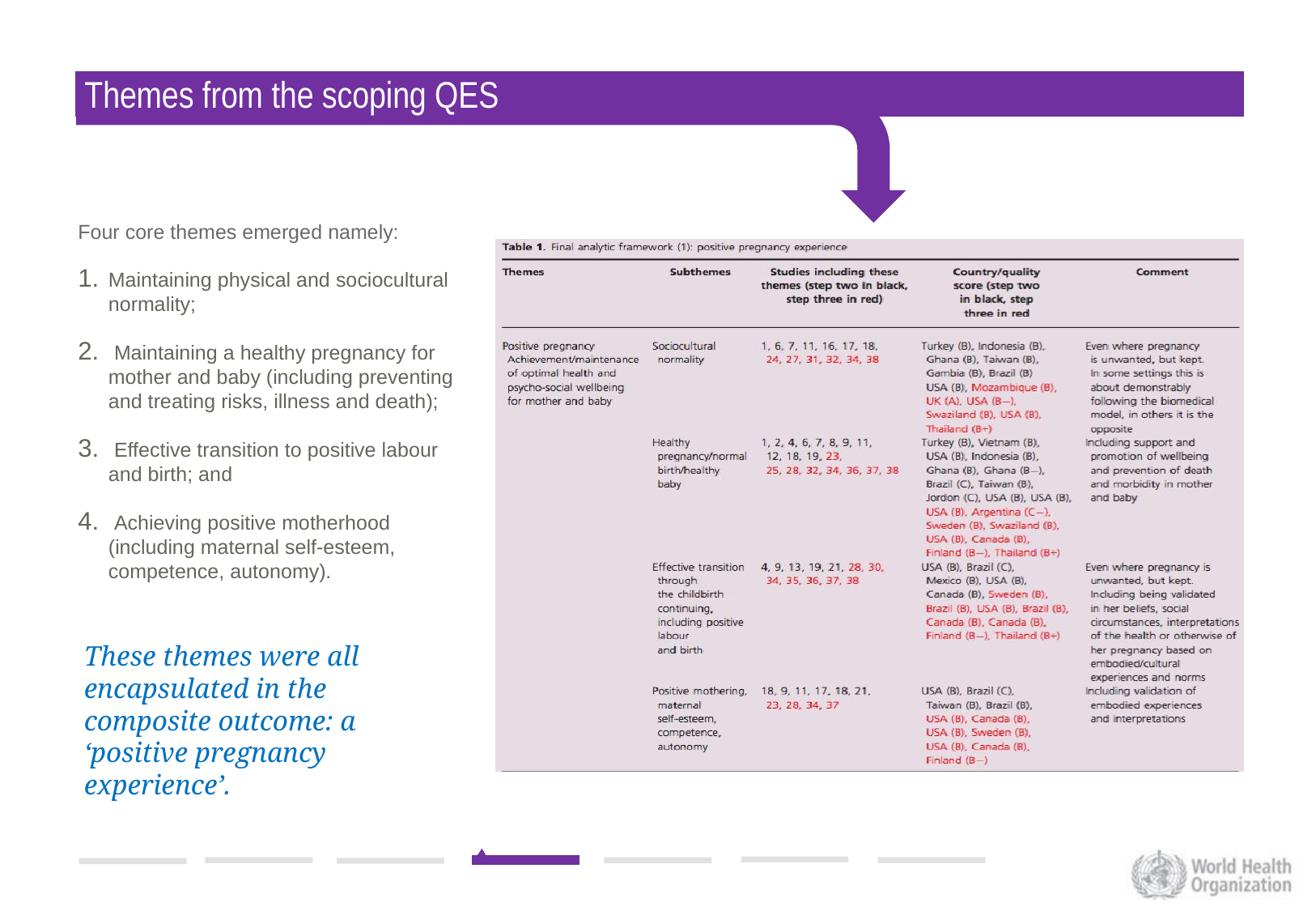

Themes from the scoping QES
Four core themes emerged namely:
Maintaining physical and sociocultural normality;
 Maintaining a healthy pregnancy for mother and baby (including preventing and treating risks, illness and death);
 Effective transition to positive labour and birth; and
 Achieving positive motherhood (including maternal self-esteem, competence, autonomy).
These themes were all encapsulated in the composite outcome: a ‘positive pregnancy experience’.

## Slide 23
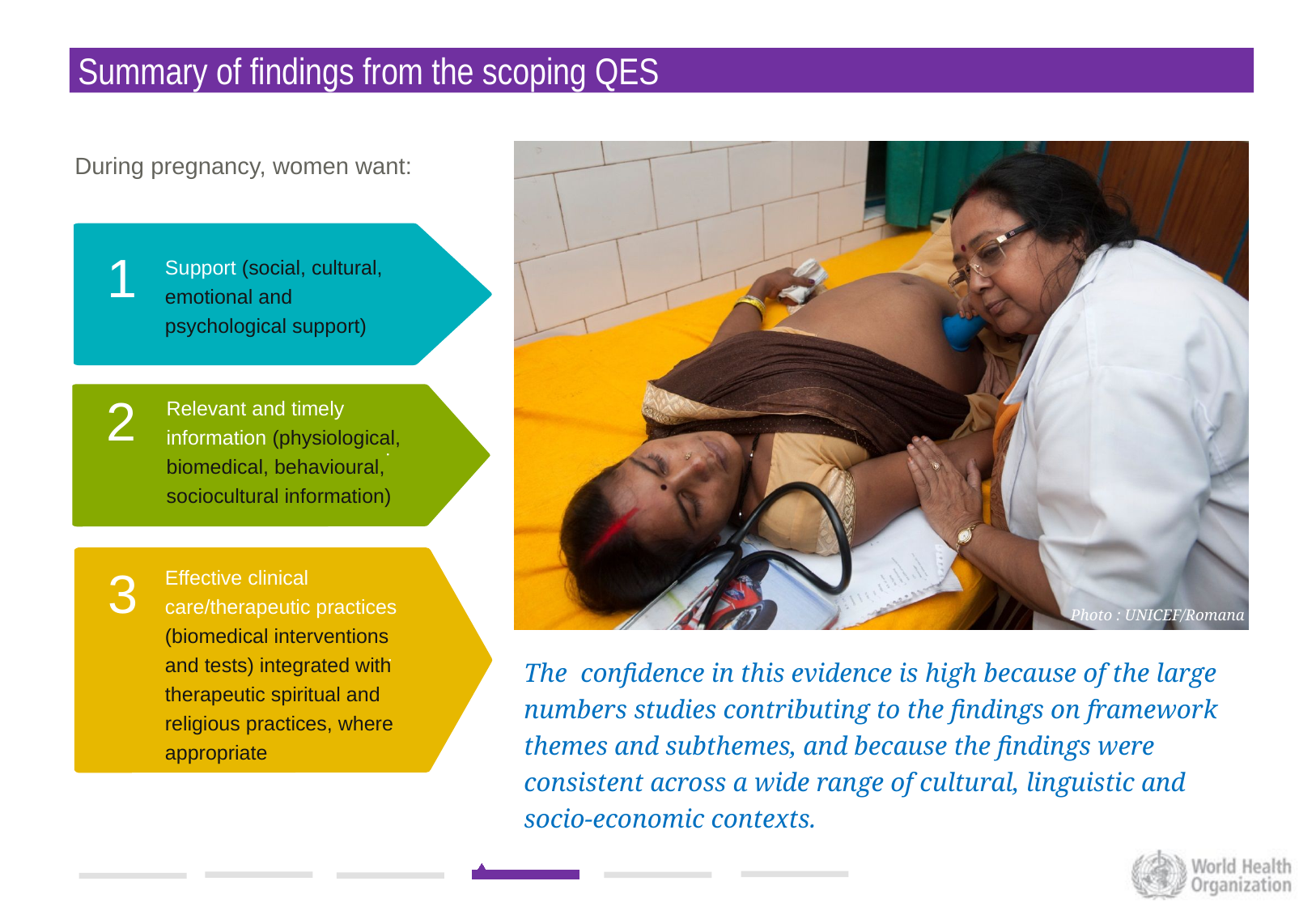

Summary of findings from the scoping QES
During pregnancy, women want:
1
Support (social, cultural, emotional and psychological support)
.
Relevant and timely information (physiological, biomedical, behavioural, sociocultural information)
2
Effective clinical care/therapeutic practices (biomedical interventions and tests) integrated with therapeutic spiritual and religious practices, where appropriate
3
.
Photo source XXXXXXX XXXXXX
Photo : UNICEF/Romana
The confidence in this evidence is high because of the large numbers studies contributing to the findings on framework themes and subthemes, and because the findings were consistent across a wide range of cultural, linguistic and socio-economic contexts.

## Slide 24
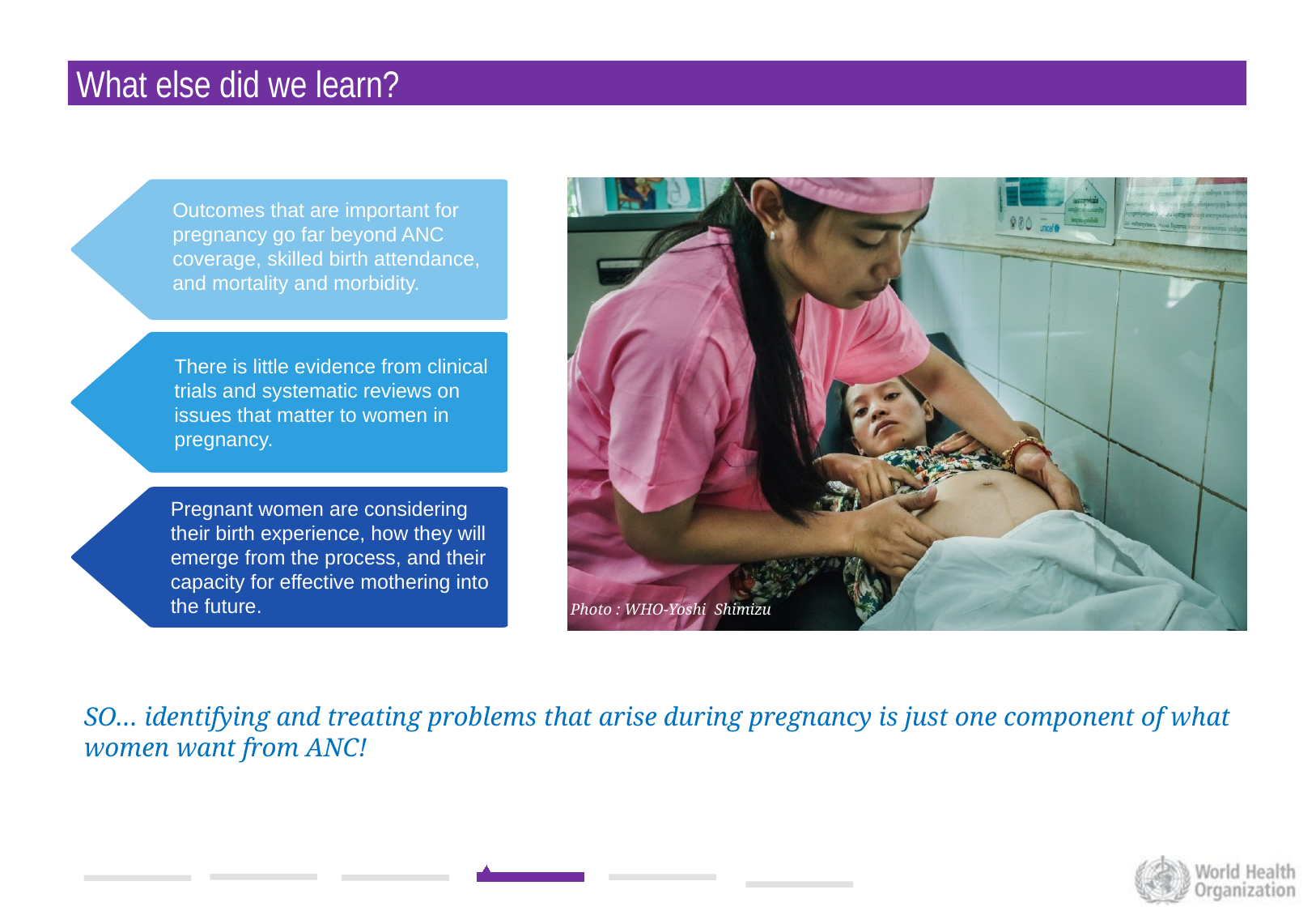

What else did we learn?
Photo : WHO-Yoshi Shimizu
Outcomes that are important for pregnancy go far beyond ANC coverage, skilled birth attendance, and mortality and morbidity.
.
There is little evidence from clinical trials and systematic reviews on issues that matter to women in pregnancy.
Pregnant women are considering their birth experience, how they will emerge from the process, and their capacity for effective mothering into the future.
SO… identifying and treating problems that arise during pregnancy is just one component of what women want from ANC!

## Slide 25
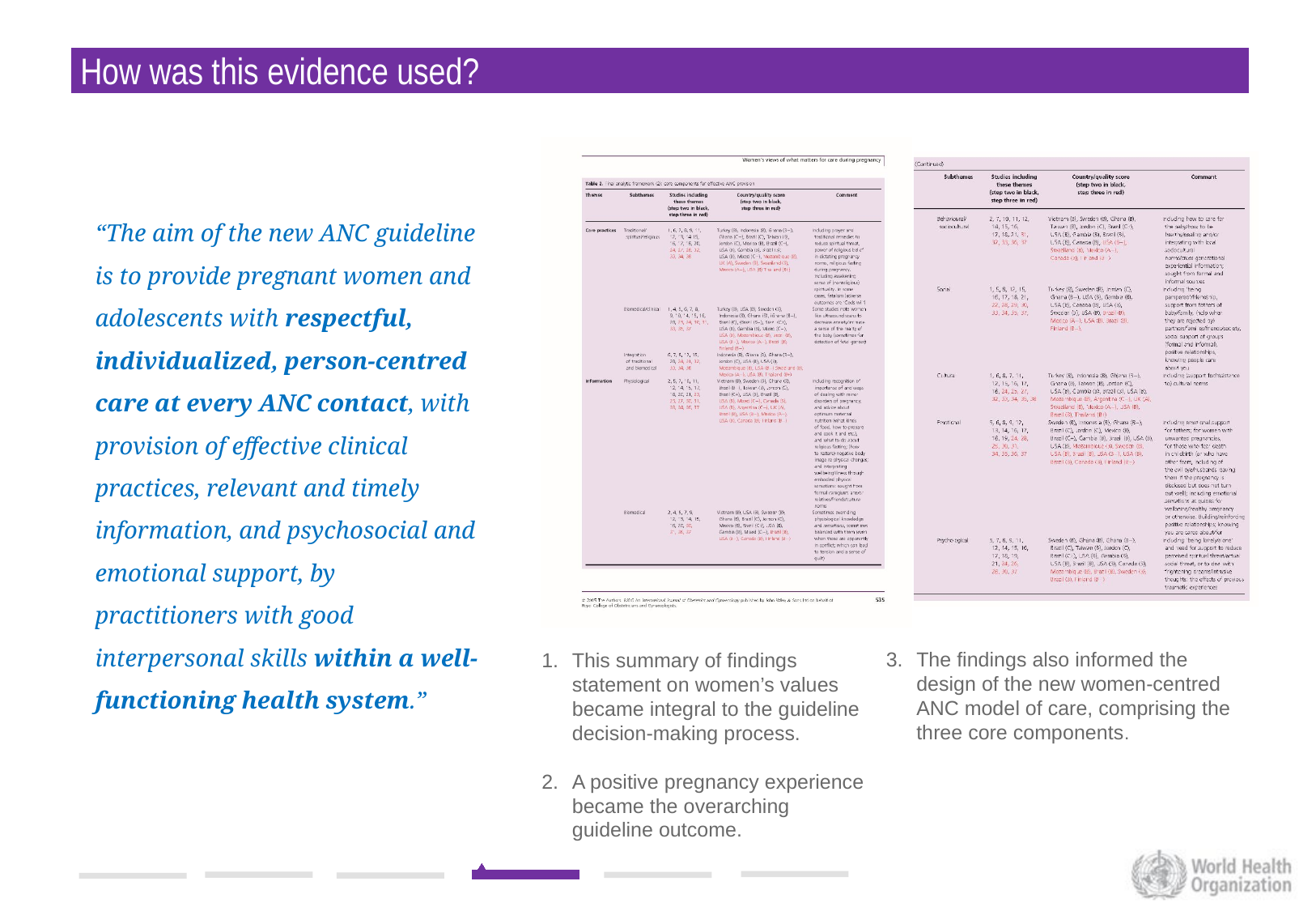

How was this evidence used?
“The aim of the new ANC guideline is to provide pregnant women and adolescents with respectful, individualized, person-centred care at every ANC contact, with provision of effective clinical practices, relevant and timely information, and psychosocial and emotional support, by practitioners with good interpersonal skills within a well-functioning health system.”
The findings also informed the design of the new women-centred ANC model of care, comprising the three core components.
This summary of findings statement on women’s values became integral to the guideline decision-making process.
A positive pregnancy experience became the overarching guideline outcome.

## Slide 26
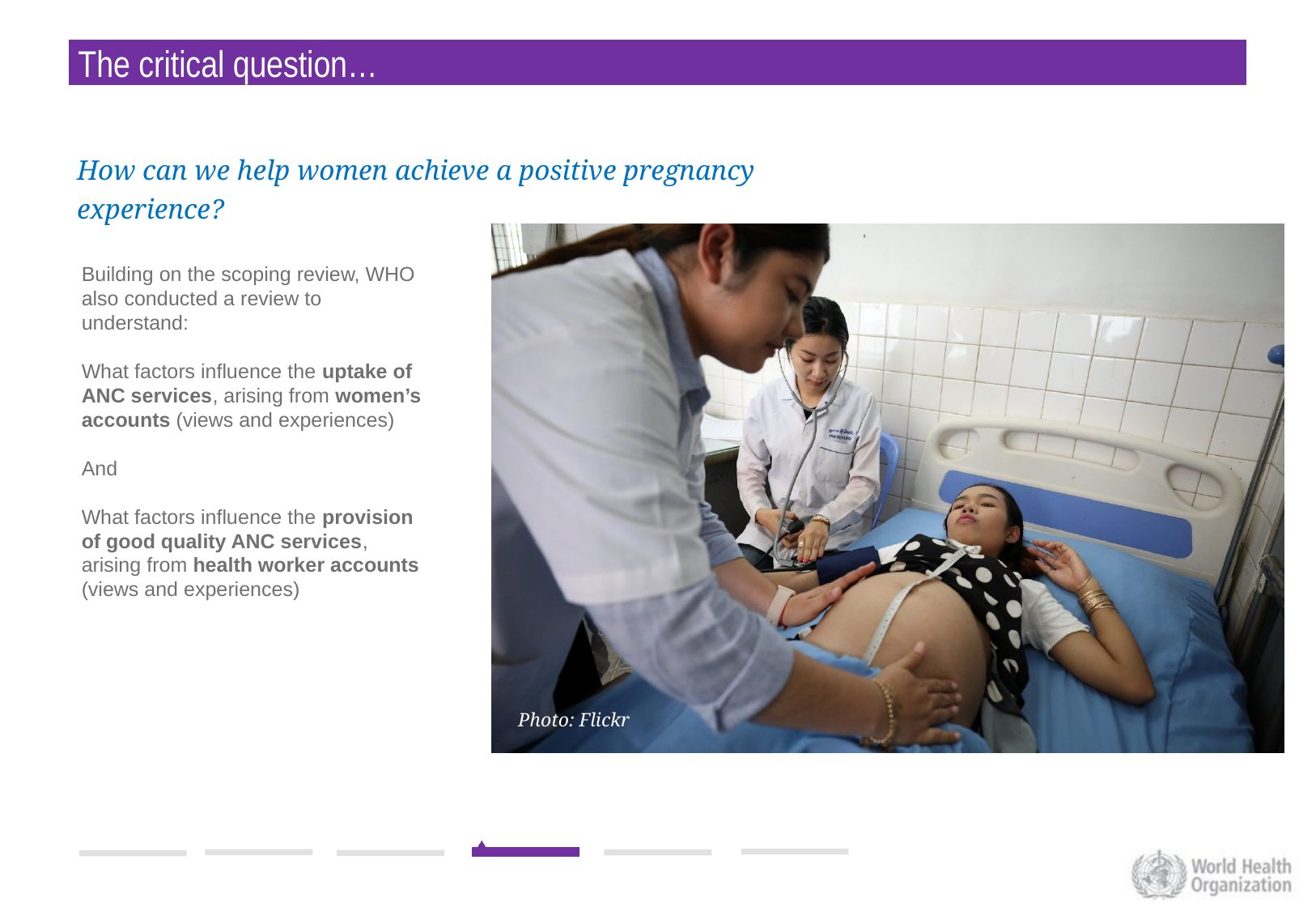

The critical question…
How can we help women achieve a positive pregnancy experience?
Building on the scoping review, WHO also conducted a review to understand:
What factors influence the uptake of ANC services, arising from women’s accounts (views and experiences)
And
What factors influence the provision of good quality ANC services, arising from health worker accounts (views and experiences)
Photo: Flickr

## Slide 27
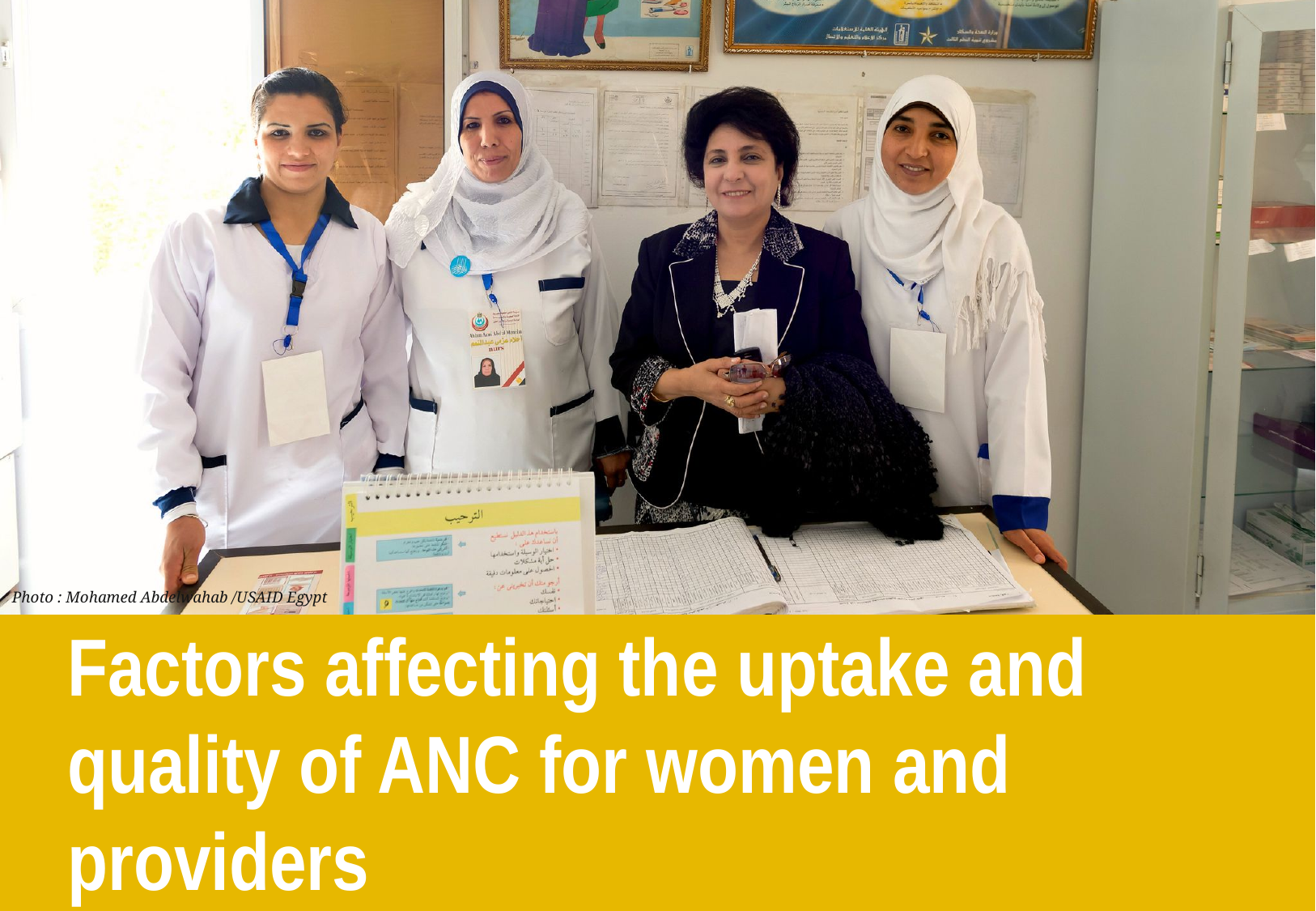

Photo : Mohamed Abdelwahab /USAID Egypt
Factors affecting the uptake and quality of ANC for women and providers

## Slide 28
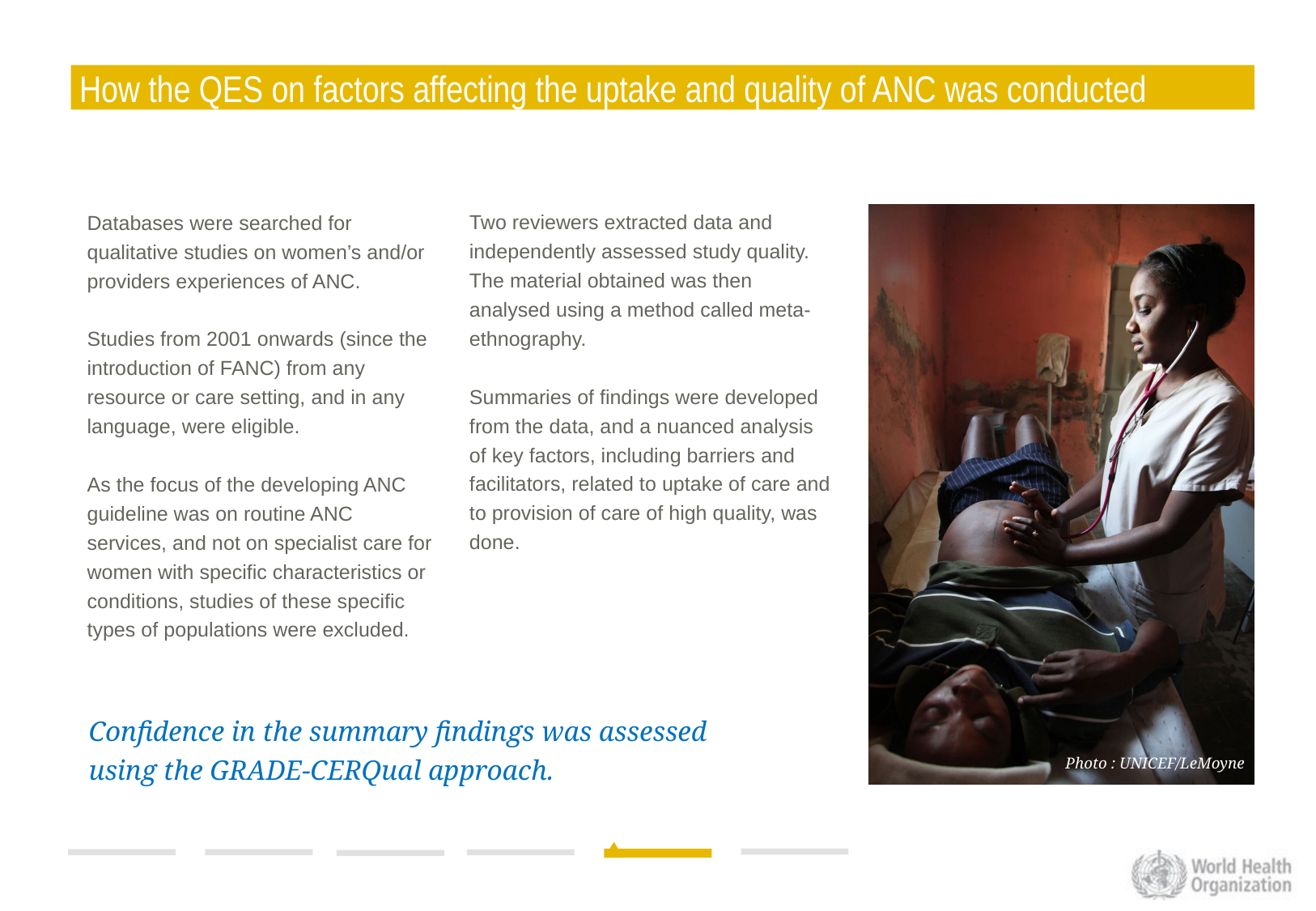

How the QES on factors affecting the uptake and quality of ANC was conducted
Two reviewers extracted data and independently assessed study quality. The material obtained was then analysed using a method called meta-ethnography.
Summaries of findings were developed from the data, and a nuanced analysis of key factors, including barriers and facilitators, related to uptake of care and to provision of care of high quality, was done.
Databases were searched for qualitative studies on women’s and/or providers experiences of ANC.
Studies from 2001 onwards (since the introduction of FANC) from any resource or care setting, and in any language, were eligible.
As the focus of the developing ANC guideline was on routine ANC services, and not on specialist care for women with specific characteristics or conditions, studies of these specific types of populations were excluded.
Confidence in the summary findings was assessed using the GRADE-CERQual approach.
Photo : UNICEF/LeMoyne

## Slide 29
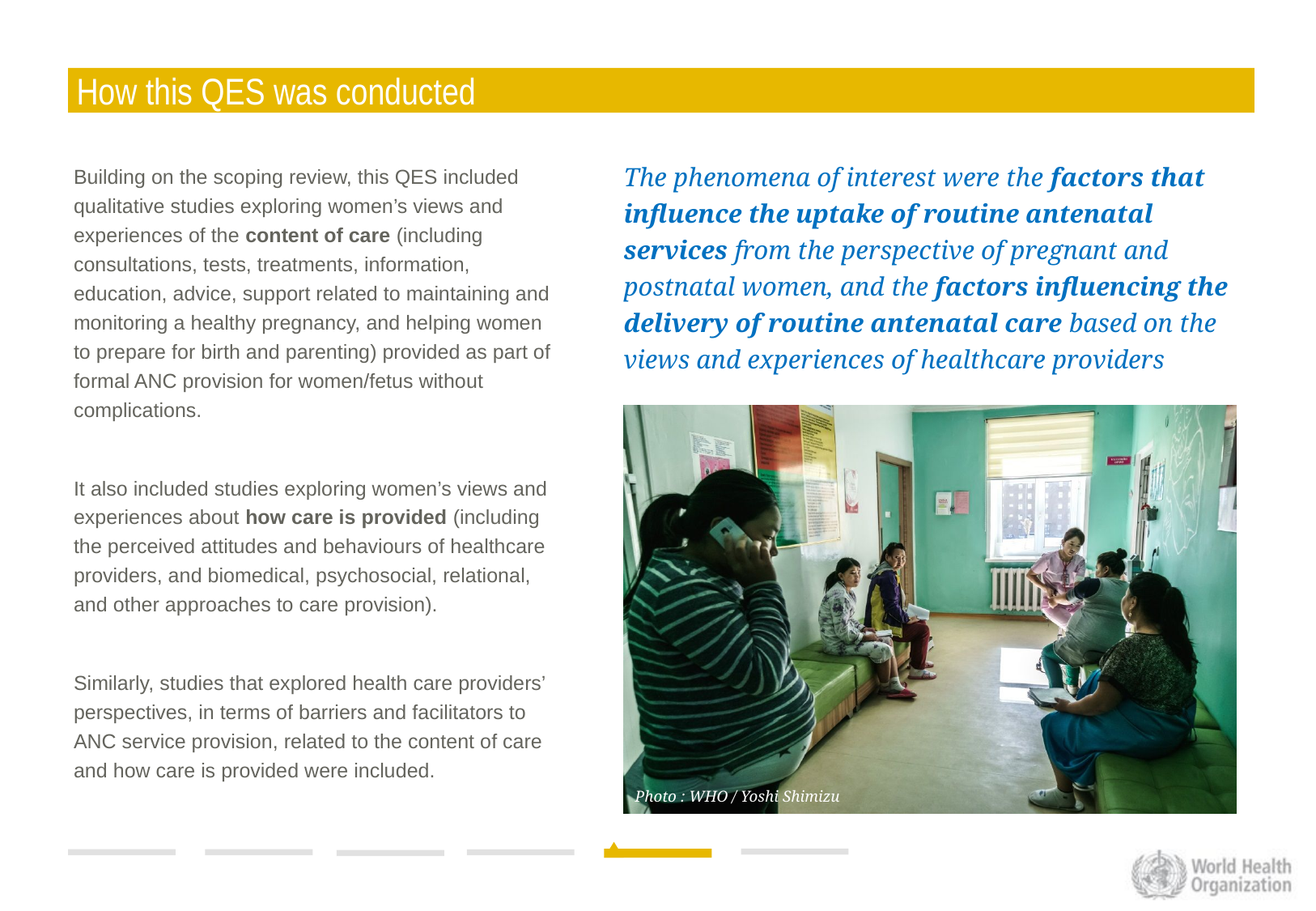

How this QES was conducted
The phenomena of interest were the factors that influence the uptake of routine antenatal services from the perspective of pregnant and postnatal women, and the factors influencing the delivery of routine antenatal care based on the views and experiences of healthcare providers
Building on the scoping review, this QES included qualitative studies exploring women’s views and experiences of the content of care (including consultations, tests, treatments, information, education, advice, support related to maintaining and monitoring a healthy pregnancy, and helping women to prepare for birth and parenting) provided as part of formal ANC provision for women/fetus without complications.
It also included studies exploring women’s views and experiences about how care is provided (including the perceived attitudes and behaviours of healthcare providers, and biomedical, psychosocial, relational, and other approaches to care provision).
Similarly, studies that explored health care providers’ perspectives, in terms of barriers and facilitators to ANC service provision, related to the content of care and how care is provided were included.
Photo : WHO / Yoshi Shimizu
Photo UNICEF/Asselin

## Slide 30
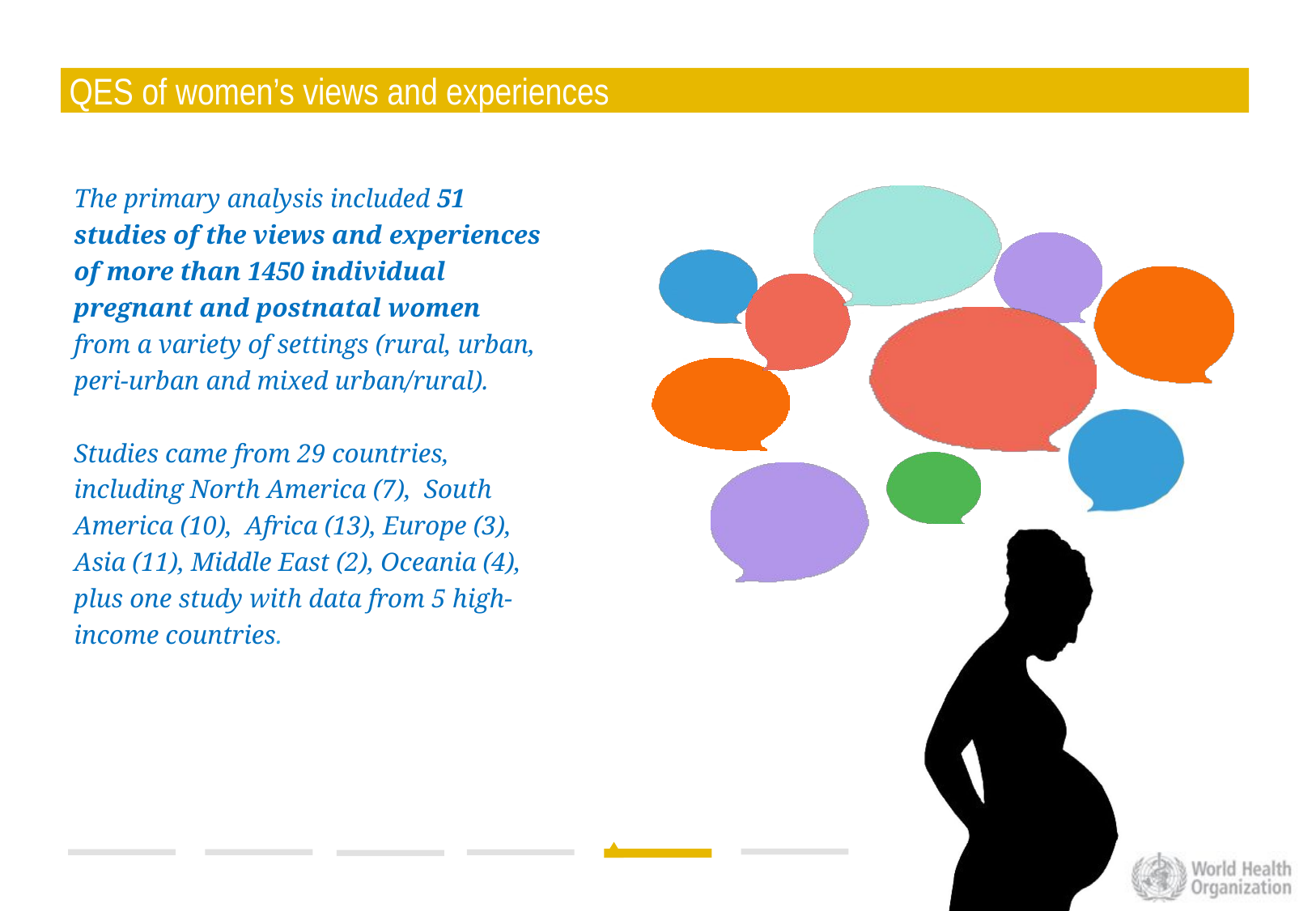

QES of women’s views and experiences
The primary analysis included 51 studies of the views and experiences of more than 1450 individual pregnant and postnatal women from a variety of settings (rural, urban, peri-urban and mixed urban/rural).
Studies came from 29 countries, including North America (7), South America (10), Africa (13), Europe (3), Asia (11), Middle East (2), Oceania (4), plus one study with data from 5 high-income countries.

## Slide 31
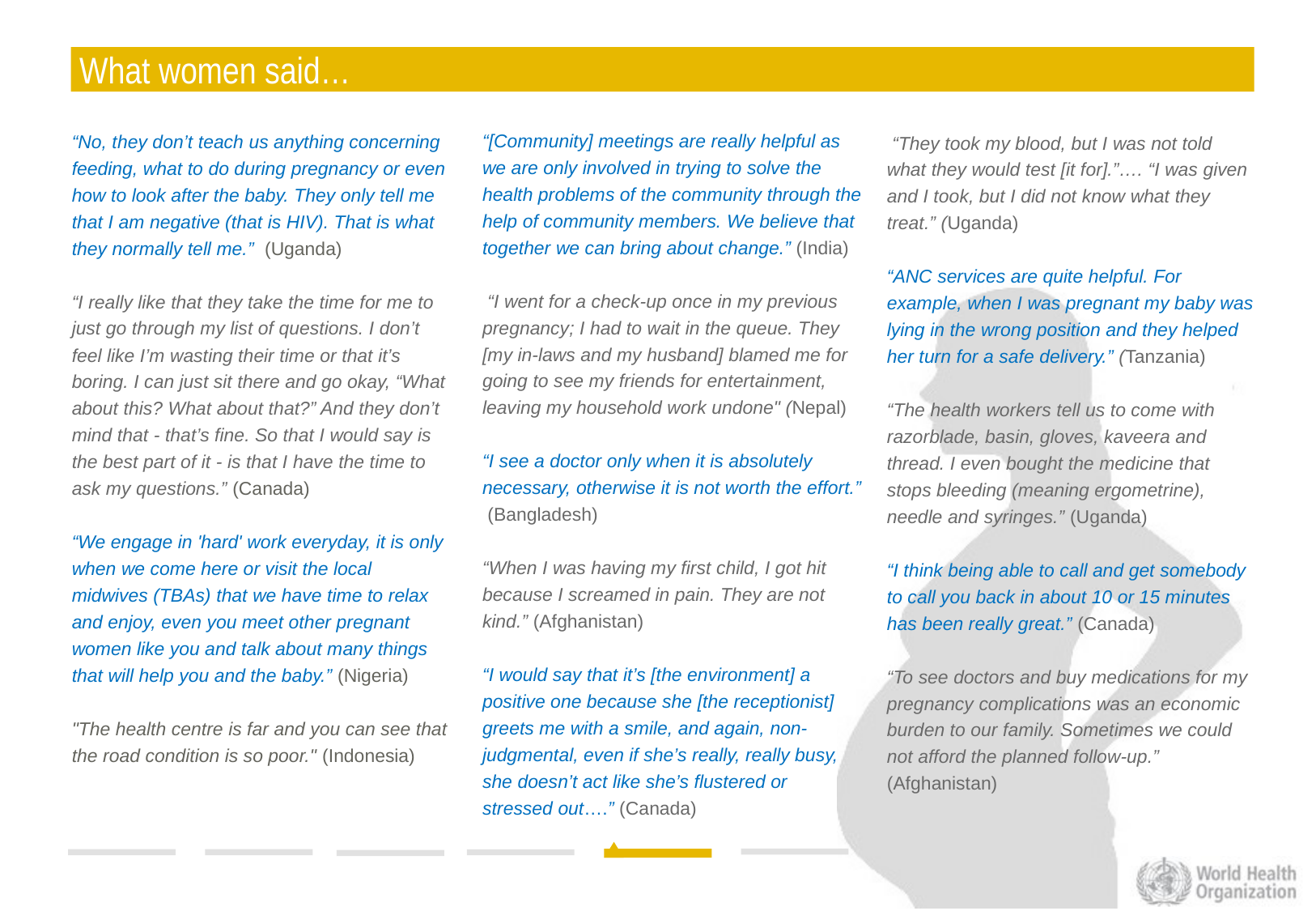

What women said…
“No, they don’t teach us anything concerning feeding, what to do during pregnancy or even how to look after the baby. They only tell me that I am negative (that is HIV). That is what they normally tell me.” (Uganda)
“I really like that they take the time for me to just go through my list of questions. I don’t feel like I’m wasting their time or that it’s boring. I can just sit there and go okay, “What about this? What about that?” And they don’t mind that - that’s fine. So that I would say is the best part of it - is that I have the time to ask my questions.” (Canada)
“We engage in 'hard' work everyday, it is only when we come here or visit the local midwives (TBAs) that we have time to relax and enjoy, even you meet other pregnant women like you and talk about many things that will help you and the baby.” (Nigeria)
"The health centre is far and you can see that the road condition is so poor." (Indonesia)
“[Community] meetings are really helpful as we are only involved in trying to solve the health problems of the community through the help of community members. We believe that together we can bring about change.” (India)
 “I went for a check-up once in my previous pregnancy; I had to wait in the queue. They [my in-laws and my husband] blamed me for going to see my friends for entertainment, leaving my household work undone" (Nepal)
“I see a doctor only when it is absolutely necessary, otherwise it is not worth the effort.” (Bangladesh)
“When I was having my first child, I got hit because I screamed in pain. They are not kind.” (Afghanistan)
“I would say that it’s [the environment] a positive one because she [the receptionist] greets me with a smile, and again, non-judgmental, even if she’s really, really busy, she doesn’t act like she’s flustered or stressed out….” (Canada)
 “They took my blood, but I was not told what they would test [it for].”…. “I was given and I took, but I did not know what they treat.” (Uganda)
“ANC services are quite helpful. For example, when I was pregnant my baby was lying in the wrong position and they helped her turn for a safe delivery.” (Tanzania)
“The health workers tell us to come with razorblade, basin, gloves, kaveera and thread. I even bought the medicine that stops bleeding (meaning ergometrine), needle and syringes.” (Uganda)
“I think being able to call and get somebody to call you back in about 10 or 15 minutes has been really great.” (Canada)
“To see doctors and buy medications for my pregnancy complications was an economic burden to our family. Sometimes we could not afford the planned follow-up.” (Afghanistan)

## Slide 32
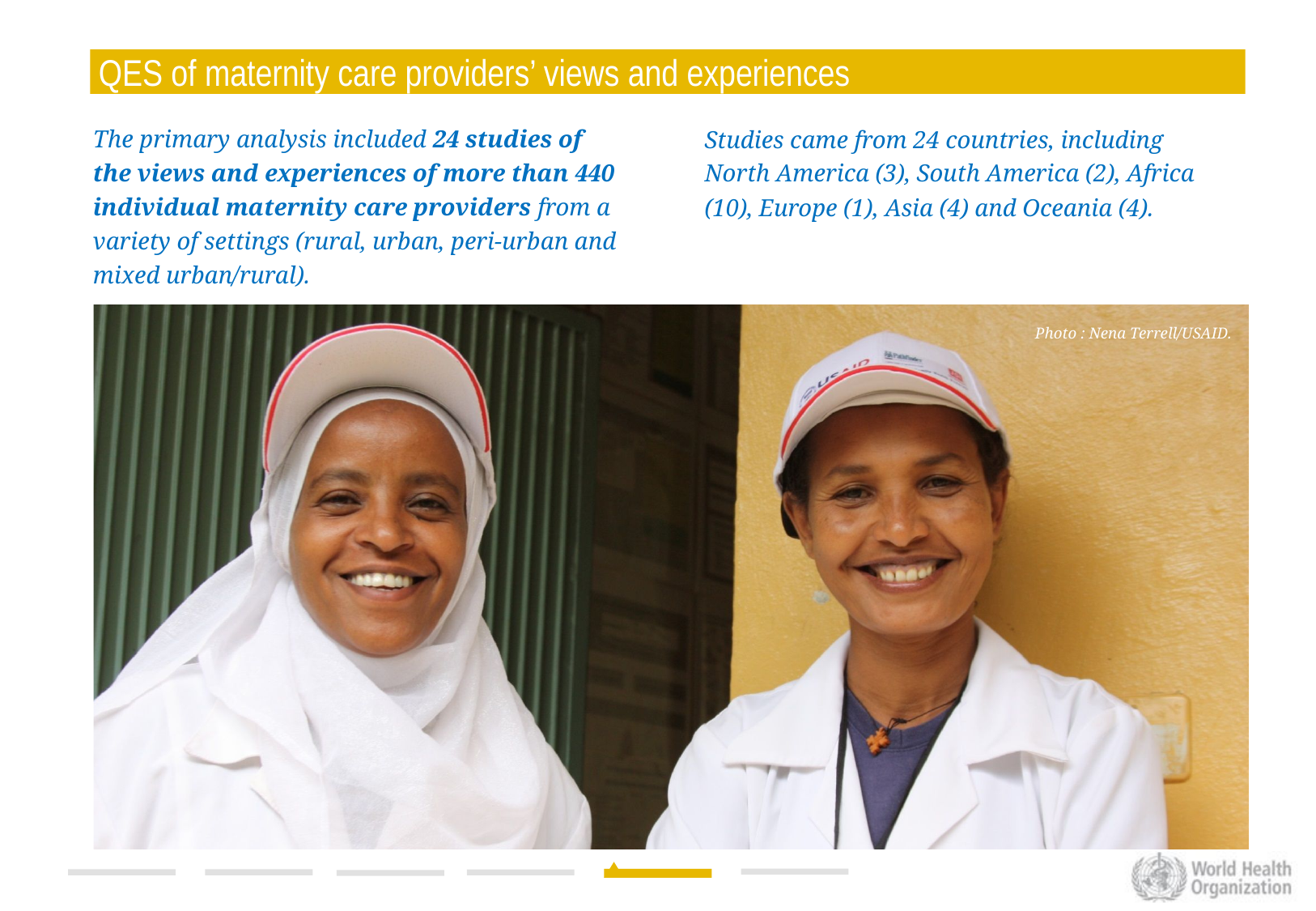

QES of maternity care providers’ views and experiences
The primary analysis included 24 studies of the views and experiences of more than 440 individual maternity care providers from a variety of settings (rural, urban, peri-urban and mixed urban/rural).
Studies came from 24 countries, including North America (3), South America (2), Africa (10), Europe (1), Asia (4) and Oceania (4).
Photo : Nena Terrell/USAID.

## Slide 33
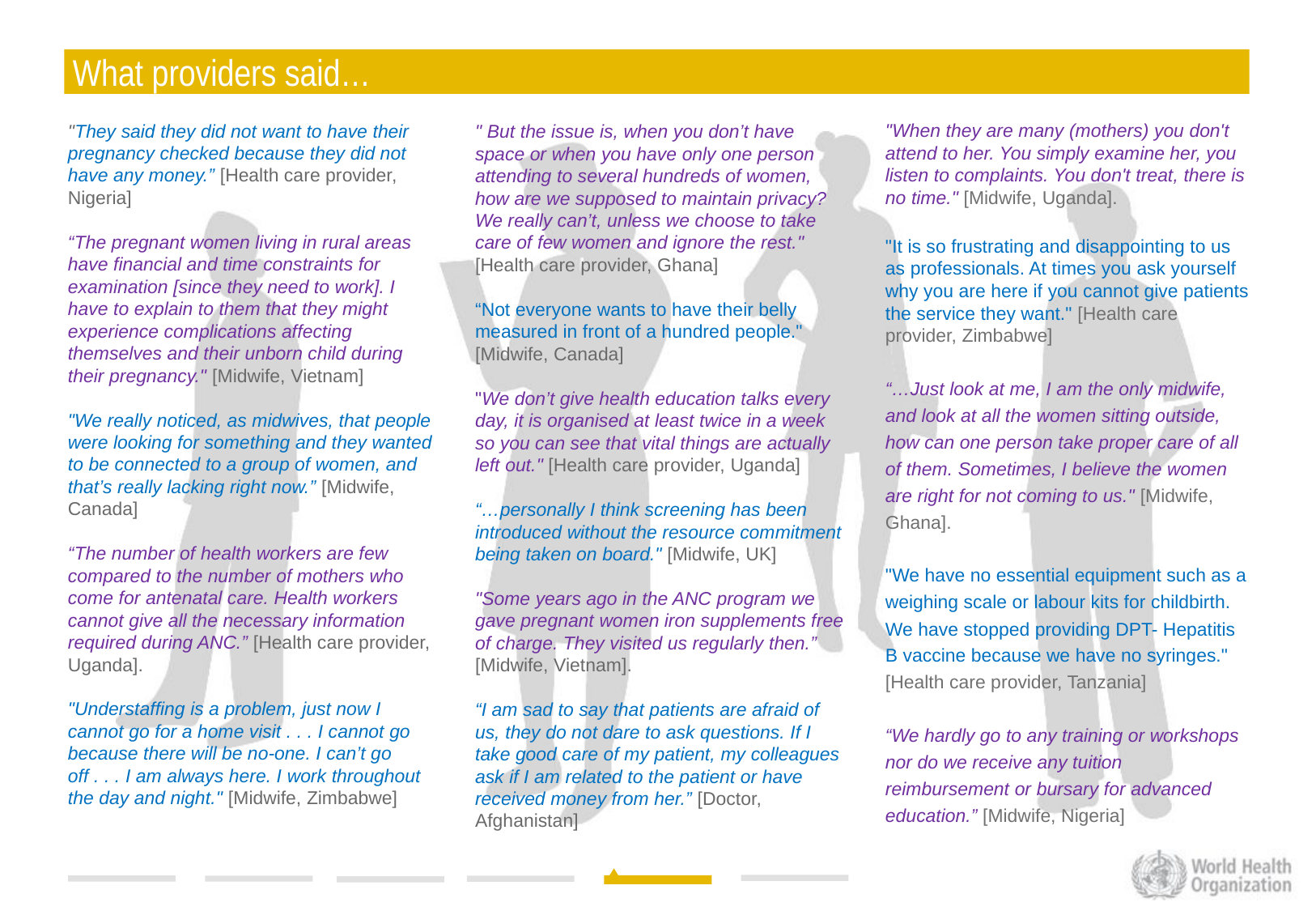

What providers said…
"When they are many (mothers) you don't attend to her. You simply examine her, you listen to complaints. You don't treat, there is no time." [Midwife, Uganda].
"It is so frustrating and disappointing to us as professionals. At times you ask yourself why you are here if you cannot give patients the service they want." [Health care provider, Zimbabwe]
“…Just look at me, I am the only midwife, and look at all the women sitting outside, how can one person take proper care of all of them. Sometimes, I believe the women are right for not coming to us." [Midwife, Ghana].
"We have no essential equipment such as a weighing scale or labour kits for childbirth. We have stopped providing DPT- Hepatitis B vaccine because we have no syringes." [Health care provider, Tanzania]
“We hardly go to any training or workshops nor do we receive any tuition reimbursement or bursary for advanced education.” [Midwife, Nigeria]
"They said they did not want to have their pregnancy checked because they did not have any money.” [Health care provider, Nigeria]
“The pregnant women living in rural areas have financial and time constraints for examination [since they need to work]. I have to explain to them that they might experience complications affecting themselves and their unborn child during their pregnancy." [Midwife, Vietnam]
"We really noticed, as midwives, that people were looking for something and they wanted to be connected to a group of women, and that’s really lacking right now.” [Midwife, Canada]
“The number of health workers are few compared to the number of mothers who come for antenatal care. Health workers cannot give all the necessary information required during ANC.” [Health care provider, Uganda].
"Understaffing is a problem, just now I cannot go for a home visit . . . I cannot go because there will be no-one. I can’t go off . . . I am always here. I work throughout the day and night." [Midwife, Zimbabwe]
" But the issue is, when you don’t have space or when you have only one person attending to several hundreds of women, how are we supposed to maintain privacy? We really can’t, unless we choose to take care of few women and ignore the rest." [Health care provider, Ghana]
“Not everyone wants to have their belly measured in front of a hundred people." [Midwife, Canada]
"We don’t give health education talks every day, it is organised at least twice in a week so you can see that vital things are actually left out." [Health care provider, Uganda]
“…personally I think screening has been introduced without the resource commitment being taken on board." [Midwife, UK]
"Some years ago in the ANC program we gave pregnant women iron supplements free of charge. They visited us regularly then.” [Midwife, Vietnam].
“I am sad to say that patients are afraid of us, they do not dare to ask questions. If I take good care of my patient, my colleagues ask if I am related to the patient or have received money from her.” [Doctor, Afghanistan]

## Slide 34
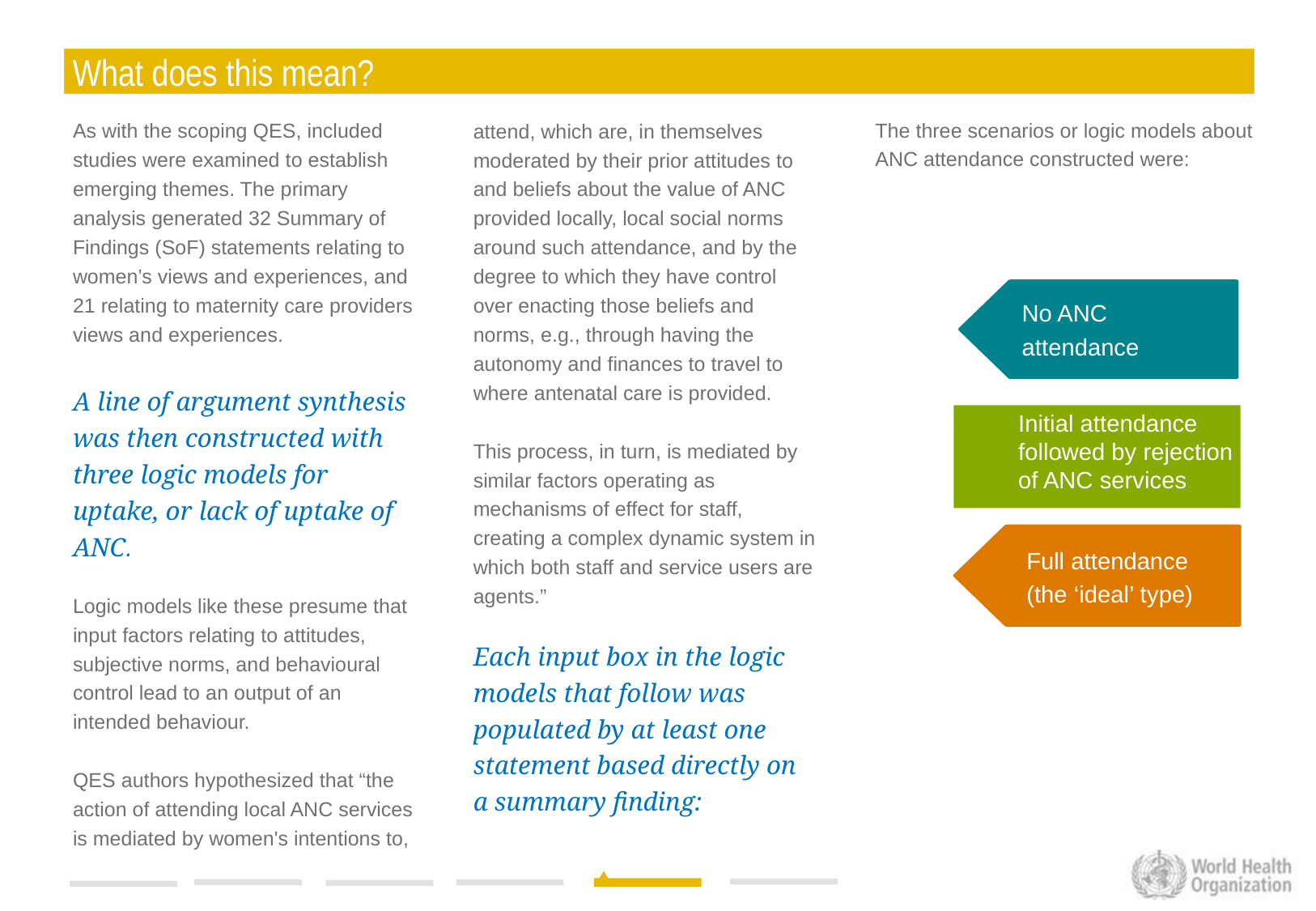

What does this mean?
As with the scoping QES, included studies were examined to establish emerging themes. The primary analysis generated 32 Summary of Findings (SoF) statements relating to women’s views and experiences, and 21 relating to maternity care providers views and experiences.
A line of argument synthesis was then constructed with three logic models for uptake, or lack of uptake of ANC.
Logic models like these presume that input factors relating to attitudes, subjective norms, and behavioural control lead to an output of an intended behaviour.
QES authors hypothesized that “the action of attending local ANC services is mediated by women's intentions to,
attend, which are, in themselves moderated by their prior attitudes to and beliefs about the value of ANC provided locally, local social norms around such attendance, and by the degree to which they have control over enacting those beliefs and norms, e.g., through having the autonomy and finances to travel to where antenatal care is provided.
This process, in turn, is mediated by similar factors operating as mechanisms of effect for staff, creating a complex dynamic system in which both staff and service users are agents.”
Each input box in the logic models that follow was populated by at least one statement based directly on a summary finding:
The three scenarios or logic models about ANC attendance constructed were:
1
Logic model
No ANC attendance
.
2
Logic model
Initial attendance
followed by rejection of ANC services
3
Full attendance
(the ‘ideal’ type)

## Slide 35
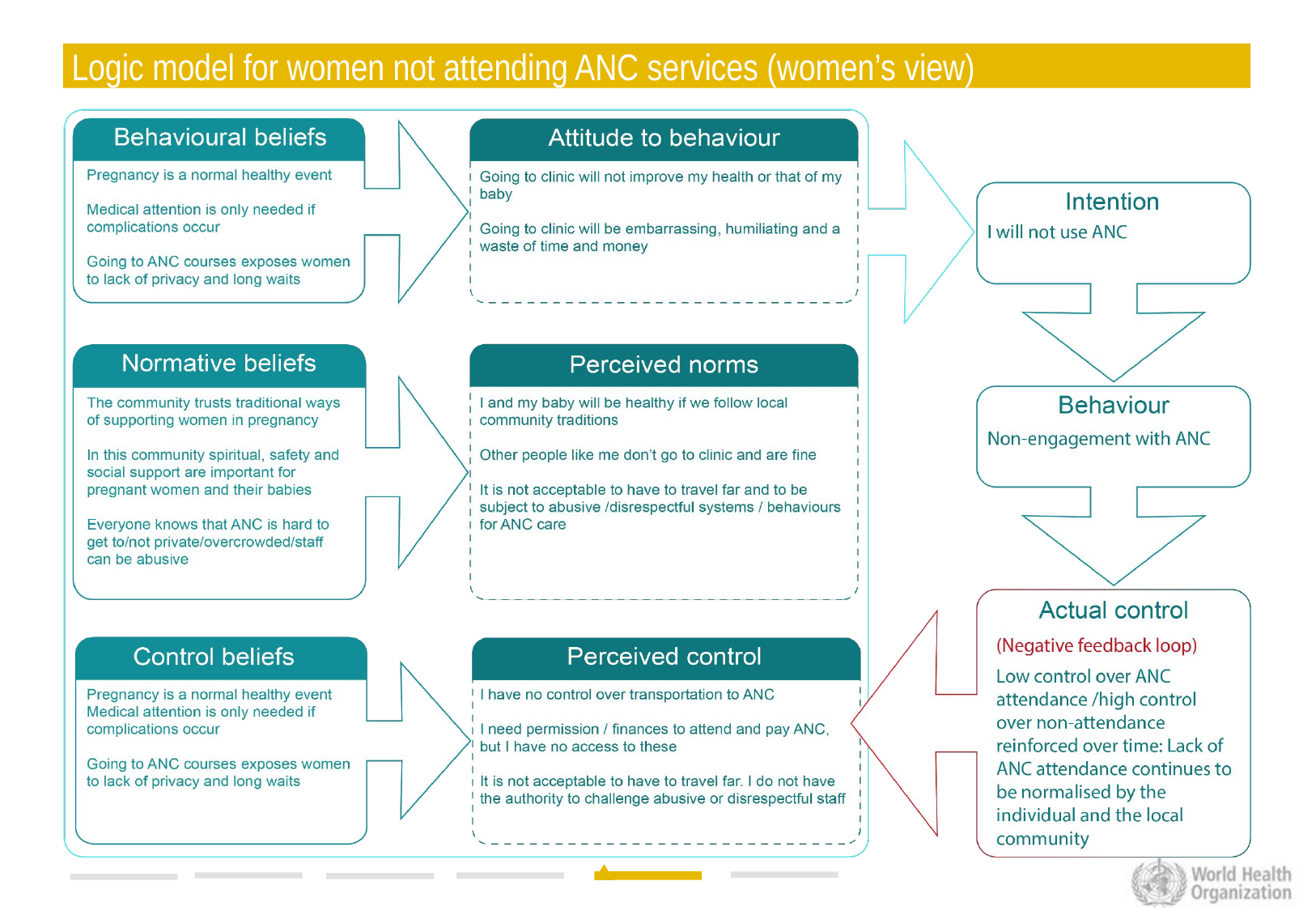

Logic model for women not attending ANC services (women’s view)

## Slide 36
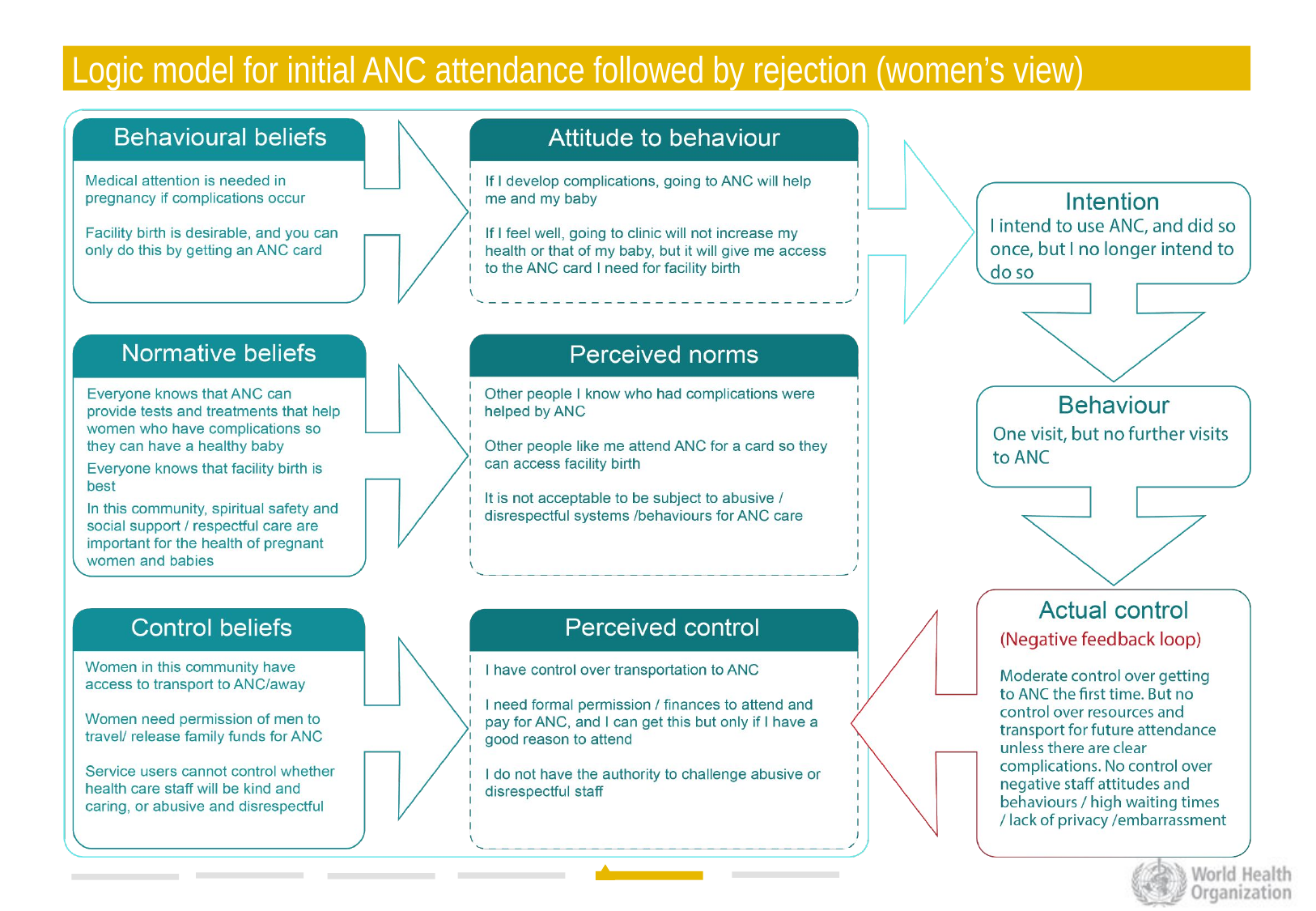

Logic model for initial ANC attendance followed by rejection (women’s view)

## Slide 37
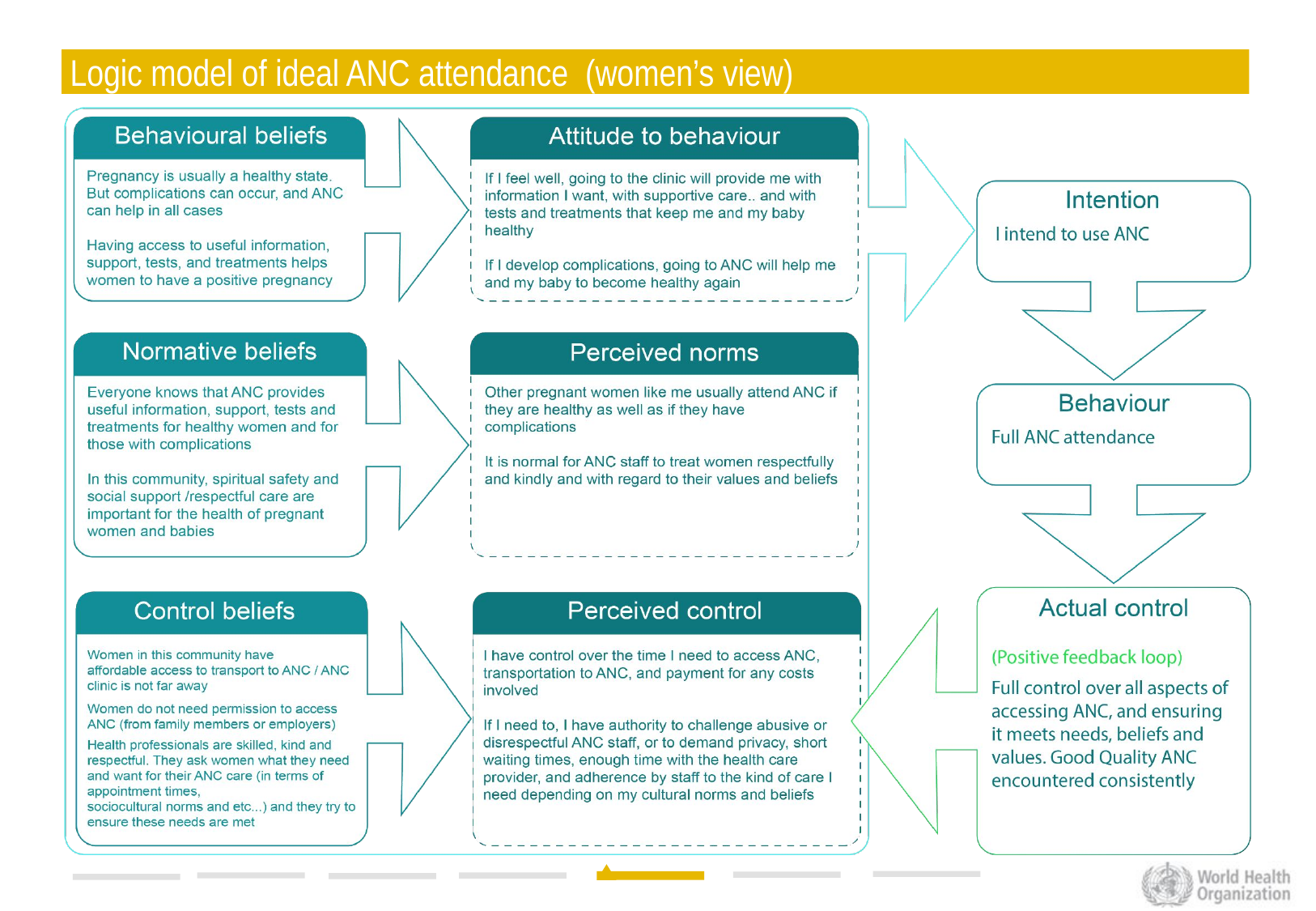

Logic model of ideal ANC attendance (women’s view)

## Slide 38
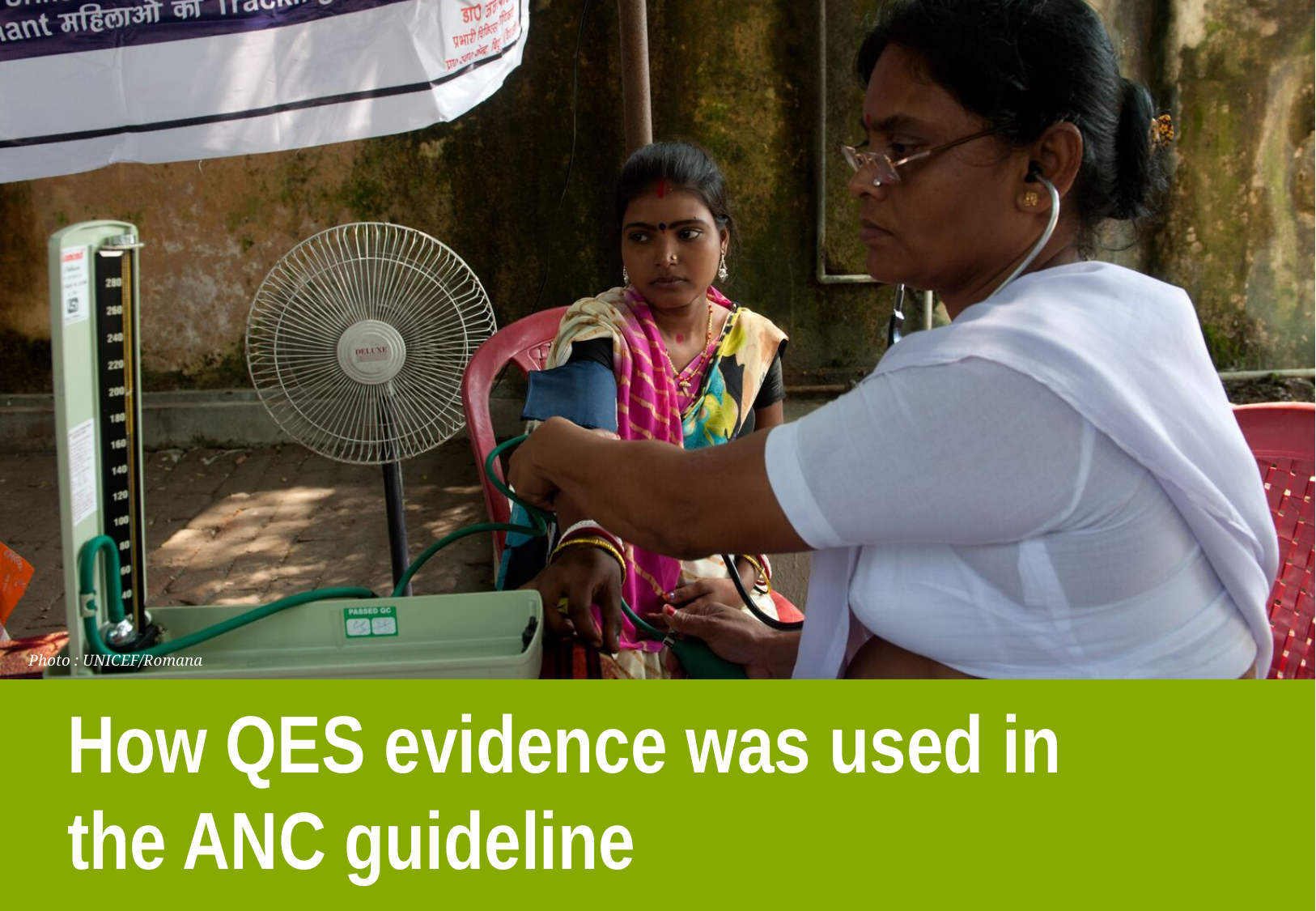

Experiences of Preventative Measures Examples
Photo : UNICEF/Romana
How QES evidence was used in the ANC guideline

## Slide 39
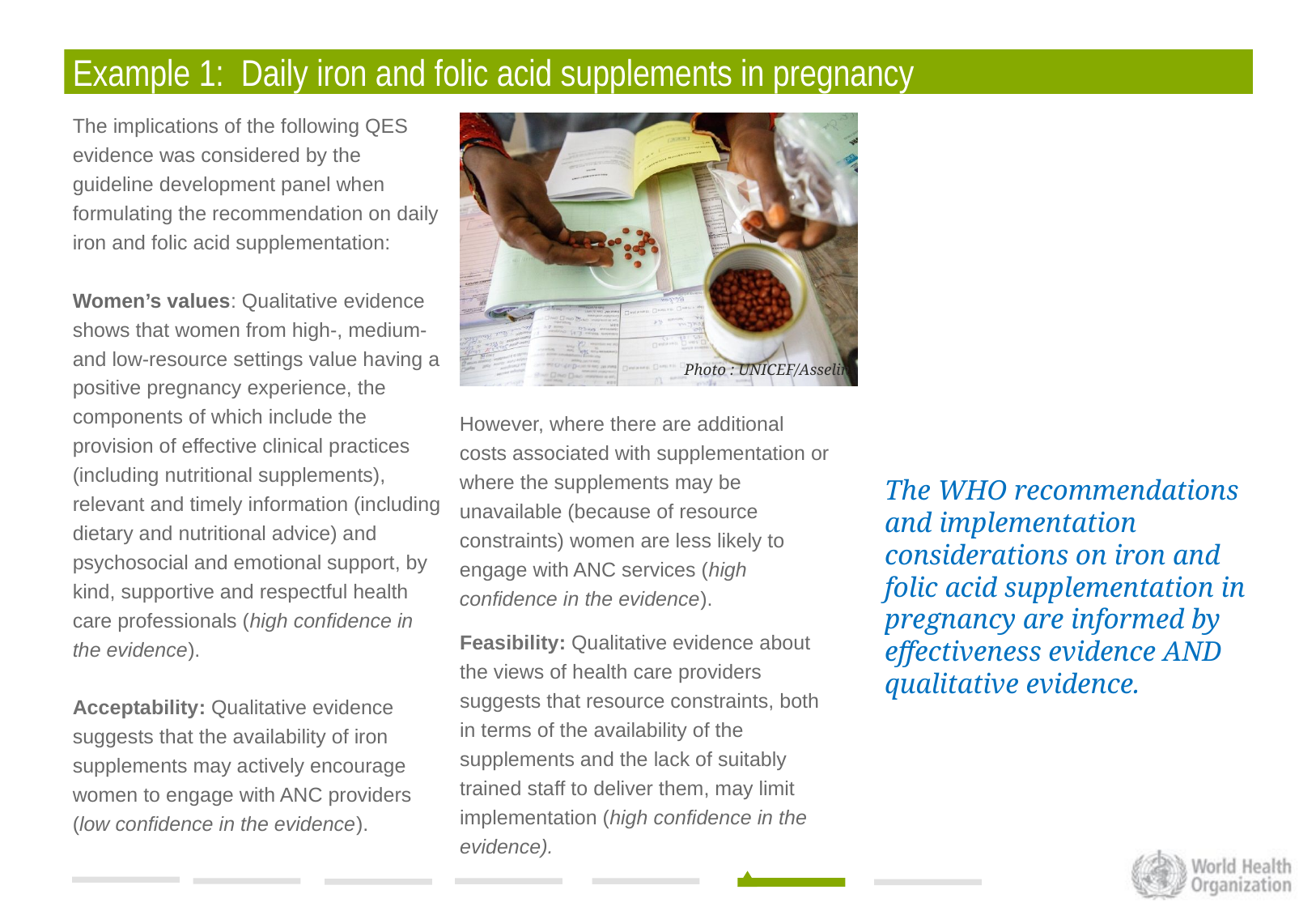

Example 1: Daily iron and folic acid supplements in pregnancy
The implications of the following QES evidence was considered by the guideline development panel when formulating the recommendation on daily iron and folic acid supplementation:
Women’s values: Qualitative evidence shows that women from high-, medium- and low-resource settings value having a positive pregnancy experience, the components of which include the provision of effective clinical practices
(including nutritional supplements), relevant and timely information (including dietary and nutritional advice) and psychosocial and emotional support, by kind, supportive and respectful health care professionals (high confidence in the evidence).
Acceptability: Qualitative evidence suggests that the availability of iron supplements may actively encourage women to engage with ANC providers (low confidence in the evidence).
Photo : UNICEF/Asselin
However, where there are additional costs associated with supplementation or where the supplements may be unavailable (because of resource constraints) women are less likely to engage with ANC services (high confidence in the evidence).
The WHO recommendations and implementation considerations on iron and folic acid supplementation in pregnancy are informed by effectiveness evidence AND qualitative evidence.
Feasibility: Qualitative evidence about the views of health care providers suggests that resource constraints, both in terms of the availability of the supplements and the lack of suitably trained staff to deliver them, may limit implementation (high confidence in the evidence).

## Slide 40
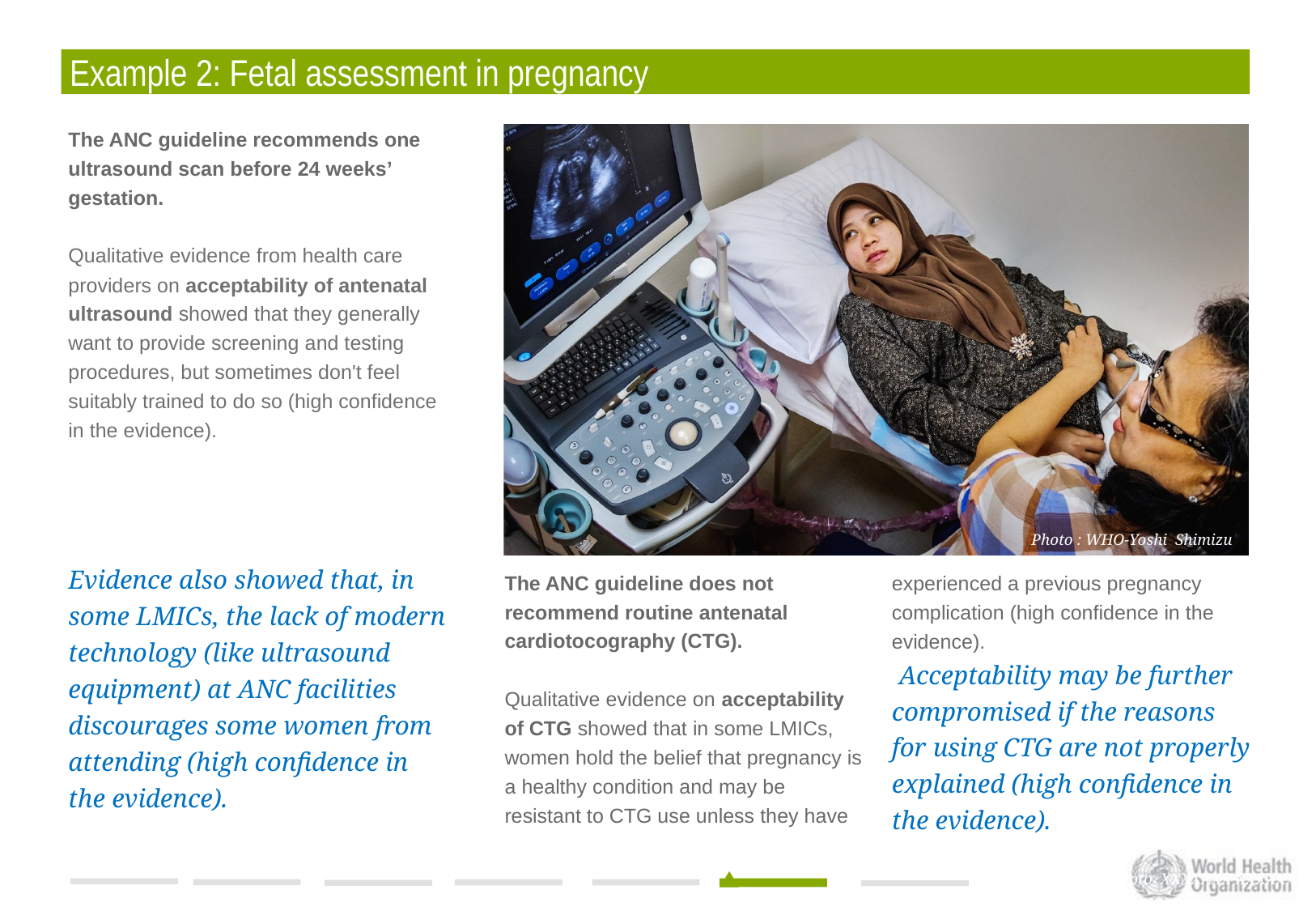

Example 2: Fetal assessment in pregnancy
The ANC guideline recommends one ultrasound scan before 24 weeks’ gestation.
Qualitative evidence from health care providers on acceptability of antenatal ultrasound showed that they generally want to provide screening and testing procedures, but sometimes don't feel suitably trained to do so (high confidence in the evidence).
Evidence also showed that, in some LMICs, the lack of modern technology (like ultrasound equipment) at ANC facilities discourages some women from attending (high confidence in the evidence).
Photo : WHO-Yoshi Shimizu
The ANC guideline does not recommend routine antenatal cardiotocography (CTG).
Qualitative evidence on acceptability of CTG showed that in some LMICs, women hold the belief that pregnancy is a healthy condition and may be resistant to CTG use unless they have
experienced a previous pregnancy complication (high confidence in the evidence).
 Acceptability may be further compromised if the reasons for using CTG are not properly explained (high confidence in the evidence).
Photo XXXXXXX XXXXXX

## Slide 41
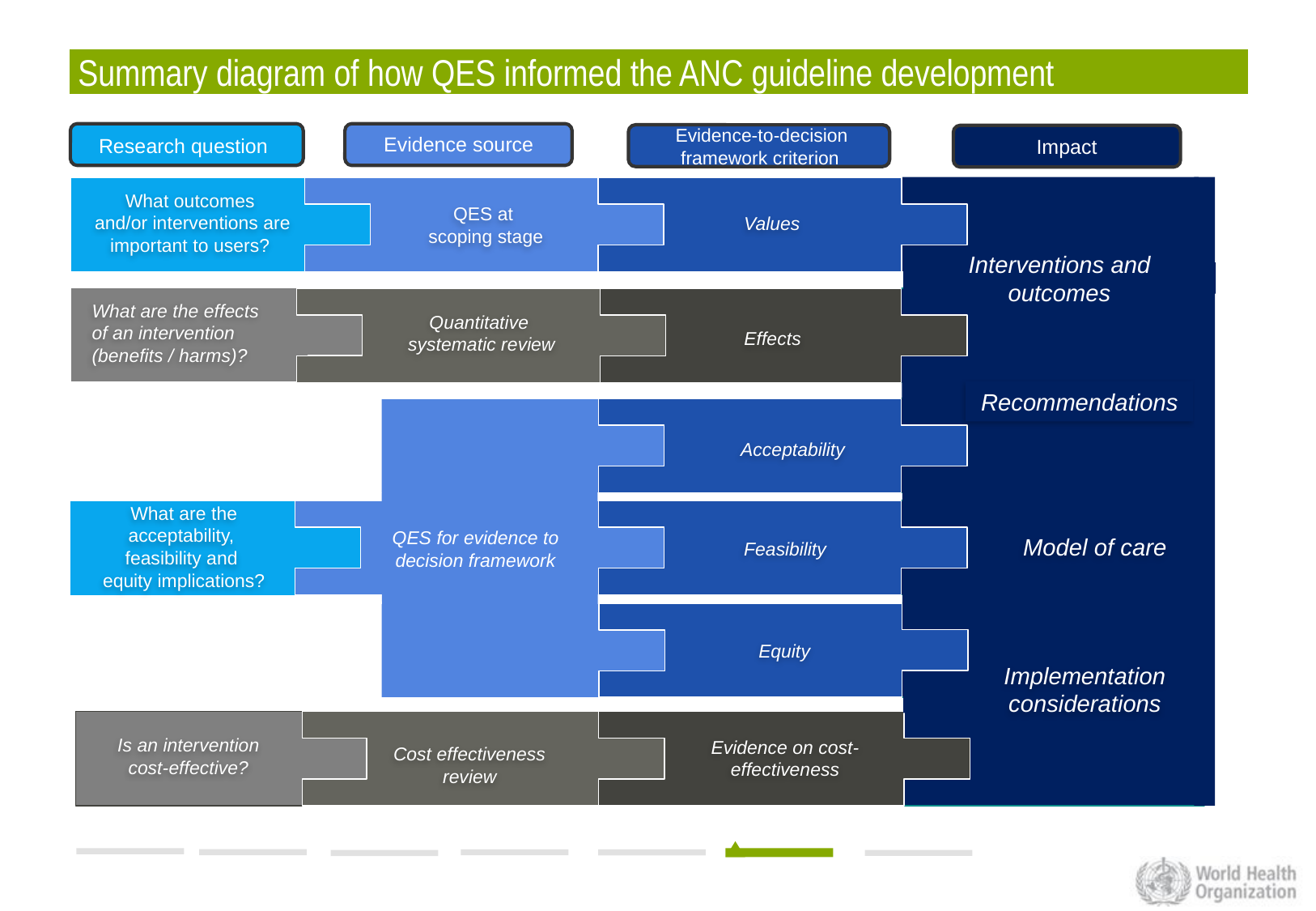

Summary diagram of how QES informed the ANC guideline development
Evidence-to-decision
 framework criterion
Impact
Evidence source
Research question
What outcomes
 and/or interventions are important to users?
QES at
scoping stage
Values
Interventions and outcomes
What are the effects
of an intervention
(benefits / harms)?
Quantitative
systematic review
Effects
Recommendations
Acceptability
What are the acceptability,
feasibility and
equity implications?
QES for evidence to decision framework
Model of care
Feasibility
Equity
Implementation
considerations
Is an intervention
cost-effective?
Evidence on cost- effectiveness
Cost effectiveness
review

## Slide 42
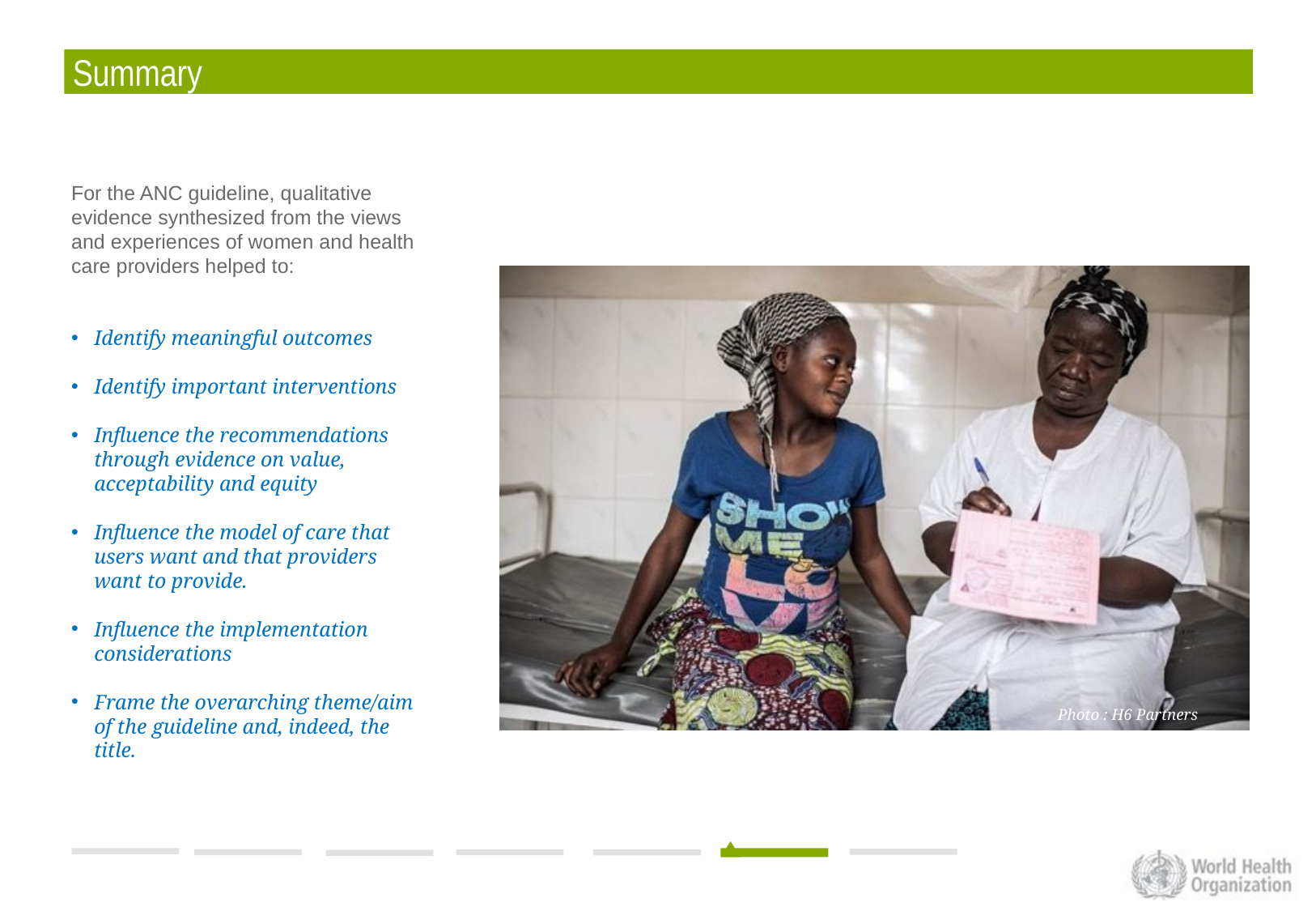

Summary
For the ANC guideline, qualitative evidence synthesized from the views and experiences of women and health care providers helped to:
Identify meaningful outcomes
Identify important interventions
Influence the recommendations through evidence on value, acceptability and equity
Influence the model of care that users want and that providers want to provide.
Influence the implementation considerations
Frame the overarching theme/aim of the guideline and, indeed, the title.
Photo : H6 Partners

## Slide 43
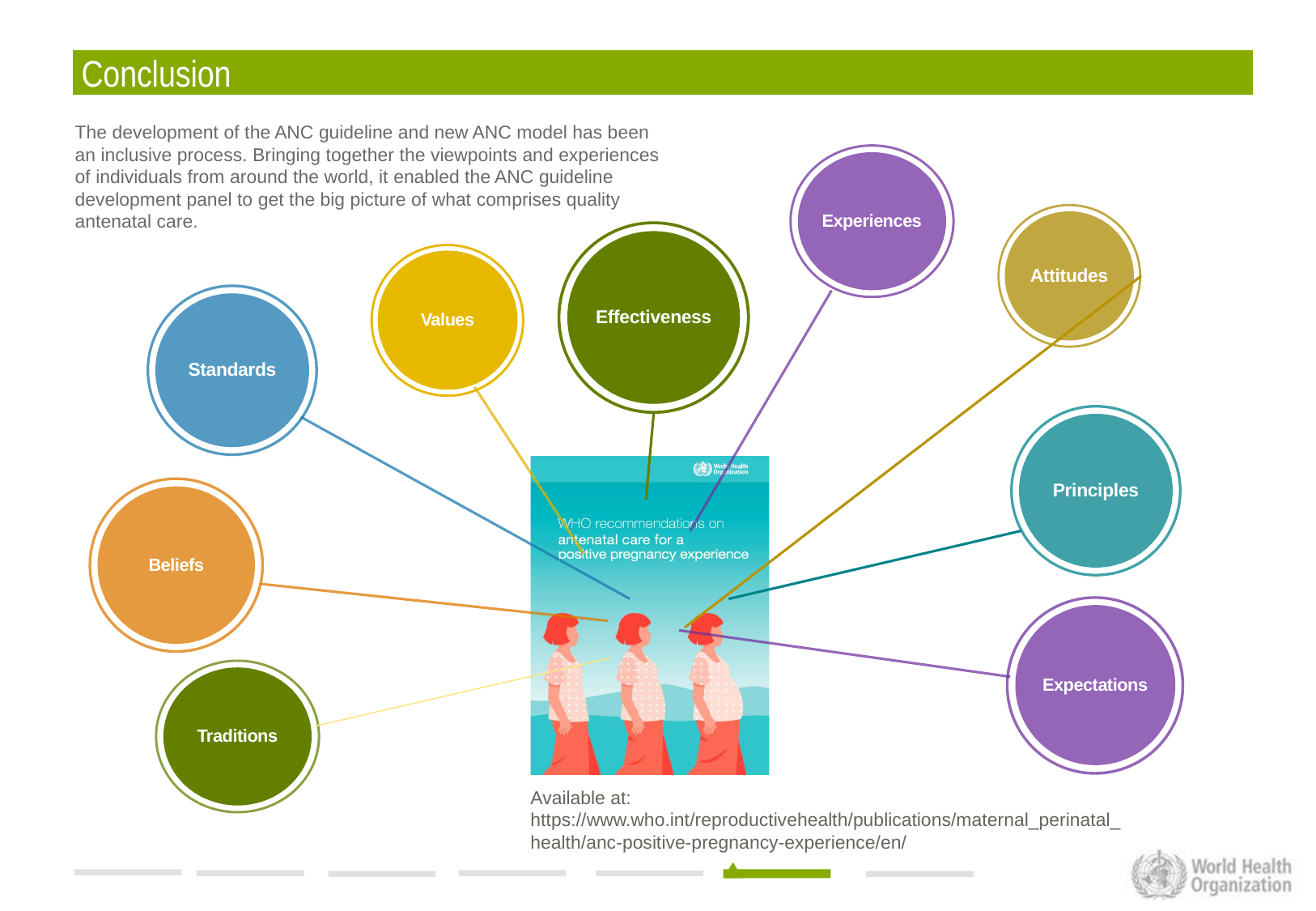

Conclusion
The development of the ANC guideline and new ANC model has been an inclusive process. Bringing together the viewpoints and experiences of individuals from around the world, it enabled the ANC guideline development panel to get the big picture of what comprises quality antenatal care.
Experiences
Attitudes
Effectiveness
Values
Standards
Principles
Beliefs
Expectations
Traditions
Available at: https://www.who.int/reproductivehealth/publications/maternal_perinatal_health/anc-positive-pregnancy-experience/en/

## Slide 44
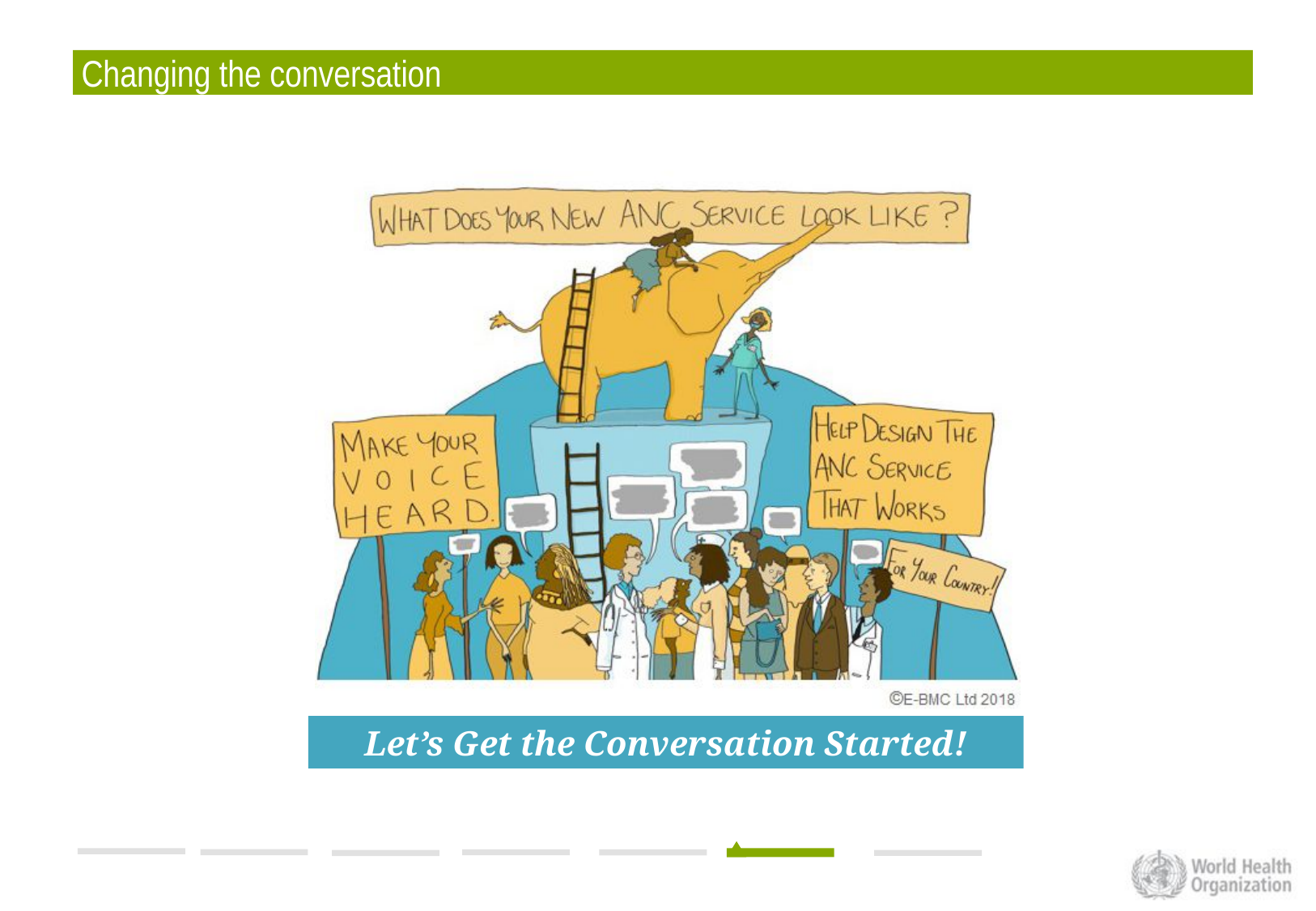

Changing the conversation
Let’s Get the Conversation Started!

## Slide 45
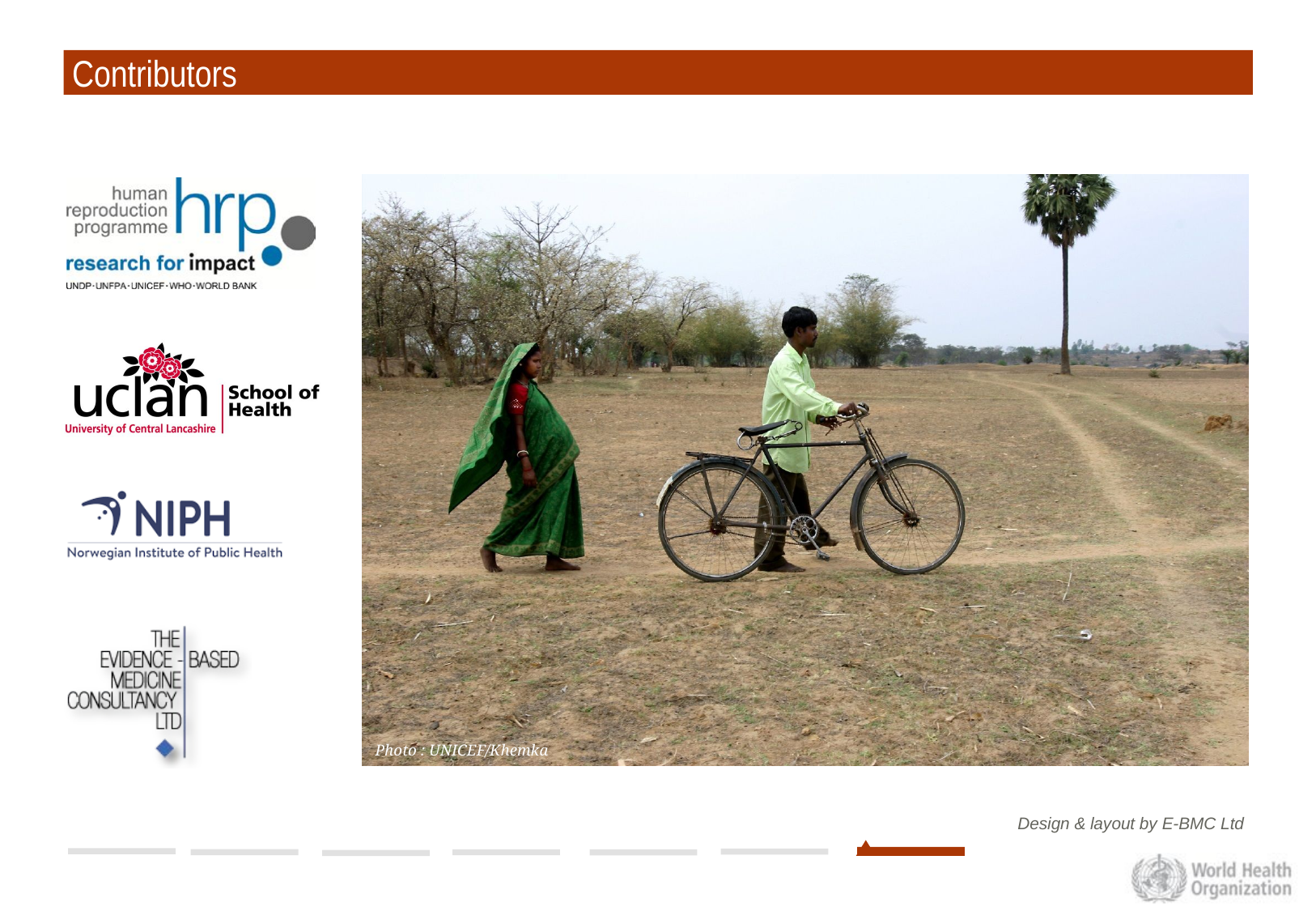

Contributors
#
Photo : UNICEF/Khemka
Design & layout by E-BMC Ltd

## Slide 46
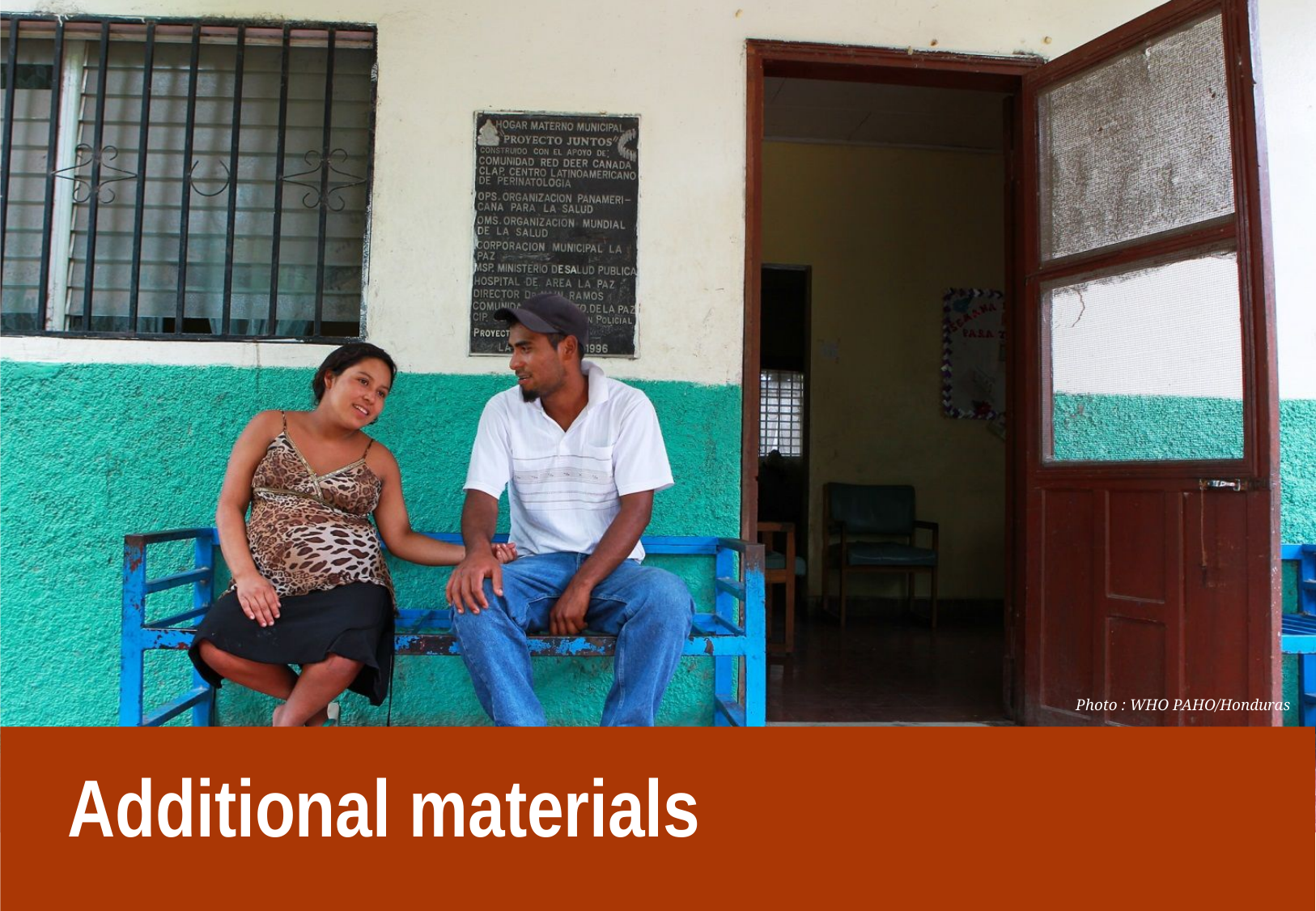

#
Photo : WHO PAHO/Honduras
Photo XXXXXXX XXXXXX
Additional materials

## Slide 47
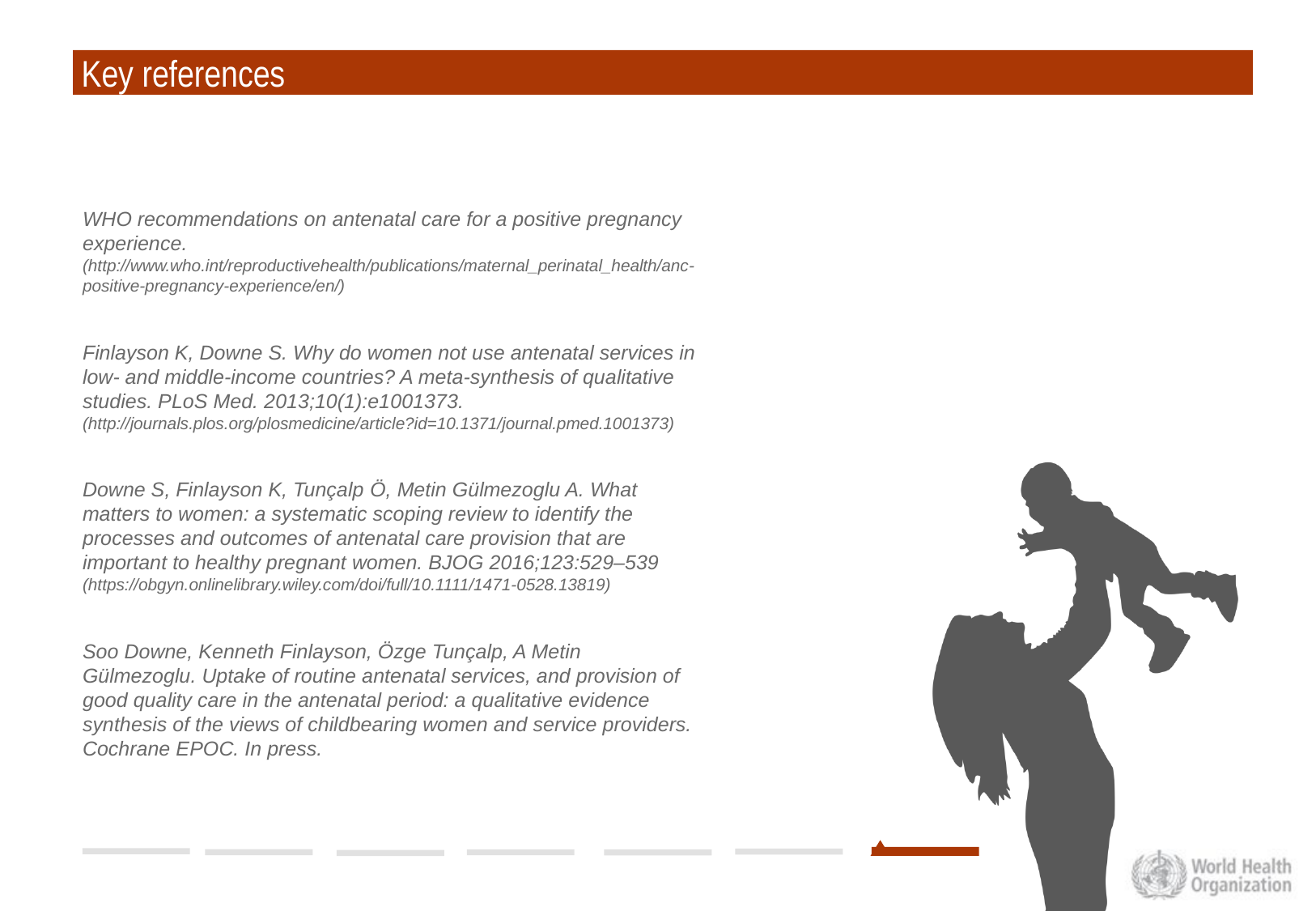

Key references
WHO recommendations on antenatal care for a positive pregnancy experience.
(http://www.who.int/reproductivehealth/publications/maternal_perinatal_health/anc-positive-pregnancy-experience/en/)
Finlayson K, Downe S. Why do women not use antenatal services in low- and middle-income countries? A meta-synthesis of qualitative studies. PLoS Med. 2013;10(1):e1001373.
(http://journals.plos.org/plosmedicine/article?id=10.1371/journal.pmed.1001373)
Downe S, Finlayson K, Tunçalp Ӧ, Metin Gülmezoglu A. What matters to women: a systematic scoping review to identify the processes and outcomes of antenatal care provision that are important to healthy pregnant women. BJOG 2016;123:529–539
(https://obgyn.onlinelibrary.wiley.com/doi/full/10.1111/1471-0528.13819)
Soo Downe, Kenneth Finlayson, Özge Tunçalp, A Metin Gülmezoglu. Uptake of routine antenatal services, and provision of good quality care in the antenatal period: a qualitative evidence synthesis of the views of childbearing women and service providers. Cochrane EPOC. In press.

## Slide 48
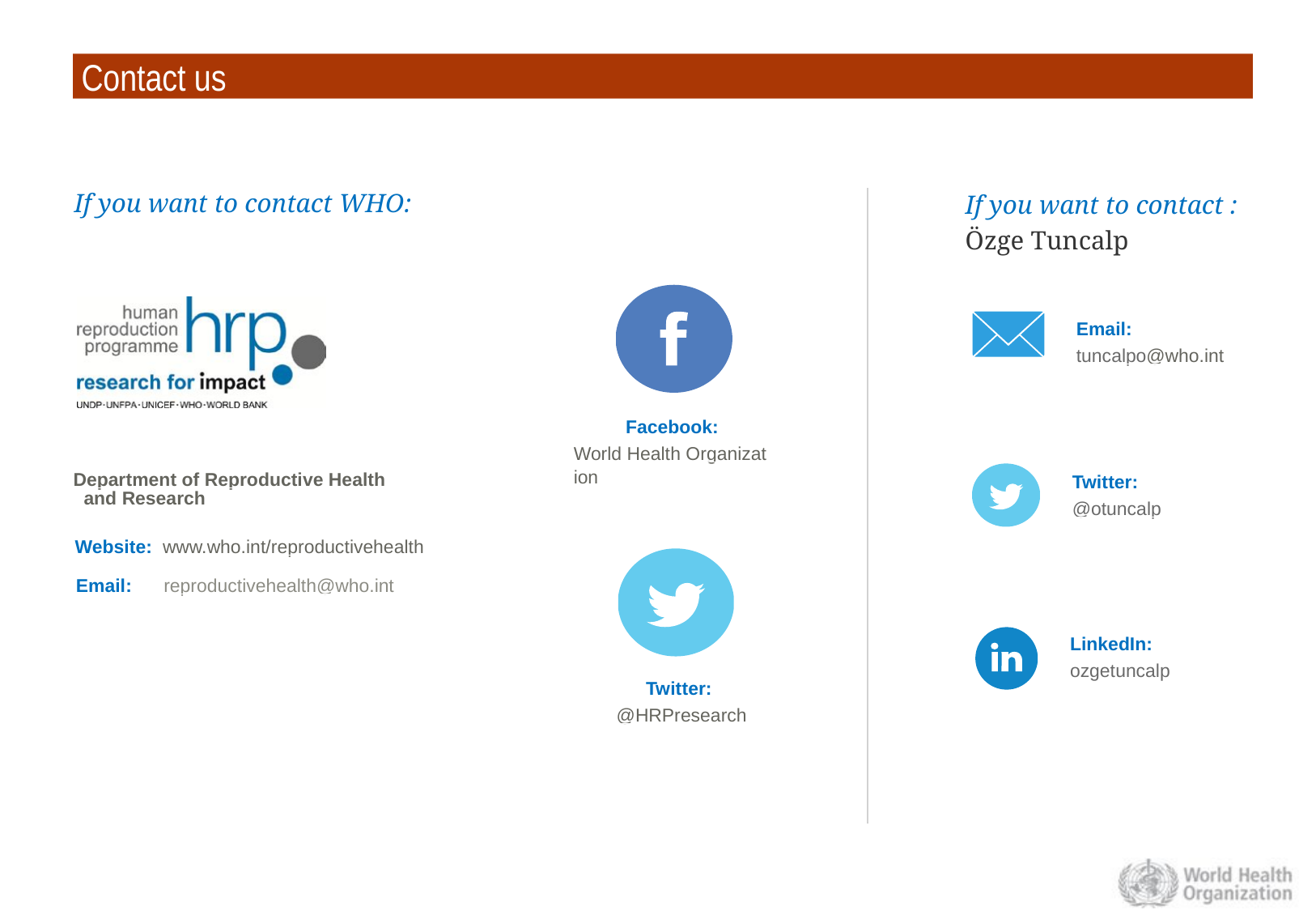

Contact us
If you want to contact WHO:
If you want to contact : Özge Tuncalp
Facebook:
World Health Organization
Email:
tuncalpo@who.int
Twitter: @otuncalp
Department of Reproductive Health and Research
Website:  www.who.int/reproductivehealth
Email: reproductivehealth@who.int
Twitter:
 @HRPresearch
LinkedIn:
ozgetuncalp

## Slide 49
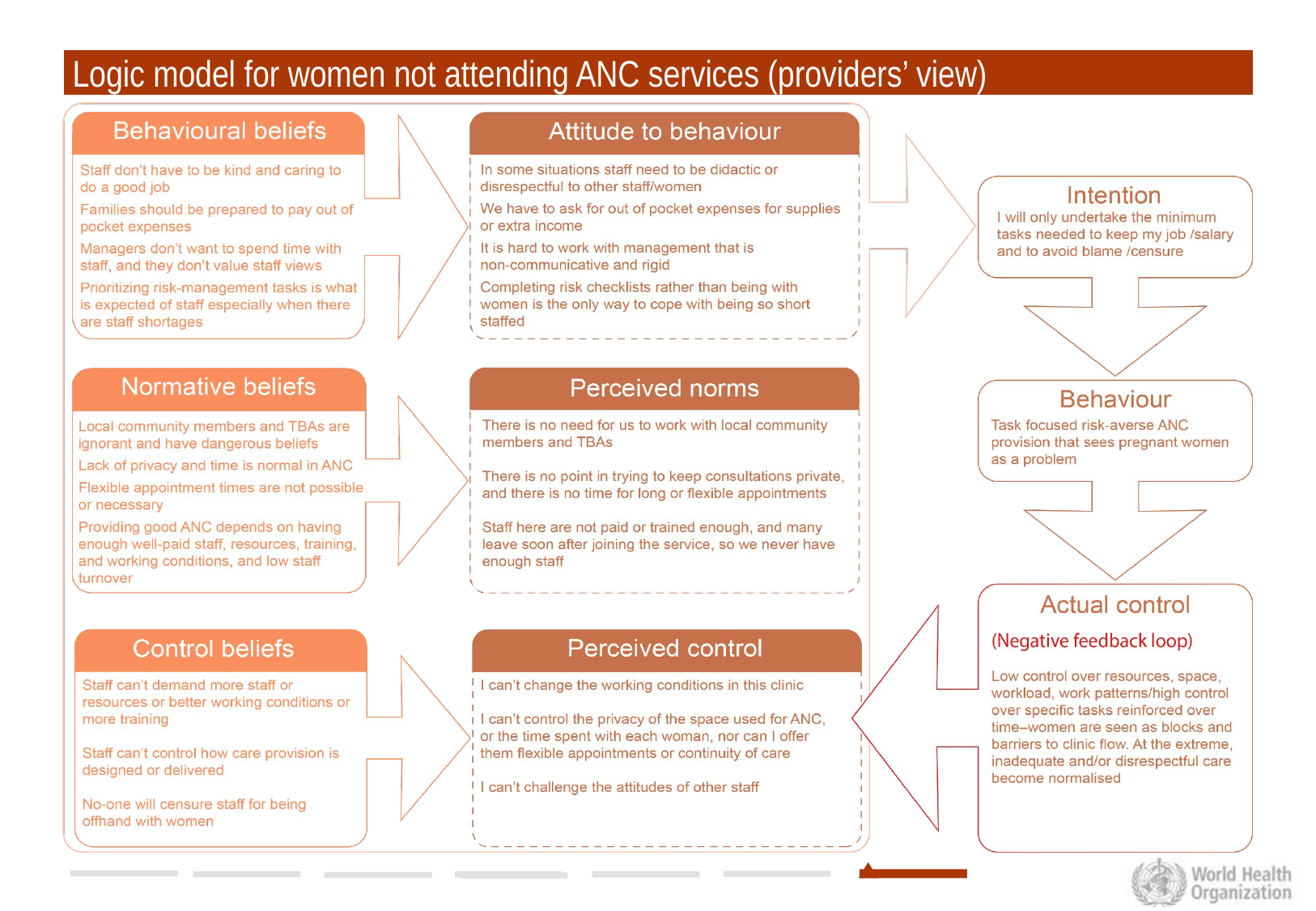

Logic model for women not attending ANC services (providers’ view)
Perceived Control
I can’t change the working conditions in this clinic
I can’t control the privacy of the space used for ANC, or the time spent with each woman, nor can I offer them flexible appointments or continuity of carer
I can’t challenge the attitudes of other staff
Perceived Control
I can’t change the working conditions in this clinic
I can’t control the privacy of the space used for ANC, or the time spent with each woman, nor can I offer them flexible appointments or continuity of carer
I can’t challenge the attitudes of other staff

## Slide 50
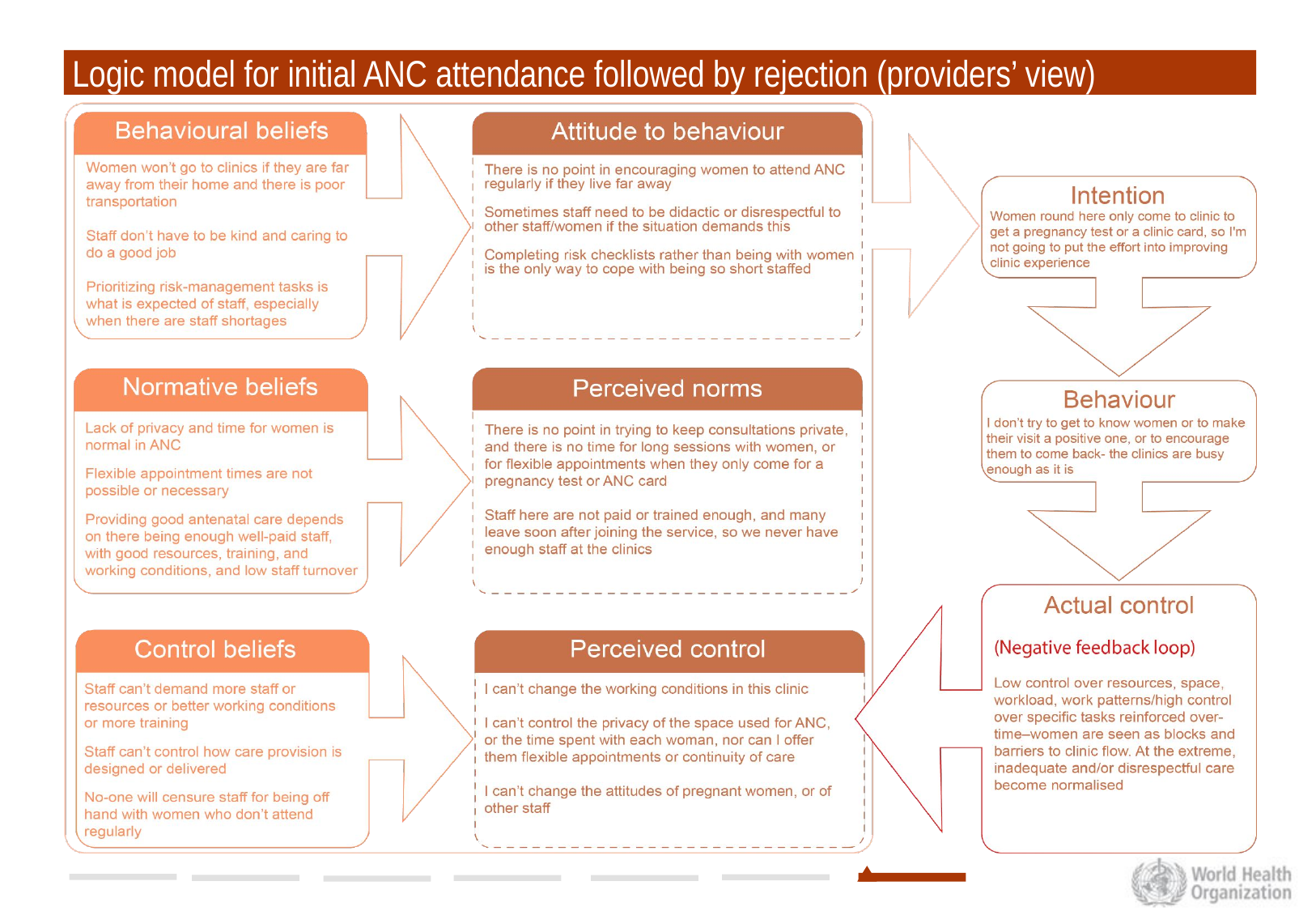

Logic model for initial ANC attendance followed by rejection (providers’ view)

## Slide 51
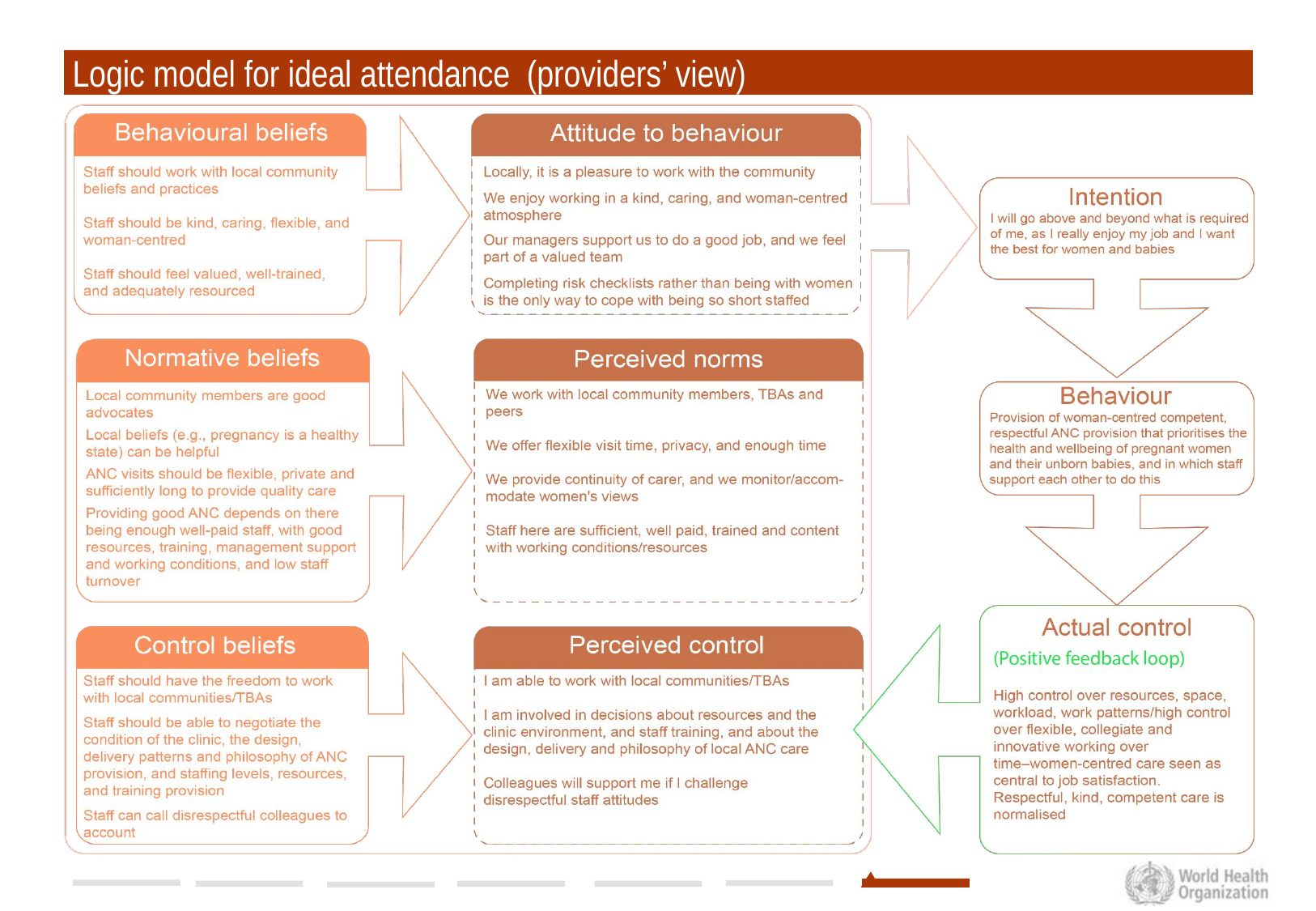

Logic model for ideal attendance (providers’ view)

## Slide 52
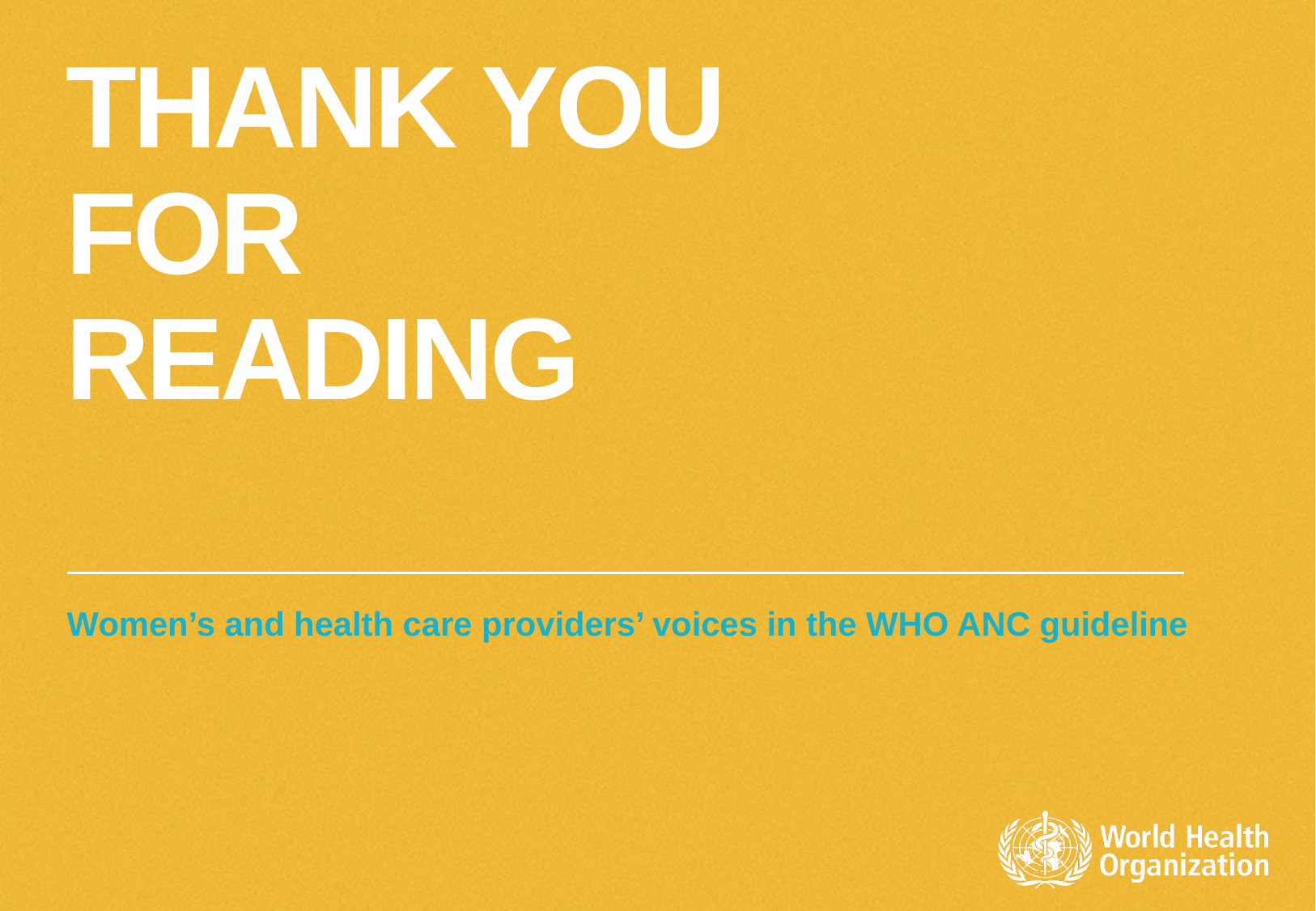

# Thank you for reading
Women’s and health care providers’ voices in the WHO ANC guideline
