# Supplementary material for: Development of the WHO Antenatal Care Recommendations Adaptation Toolkit: a standardised approach for countries
Source: Health Res Policy Syst. 2020 Jun 22;18:70. doi: 10.1186/s12961-020-00554-4 (PMC7310220; doi:10.1186/s12961-020-00554-4)
Supplement: Supplementary file 3 — Additional file 3. Instruction Manual for the WHO Antenatal Care Recommendations Adaptation Toolkit. [file 12961_2020_554_MOESM3_ESM.pdf]

World Health Organization

Instruction Manual for the WHO Antenatal Care Recommendations Adaptation Toolkit

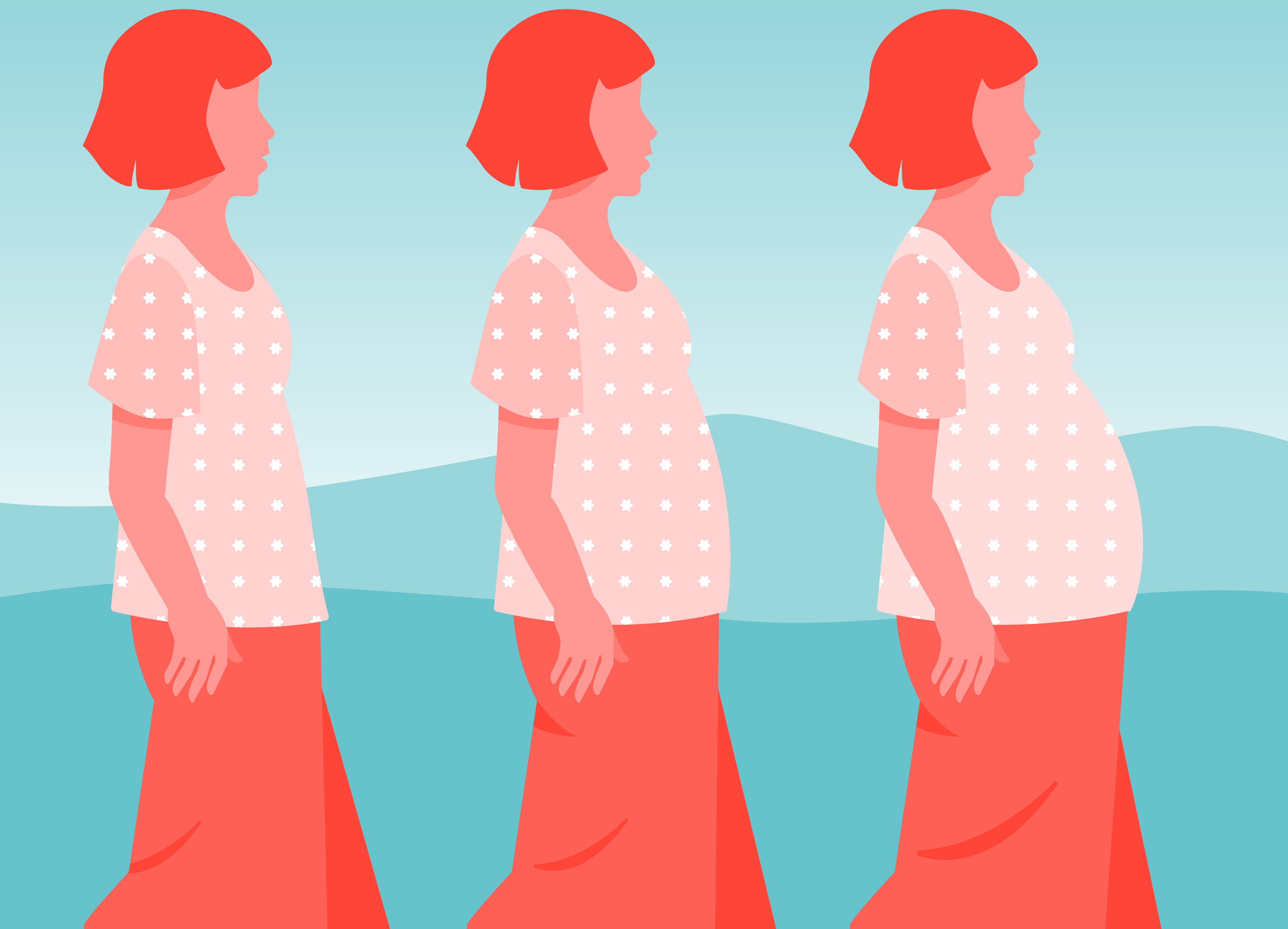

Instruction Manual for the WHO Antenatal Care Recommendations Adaptation Toolkit

World Health Organization

This instruction manual is published as an annex to the following manuscript:
Barreix M, Lawrie T, Kidula N, Tall F, Bucagu M, Chahar R, Tunçalp Ö. Development of the WHO Antenatal Care Recommendations Adaptation Toolkit: A standardized approach for countries. Health Research Policy and Systems. 2020; 18. DOI: 10.1186/s12961-020-00554-4.

The tools described in this manual can be accessed as supplementary materials to the article:

Additional file 1. Baseline Assessment Tool (BAT)
Additional file 2. Qualitative Evidence Synthesis (QES) Slidedoc®
Additional file 3. Instruction Manual for the WHO Antenatal Care Recommendations Adaptation Toolkit

Design and layout by Green Ink (www.greenink.co.uk)

Contents

1. Introduction to the WHO antenatal care recommendations adaptation toolkit **1**

2. Toolkit components **2**

3. Baseline assessment tool (BAT) for WHO recommendations on antenatal care for a positive pregnancy experience **4**
   - 3.1. Introduction 4
   - 3.2. Situational analysis (national) 5
   - 3.3. Recommendations mapping 7
   - 3.4. Output 1 – country-specific package of ANC interventions 8
   - 3.5. Output 2 – Strengths Weaknesses Opportunities and Threats analysis of new or updated recommendations 10

4. Considerations for the adaptation, scale-up and implementation of the 2016 WHO ANC model **11**

5. Implementation considerations for ANC guideline recommendations **13**

6. Remarks section from each recommendation **21**
   - A. Nutritional interventions 21
   - B.1 Maternal assessment 30
   - B.2 Fetal assessment 39
   - C. Preventive measures 42
   - D. Interventions for common physiological symptoms 48
   - E. Health systems interventions to improve the utilization and quality of ANC 51

7. The 2016 WHO ANC model for a positive pregnancy experience: recommendations mapped to eight scheduled ANC contacts **60**

8. Draft agenda for stakeholders' meeting **64**

9. Draft group-work materials for stakeholders' meeting **66**
   - GROUP 1 – PART I: Routine package of ANC for eight contacts 68
   - GROUP 2 – PART I: Routine package of ANC interventions for eight contacts 71

10. National ANC guideline table of contents template **73**

11. Example of package of ANC interventions **74**

References **80**

1. Introduction to the WHO antenatal care recommendations adaptation toolkit

On 7 November 2016, the World Health Organization (WHO) released its comprehensive recommendations on routine antenatal care (ANC) for pregnant women and adolescent girls *(1)*. The recommendations seek to respond to the complex nature of the issues surrounding the practice, organization and delivery of ANC within the health systems, and to prioritize person-centred care and well-being – not only the prevention of death and morbidity.

The WHO ANC guideline comprises recommendations related to antenatal nutrition, maternal and fetal assessment, preventive measures, interventions for common physiological symptoms (e.g. nausea, heartburn, constipation), as well as health systems interventions to improve ANC utilization and quality of care. In addition, current WHO recommendations on the treatment of malaria, tuberculosis and HIV for women during pregnancy were integrated to provide a consolidated package of interventions for routine ANC. The WHO ANC guideline emphasizes the importance of the Sustainable Development Goals (SDGs) for maternal and newborn health, and the attention the global health community must employ to attain them.

Many of the WHO ANC guideline's recommendations are context-specific *(2,3)*. Given the intricacies of the new eight-contact ANC model within health systems, outlined by the recommendations, and the known barriers to applying guidelines *(4)*, WHO has developed tools to support the implementation of the ANC recommendations.

The WHO designed the ANC recommendations adaptation toolkit described in this manual to assist national governments to systematically (i) adapt the ANC recommendations to their context and (ii) update their ANC policies according to the WHO ANC guideline. The toolkit's aim is to facilitate the building of country-specific packages of ANC interventions, including essential clinical (i.e. blood pressure, weight and height measurement, etc.) and counselling practices (i.e. birth preparedness, labour companion, etc.), tailored to the individual country contexts. The toolkit is also designed to highlight country-specific factors that are likely to influence (positively or negatively) the implementation of the stakeholder-approved country-specific package of ANC interventions, and which should be considered during the country implementation.

The toolkit can be used by ministries of health (MoHs), including district- and facility-level management, and other organizations. Within the MoH, the toolkit should be used by an interdisciplinary team, which includes programme managers from across the different vertical programmes as well as health management information system (HMIS) and supply chain managers. The process is generally led by the maternal health/safe motherhood focal person. The toolkit can also be used with support from in-country implementation partners and relevant stakeholders, when deemed necessary by the MoH.

This instruction manual aims to support policy-makers, programme managers and all other decision-makers complete the WHO ANC recommendations adaptation toolkit, with the ultimate goal of updating national ANC guidelines in accordance with WHO evidence-based recommendations.

2. Toolkit components

The WHO ANC recommendations adaptation toolkit, accompanying the WHO ANC guideline, comprises three main components:

Component 1: The baseline assessment tool (BAT)
The BAT consists of worksheets to evaluate a country's current ANC provision and practice and help plan for implementing the recommendations of the WHO ANC guideline. The worksheets are outlined in detail in section 3 of this instruction manual.

Component 2: Qualitative evidence synthesis (QES) Slidedocs® document[1]
A dual-purpose document to be read and referenced, outlining the qualitative data that helped shape the WHO ANC guideline's woman-centred perspective. This electronic presentation, built using the Duarte Slidedocs® format, details how QES was employed to inform the WHO ANC guideline (regarding women's values and the acceptability of the recommended interventions, counselling and health systems components of the ANC model). QES was also used to shape the entire guideline-development process, and explains the focus on a positive pregnancy experience. The QES Slidedoc® should be presented at the beginning of the stakeholders' meeting, to set the stage for the meeting, putting women's perspectives at the forefront. While the original Slidedoc® contains 55 slides/pages for reading, it can be reduced (e.g. to approximately 25 slides) for presentation purposes. It should be noted that a Slidedoc® is a dual-purpose tool that provides more information than a normal PowerPoint presentation because it is primarily meant for reading but can also be used for presenting.

Component 3: Supplementary materials
Within this instruction manual, users will find the toolkit's supplementary materials, some of which are drawn directly from the WHO ANC guideline. These include:

1. Considerations for the adaptation, scale-up and implementation of the 2016 WHO ANC model (section 4 of this instruction manual)
2. Implementation considerations for the ANC recommendations (section 5)
3. Remarks section from each recommendation (section 6)
4. The 2016 WHO ANC model for a positive pregnancy experience: recommendations mapped to eight scheduled ANC contacts (section 7)
5. Draft agenda for stakeholders' meeting (section 8)
6. Draft group-work materials for stakeholders' meeting (section 9)
7. National ANC guideline template (section 10)
8. Example of country-specific package of ANC interventions (section 11).

As mentioned, this toolkit was developed to assist policy-makers and programme managers to update national guidelines in line with the 2016 WHO ANC guideline.

Component 1 will be completed under the supervision of the maternal health programme manager (and his/her appointees) in collaboration with managers from across the MoH (e.g. malaria, adolescent health, and tuberculosis focal persons). A local consultant can be hired to support the entire country adaptation process. Consultants in previous implementation efforts had a medical background, were experienced and long-standing connections and relationships with MoH staff. Component 1 takes approximately one to two months to complete. Supplementary materials from Component 3 (list items 1–4 above) will aid the MoH team in this process.

Once Component 1 and the associated report summarizing the BAT situational analysis are completed (following internal MoH verification), these are to be shared at a two-day meeting of relevant ANC stakeholders. At the opening of this meeting, Component 2 will be presented in order to set the stage for the two-day session. National or state level stakeholders should be selected by the MoH officials. Overall, the following stakeholders should be considered for inclusion, depening on the setting: MoH programme managers related to ANC (including but not limited to TB, malaria, HIV, adolescent health, nursing, nutrition, M&E, HMIS, etc.); district heads/managers, as appropriate; professional associations (i.e. midwives, obstetricians/gynaecologists, nurses, etc.); United

1 Duarte. Slidedocs® (https://www.duarte.com/slidedocs/, accessed 28 November 2019).

Nations organizations (i.e. UNICEF and UNFPA); donor organizations with a strong presence in maternal health (i.e. DFID, USAID, etc.); international non-governmental organizations (NGOs) (i.e. Jhpiego, Clinton Health Access Initiative, etc.); local NGOs working in maternal health; community-based organizations working in maternal health; active women's groups, if any; private sector/faith-based organizations, if any; health worker unions, if any Stakeholders will also discuss: (i) the results of Component 1 and (ii) the components of the updated package of ANC interventions for the country. Supplementary materials from Component 3 (list items 5–8 above) will aid the MoH team to organize and hold the meeting.

Once the national guideline (including the country-specific integrated package of ANC interventions) has been prepared, countries may also conduct a two-day validation meeting, with the same stakeholders, to achieve national-level consensus and approval of both.

The entire process is further described in Barreix et al. *(5)*, to which this instruction manual is an annex.

3. Baseline assessment tool (BAT) for WHO recommendations on antenatal care for a positive pregnancy experience

The BAT contains five worksheets (and a cover sheet):

1. Introduction
2. Situational analysis (national) – designed for national level but could be tailor for use at the sub-national level
3. Recommendations mapping
4. Output 1 – country-specific package of ANC interventions
5. Output 2 - a strengths, weaknesses, opportunities and threats (SWOT) analysis of new or updated recommendations.

This section details how to complete each of the BAT worksheets.

3.1. Introduction

The BAT can be used to evaluate to what extent current ANC provision and practice in a country is in line with the recommendations in the 2016 WHO ANC guideline *(1)*. It can also help to plan activities to implement the recommendations. The term ANC refers to all the services that women need during pregnancy, including health promotion activities, maternal and fetal assessment, vaccination, preventive health measures, and management of human immunodeficiency virus (HIV), tuberculosis (TB), malaria and helminth infections.

The purpose of the BAT is to inform two outputs: Output 1, a draft integrated package of ANC interventions (to identify which bundles of interventions are applicable to a specific country or district context); and Output 2, a SWOT analysis for each of the new recommendations and scaling up best practices in service delivery. The SWOT analysis identifies the strengths, weaknesses, opportunities and threats to the successful implementation of any new or updated ANC services. It can also facilitate mid- and long-term service planning.

The BAT can be used by MoHs, including district- and facility-level management, and other organizations. While support for completing the BAT must come from the highest level of MoH policy-makers, the person overseeing or conducting the effort is typically the maternal health/safe motherhood programme manager.

The two data collection worksheets – the situational analysis (national level) and the recommendations mapping sheet – are there to help MoHs adapt and implement the WHO ANC guideline at country, district and facility levels. The situational analysis is loosely structured around the WHO's health systems building blocks *(6)*. The recommendations mapping sheet facilitates the plotting of current activities related to the clinical and health system interventions included in the guideline, with links to the considerations for implementation of recommended interventions. Useful documents, such as organograms and charts, can be copied and pasted into these data collection worksheets or added as hyperlinks. Data sheets can be supplemented or extended to include other local information that stakeholders consider to be useful.

The full guideline, *WHO recommendations on antenatal care for a positive pregnancy experience*, can be found at: https://www.who.int/reproductivehealth/publications/maternal_perinatal_health/anc-positive-pregnancy-experience/en/.

This BAT is based on similar NICE resources on ANC (available at: https://www.nice.org.uk/guidance/cg62/resources) and cerebral palsy (available at: https://www.nice.org.uk/guidance/ng62/resources).

3.2. Situational analysis (national)

The situational analysis worksheet is a data-collection guide, and is loosely adapted from the WHO health system building blocks *(6,7)*.

To complete the national-level situational analysis, the person responsible should consult existing data sources. These sources could include the national HMIS, demographic and health surveys, service availability and readiness assessments, national health accounts, research, etc. The point of consulting existing data sources is to minimize the cost and effort of completing the situational analysis. It is crucial that the person also liaises with programme managers across the MoH, including from the different vertical programmes, as well as with HMIS and supply chain managers.

Section A

This section seeks to find any direct feedback or perspectives from pregnant women in the country where the BAT is being implemented.

If routine feedback is not incorporated into the HMIS system, a brief search for qualitative studies of the national pregnant population could be conducted, either through an internet search or by reaching out to stakeholders associated with such research (universities, non-governmental organizations (NGOs), etc.). Additionally, the reference section of the review: "Provision and uptake of routine antenatal services: a qualitative evidence synthesis" in the Cochrane Database of Systematic Reviews could be a possible starting point for the search *(8)*.

Section B

This section captures a wide array of data on current ANC provision in the country. Questions (asking mostly for yes/no or numerical responses) are grouped as follows:

1. Leadership and governance
2. Health information systems
3. Service delivery
4. Health workforce
5. Financing
6. Access to essential medicines
7. Patient and population engagement
8. Existing model of ANC.

1. Leadership and governance

This section contains the leadership and governance questions, describing the political structure within which ANC health services are provided, as well as details of any additional actors, outside the MoH, and their roles. A current version of the MoH organogram should be attached in this section.

2. Health information systems

This section requests details of the existing data collection system (at facility and community level) and indicators used for decision-making in ANC programming at the national level. The first three items regard the routine data collection process at both facility (2.1) and community levels (2.3 and 2.5), as well as the types of health information systems employed (2.2) and what information is routinely gathered and monitored (2.4).

For item 2.4, indicator lists will vary depending on country context. An example of responses is as follows.

2.4	Other existing maternal and perinatal health indicators measured/monitored at country level by HMIS (expand table as necessary)			Regional or population* variation			
	Statistic	Data	Date (MM/YY)	Y	N	N/A	Describe variation, if any:
2.4.1	Percentage (%) of women receiving one ANC visit	81.0%	2017	YES			See ANNEX 8 for regional variations
2.4.2	Percentage (%) of women receiving four ANC visits	25.0%	2017		NO		
2.4.3	HIV prevalence among women of reproductive age (%)	15.0%	2013/2014			No data	
2.4.4	Percentage of women (%) receiving at least one dose of intermittent preventive treatment in pregnancy with sulfadoxine-pyrimethamine (IPTp) (malaria-endemic countries)	73%	2013/2014		NO		
2.4.5	Percentage (%) of ANC clients tested positive for HIV	0.6%	2017		NO		
2.4.6	Number of HIV positive pregnant women provided with Antiretrovirals (ARVs) for Prevention of mother-to-child transmission (PMTCT)	65 898	2017		NO		

* For example, among vulnerable populations such as migrants, adolescents, ethnic and racial minorities, indigenous women, women with disabilities, sex workers, HIV-positive women, displaced and war-affected women, women living in underserved areas and other stigmatized and excluded populations.

Digital technologies and other innovations, such as rapid diagnostic test are included in items 2.6 and 2.7, respectively.

Item 2.8 seeks a broad overview of maternal health in the country. Most of the indicators are derived from population-based surveys, such as demographic and health surveys; others are available only from aggregate national data (case fatality rate for top maternal causes of death – item 2.8.11) or by special inquiry (facilities with basic emergency obstetric care and comprehensive emergency obstetric care signal functions – items 2.8.12 and 13).

Item 2.9, which outlines population characteristics, is crucial for establishing the parameters within which context-specific recommendations are applied. For example, if the country is not malaria-endemic (item 2.9.11), the ANC recommendation in BAT worksheet 3 (Recommendations mapping) for recommendation C.6 on intermittent preventive treatment with sulfadoxine-pyrimethamine (IPTp-SP) does not apply. Please note that row 41 of the recommendations-mapping worksheet will automatically turn grey.

Additionally, for many questions in item 2.9, data might not be available. If this is the case, the associated recommendation should be included in worksheet 3 (Recommendation mapping) and discussed during the stakeholders' meeting (see section 9 of this instruction manual for stakeholders' meeting materials). An example of this could be: when dietary calcium intake (item 2.9.4) is unknown, calcium supplementation should be included in the draft national package of ANC interventions (Output 1) and presented to stakeholders. Lack of data for questions in population characteristics (item 2.9) may also identify research priorities for the country.

Finally, items 2.10 and 2.11 seeks further details on how and how often ANC indicators are reviewed (item 2.11) and when they were last revised (item 2.10).

3. Service delivery

This section seeks to identify at what level of the health system ANC services are currently provided (i.e. community, primary, secondary, tertiary levels), and to describe briefly the structure of the referral system. If any ANC outreach activities are conducted, they should be documented in item 3.1.6.

4. Health workforce

Item 4.1 seeks to identify existing human resources (focusing on the public sector) and practitioners' current in-service training (item 4.2) on ANC.

Item 4.3 records which cadre of practitioner (clinician, midwife, lay health worker, etc.) is allowed to complete which ANC-related tasks, such as taking blood pressure measurements, distributing nutrition supplements or performing ultrasound scans.

An example of responses in parts of item 4.3 is as follows.

4.3	Please indicate with cadre is allowed to perform the following ANC tasks	Advanced-level associate clinician	Associate clinician	Non-specialist doctor	Midwife	Auxiliary nurse midwife	Nurse	Auxillary nurse	Lay health worker	Other cadre
4.3.1	Maternal weight/height measurement	×	×	×	×	×	×	×	×	
4.3.2	Blood pressure measurement	×	×	×	×	×	×			
4.3.3	Clinical estimate of gestational age	×	×	×	×	×	×			
4.3.4	Fetal heart rate (FHR) auscultation	×	×	×	×	×	×			
4.3.5	Detection of breech presentation	×	×	×	×	×	×			
4.3.6	Vaginal examination	×	×	×	×	×	×			
4.3.7	Syphilis screening	×	×	×	×	×	×			
4.3.16	Iron/folic acid supplement dispensing	×	×	×	×	×	×	×	×	×
4.3.28	Counselling on postpartum contraception	×	×	×	×	×	×	×	×	×

5. Financing

The questions in item 5 refer to financing for ANC services, including available insurance schemes, if any, and whether ANC services have been costed. Item 5.4 seeks to identify which services pregnant women pay a fee for, according to government policies.

6. Access to essential medicines

This section seeks to understand the supply chain management system, specifically which ANC-related supplies are captured and monitored (for stock-outs) in district-level systems.

Item 6.3 expands on which ANC-related amenities (supplies and equipment, including for the laboratory) are required at which level of the health system. If a service availability and readiness assessment (SARA) *(9)* has recently been completed, the data can be incorporated in this section.

7. Patient and population engagement

This section seeks to uncover information regarding community-level participation, as well as any special programmes to address health inequities (i.e. programmes for vulnerable groups). Also, it seeks to identify any pregnancy surveillance programmes being implemented in the country.

8. Existing model of ANC

This section refers to the current model for providing ANC care, whether it's based on the focused ANC model *(10)* or another model. The current contact schedule for ANC services should be detailed here. Item 8.7 focuses on quality of care. Results from locally conducted qualitative research could also inform this section.

All of the results from this exercise should then be summarized in a narrative report that also highlights challenges and promising initiatives supporting the implementation of the country's current ANC model. The report should be internally validated by the MoH team (and, if deemed necessary, a select group of stakeholders) before being presented at the larger stakeholders' meeting (see section 9 of this instruction manual for stakeholders' meeting materials).

3.3. Recommendations mapping

This worksheet assists users to map the country's existing ANC policies to the recommendations in the WHO ANC guideline. It also shows which recommendations apply to the specific country setting, based on the population parameters (item 2.9). Please note that cells are linked to responses in BAT worksheet 2 – Situational analysis (national). Recommendations that are not applicable appear as grey cells.

Users are asked to compare how current activities (and related policies) align with each of the 49 ANC recommendations, who performs the index activity (main ANC provider or other), and whether a national policy change or update is necessary. Additionally, the MoH programme responsible for change should be identified, as well as a timeline for the update to take place.

An example of the mapping for recommendation C.1 is follows:

**Guideline reference**		**C.1**
**WHO recommendation**		A seven-day antibiotic regimen is recommended for all pregnant women with asymptomatic bacteriuria (ASB) to prevent persistent bacteriuria, preterm birth and low birth weight.
**Type of recommendation**		Recommended
**Is the recommendation relevant?/Does this recommendation apply to your context?**		Yes
**Current activity**	**– what is the current service provided related to this recommendation?**	Antibiotics for asymptomatic bacteriuria (ASB) are not currently given.
**Current activity**	**– is this service given by the primary ANC provided? If not, please expand.**	No
**Is the recommendation already implemented?**		No
**Any improvement required?**		Checklist and training updates are being planned for implementation in 2020.
**Responsible programme**		Maternal, Neonatal and Child Health Department
**Timeline for possible implementation**		Planned for Q1 2020

3.4. Output 1 – country-specific package of ANC interventions

Output 1, the country-specific package of ANC interventions, outlines all recommended ANC activities to be implemented during each of the eight contacts throughout the course of the pregnancy as in recommendation E.7 of the guideline. The 2016 WHO ANC guideline recommends eight contacts to be distributed as follows:

WHO focused ANC (FANC) model	2016 WHO ANC model
First trimester	
Visit 1: 8–12 weeks	Contact 1: up to 12 weeks
Second trimester	
Visit 2: 24–26 weeks	Contact 2: 20 weeks<br>Contact 3: 26 weeks
Third trimester	
Visit 3: 32 weeks	Contact 4: 30 weeks<br>Contact 5: 34 weeks
Visit 4: 36–38 weeks	Contact 6: 36 weeks<br>Contact 7: 38 weeks<br>Contact 8: 40 weeks
Return for delivery at 41 weeks if not given birth.	

The activities which will take place during each of the eight contacts are broken down into three categories which emerged from a scoping review *(11)* on what women want from ANC care:

- information
- medical interventions
- interpersonal support.

This output also outlines which health-care cadre provides each activity, the level of the health system at which the intervention will be provided, and the phasing, if necessary, to scale up that activity (e.g. the country may decide to phase the implementation of the ultrasound recommendation to procure equipment, train staff, etc.). This output is not automatically populated, and the person responsible for completing the toolkit should enter this information manually, based on the results of both worksheet 2 and 3.

An example of a contact description is as follows.

Contact	Activity	By health-care cadre	By health system level (including community and home)	Phasing if any	Support mechanisms (e.g. women's groups, companions)
Contact 1 (up to 12 weeks)	1. Nutritional counselling on healthy diet and physical activity<br>2. Nutritional education on increasing daily energy and protein intake<br>3. Balanced energy and protein supplementation in undernourished populations<br>4. Daily iron and folic acid supplements<br>5. Calcium supplements<br>6. Vitamin A supplements<br>7. Restricting caffeine intake	Auxiliary nurse midwife, nurse or midwife	Health centre	Phasing provision of these services to clinics in next 5 years	Ensure linkages with women's groups in the community (through community health workers)
	1. Detection of gestational diabetes mellitus (GDM)<br>2. Ultrasound scan<br>3. Enquiry on tobacco use and substance use<br>3. Clinical enquiry about the possibility of intimate partner violence<br>4. Diagnosis of anaemia in pregnancy<br>5. Testing and counselling for HIV and screening for TB<br>6. Antibiotics for asymptomatic bacteriuria (ASB)<br>7. Tetanus toxoid vaccination<br>8. Pre-exposure prophylaxis for HIV prevention<br>9. Nausea and vomiting management (if needed)<br>10. Heartburn, low back and pelvic pain, leg cramps, constipation, varicose veins management (if needed)<br>11. Essential good clinical practices and counselling – maternal weight/height measurement, blood pressure measurement, birth preparedness, family planning counselling, blood typing and Rhesus (Rh) factor blood testing, management of pain during labour	Nurse or midwife	Health centre	Phasing provision of these services to clinics in next 5 years	Counsel woman on her choice of birth companion

This worksheet (Output 1 – country-specific package of ANC interventions) will form the basis of the group-work materials for the stakeholders' meeting (see section 9 of this instruction manual). Stakeholders will review and validate the country-specific package of ANC interventions.

3.5. Output 2 – Strengths Weaknesses Opportunities and Threats analysis of new or updated recommendations

Output 2 consists of a strengths, weaknesses, opportunities and threats (SWOT) analysis. It should address the SWOT of all new or updated recommendations that the country will implement.

An example is as follows.

New/updated recommendation	Strengths	Weaknesses	Opportunities	Threats/ risks	Ongoing implementation/ research efforts
To introduce the ultrasound scan before 24 weeks of gestation at the health centre level	• Availability of qualified health providers to be trained on ultrasound service provision<br>• Existence of midwives/ nurses trained as ToTs	• Qualified health providers are not currently trained for ultrasound scan	• Commitment and alignment of partners and stakeholders (donor support for maternal health services)	• Cost of the equipment and maintenance	• Current implementation efforts by the MoH to equip 10 health centres and train 100 health-care providers in one district

* ToTs: training of trainers

4. Considerations for the adaptation, scale-up and implementation of the 2016 WHO ANC model

As described in the 2016 WHO ANC guideline *(1)* (page 117), policy-makers and stakeholders embarking on a national adaptation of these recommendations should take the following factors into consideration.

Health policy considerations for adoption and scale-up of the model.

- There needs to be a firm government commitment to scale up implementation of ANC services to achieve national coverage at health-care facilitates; national support must be secured for the whole package of ANC interventions rather than for specific components, to avoid fragmentation of services.
- In low-income countries, donors may play a significant role in scaling up the implementation of the model. Sponsoring mechanisms that support domestically driven processes to scale up the whole model are more likely to be helpful than mechanisms that support only a part of the package of ANC interventions.
- To set the policy agenda, to secure broad anchoring and to ensure progress in policy formulation and decision-making, stakeholders should be targeted among both elected and bureaucratic officials. In addition, representatives of training facilities and the relevant medical specialties should be included in participatory processes at all stages, including prior to an actual policy decision, to secure broad support for scaling up.
- To facilitate negotiations and planning, information on the expected impact of the model on users (i.e. health workers and pregnant women), providers (e.g. workload, training requirements) and costs should be assessed and disseminated.
- The model must be adapted to local contexts and service-delivery settings.

Health system or organizational-level considerations for implementation of the model.

- Introduction of the model should involve pre-service training institutions and professional bodies, so that training curricula for ANC can be updated as quickly and smoothly as possible.
- Long-term planning is needed for resource generation and budget allocation to strengthen and sustain high-quality ANC services.
- In-service training and supervisory models will need to be developed according to health-care providers' professional requirements, considering the content and duration, and procedures for the selection of providers for training. These models can also be explicitly designed to address staff turnover, particularly in low-resource settings.
- Standardized tools will need to be developed for supervision, ensuring that supervisors are able to support and enable health-care providers to deliver integrated, comprehensive ANC services.
- A strategy for task shifting may need to be developed to optimize the use of human resources.
- Tools or "job aids" for ANC implementation (e.g. ANC cards) will need to be simplified and updated with all key information in accordance with the model.
- Strategies will need to be devised to improve supply chain management according to local requirements, such as developing protocols for the procedures of obtaining and maintaining the stock of supplies, encouraging providers to collect and monitor data on the stock levels and strengthening the provider-level coordination and follow-up of medicines and health-care supplies required for implementation of the ANC model.

End-user-level considerations for implementation of the model.

- Community-sensitizing activities should be undertaken to disseminate information about the importance of each component of ANC, and pregnant women's right to attend ANC for their health and the health of their unborn baby. This information should provide details about the timing and content of the recommended ANC contacts, and about the expected user fees.
- It may be possible to reduce waiting times by reorganizing ANC services and/or client flow.
- Implementation considerations for ANC guideline recommendations.

5. Implementation considerations for ANC guideline recommendations

As described in the 2016 WHO ANC guideline *(1)* (page 145), policy-makers and stakeholders embarking on a national adaptation of the recommendations should consider the following factors. The implementation considerations and the remarks section from each recommendation aim to help toolkit users develop Outputs 1 and 2. Interventions that are not recommended are excluded from the implementation considerations table.

	Need to know	Need to do	Need to have	Consider
**A. Nutritional interventions**				
**A.1.1.** Nutritional counselling on a healthy diet and physical activity	• Healthy diet and exercise in local context<br>• Prevalence of overweight	• Counselling	• Counselling skills<br>• Time and space for counselling	• Gender issues and cultural expectations of women<br>• Local food security
**A.1.2.** Nutritional education on increasing daily energy and protein intake	• If your setting has an undernourished population<br>• How to do counselling	• Counselling	• Time to counsel<br>• Counselling skills	• Capacity-building for ANC providers on nutrition counselling<br>• Task shifting<br>• Group-based counselling
**A.1.3.** Balanced energy and protein supplementation in undernourished populations	• What balanced energy and protein supplementation means<br>• What is available locally that provides this	• Counselling	• Time to counsel<br>• Counselling skills	• Capacity-building for ANC providers on nutrition counselling<br>• Task shifting<br>• Group-based counselling
**A.1.4.** High protein supplementation in undernourished populations	• If this is in use	• If in use, advise against high protein supplementation during pregnancy	• N/A	• N/A
**A.2.** Iron and folic acid supplements	• That iron and folic acid is still recommended	• Counselling<br>• Dispensing	• Time to counsel<br>• Counselling skills<br>• Commodities management	• Timing of iron vs calcium dosing<br>• Community-based dispensing<br>• Task shifting<br>• Group-based counselling
**A.3.** Calcium supplements	• If dietary calcium is low in the local population	• Counselling<br>• Dispensing	• Time to counsel<br>• Counselling skills<br>• Commodities management	• Timing of iron vs calcium dosing<br>• Community-based dispensing<br>• Task shifting<br>• Group-based counselling

	Need to know	Need to do	Need to have	Consider
**A.4.** Vitamin A supplements	• If night blindness is endemic	• Counselling<br>• Dispensing	• Time to counsel<br>• Counselling skills<br>• Commodities management	• Referencing existing guideline<br>• Community-based dispensing<br>• Task shifting<br>• Group-based counselling
**A.10.** Restricting caffeine intake	• Whether local women typically have caffeine in their diet	• Counselling	• Counselling skills<br>• Time and space for counselling	• Gender issues and cultural norms for and expectations of women<br>• Task shifting
**B. Maternal and fetal assessment**				
**B.1. Maternal assessment**				
**B.1.1.** Diagnosing anaemia	• What method is in place to diagnose anaemia<br>• What method is feasible to start with<br>• How to interpret and manage	• Collect specimens<br>• Follow kit instructions<br>• Maintain infection control standards	• Capacity to conduct<br>• Kits<br>• Quality assurance/quality control (QA/QC) for any lab testing<br>• Commodities for treatment	• Switching to full blood count or haemoglobinometer method, if feasible
**B.1.2.** Diagnosing asymptomatic bacteriuria (ASB)	• What method is in place to diagnose ASB<br>• What method is feasible to start with<br>• How to interpret and manage	• Collect specimens<br>• Follow kit instructions<br>• Maintain infection control standards	• Capacity to conduct<br>• Kits<br>• QA/QC for any lab testing<br>• Commodities for treatment	• What levels of care are feasible for each type of test, with urine culture and sensitivity (C&S) being gold standard but dipstick sufficient in facilities without capacity
**B.1.3.** Enquiry about intimate partner violence (IPV)	• Local resources available to address IPV if identified during ANC<br>• How to enquire if WHO minimum requirements are in place[a]<br>• Country-level guidelines and policies	• Ask about IPV<br>• Counselling	• Well trained providers on first-line response<br>• Resources and referral mechanisms in place<br>• Time to counsel<br>• Sufficient confidential counselling space<br>• Counselling skills	• Forming linkages to supportive and social services if not already in place
**B.1.4.** Diagnosing gestational diabetes mellitus (GDM)	• National guidance/standard of care<br>• Guidelines for management of abnormal results<br>• Information on local context	• Counselling and testing	• Mechanisms and systems for testing and receiving results<br>• Time and space to counsel<br>• Counselling skills<br>• Commodity management for oral glucose solution and testing supplies<br>• QA/QC<br>• Clinical algorithm	• Reference existing guideline<br>• Feasibility and acceptability of screening strategies

	Need to know	Need to do	Need to have	Consider
**B.1.5.** Screening for tobacco use	• How to screen/ enquire	• Counselling	• Counselling skills<br>• Time to counsel	• Gender issues and cultural norms for and expectations of women<br>• Task shifting
**B.1.6.** Screening for alcohol and substance abuse	• Information on local context<br>• Local norms and behaviours around these risks<br>• Refer to the specific WHO guideline (12)	• Counselling and testing	• Time and space to counsel<br>• Counselling skills	• Referencing existing guideline<br>• Cultural context and local norms, impact of gender<br>• Impact of routine questioning in specific settings<br>• Task shifting<br>• Group-based counselling
**B.1.7.** HIV testing	• Retest women in high prevalence settings or in key high-risk groups	• Counselling and testing	• Commodities for testing<br>• Time to counsel<br>• Counselling skills<br>• Linkage to treatment	• Task shifting
**B.1.8.** Tuberculosis (TB) screening	• Population prevalence of TB	• Refer to the specific WHO guidance *(13)*		• Consider having TB clinics track pregnancy as a column in the register, to allow for better estimation of the local burden of TB in pregnancy
**B.2. Fetal assessment**				
**B.2.1.** Routine daily fetal movement (FM) counting	• If routine daily FM counting is being advised	• If ANC providers are advising daily FM counting in routine ANC counselling, instruct them to omit it, due to lack of evidence	• N/A	• N/A
**B.2.2.** Symphysis-fundal height (SFH) measurement	• What methods are being used for fetal growth and gestational age (GA) assessment	• Continue to include GA assessment and fetal growth assessment (by SFH or clinical palpation) in ANC contacts and documentation	• N/A	• No proven benefit to switching to fundal height measurement in settings where not currently in place
**B.2.3.** Routine antenatal cardiotocography (CTG)	• If routine antenatal CTG is being conducted	• If being conducted, instruct providers to omit this from practice, due to lack of evidence	• N/A	• N/A

	Need to know	Need to do	Need to have	Consider
**B.2.4.** Routine ultrasound scans	• Health system level<br>– Number and capacity of ultrasound providers to act as providers and trainers/mentors<br>– Number of functional machines available and geographic distribution<br>– Regulations around ultrasound use<br>– Cadres – who can perform?<br>– Available pre-service education and other certification<br>• Provider level<br>– Training to do anatomy scan or on referral<br>– How to interpret results and do counselling	• Health system level<br>– Determine appropriate settings and timeline for introduction of ultrasound<br>– Obtain machines<br>– Capacity-building plan<br>• Provider level<br>– Conduct or refer<br>– Document results<br>– Provide guidance on how to estimate GA and delivery date (EDD), depending on certainty of last menstrual period (LMP) and estimated GA at time of ultrasound, e.g. WHO's *Manual of diagnostic ultrasound (14)* and the American Institute of Ultrasound in Medicine (AIUM) guidelines *(15)*	• Health system level<br>– Transportation for women if services are not sufficiently decentralized<br>– Cadres with skills to provide quality services<br>• Facility level<br>– Machines<br>– Mechanism to review results and get reports<br>– Service contracts for machines<br>– Surge protection<br>– Power supply<br>– Counselling skills<br>– Security and environmental protection for costly machine<br>– Space for machine<br>– Ultrasound gel supply<br>– Staff and supplies to keep equipment clean	• Cost – of purchase, maintenance, training, impact of shifting resources to ultrasound from other key costs<br>• Local availability/feasibility of service contracts to support machine maintenance, especially in areas not previously prioritized for ultrasound market development<br>• Power supply – availability and stability<br>• Protection from power surges, which can permanently damage machines<br>• Extreme fragility of ultrasound transducers (one drop on a concrete floor may necessitate purchase of a new transducer, costing thousands of dollars)<br>• Relative benefits compared to other interventions<br>• Burden to mother<br>• Burden to providers and facility<br>• Creative, alternative models of service delivery that do not burden women with travel and related costs<br>• Feasibility studies in settings without widely available ultrasonography<br>• Studies on quality of ultrasound
**B.2.5.** Routine Doppler ultrasound	• If routine Doppler ultrasound is being conducted	• If being conducted, instruct providers to omit or consider in the context of research	• N/A	• Research context

	Need to know	Need to do	Need to have	Consider
**C. Preventive measures**				
**C.1.** Antibiotics for ASB	• What ASB is and how to diagnose it	• Prescribing<br>• Counselling	• Commodity management<br>• Counselling skills<br>• Time and space for counselling	• Capacity-building for providers in contexts where this is an unfamiliar concept and practice – value of treatment, risk of non-treatment, antibiotic stewardship/ avoidance of resistance
**C.2.** Antibiotic prophylaxis to prevent recurrent urinary tract infections	• Whether currently being performed	• Instruct to omit, if necessary	• N/A	• Research context
**C.3.** Antenatal anti-D immunoglobulin administration	• What is practised in the context	• Context-specific	• Availability of blood-typing	• Research context<br>• Recognize that this practice is routine in many high-resource settings; however, more evidence may be needed
**C.4.** Preventive anthelminthic treatment	• Local endemicity of helminth infections<br>• Local status of worm infestation-reduction programmes	• Provide or omit, depending on context	• Commodities management	• Task shifting<br>• Community-based distribution
**C.5.** Tetanus toxoid vaccination	• That this practice is still recommended<br>• Local prevalence of neonatal tetanus	• Provide vaccine according to established guidance	• Commodities management	• Consider quality improvement (QI) activities if gaps in coverage<br>• NOTE: Refer to dosing schedule in WHO 2006 guideline on maternal immunization against tetanus *(16)*
**C.6.** Intermittent preventive treatment in pregnancy	• See detailed implementation guidance in the specific WHO guideline on malaria *(17)*			• Emerging evidence on task shifting to community-based distribution<br>• Ways of ensuring that women receive the first dose at 13 weeks of gestation

	Need to know	Need to do	Need to have	Consider
**C.7.** Pre-exposure prophylaxis (PrEP) for HIV prevention	• Health system level: status of national PrEP guidelines and whether they include pregnant women, who to consider at substantial risk of HIV infection, sociocultural barriers to antiretroviral (ARV) use by HIV-uninfected pregnant women, availability of providers to counsel and train, availability of ARVs, cost to patients, capacity of laboratory to conduct recommended baseline and follow-up renal function tests<br>• Provider level: how to initiate and follow up, how to recognize renal toxicity, when to discontinue PrEP	• Health system: capacity-building plan<br>• Provider level: prescribe and/or dispense; counselling about the risks, benefits and alternatives to continuing to use PrEP during pregnancy and breastfeeding	• Commodities management<br>• Time and space for counselling, confidential dispensing	• Best mechanisms for the setting (ANC vs other)<br>• Stigma associated with ARV use<br>• Potential social harms to pregnant women, including IPV<br>• Pending evidence from the National Institute of Child Health and Human Development (NICHD) study on safety and feasibility of PrEP in pregnancy<br>• Additional research recommended by WHO and others<br>• Cost and frequency of stock-outs – distribution of drug for treatment vs PrEP
**D. Interventions for common physiological symptoms**				
**D.1–6**	• Cultural norms around treatment, harmful vs non-harmful practices	• Counselling	• Time to counsel<br>• Counselling skills	• Building ANC providers' capacity for counselling and listening, woman-centred care, etc.
**E. Health system interventions to improve utilization and quality of antenatal care**				
**E.1.** Woman-held case notes	• What is currently being used	• Ensure case notes are available in the appropriate language and at the appropriate education level for setting<br>• Adapt the case notes according to context<br>• Ensure durable product	• Commodities management<br>• Resources for production<br>• Method for retaining a facility copy	• What format is appropriate<br>• Whether it is necessary to exclude certain personal information to avoid stigmatization

	Need to know	Need to do	Need to have	Consider
**E.2.** Midwife-led continuity of care (MLCC)	• What model of care is currently being used<br>• Whether there are sufficient numbers of trained midwives<br>• Whether resources are available or can be shifted to facilitate this model	• Consult all relevant stakeholders, including human resource departments and professional bodies<br>• Assess the need for additional training in MLCC<br>• Ensure that that there is a well-functioning referral system in place<br>• Monitor midwife workload and burnout	• A well-functioning midwifery programme	• Strategies to scale up the quality and number of practising midwives<br>• Ways of providing continuity of care through other care providers, e.g. lay health workers<br>• Whether a caseload or team MLCC model is more appropriate
**E.3.** Group ANC	• Cultural norms and women's preferences regarding group ANC	• Consider evaluating in research context	• Appropriate facilities to deal with group sessions, including access to large, well-ventilated rooms, or sheltered spaces and adequate seating, and a private area for individual examination<br>• Providers trained in group facilitation and communication	• Research context
**E.4.** Community-based interventions to improve communication and support	• Community demographics and cultural norms<br>• Who are the key stakeholders in the community	• Train facilitators in group facilitation, convening public meetings, and communication techniques<br>• Ensure sufficient facilitators and resources to support them<br>• Train community volunteers/lay health workers to identify pregnant women in the community and encourage their attendance<br>• Ensure that the individual woman's preferences are respected, e.g. with regard to partner involvement	• Group spaces to hold meetings<br>• Culturally and educationally appropriate educational material, e.g. videos, flip charts, pictorial booklets and/or cards<br>• Ongoing supervision and monitoring of facilitators<br>• Resources, e.g. additional staff, transport and budget for material, for community mobilization initiatives	• Appropriate format and language of communication<br>• Whether meetings should include men and women together or separately<br>• Offering women a range of opportunities for communication and support, so that their individual preferences and circumstances can be catered for<br>• Implementing health system strengthening interventions, such as staff training, and improving equipment, transport, supplies, etc.

	Need to know	Need to do	Need to have	Consider
**E.5.** Task shifting components of ANC delivery	• Task shifting allows flexibility in certain contexts, but policy-makers need to work towards MLCC for all women	• Give health workers involved in task shifting a clear mandate<br>• Ensure that lay health workers are integrated into the health system and given appropriate supervision	• Ongoing supervision and monitoring<br>• Commodities management	• Refer to specific WHO guideline on task shifting *(18)*
**E.6.** Recruitment and retention of staff in rural and remote areas	• Refer to specific WHO guideline on recruitment and retention *(19)*			• Many pregnant women prefer receiving care from women health workers<br>• Personal safety can impact a woman health worker's decision to apply for, and remain in, rural positions<br>• Rotation of health workers from urban to rural areas and vice versa<br>• Agreeing the terms and period of rural deployment upfront
**E.7.** ANC contact schedules	• Timing and content and of ANC contacts<br>• How to adapt to local settings, e.g. which context-specific recommendations apply?, what can be task shifted?	• Secure national support for increased number of ANC contacts<br>• Conduct community sensitizing activities<br>• Involve pre-service training institutions and professional bodies<br>• Assess context-specific implications for resources, including staff, infrastructure, equipment, etc.	• Long-term planning and resource generation<br>• Provider training and supervision for newly introduced interventions<br>• Updated "job aids" (e.g. ANC case notes) that reflect changes<br>• Updated ANC training curricula and clinical manuals<br>• Ongoing supervision and monitoring	• Reorganizing services to reduce waiting times<br>• Other considerations can be found in Chapter 4 of the ANC guideline (Implementation of the ANC guideline and recommendations)

[a] Minimum requirements are: a protocol/standard operating procedure; training on how to ask about IPV, and on how to provide the minimum response or beyond; a private setting; confidentiality ensured; system for referral in place; and time to allow for appropriate disclosure.

6. Remarks section from each recommendation

The following boxes are taken directly from the 2016 WHO ANC guideline *(1)*. For more information please refer to the source document (available here: https://www.who.int/reproductivehealth/publications/maternal_perinatal_health/anc-positive-pregnancy-experience/en/). The remarks sections from each recommendation aim to help toolkit users develop Outputs 1 and 2.

A. Nutritional interventions

RECOMMENDATION A.1.1: Counselling about healthy eating and keeping physically active during pregnancy is recommended for pregnant women to stay healthy and to prevent excessive weight gain during pregnancy.[2] *(Recommended)*

Remarks

- A healthy diet contains adequate energy, protein, vitamins and minerals, obtained through the consumption of a variety of foods, including green and orange vegetables, meat, fish, beans, nuts, whole grains and fruit *(20)*.
- Stakeholders may wish to consider culturally appropriate healthy eating and exercise interventions to prevent excessive weight gain in pregnancy, particularly for populations with a high prevalence of overweight and obesity, depending on resources and women's preferences. Interventions should be woman-centred and delivered in a non-judgemental manner, and developed to ensure appropriate weight gain (see further information in points below).
- A healthy lifestyle includes aerobic physical activity and strength-conditioning exercise aimed at maintaining a good level of fitness throughout pregnancy, without trying to reach peak fitness level or train for athletic competition. Women should choose activities with minimal risk of loss of balance and fetal trauma *(21)*.
- Most normal gestational weight gain occurs after 20 weeks of gestation and the definition of "normal" is subject to regional variations, but should take into consideration pre-pregnant body mass index (BMI). According to the Institute of Medicine classification *(22)*, women who are underweight at the start of pregnancy (i.e. BMI < 18.5 kg/m^2) should aim to gain 12.5–18 kg, women who are normal weight at the start of pregnancy (i.e. BMI 18.5–24.9 kg/m^2) should aim to gain 11.5–16 kg, overweight women (i.e. BMI 25–29.9 kg/m^2) should aim to gain 7–11.5 kg, and obese women (i.e. BMI > 30 kg/m^2) should aim to gain 5–9 kg.
- Most evidence on healthy eating and exercise interventions comes from high-income countries (HICs), and the Guideline Development Group (GDG) noted that that there are at least 40 ongoing trials in HICs in this field. The GDG noted that research is needed on the effects, feasibility and acceptability of healthy eating and exercise interventions in low- and middle-income countries (LMICs).
- Pregnancy may be an optimal time for behaviour change interventions among populations with a high prevalence of overweight and obesity, and the longer-term impact of these interventions on women, children and partners needs investigation.
- The GDG noted that a strong training package is needed for practitioners, including standardized guidance on nutrition. This guidance should be evidence-based, sustainable, reproducible, accessible and adaptable to different cultural settings.

2 A healthy diet contains adequate energy, protein, vitamins and minerals, obtained through the consumption of a variety of foods, including green and orange vegetables, meat, fish, beans, nuts, whole grains and fruit.

Acceptability

Qualitative evidence indicates that women in a variety of settings tend to view ANC as a source of knowledge and information and that they generally appreciate any advice (including dietary or nutritional) that may lead to a healthy baby and a positive pregnancy experience (high confidence in the evidence) *(8)*. It also suggests that women may be less likely to engage with health services if advice is delivered in a hurried or didactic manner (high confidence in the evidence) *(8)*. Therefore, these types of interventions are more likely to be acceptable if the interventions are delivered in an unhurried and supportive way, which may also facilitate better engagement with ANC services. Qualitative evidence on health-care providers' views of ANC suggests that they may be keen to offer general health-care advice and specific pregnancy-related information (low confidence in the evidence) but they sometimes feel they do not have the appropriate training and lack the resources and time to deliver the service in the informative, supportive and caring manner that women want (high confidence in the evidence) *(8)*.

Feasibility

In a number of LMIC settings, providers feel that a lack of resources may limit implementation of recommended interventions (high confidence in the evidence) *(8)*.

RECOMMENDATION A.1.2: In undernourished populations, nutrition education on increasing daily energy and protein intake is recommended for pregnant women to reduce the risk of low-birth-weight neonates. *(Context-specific recommendation)*

Remarks

- Undernourishment is usually defined by a low BMI (i.e. being underweight). For adults, a 20–39% prevalence of underweight women is considered a high prevalence of underweight and 40% or higher is considered a very high prevalence *(23)*. Mid-upper arm circumference (MUAC) may also be useful to identify protein–energy malnutrition in individual pregnant women and to determine its prevalence in this population *(24)*. However, the optimal cut-off points may need to be determined for individual countries based on context-specific cost–benefit analyses *(24)*.
- Anthropometric characteristics of the general population are changing, and this needs to be taken into account by regularly reassessing the prevalence of undernutrition to ensure that the intervention remains relevant.
- The GDG noted that a strong training package is needed for practitioners, including standardized guidance on nutrition. This guidance should be evidence-based, sustainable, reproducible, accessible and adaptable to different cultural settings.
- Stakeholders might wish to consider alternative delivery platforms (e.g. peer counsellors, media reminders) and task shifting for delivery of this intervention.
- Areas that are highly food insecure or those with little access to a variety of foods may wish to consider additional complementary interventions, such as distribution of balanced protein and energy supplements (see Recommendation A.1.3).

Acceptability

Qualitative evidence indicates that women in a variety of settings tend to view ANC as a source of knowledge and information and that they generally appreciate any advice (including dietary or nutritional) that may lead to a healthy baby and a positive pregnancy experience (high confidence in the evidence) *(8)*. It also suggests that women may be less likely to engage with health services if advice is delivered in a hurried or didactic manner (high confidence in the evidence) *(8)*. Therefore, these types of interventions are more likely to be acceptable if the interventions are delivered in an unhurried and supportive way, which may also facilitate better engagement with ANC services. Qualitative evidence on health-care providers' views of ANC suggests that they may be keen to offer general health-care advice and specific pregnancy-related information (low confidence in the evidence) but they sometimes feel they do not have the appropriate training and lack the resources and time to deliver the service in the informative, supportive and caring manner that women want (high confidence in the evidence) *(8)*.

Feasibility

In a number of LMIC settings, providers feel that a lack of resources may limit implementation of recommended interventions (high confidence in the evidence) *(8)*.

RECOMMENDATION A.1.3: In undernourished populations, balanced energy and protein dietary supplementation is recommended for pregnant women to reduce the risk of stillbirths and small-for-gestational-age neonates. *(Context-specific recommendation)*

Remarks

- The GDG stressed that this recommendation is for populations or settings with a high prevalence of undernourished pregnant women, and not for individual pregnant women identified as being undernourished.
- Undernourishment is usually defined by a low BMI (i.e. being underweight). For adults, a 20–39% prevalence of underweight women is considered a high prevalence of underweight and 40% or higher is considered a very high prevalence *(23)*. MUAC may also be useful to identify protein–energy malnutrition in individual pregnant women and to determine its prevalence in this population *(24)*. However, the optimal cut-off points may need to be determined for individual countries based on context-specific cost–benefit analyses *(24)*.
- Establishment of a quality assurance process is important to guarantee that balanced energy and protein food supplements are manufactured, packaged and stored in a controlled and uncontaminated environment. The cost and logistical implications associated with balanced energy and protein supplements might be mitigated by local production of supplements, provided that a quality assurance process is established.
- A continual, adequate supply of supplements is required for programme success. This requires a clear understanding and investment in procurement and supply chain management.
- Programmes should be designed and continually improved based on locally generated data and experiences. Examples relevant to this guideline include:
  - Improving delivery, acceptability and utilization of this intervention by pregnant women (i.e. overcoming supply and utilization barriers).
  - Distribution of balanced energy and protein supplements may not be feasible only through the local schedule of ANC visits; additional visits may need to be scheduled. The costs related to these additional visits should be considered. In the absence of antenatal visits, too few visits, or when the first visit comes too late, consideration should be given to alternative platforms for delivery (e.g. community health workers, task shifting in specific settings).
  - Values and preferences related to the types and amounts of balanced energy and protein supplements may vary.
- Monitoring and evaluation should include evaluation of household-level storage facilities, spoilage, wastage, retailing, sharing and other issues related to food distribution.
- Each country will need to understand the context-specific etiology of undernutrition at the national and sub-national levels. For instance, where seasonality is a predictor of food availability, the programme should consider this and adapt to the conditions as needed (e.g. provision of more or less food of different types in different seasons). In addition, a better understanding is needed of whether alternatives to energy and protein supplements – such as cash or vouchers, or improved local and national food production and distribution – can lead to better or equivalent results.
- Anthropometric characteristics of the general population are changing, and this needs to be taken into account to ensure that only those women who are likely to benefit (i.e. only undernourished women) are included.
- The GDG noted that it is not known whether there are risks associated with providing this intervention to women with a high BMI.

**Acceptability**<br>Qualitative evidence indicates that women in a variety of settings tend to view ANC as a source of knowledge and information and that they generally appreciate any advice (including dietary or nutritional) that may lead to a healthy baby and a positive pregnancy experience (high confidence in the evidence) *(8)*. It also suggests that women may be less likely to engage with health services if advice is delivered in a hurried or didactic manner (high confidence in the evidence) *(8)*. Therefore, these types of interventions are more likely to be acceptable if the interventions are delivered in an unhurried and supportive way, which may also facilitate better engagement with ANC services. Qualitative evidence on health-care providers' views of ANC suggests that they may be keen to offer general health-care advice and specific pregnancy-related information (low confidence in the evidence) but they sometimes feel they do not have the appropriate training and lack the resources and time to deliver the service in the informative, supportive and caring manner that women want (high confidence in the evidence) *(8)*.
**Feasibility**<br>Providing balanced protein and energy supplements may be associated with logistical issues, as supplements are bulky and will require adequate transport and storage facilities to ensure continual supplies. Qualitative evidence from LMIC settings indicates that providers feel that a lack of resources may limit implementation of recommended interventions (high confidence in the evidence) *(8)*.

RECOMMENDATION A.2.1: Daily oral iron and folic acid supplementation with 30 mg to 60 mg of elemental iron[3] and 400 µg (0.4 mg) folic acid[4] is recommended for pregnant women to prevent maternal anaemia, puerperal sepsis, low birth weight, and preterm birth.[5] *(Recommended)*

Remarks

- This recommendation supersedes the 2012 WHO Guideline: daily iron and folic acid supplementation in pregnant women *(25)* and should be considered alongside Recommendation A.2.2 on intermittent iron.
- In settings where anaemia in pregnant women is a severe public health problem (i.e. where at least 40% of pregnant women have a blood haemoglobin [Hb] concentration < 110 g/L), a daily dose of 60 mg of elemental iron is preferred over a lower dose.
- In the first and third trimesters, the Hb threshold for diagnosing anaemia is 110 g/L; in the second trimester, the threshold is 105 g/L *(26)*.
- If a woman is diagnosed with anaemia during pregnancy, her daily elemental iron should be increased to 120 mg until her Hb concentration rises to normal (Hb 110 g/L or higher) *(27, 28)*. Thereafter, she can resume the standard daily antenatal iron dose to prevent recurrence of anaemia.
- Effective communication with pregnant women about diet and healthy eating – including providing information about food sources of vitamins and minerals, and dietary diversity – is an integral part of preventing anaemia and providing quality ANC.
- Effective communication strategies are vital for improving the acceptability of, and adherence to, supplementation schemes.
- Stakeholders may need to consider ways of reminding pregnant women to take their supplements and of assisting them to manage associated side-effects.
- In areas with endemic infections that may cause anaemia through blood loss, increased red cell destruction or decreased red cell production, such as malaria and hookworm, measures to prevent, diagnose and treat these infections should be implemented.
- Oral supplements are available as capsules or tablets (including soluble tablets, and dissolvable and modified-release tablets) *(29)*. Establishment of a quality assurance process is important to guarantee that supplements are manufactured, packaged and stored in a controlled and uncontaminated environment *(30)*.
- A better understanding of the etiology of anaemia (e.g. malaria endemicity, haemoglobinopathies) and the prevalence of risk factors is needed at the country level, to inform context-specific adaptations of this recommendation.
- Standardized definitions of side-effects are needed to facilitate monitoring and evaluation.
- Development and improvement of integrated surveillance systems are needed to link the assessment of anaemia and iron status at the country level to national and global surveillance systems.
- To reach the most vulnerable populations and ensure a timely and continuous supply of supplements, stakeholders may wish to consider task shifting the provision of iron supplementation in community settings with poor access to health-care professionals (see Recommendation E.6.1, in section E: Health systems interventions to improve the utilization and quality of ANC).

Acceptability

Qualitative evidence suggests that the availability of iron supplements may actively encourage women to engage with ANC providers (low confidence in the evidence) *(8)*. However, where there are additional costs associated with supplementation or where the supplements may be unavailable (because of resource constraints) women are less likely to engage with ANC services (high confidence in the evidence). Lower doses of iron may be associated with fewer side-effects and therefore may be more acceptable to women than higher doses.

3 The equivalent of 60 mg of elemental iron is 300 mg of ferrous sulfate hepahydrate, 180 mg of ferrous fumarate or 500 mg of ferrous gluconate.

4 Folic acid should be commenced as early as possible (ideally before conception) to prevent neural tube defects.

5 This recommendation supersedes the previous WHO recommendation found in the 2012 Guideline: daily iron and folic acid supplementation in pregnant women *(25)*.

Feasibility

Qualitative evidence about the views of health-care providers suggests that resource constraints, both in terms of the availability of the supplements and the lack of suitably trained staff to deliver them, may limit implementation (high confidence in the evidence) *(8)*.

RECOMMENDATION A.2.2: Intermittent oral iron and folic acid supplementation with 120 mg of elemental iron[6] and 2800 µg (2.8 mg) of folic acid once weekly is recommended for pregnant women to improve maternal and neonatal outcomes if daily iron is not acceptable due to side-effects, and in populations with an anaemia prevalence among pregnant women of less than 20%. *(Context-specific recommendation)*

Remarks

- This recommendation supersedes the previous WHO recommendation in the 2012 Guideline: intermittent iron and folic acid supplementation in non-anaemic pregnant women *(31)* and should be considered alongside Recommendation A.1.1.
- In general, anaemia prevalence of less than 20% is classified as a mild public health problem *(32)*.
- Before commencing intermittent iron supplementation, accurate measurement of maternal blood Hb concentrations is needed to confirm the absence of anaemia. Therefore, this recommendation may require a strong health system to facilitate accurate Hb measurement and to monitor anaemia status throughout pregnancy.
- If a woman is diagnosed with anaemia (Hb < 110 g/L) during ANC, she should be given 120 mg of elemental iron and 400 µg (0.4 mg) of folic acid daily until her Hb concentration rises to normal (Hb 110 g/L or higher) *(27, 28)*. Thereafter, she can continue with the standard daily antenatal iron and folic acid dose (or the intermittent regimen if daily iron is not acceptable due to side-effects) to prevent recurrence of anaemia.
- Stakeholders may need to consider ways of reminding pregnant women to take their supplements on an intermittent basis and of assisting them to manage associated side-effects.

Acceptability

Qualitative evidence suggests that the availability of iron supplements may actively encourage women to engage with ANC providers (low confidence in the evidence) *(8)*. However, where there are additional costs associated with supplementation or where the supplements may be unavailable (because of resource constraints) women are less likely to engage with ANC services (high confidence in the evidence). Women may find intermittent iron supplementation more acceptable than daily iron supplementation, particularly if they experience side-effects with daily iron supplements.

Feasibility

Intermittent iron may be more feasible in some low-resource settings if it costs less than daily iron.

6 The equivalent of 120 mg of elemental iron is 600 mg of ferrous sulfate hepahydrate, 360 mg of ferrous fumarate or 1000 mg of ferrous gluconate.

RECOMMENDATION A.3: In populations with low dietary calcium intake, daily calcium supplementation (1.5–2.0 g oral elemental calcium) is recommended for pregnant women to reduce the risk of pre-eclampsia. *(Context-specific recommendation)*

Remarks

- This recommendation is consistent with the 2011 WHO recommendations for prevention and treatment of pre-eclampsia and eclampsia *(33)* (strong recommendation, moderate-quality evidence) and supersedes the WHO recommendation found in the 2013 Guideline: calcium supplementation in pregnant women *(34)*.
- Dietary counselling of pregnant women should promote adequate calcium intake through locally available, calcium-rich foods.
- Dividing the dose of calcium may improve acceptability. The suggested scheme for calcium supplementation is 1.5–2 g daily, with the total dose divided into three doses, preferably taken at mealtimes.
- Negative interactions between iron and calcium supplements may occur. Therefore, the two nutrients should preferably be administered several hours apart rather than concomitantly *(34)*.
- As there is no clear evidence on the timing of initiation of calcium supplementation, stakeholders may wish to commence supplementation at the first ANC visit, given the possibility of compliance issues.
- To reach the most vulnerable populations and ensure a timely and continuous supply of supplements, stakeholders may wish to consider task shifting the provision of calcium supplementation in community settings with poor access to health-care professionals (see Recommendation E.6.1, in section E: Health systems interventions to improve the utilization and quality of ANC).
- The implementation and impact of this recommendation should be monitored at the health service, regional and country levels, based on clearly defined criteria and indicators associated with locally agreed targets. Successes and failures should be evaluated to inform integration of this recommendation into the package of ANC interventions.
- Further WHO guidance on prevention and treatment of pre-eclampsia and eclampsia is available in the 2011 WHO recommendations *(33)*, available at: https://apps.who.int/iris/bitstream/10665/44703/1/9789241548335_eng.pdf.

Acceptability

Qualitative evidence indicates that women in a variety of settings tend to view ANC as a source of knowledge and information and that they generally appreciate any advice (including dietary or nutritional) that may lead to a healthy baby and a positive pregnancy experience (high confidence in the evidence) *(8)*. However, calcium carbonate tablets might be unpalatable to many women, as they can be large and have a powdery texture *(35)*.In addition, this intervention usually involves taking three tablets a day, which significantly increasing the number of tablets a woman is required to take on a daily basis (i.e. in addition to iron and folic acid). This could have implications for both acceptability and compliance, which needs to be assessed in a programmatic context.

Feasibility

In addition to the cost, providing calcium supplements may be associated with logistical issues (e.g. supplements are bulky and require adequate transport and storage to maintain stock in facilities) and other challenges (e.g. forecasting). Qualitative evidence on health-care providers' views suggests that resource constraints may limit implementation (high confidence in the evidence) *(8)*.

RECOMMENDATION A.4: Vitamin A supplementation is only recommended for pregnant women in areas where vitamin A deficiency is a severe public health problem, to prevent night blindness. *(Context-specific recommendation)*

Remarks

- This recommendation supersedes the previous WHO recommendation found in the 2011 Guideline: vitamin A supplementation in pregnant women *(36)*.
- Vitamin A is not recommended to improve maternal and perinatal outcomes.
- Vitamin A deficiency is a severe public health problem if 5% or more of women in a population have a history of night blindness in their most recent pregnancy in the previous 3–5 years that ended in a live birth, or if 20% or more of pregnant women have a serum retinol level below 0.70 µmol/L *(37)*. Determination of vitamin A deficiency as a public health problem involves estimating the prevalence of deficiency in a population by using specific biochemical and clinical indicators of vitamin A status.
- Pregnant women should be encouraged to receive adequate nutrition, which is best achieved through consumption of a healthy, balanced diet, and to refer to WHO guidance on healthy eating *(20)*.
- In areas where supplementation is indicated for vitamin A deficiency, it can be given daily or weekly. Existing WHO guidance suggests a dose of up to 10 000 IU vitamin A per day, or a weekly dose of up to 25 000 IU *(36)*.
- A single dose of a vitamin A supplement greater than 25 000 IU is not recommended as its safety is uncertain. Furthermore, a single dose of a vitamin A supplement greater than 25 000 IU might be teratogenic if consumed between day 15 and day 60 from conception *(36)*.
- There is no demonstrated benefit from taking vitamin A supplements in populations where habitual daily vitamin A intakes exceed 8000 IU or 2400 µg, and the potential risk of adverse events increases with higher intakes (above 10 000 IU) if supplements are routinely taken by people in these populations *(38)*.

Acceptability

Qualitative evidence suggests that women in a variety of settings tend to view ANC as a source of knowledge and information and that they generally appreciate any advice (including dietary or nutritional) that may lead to a healthy baby and a positive pregnancy experience (high confidence in the evidence) *(8)*.

Feasibility

Qualitative evidence shows that where there are additional costs associated with supplements (high confidence in the evidence) or where the recommended intervention is unavailable because of resource constraints (low confidence in the evidence), women may be less likely to engage with ANC *(8)*.

RECOMMENDATION A.5: Zinc supplementation for pregnant women is only recommended in the context of rigorous research. *(Context-specific recommendation – research)*

Remarks

- Many of the included studies were at risk of bias, which influenced the certainty of the review evidence on the effects of zinc supplementation.
- The low-certainty evidence that zinc supplementation may reduce preterm birth warrants further investigation, as do the other outcomes for which the evidence is very uncertain (e.g. perinatal mortality, neonatal sepsis), particularly in zinc-deficient populations with no food fortification strategy in place. Further research should aim to clarify to what extent zinc supplementation competes with iron and/or calcium antenatal supplements for absorption. The GDG considered that food fortification may be a more cost-effective strategy and that more evidence is needed on the cost-effectiveness of food fortification strategies.

Acceptability

Qualitative evidence suggests that women in a variety of settings tend to view ANC as a source of knowledge and information and they generally appreciate any advice (including dietary or nutritional) that may lead to a healthy baby and a positive pregnancy experience (high confidence in the evidence) *(8)*.

Feasibility

It may be more feasible to fortify food with zinc rather than to provide zinc as a single supplement, particularly in settings with a high prevalence of stunting in children.

RECOMMENDATION A.10: For pregnant women with high daily caffeine intake (more than 300 mg per day),[7] lowering daily caffeine intake during pregnancy is recommended to reduce the risk of pregnancy loss and low-birth-weight neonates. *(Context-specific recommendation)*

Remarks

- Pregnant women should be informed that a high daily caffeine intake (> 300 mg per day) is probably associated with a higher risk of pregnancy loss and low birth weight.
- Caffeine is a stimulant found in tea, coffee, soft drinks, chocolate, kola nuts and some over-the-counter medicines. Coffee is probably the most common source of high caffeine intake. A cup of instant coffee can contain about 60 mg of caffeine; however, some commercially brewed coffee brands contain more than 150 mg of caffeine per serving.
- Caffeine-containing teas (black tea and green tea) and soft drinks (colas and iced tea) usually contain less than 50 mg per 250 mL serving.

Acceptability

Qualitative evidence indicates that women in a variety of settings generally appreciate any advice (including dietary or nutritional) that may lead to a healthy baby and a positive pregnancy experience (high confidence in the evidence) *(8)*. Evidence on health-care providers' views on ANC suggests that they may be keen to offer general health-care advice and specific pregnancy-related information (low confidence in the evidence) but they sometimes feel they do not have the appropriate training and lack the resources and time to deliver the service in the informative, supportive and caring manner that women want (high confidence in the evidence) *(8)*.

Feasibility

A lack of suitably trained staff to deliver health promotion interventions may limit implementation (high confidence in the evidence) *(8)*.

7 This includes any product, beverage or food containing caffeine (i.e. brewed coffee, tea, cola-type soft drinks, caffeinated energy drinks, chocolate, caffeine tablets).

B.1 Maternal assessment

RECOMMENDATION B.1.1: Full blood count testing is the recommended method for diagnosing anaemia during pregnancy. In settings where full blood count testing is not available, onsite haemoglobin testing with a haemoglobinometer is recommended over the use of the haemoglobin colour scale as the method for diagnosing anaemia in pregnancy. *(Context-specific recommendation)*

Remarks

- The GDG agreed that the high recurrent costs of Hb testing with haemoglobinometers might reduce the feasibility of this method in some low-resource settings, in which case the WHO haemoglobin colour scale method may be used.
- Other low-technology on-site methods for detecting anaemia need development and/or investigation.

Acceptability

Qualitative evidence from a variety of settings indicates that women generally appreciate clinical tests that support their well-being during pregnancy (moderate confidence in the evidence) *(8)*. However, evidence from LMICs indicates that where there are likely to be additional costs associated with tests, or where the recommended interventions are unavailable because of resource constraints, women may be less likely to engage with ANC services (high confidence in the evidence).

Feasibility

Qualitative evidence from providers in various LMICs indicates that a lack of resources, both in terms of the availability of the diagnostic equipment and potential treatments, as well as the lack of suitably trained staff to deliver the service, may limit implementation of recommended interventions (high confidence in the evidence) *(8)*.

RECOMMENDATION B.1.2: Midstream urine culture is the recommended method for diagnosing asymptomatic bacteriuria (ASB) in pregnancy. In settings where urine culture is not available, on-site midstream urine Gram staining is recommended over the use of dipstick tests as the method for diagnosing ASB in pregnancy. *(Context-specific recommendation)*

Remarks

- This recommendation should be considered alongside Recommendation C.1 on ASB treatment (see section C: Preventive measures).
- The GDG agreed that the higher resource costs associated with Gram stain testing might reduce the feasibility of this method in low-resource settings, in which case, dipstick tests may be used.
- The GDG agreed that ASB is a priority research topic, given its association with preterm birth and the uncertainty around urine testing and treatment in settings with different levels of ASB prevalence. Specifically, studies are needed that compare on-site testing and treatment versus testing plus confirmation of test with treatment on confirmatory culture, to explore health and other relevant outcomes, including acceptability, feasibility and antimicrobial resistance. In addition, better on-site tests need to be developed to improve accuracy and feasibility of testing and to reduce overtreatment of ASB. Research is also needed to determine the prevalence of ASB at which targeted testing and treatment rather than universal testing and treatment might be effective.

Acceptability

Qualitative evidence from a range of settings suggests that women view ANC as a source of knowledge, information and clinical expertise and that they generally appreciate the tests and advice they are offered (high confidence in the evidence) *(8)*. However, engagement with ANC services may be limited if tests and procedures are not explained properly or when women feel their beliefs and traditions are being overlooked or ignored by health-care professionals. In addition, if the Gram stain test is associated with long waiting times at ANC or having to return for test results, this may be less acceptable to women, as it might have additional cost and convenience implications for them (high confidence in the evidence). Health professionals are likely to prefer the dipstick test as it is associated with less effort (no need to label samples for laboratory assessment, perform tests or schedule follow-up visits to provide the results) and might provide additional information pertaining to other conditions (pre-eclampsia and diabetes mellitus) (high confidence in the evidence).

Feasibility

Qualitative evidence indicates that, in some LMIC settings, the lack of diagnostic equipment at ANC facilities discourages women from attending, and that providers often do not have the diagnostic equipment, supplies or skills to perform tests (high confidence in the evidence) *(8)*. Therefore, urine dipstick tests, which are cheaper and easy to perform, might be more feasible in low-resource settings.

RECOMMENDATION B.1.3: Clinical enquiry about the possibility of intimate partner violence (IPV) should be strongly considered at antenatal care visits when assessing conditions that may be caused or complicated by IPV in order to improve clinical diagnosis and subsequent care, where there is the capacity to provide a supportive response (including referral where appropriate) and where the WHO minimum requirements are met.[8] *(Context-specific recommendation)*

Remarks

- This recommendation is consistent with the 2013 publication *Responding to intimate partner violence and sexual violence against women: WHO clinical and policy guidelines (39)*. The evidence on clinical enquiry was indirect (strong recommendation) and the evidence on universal screening was judged as being of low to moderate quality (conditional recommendation).
- "Universal screening" or "routine enquiry" (i.e. asking all women at all health-care encounters) about IPV is not recommended. However, the WHO guidelines identify ANC as a setting where routine enquiry could be implemented if providers are well trained on a first-line response and minimum requirements are met *(39)*.
- Examples of conditions during pregnancy that may be caused or complicated by IPV include *(39)*:
  - traumatic injury, particularly if repeated and with vague or implausible explanations;
  - intrusive partner or husband present at consultations;
  - adverse reproductive outcomes, including multiple unintended pregnancies and/or terminations, delay in seeking ANC, adverse birth outcomes, repeated sexually transmitted infections (STIs);
  - unexplained or repeated genitourinary symptoms;
  - symptoms of depression and anxiety;
  - alcohol and other substance use;
  - self-harm, suicidality, symptoms of depression and anxiety.
- The GDG agreed that, despite a paucity of evidence, it was important to make a recommendation due to the high prevalence and significance of IPV. ANC provides an opportunity to enquire about IPV among women for whom barriers to accessing health care may exist, and also allows for the possibility for follow-up during ANC with appropriate supportive interventions, such as counselling and empowerment interventions. However, the evidence on benefits and potential harms of clinical enquiry and subsequent interventions is lacking or uncertain.
- A minimum condition for health-care providers to ask women about violence is that it must be safe to do so (i.e. the partner is not present) and that identification of IPV is followed by an appropriate response. In addition, providers must be trained to ask questions in the correct way and to respond appropriately to women who disclose violence *(39)*.
- Research on IPV is needed to answer the following questions:
  - Which are the most effective strategies for identifying, preventing and managing IPV in pregnancy?
  - Does asking routinely about violence impact on ANC attendance?
  - Can interventions targeted at partners of pregnant women prevent IPV?
- Detailed guidance on responding to IPV and sexual violence against women can be found in the 2013 WHO clinical and policy guidelines *(39)*, available at: https://www.who.int/reproductivehealth/publications/violence/9789241548595/en/.

8 Minimum requirements are: a protocol/standard operating procedure; training on how to ask about IPV, and on how to provide the minimum response or beyond; private setting; confidentiality ensured; system for referral in place; and time to allow for appropriate disclosure.

Acceptability
Qualitative evidence from a range of settings on women's views of ANC suggests that pregnant women would like to be seen by a kind and supportive health-care provider who has the time to discuss issues of this nature in a private setting (high confidence in the evidence) *(8)*. However, evidence from LMICs suggests that women may be unlikely to respond favourably to cursory exchanges of information with providers who they sometimes perceive to be hurried, uncaring and occasionally abusive (high confidence in the evidence). In addition, some women may not appreciate enquiries of this nature, particularly those living in male-dominated, patriarchal societies, where women's financial dependence on their husbands may influence their willingness to discuss IPV, especially if the health professional is male *(8)*. From the providers' perspective, qualitative evidence mainly from HICs suggests that providers often find it difficult to enquire about for IPV for the following reasons: they do not feel they have enough knowledge, training or time to discuss IPV in a sensitive manner; the presence of the partner acts as a barrier; they may have experienced IPV themselves; and they lack knowledge and guidance about the availability of additional support services (counselling, social work, etc.) (high confidence in the evidence). Providers highlight the midwife-led continuity of care (MLCC) model as a way of achieving a positive, trusting and empathetic relationship with pregnant women (moderate confidence in the evidence) (see Recommendation E.2, in section E: Health systems interventions to improve the utilization and quality of ANC).
**Feasibility**
Following IPV clinical enquiry, complex, multifaceted, culturally specific interventions are required to manage IPV, which could be challenging in many low-resource settings. However, emerging evidence from HICs shows that medium-duration empowerment counselling and advocacy/support, including a safety component, offered by trained health-care providers could be beneficial, and the feasibility of such interventions in LMIC settings needs investigation *(39)*.

RECOMMENDATION B.1.4: Hyperglycaemia first detected at any time during pregnancy should be classified as either gestational diabetes mellitus (GDM) or diabetes mellitus in pregnancy, according to WHO criteria.[9] *(Recommended)*

Remarks

- This recommendation has been integrated from the 2013 WHO publication *Diagnostic criteria and classification of hyperglycaemia first detected in pregnancy* (the strength of the recommendation and the quality of the evidence were not stated) *(40)*.
- WHO currently does not have a recommendation on whether or how to screen for GDM, and screening strategies for GDM are considered a priority area for research, particularly in LMICs.
- Diabetes mellitus in pregnancy differs from GDM in that the hyperglycaemia is more severe and does not resolve after pregnancy as it does with GDM.
- A systematic review of cohort studies shows that women with hyperglycaemia (diabetes mellitus and GDM) detected during pregnancy are at greater risk of adverse pregnancy outcomes, including macrosomia, pre-eclampsia/hypertensive disorders in pregnancy, and shoulder dystocia. Treatment of GDM, which usually involves a stepped approach of lifestyle changes (nutritional counselling and exercise) followed by oral blood-glucose-lowering agents or insulin if necessary, is effective in reducing these poor outcomes *(40)*.
- There are many uncertainties about the cost–effectiveness of different screening strategies, the prevalence of GDM and diabetes mellitus according to the 2013 criteria in diverse populations, and the impact of earlier diagnosis on pregnancy outcomes (see WHO ANC guideline – Chapter 5: Research implications) *(40)*.
- The usual window for diagnosing GDM is between 24 and 28 weeks of gestation. Risk-factor screening is used in some settings as a strategy to determine the need for a 2-hour 75 g oral glucose tolerance test (OGTT). These include a BMI of greater than 30 kg/m^2, previous GDM, previous macrosomia, family history of diabetes mellitus, and ethnicity with a high prevalence of diabetes mellitus *(41)*. In addition, glycosuria on dipstick testing (2+ or above on one occasion, or 1+ on two or more occasions) may indicate undiagnosed GDM and, if this is observed, performing an OGTT could be considered (41).
- The management approach for women classified with diabetes mellitus in pregnancy (i.e. severe hyperglycaemia first detected in pregnancy) usually differs from the approach for women with GDM, particularly when diagnosed early in pregnancy; however, the principles of management are similar and both require referral and increased monitoring.
- Further information and considerations related to this recommendation can be found in the 2013 WHO guideline *(40)*, available at: https://www.who.int/diabetes/publications/Hyperglycaemia_In_Pregnancy/en/.

9 This is not a recommendation on routine screening for hyperglycaemia in pregnancy. It has been adapted and integrated from the 2013 WHO publication (40), which states that GDM should be diagnosed at any time in pregnancy if one or more of the following criteria are met:
- fasting plasma glucose 5.1–6.9 mmol/L (92–125 mg/dL)
- 1-hour plasma glucose 10.0 mmol/L (180 mg/dL) following a 75 g oral glucose load
- 2-hour plasma glucose 8.5–11.0 mmol/L (153–199 mg/dL) following a 75 g oral glucose load.

Diabetes mellitus in pregnancy should be diagnosed if one or more of the following criteria are met:
- fasting plasma glucose 7.0 mmol/L (126 mg/dL)
- 2-hour plasma glucose 11.1 mmol/L (200 mg/dL) following a 75 g oral glucose load
- random plasma glucose 11.1 mmol/L (200 mg/dL) in the presence of diabetes symptoms.

RECOMMENDATION B.1.5: Health-care providers should ask all pregnant women about their tobacco use (past and present) and exposure to second-hand smoke as early as possible in pregnancy and at every antenatal care visit. *(Recommended)*

Remarks

- This strong recommendation based on low-quality evidence has been integrated from the 2013 WHO recommendations for the prevention and management of tobacco use and second-hand smoke exposure in pregnancy *(42)*. Related recommendations from this guideline include the following.
  - Health-care providers should routinely offer advice and psychosocial interventions for tobacco cessation to all pregnant women who are either current tobacco users or recent tobacco quitters (strong recommendation based on moderate quality evidence).
  - All health-care facilities should be smoke-free to protect the health of all staff, patients and visitors, including pregnant women (strong recommendation based on low-quality evidence).
  - Health-care providers should provide pregnant women, their partners and other household members with advice and information about the risks of second-hand smoke (SHS) exposure from all forms of smoked tobacco, as well as strategies to reduce SHS in the home (strong recommendation based on low-quality evidence).
  - Health-care providers should, wherever possible, engage directly with partners and other household members to inform them of all the risks of SHS exposure to pregnant women from all forms of tobacco, and to promote reduction of exposure and offer smoking cessation support (strong recommendation based on low-quality evidence).
- Further guidance on strategies to prevent and manage tobacco use and SHS exposure can be found in the 2013 WHO recommendations *(42)*, available at: https://www.who.int/tobacco/publications/pregnancy/guidelinestobaccosmokeexposure/en/.

RECOMMENDATION B.1.6: Health-care providers should ask all pregnant women about their use of alcohol and other substances (past and present) as early as possible in the pregnancy and at every antenatal care visit. *(Recommended)*

Remarks

- This strong recommendation based on low-quality evidence has been integrated from the 2014 WHO Guidelines for the identification and management of substance use and substance use disorders in pregnancy *(12)*. The overarching principles of this guideline aimed to prioritize prevention, ensure access to prevention and treatment services, respect women's autonomy, provide comprehensive care, and safeguard against discrimination and stigmatization.
- The GDG responsible for the recommendation noted that asking women at every ANC visit is important as some women are more likely to report sensitive information only after a trusting relationship has been established.
- Pregnant women should be advised of the potential health risks to themselves and to their babies posed by alcohol and drug use.
- Validated screening instruments for alcohol and other substance use and substance use disorders are available (refer to Annex 3 of the 2014 guidelines *(12)*).
- Health-care providers should be prepared to intervene or refer all pregnant women who are identified as using alcohol and/or drugs (past and present).
- For women identified as being dependent on alcohol or drugs, further recommendations from the guideline include the following:
  - Health-care providers should at the earliest opportunity advise pregnant women dependent on alcohol or drugs to cease their alcohol or drug use and offer, or refer them to, detoxification services under medical supervision, where necessary and applicable (strong recommendation based on very low-quality evidence).
  - Health-care providers should offer a brief intervention to all pregnant women using alcohol or drugs (strong recommendation based on low-quality evidence).
- It was decided that despite the low-quality evidence on effects of brief psychosocial interventions, the benefit (potential reduction of alcohol and substance use) outweighed any potential harms, which were considered to be minimal.
- A brief intervention is a structured therapy of short duration (typically 5–30 minutes) offered with the aim of assisting an individual to cease or reduce use of a psychoactive substance.
- Further guidance on interventions and strategies to identify and manage substance use and substance use disorders in pregnancy can be found in the 2014 WHO guidelines *(12)*, available at: https://www.who.int/substance_abuse/publications/pregnancy_guidelines/en/

RECOMMENDATION B.1.7: In high-prevalence settings,[10] provider-initiated testing and counselling (PITC) for HIV should be considered a routine component of the package of care for pregnancy women in all antenatal care settings. In low-prevalence settings, PITC can be considered for pregnant women in antenatal care settings as a key component of the effort to eliminate mother-to-child transmission of HIV, and to integrate HIV testing with syphilis, viral or other key tests, as relevant to the setting, and to strengthen the underlying maternal and child health systems. *(Recommended)*

Remarks

- This recommendation has been integrated from the 2015 WHO *Consolidated guidelines on HIV testing services (43)* (the strength of the recommendation and the quality of the evidence were not stated).
- PITC denotes an HIV testing service that is routinely offered in a health-care facility and includes providing pre-test information and obtaining consent, with the option for individuals to decline testing. PITC has proved highly acceptable and has increased the uptake of HIV testing in LMICs *(43)*.
- The availability of HIV testing at ANC services is responsible for the high level of knowledge of HIV status among women in many countries, which has allowed women and infants to benefit from ART.
- WHO recommends that ART should be initiated in all pregnant women diagnosed with HIV at any CD4 count and continued lifelong *(44)*. This recommendation is based on evidence that shows that providing ART to all pregnant and breastfeeding women living with HIV improves individual health outcomes, prevents mother-to-child transmission of HIV, and prevents horizontal transmission of HIV from the mother to an uninfected sexual partner.
- Other recommendations relevant to ANC services from the Consolidated guidelines on HIV testing services include the following *(43)*:
  - On disclosure: Initiatives should be put in place to enforce privacy protection and institute policy, laws and norms that prevent discrimination and promote tolerance and acceptance of people living with HIV. This can help create environments where disclosure of HIV status is easier (strong recommendation, low-quality evidence).
  - On retesting: In settings with a generalized HIV epidemic:[11] Retest all HIV-negative pregnant women in the third trimester, during labour or postpartum because of the high risk of acquiring HIV infection during pregnancy (strength of recommendation and quality of evidence not stated).
  - On retesting: In settings with a concentrated HIV epidemic:[12] Retest HIV-negative pregnant women who are in a serodiscordant couple or from a key population group[13] (strength of recommendation and quality of evidence not stated).
  - On retesting before ART initiation: National programmes should retest all people newly and previously diagnosed with HIV before they enrol in care and initiate ART (strength of recommendation and quality of evidence not stated).
  - On testing strategies: In settings with greater than 5% HIV prevalence in the population being tested, a diagnosis of HIV-positive should be issued to people with two sequential reactive tests. In settings with less than 5% HIV prevalence in the population being tested, a diagnosis of HIV-positive should be issued to people with three sequential reactive tests (strength of recommendation and quality of evidence not stated).
  - On task shifting: Lay providers who are trained and supervised can independently conduct safe and effective HIV testing using rapid diagnostic tests (strong recommendation, moderate-quality evidence).
- Further guidance on HIV testing can be found in the 2015 WHO guidelines *(43)*, available at: https://www.who.int/hiv/pub/guidelines/hiv-testing-services/en/.

10 High-prevalence settings are defined in the 2015 WHO publication Consolidated guidelines on HIV testing services as settings with greater than 5% HIV prevalence in the population being tested. Low-prevalence settings are settings with less than 5% HIV prevalence in the population being tested (44).

11 A generalized HIV epidemic is when HIV is firmly established in the general population. Numerical proxy: HIV prevalence is consistently over 1% in pregnant women attending antenatal clinics *(44)*.

12 A concentrated HIV epidemic is when HIV has spread rapidly in a defined subpopulation (or key population, see next footnote) but is not well established in the general population *(44)*.

13 Key populations are defined in the 2015 WHO guidelines as the following groups: men who have sex with men, people in prison or other closed settings, people who inject drugs, sex workers and transgender people *(44)*.

- In addition, the 2015 Guideline on when to start antiretroviral therapy and on pre-exposure prophylaxis for HIV *(44)* is available at: http://www.who.int/hiv/pub/guidelines/earlyrelease-arv/en/.
- To prevent mother-to-child transmission of syphilis, all pregnant women should be screened for syphilis at the first ANC visit in the first trimester and again in the third trimester of pregnancy. For further guidance on screening, please refer to the 2006 WHO publication *Prevention of mother-to-child transmission of syphilis (45)*, available at: https://www.who.int/reproductivehealth/publications/maternal_perinatal_health/prevention_mtct_syphilis.pdf
- The latest (2016) WHO guidelines on the treatment of chlamydia, gonorrhoea and syphilis, and on the prevention of sexual transmission of Zika virus *(46–49)*, are available at: https://www.who.int/reproductivehealth/publications/rtis/clinical/en/.

RECOMMENDATION B.1.8: In settings where the tuberculosis (TB) prevalence in the general population is 100/100 000 population or higher, systematic screening for active TB should be considered for pregnant women as part of antenatal care. *(Context-specific recommendation)*

Remarks

- This recommendation has been adapted and integrated from the 2013 WHO publication *Systematic screening for active tuberculosis: principles and recommendations*, where it was considered a conditional recommendation based on very low-quality evidence *(13)*.
- Systematic screening is defined as the systematic identification of people with suspected active TB in a predetermined target group, using tests, examinations or other procedures that can be applied rapidly. Options for initial screening include screening for symptoms (either for cough lasting longer than two weeks, or any symptoms compatible with TB, including a cough of any duration, haemoptysis, weight loss, fever or night sweats) or screening with chest radiography. The use of chest radiography in pregnant women poses no significant risk but the national guidelines for the use of radiography during pregnancy should be followed *(13)*.
- Before screening is initiated, high-quality TB diagnosis, treatment, care, management and support should be in place, and there should be the capacity to scale these up further to match the anticipated rise in case detection that may occur as a result of screening.
- The panel responsible for making this recommendation noted that it may not be possible to implement it in resource-constrained settings.
- Other recommendations relevant to ANC services from the same publication include the following *(13)*:
  - Household contacts and other close contacts should be systematically screened for TB (strong recommendation, very low-quality evidence).
  - People living with HIV should be systematically screened for active TB at each visit to a health-care facility (strong recommendation, very low-quality evidence).
  - Systematic screening for active TB may be considered also for other subpopulations that have very poor access to health care, such as people living in urban slums, homeless people, people living in remote areas with poor access to health care, and other vulnerable or marginalized groups including some indigenous populations, migrants and refugees (conditional recommendation, very low-quality evidence).
- TB increases the risk of preterm birth, perinatal death and other pregnancy complications. Initiating TB treatment early is associated with better maternal and infant outcomes than late initiation *(13)*.
- To better understand the local burden of TB in pregnancy, health systems may benefit from capturing pregnancy status in registers that track TB screening and treatment.
- Further information and considerations related to this recommendation can be found in the 2013 WHO recommendations *(13)*, available at: https://www.who.int/tb/tbscreening/en/.

B.2 Fetal assessment

RECOMMENDATION B.2.1: Daily fetal movement counting, such as with "count-to-ten" kick charts, is only recommended in the context of rigorous research. *(Context-specific recommendation – research)*

Remarks

- Fetal movement counting is when a pregnant woman counts and records her baby's movements in order to monitor the baby's health. Various methods have been described, with further monitoring variously indicated depending on the method used, for example, if fewer than six distinct movements are felt within 2 hours *(50)* or fewer than 10 distinct movements are felt within 12 hours (the Cardiff "count to ten" method) *(51)*.
- While daily fetal movement counting is not recommended, healthy pregnant women should be made aware of the importance of fetal movements in the third trimester and of reporting reduced fetal movements.
- Clinical enquiry by ANC providers at each ANC visit about maternal perception of fetal movements is recommended as part of good clinical practice. Women who perceive poor or reduced fetal movements require further monitoring (e.g. with daily fetal movement counting) and investigation, if indicated.
- The GDG agreed that more research is needed on the effects of daily fetal movement counting in the third trimester of pregnancy, particularly in LMIC settings with a high prevalence of unexplained stillbirths.

Acceptability

Qualitative evidence shows that women generally appreciate the knowledge and information they can acquire from health-care providers during ANC visits, provided this is explained properly and delivered in a consistent, caring and culturally sensitive manner (high confidence in the evidence) *(8)*. It also shows that health professionals want to give appropriate information and advice to women but sometimes they don't feel suitably trained to do so (high confidence in the evidence) *(8)*.

Feasibility

From the perspective of women who live far from ANC clinics and who may not have the resources or time to attend ANC regularly, and the perspective of ANC providers with limited resources, this intervention may offer a practical and cost-effective approach to monitoring fetal well-being if it's shown to be effective (high confidence in the evidence) *(8)*.

RECOMMENDATION B.2.2: Replacing abdominal palpation with symphysis-fundal height (SFH) measurement for the assessment of fetal growth is not recommended to improve perinatal outcomes. A change from what is usually practiced (abdominal palpation or SFH measurement) in a particular setting is not recommended. *(Context-specific recommendation)*

Remarks

- SFH measurement is routinely practiced in many ANC settings. Due to a lack of clear evidence of accuracy or superiority of either SFH measurement or clinical palpation to assess fetal growth, the GDG does not recommend a change of practice.
- The GDG agreed that there is a lack of evidence on SFH, rather than a lack of effectiveness, particularly in LMIC settings.
- Apart from false reassurance, which might occur with both SFH measurement and clinical palpation, there is no evidence of harm with SFH measurement.
- Research is needed to determine the role of SFH measurement in detecting abnormal fetal growth and other risk factors for perinatal morbidity (e.g. multiple pregnancy, polyhydramnios) in settings where antenatal ultrasound is not available.

Acceptability

SFH and clinical palpation are non-invasive approaches for fetal assessment, which are widely used and not known to be associated with acceptability issues. However, in some settings women experience a sense of shame during physical examinations, and this needs to be addressed with sensitivity by health-care providers (low confidence in the evidence) *(8)*.

Feasibility

Both methods are considered equally feasible, provided tape measures are available.

RECOMMENDATION B.2.4: One ultrasound scan before 24 weeks of gestation (early ultrasound) is recommended for pregnant women to estimate gestational age, improve detection of fetal anomalies and multiple pregnancies, reduce induction of labour for post-term pregnancy, and improve a woman's pregnancy experience. *(Recommended)*

Remarks

- The benefits of an early ultrasound scan are not improved upon and cannot be replicated with a late ultrasound scan where there has not been an early ultrasound scan. Therefore, an ultrasound scan after 24 weeks of gestation (late ultrasound) is not recommended for pregnant women who have had an early ultrasound scan. However, stakeholders should consider offering a late ultrasound scan to pregnant women who have not had an early ultrasound scan, for the purposes of identifying the number of fetuses, presentation and placental location.
- The GDG noted that the effects of introducing antenatal ultrasound on population health outcomes and health systems in rural, low-resource settings are unproven. However, the introduction of ultrasound to detect pregnancy complications and confirm fetal viability to the woman and her family in these settings could plausibly increase ANC service utilization and reduce morbidity and mortality, when accompanied by appropriate gestational age estimation, diagnosis, referral and management.
- The ongoing multicountry trial that is under way should contribute further evidence on health effects, health-care utilization and implementation-related information on ultrasound in rural, low-resource settings *(52)*.
- The GDG acknowledged that the use of early pregnancy ultrasound has not been shown to reduce perinatal mortality. The GDG put emphasis on other benefits of ultrasound (mentioned in points above) and the increased accuracy of gestational age assessment, which would assist management in case of suspected preterm birth and reduce labour induction for post-term pregnancies.
- The GDG acknowledges that implementing and scaling up this recommendation in low-resource settings will be associated with a variety of challenges that may include political (budgeting for fees and tariffs), logistical (equipment maintenance, supplies, technical support), infrastructural (ensuring a reliable power supply and secure storage) and resources.
- The GDG noted that antenatal ultrasound is an intervention that can potentially be task shifted from trained sonographers and doctors to trained nurses, midwives and clinical officers, provided that ongoing training, staff retention, quality improvement activities and supervision are ensured.
- Stakeholders might be able to offset/reduce the cost of antenatal ultrasound if the ultrasound equipment is also used for other indications (e.g. obstetric emergencies) or by other medical departments.
- The implementation and impact of this recommendation on health outcomes, facility utilization and equity should be monitored at the health service, regional and country levels, based on clearly defined criteria and indicators associated with locally agreed targets.[14]
- For further guidance, please refer to the WHO *Manual of diagnostic ultrasound (53)*, available at: https://www.who.int/medical_devices/publications/manual_ultrasound_pack1-2/en/.

14 Two members of the GDG (Lisa Noguchi and Charlotte Warren) indicated that they would prefer to recommend this intervention in specific contexts with capacity to conduct close monitoring and evaluation to ensure a basic standard of implementation (including adequate capacity to diagnose and manage complications) and monitor for potential adverse effects on delivery of other critical maternal and newborn health interventions.

Acceptability

Qualitative evidence shows that women generally appreciate the knowledge and information they can acquire from health-care providers and that they are willing to be screened and tested for a variety of conditions, provided the information and procedures are explained properly and delivered in a caring and culturally sensitive manner (high confidence in the evidence) *(8)*. Evidence also shows that, in some LMICs, the lack of modern technology (like ultrasound equipment) at ANC facilities discourages some women from attending (high confidence in the evidence) *(8)*. This suggests that the offer of ultrasound might attract women to use ANC facilities, which may also lead to earlier ANC attendance. Specific studies not included in the main qualitative review indicate that women value the opportunity to see their baby via ultrasound and find the test reassuring *(54)*. However, there is some evidence that women do not understand that ultrasound is a diagnostic tool, and that adverse findings during scans might increase anxiety and distress *(55)*. Qualitative evidence from health-care providers shows that they generally want to provide screening and testing procedures, but sometimes don't feel suitably trained to do so (high confidence in the evidence) *(8)*. This suggests that they might welcome ultrasound scans to assist with accurate gestational age estimation and to identify potential risk factors, such as multiple pregnancies, if appropriately trained and supported.

Feasibility

Feasibility challenges of antenatal ultrasound scans in LMICs includes equipment procurement and staff training, ensuring a power supply (via a power point or rechargeable batteries) and secure storage, regular equipment maintenance, maintaining adequate and continual supplies of ultrasound gel, and ongoing technical support and supervision.

C. Preventive measures

RECOMMENDATION C.1: A seven-day antibiotic regimen is recommended for all pregnant women with asymptomatic bacteriuria (ASB) to prevent persistent bacteriuria, preterm birth and low birth weight. *(Recommended)*

Remarks

- This recommendation should be considered alongside the recommendation on ASB diagnosis (Recommendation B.1.2).
- Stakeholders may wish to consider context-specific ASB screening and treatment based on ASB and preterm birth prevalence, as it may not be appropriate in settings with low prevalence.
- Evidence on preterm birth is of low certainty and large multicentre trials are needed to confirm whether screening and antibiotic treatment reduces preterm birth and perinatal mortality in LMICs. Such trials should also aim to evaluate the effects of group B streptococcus (GBS) screening and treatment.
- Studies have shown that GBS bacteriuria is a sign of heavy GBS colonization, which may not be eradicated by antibiotic treatment. GBS bacteriuria is a risk factor for having an infant with early onset GBS disease. WHO recommends that pregnant women with GBS colonization receive intrapartum antibiotic administration to prevent early neonatal GBS infection (see WHO recommendations for prevention and treatment of maternal peripartum infections *[56]*).
- Preterm birth indicators should be monitored with this intervention, as should changes in antimicrobial resistance.

Acceptability

In LMICs, some women hold the belief that pregnancy is a healthy condition and may not accept the use of antibiotics in this context (particularly if they have no symptoms) unless they have experienced a previous pregnancy complication (high confidence in the evidence) *(8)*. Others view ANC as a source of knowledge, information and medical safety, and generally appreciate the interventions and advice they are offered (high confidence in the evidence). However, engagement may be limited if this type of intervention is not explained properly. In addition, where there are likely to be additional costs associated with treatment, women are less likely to engage (high confidence in the evidence).

Feasibility

A lack of resources in LMICs, both in terms of the availability of the medicines and testing, and the lack of suitably trained staff to provide relevant information and perform tests, may limit implementation (high confidence in the evidence) *(8)*.

RECOMMENDATION C.2: Antibiotic prophylaxis is only recommended to prevent recurrent urinary tract infections in pregnant women in the context of rigorous research. *(Context-specific recommendation – research)*

Remarks

- Further research is needed to determine the best strategies for preventing recurrent urinary tract infection (RUTI) in pregnancy, including the effects of antibiotic prophylaxis on pregnancy-related outcomes and changes in antimicrobial resistance.

Acceptability

In LMICs, some women hold the belief that pregnancy is a healthy condition and may not accept the use of antibiotics in this context (particularly if they have no symptoms) unless they have experienced a previous pregnancy complication (high confidence in the evidence) *(8)*. Others view ANC as a source of knowledge, information and medical safety, and generally appreciate the interventions and advice they are offered (high confidence in the evidence). However, engagement may be limited if this type of intervention is not explained properly. In addition, where there are likely to be additional costs associated with treatment, women are less likely to engage (high confidence in the evidence).

Feasibility

A lack of resources in LMICs, both in terms of the availability of the medicines and testing, and the lack of suitably trained staff to provide relevant information and perform tests, may limit implementation (high confidence in the evidence) *(8)*.

RECOMMENDATION C.3: Antenatal prophylaxis with anti-D immunoglobulin in non-sensitized Rh-negative pregnant women at 28 and 34 weeks of gestation to prevent RhD alloimmunization is recommended only in the context of rigorous research. *(Context-specific recommendation – research)*

Remarks

- This context-specific recommendation relates to anti-D prophylaxis during pregnancy and not the practice of giving anti-D after childbirth, for which there is high-certainty evidence of its effect of reducing RhD alloimmunization in subsequent pregnancies *(57)*. Anti-D should still be given postnatally when indicated.
- Determining the prevalence of RhD alloimmunization and associated poor outcomes among women in LMIC settings, as well as developing strategies to manage this condition, is considered a research priority.

Acceptability

Anti-D immunoglobulin is derived from human plasma and is administered by injection, which may not be acceptable to all women. Qualitative evidence indicates that engagement may be limited if tests and procedures are not explained properly to women, or when women feel their beliefs, traditions and social support mechanisms are overlooked or ignored by health-care professionals (high confidence in the evidence) *(8)*.

Feasibility

In a number of LMIC settings providers feel that a lack of resources, both in terms of the availability of the medicines and the lack of suitably trained staff to provide relevant information, may limit implementation of recommended interventions (high confidence in the evidence) *(8)*. Anti-D needs refrigeration at 2–8°C, which may not be feasible in some LMIC settings.

RECOMMENDATION C.4: In endemic areas,[15] preventive anthelminthic treatment is recommended for pregnant women after the first trimester as part of worm infection reduction programmes. *(Context-specific recommendation)*

Remarks

- This recommendation is consistent with the WHO Guideline: preventive chemotherapy to control soil-transmitted helminth infections in high-risk groups (58), which states that: "Preventive chemotherapy (deworming), using single-dose albendazole (400 mg) or mebendazole (500 mg) is recommended as a public health intervention for pregnant women, after the first trimester, living in areas where both: (1) the baseline prevalence of hookworm and/or *T. trichiura* infection is 20% or more and (2) where anaemia is a severe public health problem, with prevalence of 40% or higher among pregnant women, in order to reduce the burden of hookworm and T. *trichiura* infection (conditional recommendation, moderate quality of evidence)."
- Endemic areas are areas where the prevalence of hookworm and/or whipworm infection is 20% or more. Anaemia is considered a severe public health problem when the prevalence among pregnant women is 40% or higher.
- Infected pregnant women in non-endemic areas should receive anthelminthic treatment in the second or third trimester on a case-by-case basis *(58)*. A single dose of albendazole (400 mg) or mebendazole (500 mg) should be used *(58, 59)*.
- The safety of these drugs in pregnancy has not been unequivocally established; however, the benefits are considered to outweigh the disadvantages *(59, 60)*.
- WHO recommends a treatment strategy comprising two treatments per year in high-risk settings with a prevalence of 50% for soil-transmitted helminthiasis, and once per year in areas with a 20–50% prevalence *(58)*.
- For further guidance on soil-transmitted helminth infections, refer to the WHO *Guideline: preventive chemotherapy to control soil-transmitted helminth infections in high-risk groups (58)*.

Acceptability

Affected women are often asymptomatic and may not perceive the need for treatment. Therefore, the prevalence of soil-based helminthiasis in a particular setting is likely to influence women's and providers' preferences. Studies of anthelminthic programmes among non-pregnant cohorts, e.g. schoolchildren, in endemic areas have shown high levels of acceptability *(61)*. For women receiving preventive treatment in endemic areas, worms are often visible in the stools the day after treatment, and this may reinforce the value of the intervention. However, where there are likely to be additional costs associated with treatment (high confidence in the evidence) or where the intervention is unavailable because of resource constraints (low confidence in the evidence) women may be less likely to engage with services *(8)*.

Feasibility

In a number of LMIC settings providers feel that a lack of resources, both in terms of the availability of the medicines and the lack of suitably trained staff to provide relevant information, may limit implementation of recommended interventions (high confidence in the evidence) *(8)*.

15 Greater than 20% prevalence of infection with any soil-transmitted helminths.

RECOMMENDATION C.5: Tetanus toxoid vaccination is recommended for all pregnant women, depending on previous tetanus vaccination exposure, to prevent neonatal mortality from tetanus. *(Recommended)*

Remarks

- This recommendation is consistent with recommendations from the 2006 WHO guideline on Maternal immunization against tetanus *(62)*. The GDG endorses the 2006 guideline approach, which recommends the following.
  - If a pregnant woman has not previously been vaccinated, or if her immunization status is unknown, she should receive two doses of a tetanus toxoid-containing vaccine (TT-CV) one month apart with the second dose given at least two weeks before delivery. Two doses protect against tetanus infection for 1–3 years in most people. A third dose is recommended six months after the second dose, which should extend protection to at least five years.
  - Two further doses for women who are first vaccinated against tetanus during pregnancy should be given after the third dose, in the two subsequent years or during two subsequent pregnancies.
  - If a woman has had 1–4 doses of a TT-CV in the past, she should receive one dose of a TT-CV during each subsequent pregnancy to a total of five doses (five doses protects throughout the childbearing years).
- Tetanus vaccination and clean delivery practices are major components of the strategy to eradicate maternal and neonatal tetanus globally *(63)*.
- Effective surveillance is critical for identifying areas or populations at high risk of neonatal tetanus and for monitoring the impact of interventions.
- A monitoring system should include an immunization register, personal vaccination cards and maternal health records, which should be held by the woman.
- For effective implementation, ANC health-care providers need to be trained in tetanus vaccination and the vaccine, equipment and supplies (refrigerator, needles and syringes) need to be readily available at ANC services.
- Policy-makers in low prevalence/high-income settings may choose not to include tetanus vaccination among ANC interventions if effective tetanus immunization programmes and good post-exposure prophylaxis exist outside of pregnancy.
- ANC contacts should be used to verify the vaccination status of pregnant women, and administer any vaccines that are recommended in the national immunization schedule. ANC contacts are also opportunities to explain the importance of infant vaccination and communicate the infant/child vaccination schedule to pregnant women.
- Further information can be found in the WHO guidance (62), available at: https://www.who.int/reproductivehealth/publications/maternal_perinatal_health/immunization_tetanus.pdf; and in WHO's vaccine position papers, available at: https://www.who.int/immunization/documents/positionpapers/en/.

Acceptability

Qualitative evidence indicates that most women view ANC as a source of knowledge, information and medical safety, and generally appreciate the interventions and advice they are offered. However, engagement may be limited if vaccinations are not explained properly or when women feel their beliefs, traditions and social support mechanisms are overlooked or ignored by health-care professionals (high confidence in the evidence) *(8)*. Lack of engagement may be compounded if services are delivered in a hurried, inflexible, didactic manner (high confidence in the evidence).

Feasibility

Antenatal services provide a convenient opportunity for vaccinating pregnant women, particularly in settings without effective childhood immunization programmes. Qualitative evidence indicates that if there are additional costs associated with vaccination (including transport costs and loss of earnings), uptake may be limited (high confidence in the evidence) *(8)*. In addition, ANC providers in many LMIC settings feel that a lack of resources, both in terms of the availability of vaccines and the lack of suitably trained staff, may limit implementation (high confidence in the evidence) *(8)*.

RECOMMENDATION C.6: In malaria-endemic areas in Africa, intermittent preventive treatment with sulfadoxine-pyrimethamine (IPTp-SP) is recommended for all pregnant women. Dosing should start in the second trimester, and doses should be given at least one month apart, with the objective of ensuring that at least three doses are received. *(Context-specific recommendation)*

Remarks

- This recommendation has been integrated from the WHO *Guidelines for the treatment of malaria* (2015), where it is considered to be a strong recommendation based on high-quality evidence *(17)*.
- Malaria infection during pregnancy is a major public health problem, with substantial risks for the mother, her fetus and the newborn. WHO recommends a package of interventions for preventing and controlling malaria during pregnancy, which includes promotion and use of insecticide-treated nets, appropriate case management with prompt, effective treatment, and, in areas with moderate to high transmission of *Plasmodium falciparum*, administration of IPTp-SP *(17)*.
- The high-quality evidence supporting this recommendation was derived from a systematic review of seven randomized controlled trial (RCTs) conducted in malaria-endemic countries, which shows that three or more doses of sulfadoxine-pyrimethamine (SP) is associated with reduced maternal parasitaemia, fewer low-birth weight infants and increased mean birth weight compared with two doses only *(64)*.
- The malaria GDG noted that most evidence was derived from women in their first and second pregnancies; however, the limited evidence on IPTp-SP from women in their third and subsequent pregnancies was consistent with benefit *(17)*.
- To ensure that pregnant women in endemic areas start IPTp-SP as early as possible in the second trimester, policy-makers should ensure health system contact with women at 13 weeks of gestation. Policy-makers could also consider supplying women with their first SP dose at the first ANC visit with instructions about the date (corresponding to 13 weeks of gestation) on which the medicine should be taken.
- SP acts by interfering with folic acid synthesis in the malaria parasite, thereby inhibiting its life-cycle. There is some evidence that high doses of supplemented folic acid (i.e. 5 mg daily or more) may interfere with the efficacy of SP in pregnancy *(65)*. Countries should ensure that they procure and distribute folic acid supplements for antenatal use at the recommended antenatal dosage (i.e. 0.4 mg daily).
- The malaria GDG noted that there is insufficient evidence on the safety, efficacy and pharmacokinetics of most antimalarial agents in pregnancy, particularly during the first trimester *(17)*.
- Detailed evidence and guidance related to the recommendation can be found in the 2015 guidelines *(17)*, available at: https://www.who.int/malaria/publications/atoz/9789241549127/en/.

RECOMMENDATION C.7: Oral pre-exposure prophylaxis (PrEP) containing tenofovir disoproxil fumarate (TDF) should be offered as an additional prevention choice for pregnant women at substantial risk of HIV infection as part of combination prevention approaches. *(Context-specific recommendation)*

Remarks

- This recommendation has been integrated from the WHO guideline on when to start antiretroviral therapy and on pre-exposure prophylaxis for HIV (2015), where it is considered to be a strong recommendation based on high-quality evidence *(44)*. The evidence and further guidance related to the recommendation can be found in this guideline.
- "Substantial risk" is provisionally defined as HIV incidence greater than 3 per 100 person-years in the absence of PrEP, but individual risk varies within this group depending on individual behaviour and the characteristics of sexual partners. Local epidemiological evidence concerning risk factors and HIV incidence should be used to inform implementation.
- Thresholds for offering PrEP may vary depending on a variety of considerations, including resources, feasibility and demand.
- The level of protection is strongly correlated with adherence.
- Detailed evidence and guidance related to this recommendation can be found in the 2015 guideline *(44)*, available at: https://www.who.int/hiv/pub/guidelines/earlyrelease-arv/en/.

D. Interventions for common physiological symptoms

RECOMMENDATION D.1: Ginger, chamomile, vitamin B6 and/or acupuncture are recommended for the relief of nausea in early pregnancy, based on a woman's preferences and available options. *(Recommended)*

Remarks

- In the absence of stronger evidence, the GDG agreed that these non-pharmacological options are unlikely to have harmful effects on mother and baby.
- Women should be informed that symptoms of nausea and vomiting usually resolve in the second half of pregnancy.
- Pharmacological treatments for nausea and vomiting, such as doxylamine and metoclopramide, should be reserved for those pregnant women experiencing distressing symptoms that are not relieved by non-pharmacological options, under the supervision of a medical doctor.

Acceptability

Qualitative evidence from a range of LMICs suggests that women may be more likely to turn to traditional healers, herbal remedies or traditional birth attendants (TBAs) to treat these symptoms (moderate confidence in the evidence) *(8)*. In addition, evidence from a diverse range of settings indicates that while women generally appreciate the interventions and information provided during antenatal visits, they are less likely to engage with services if their beliefs, traditions and socioeconomic circumstances are ignored or overlooked by health-care providers and/or policy-makers (high confidence in the evidence). This may be particularly pertinent for an interventions like acupuncture or acupressure, which may be culturally alien and/or poorly understood in certain contexts.

Feasibility

A lack of suitably trained staff may limit feasibility of certain interventions (high confidence in the evidence) *(8)*.

RECOMMENDATION D.2: Advice on diet and lifestyle is recommended to prevent and relieve heartburn in pregnancy. Antacid preparations can be offered to women with troublesome symptoms that are not relieved by lifestyle modification. *(Recommended)*

Remarks

- Lifestyle advice to prevent and relieve symptoms of heartburn includes avoidance of large, fatty meals and alcohol, cessation of smoking, and raising the head of the bed to sleep.
- The GDG agreed that antacids, such as magnesium carbonate and aluminium hydroxide preparations, are probably unlikely to cause harm in recommended dosages.
- There is no evidence that preparations containing more than one antacid are better than simpler preparations.
- Antacids may impair absorption of other drugs *(66)*, and therefore should not be taken within two hours of iron and folic acid supplements.

Acceptability

See ACCEPTABILITY section for D.1.

Additionally, indirect evidence also indicates that women welcome the pregnancy-related advice and guidance given by health-care professionals during antenatal visits, so may respond to lifestyle suggestions favourably (moderate confidence in the evidence).

Feasibility

Qualitative evidence suggests that a lack of resources may limit the offer of treatment for this condition (high confidence in the evidence) *(8)*. In addition, where there are additional costs for pregnant women associated with treatment, women are less likely to use it.

RECOMMENDATION D.3: Magnesium, calcium or non-pharmacological treatment options can be used for the relief of leg cramps in pregnancy, based on a woman's preferences and available options. *(Recommended)*

Remarks

- The review found no evidence on the effect of non-pharmacological therapies, such as muscle stretching, relaxation, heat therapy, dorsiflexion of the foot and massage.
- The evidence on magnesium and calcium is generally of low certainty. However, the GDG agreed that they are unlikely to be harmful in the dose schedules evaluated in included studies.
- Further research into the etiology and prevalence of leg cramps in pregnancy, and the role (if any) of magnesium and calcium in symptom relief, is needed.

Acceptability

Qualitative evidence from a diverse range of settings suggests that women generally appreciate the pregnancy-related advice given by healthcare professionals during ANC, so may respond to supplement suggestions favourably (moderate confidence in the evidence) *(8)*. Evidence from some LMICs suggests that women hold the belief that pregnancy is a healthy condition and may turn to traditional healers and/or herbal remedies to treat these kinds of associated symptoms (high confidence in the evidence).

Feasibility

See FEASIBILITY section for D.2.

RECOMMENDATION D.4: Regular exercise throughout pregnancy is recommended to prevent low back and pelvic pain. There are a number of different treatment options that can be used, such as physiotherapy, support belts and acupuncture, based on a woman's preferences and available options. *(Recommended)*

Remarks

- Exercise to prevent low back and pelvic pain in pregnancy can take place on land or in water. While exercise may also be helpful to relieve low back pain, it could exacerbate pelvic pain associated with symphysis pubis dysfunction and is not recommended for this condition.
- Regular exercise is a key component of lifestyle interventions, which are recommended for pregnant women as part of ANC to prevent excessive weight gain in pregnancy (see Recommendation A.9).
- Pregnant women with low back and/or pelvic pain should be informed that symptoms usually improve in the months after birth.
- Women should be informed that it is unclear whether there are side-effects to alternative treatment options due to a paucity of data.
- Standardized reporting of outcomes is needed for future research on treatment for low back and/or pelvic pain in pregnancy.

Acceptability

Qualitative evidence from a diverse range of settings indicates that while women generally appreciate the interventions and information provided during antenatal visits, they are less likely to engage with services if their beliefs, traditions and socioeconomic circumstances are ignored or overlooked by healthcare providers and/or policy-makers (high confidence in the evidence) *(8)*. This may be particularly pertinent for an intervention like acupuncture, which may be culturally alien and/or poorly understood in certain contexts. In addition, where there are likely to be additional costs associated with treatment or where the treatment may be unavailable (because of resource constraints), women are less likely to engage with health services (high confidence in the evidence).

Feasibility

A lack of resources may limit the offer of treatment for this condition (high confidence in the evidence) *(8)*.

RECOMMENDATION D.5: Wheat bran or other fibre supplements can be used to relieve constipation in pregnancy if the condition fails to respond to dietary modification, based on a woman's preferences and available options. *(Recommended)*

Remarks

- Dietary advice to reduce constipation during pregnancy should include promoting adequate intake of water and dietary fibre (found in vegetables, nuts, fruit and whole grains).
- For women with troublesome constipation that is not relieved by dietary modification or fibre supplementation, stakeholders may wish to consider intermittent use of poorly absorbed laxatives.

Acceptability

See ACCEPTABILITY section for D.1.

Also, evidence from a diverse range of settings indicates that while women generally appreciate the interventions and information provided during antenatal visits, they are less likely to engage with services if their beliefs, traditions and socioeconomic circumstances are ignored or overlooked by healthcare providers and/or policy-makers (high confidence in the evidence).

Feasibility

Other qualitative evidence suggests that a lack of resources may limit the offer of treatment for constipation (high confidence in the evidence) *(8)*.

RECOMMENDATION D.6: Non-pharmacological options, such as compression stockings, leg elevation and water immersion, can be used for the management of varicose veins and oedema in pregnancy based on a woman's preferences and available options. *(Recommended)*

Remarks

- Women should be informed that symptoms associated with varicose veins may worsen as pregnancy progresses but that most women will experience some improvement within a few months of giving birth.
- Rest, leg elevation and water immersion are low-cost interventions that are unlikely to be harmful.

Acceptability

See ACCEPTABILITY section for D.1.

Also, qualitative evidence shows that, where there are likely to be additional costs associated with treatment or where the treatment may be unavailable (because of resource constraints), women are less likely to engage with health services (high confidence in the evidence).

Feasibility

The evidence also suggests that a lack of resources may limit the offer of treatment for varicose veins and oedema (high confidence in the evidence) *(8)*.

E. Health systems interventions to improve the utilization and quality of ANC

RECOMMENDATION E.1: It is recommended that each pregnant woman carries her own case notes during pregnancy to improve continuity, quality of care and her pregnancy experience. *(Recommended)*

Remarks

- The GDG noted that women-held case notes are widely used and are often the only medical records available in various LMIC settings.
- The GDG agreed that the benefits of women-held case notes outweigh the disadvantages. However, careful consideration should be given as to what personal information it is necessary to include in the case notes, to avoid stigma and discrimination in certain settings. In addition, health-system planners should ensure that admission to hospitals or other health-care facilities do not depend on women presenting their case notes.
- Health-system planners should consider which form the women-held case notes should take (electronic or paper-based), whether whole sets of case notes will be held by women or only specific parts of them, and how copies will be kept by health-care facilities.
- For paper-based systems, health-system planners also need to ensure that case notes are durable and transportable. Health systems that give women access to their case notes through electronic systems need to ensure that all pregnant women have access to the appropriate technology and that attention is paid to data security.
- Health-system planners should ensure that the contents of the case notes are accessible to all pregnant women through the use of appropriate, local languages and appropriate reading levels.

Acceptability

Qualitative evidence suggests that women from a variety of settings are likely to favour carrying their case notes because of the increased opportunity to acquire pregnancy and health-related information and the associated sense of empowerment this brings (high confidence in the evidence) *(8)*. There may be potential for abuse of the system in some LMIC settings, for example, by limiting access to hospitals for women who do not have case notes, particularly where maternity services are under-resourced (moderate confidence in the evidence). Further evidence from a mixed-methods review supports RCT evidence that women feel more satisfied when they carry, or have access to, their own case notes *(67)*. These review findings were not subject to GRADE-Confidence in Evidence from Reviews of Qualitative research (CERQual)* assessments, and were derived primarily from high-income settings (36 out of 37 studies). Findings also suggest that providers are generally happy for women to carry their own case notes, but feel the implementation of the approach may generate additional administrative responsibilities. Providers also raised concerns about data security, sensitivity of the shared information, and the potential for data to be lost because of fragmented systems.

Feasibility

There may be prohibitive additional costs associated with using an electronic system (USB memory sticks, software packages, etc.) in some LMIC settings (high confidence in the evidence), although paper-based records may require little in the way of extra cost or resources *(8)*.

* The GRADE-CERQual approach was developed by the GRADE (Grading of Recommendations Assessment, Development and Evaluation) Working Group, and provides a transparent method for assessing and assigning the level of confidence that can be placed in evidence from reviews of qualitative research.

RECOMMENDATION E.2: Midwife-led continuity-of-care (MLCC) models, in which a known midwife or small group of known midwives supports a woman throughout the antenatal, intrapartum and postnatal continuum, are recommended for pregnant women in settings with well functioning midwifery programmes. *(Context-specific recommendation)*

Remarks

- MLCC models are models of care in which a known and trusted midwife (caseload midwifery), or small group of known midwives (team midwifery), supports a woman throughout the antenatal, intrapartum and postnatal period, to facilitate a healthy pregnancy and childbirth, and healthy parenting practices.
- MLCC models are complex interventions and it is unclear whether the pathway of influence producing these positive effects is the continuity of care, the midwifery philosophy of care or both. The midwifery philosophy inherent in MLCC models may or may not be enacted in standard midwife practice in other models of care.
- Policy-makers in settings without well functioning midwife programmes should consider implementing this model only after successfully scaling up of the number and quality of practising midwives. In addition, stakeholders may wish to consider ways of providing continuous care through other care providers, because women value continuity of care.
- The panel noted that with this model of care it is important to monitor resource use, and provider burnout and workload, to determine whether caseload or team care models are more sustainable in individual settings.
- MLCC requires that well-trained midwives are available in sufficient numbers for each woman to see one or only a small group of midwives throughout pregnancy and during childbirth. This model may therefore require a shift in resources to ensure that the health system has access to a sufficient number of midwives with reasonable caseloads.
- The introduction of MLCC may lead to a shift in the roles and responsibilities of midwives as well as other health-care professionals who have previously been responsible for antenatal and postnatal care. Where this is the case, implementation is likely to be more effective if all relevant stakeholders are consulted and human resources departments are involved. In some settings, government-level consultation with professional organizations could also aid implementation processes.
- The need for additional one-off or continuing training and education should be assessed, and should be provided where necessary.

Acceptability

Qualitative evidence synthesized from a wide variety of settings and contexts indicates that women welcome the opportunity to build supportive, caring relationships with a midwife or a small number of midwives during the maternity phase (high confidence in the evidence) and appreciate a consistent, unhurried, woman-centred approach during ANC visits (high confidence in the evidence) *(8)*. Evidence from providers, mainly in HICs, indicates that they view MLCC as a way of achieving the authentic, supportive relationships that women desire (moderate confidence in the evidence). There is very little evidence on MLCC models from LMICs. However, indirect evidence from providers in these locations suggests that they would welcome the opportunity to use an MLCC model but feel they do not have the resources to do so (low confidence in the evidence).

Feasibility

Qualitative evidence from high-, medium- and low-resource settings highlights concerns among providers about potential staffing issues, e.g. for the delivery of caseload or one-to-one approaches (high confidence in the evidence) *(8)*.

RECOMMENDATION E.3: Group antenatal care provided by qualified health-care professionals may be offered as an alternative to individual antenatal care for pregnant women in the context of rigorous research, depending on a woman's preferences and provided that the infrastructure and resources for delivery of group antenatal care are available. *(Context-specific recommendation – research)*

Remarks

- With the group ANC model, the first visit for all pregnant women is an individual visit. Then at subsequent visits, the usual individual pregnancy health assessment, held in a private examination area, is integrated into a group ANC session, with facilitated educational activities and peer support.
- Health-care facilities need to be seeing sufficient numbers of pregnant women, as allocation to groups is ideally performed according to gestational age.
- Health-care providers need to have appropriate facilities to deal with group sessions, including access to large, well ventilated rooms or sheltered spaces with adequate seating. A private space should be available for examinations, and opportunities should be given for private conversations.
- Group ANC may take longer than individual ANC, and this may pose practical problems for some women in terms of work and childcare. Health-care providers should be able to offer a variety of time slots for group sessions (morning, afternoon, evening) and should consider making individual care available as well.
- The GDG noted that group ANC may have acceptability and feasibility issues in settings where perceived differences keep people apart, e.g. women from different castes in India may not wish to be in a group together.
- Group ANC studies are under way in Nepal, Uganda and five other low-income countries, and the GDG was informed by a GDG member that some of these studies are due to report soon. Core outcomes of studies of group ANC should include maternal and perinatal health outcomes, coverage, and women's and providers' experiences.

Acceptability

Qualitative evidence from several HICs suggests that women enjoy the group format and use the opportunity to build socially supportive relationships with other pregnant women and health-care professionals (high confidence in the evidence) *(8)*. The flexibility of the format allows women to exchange valuable information with each other and discuss pregnancy-related concerns in a relaxed and informal manner (high confidence in the evidence). Most women appreciate the additional time inherent in the group approach (high confidence in the evidence), although some women do not attend group sessions because of the additional time commitments (moderate confidence in the evidence). Some women have reservations about the lack of privacy during the group sessions, particularly during physical examinations (low confidence in the evidence) and the desire to have partners/husbands included varies (moderate confidence in the evidence). Evidence from providers in HICs suggests they find group sessions to be enjoyable and satisfying and a more efficient use of their time (moderate confidence in the evidence) *(8)*. Providers also identified the group approach as a way of providing continuity of care (moderate confidence in the evidence).

Feasibility

Qualitative evidence from high-resource settings suggests that health-care professionals view the facilitative components of group ANC as a skill requiring additional investment in terms of training and provider commitment (moderate confidence in the evidence) *(8)*. Some providers also feel that clinics need to be better equipped to deliver group sessions, i.e. clinics need to have large enough rooms with adequate seating (moderate confidence in the evidence). The feasibility of group ANC in low-resource settings needs further research; however, pilot studies in Ghana, Malawi and the United Republic of Tanzania suggest that group ANC is feasible in these settings *(68)*. It has been suggested that group ANC may be a feasible way of improving ANC quality in settings where relatively few providers attend to relatively large numbers of women in a limited time and, as such, effective communication can be challenging *(69)*. Others have suggested that the group approach may be a sustainable way of providing continuity of care *(68)*.

RECOMMENDATION E.4.1: The implementation of community mobilization through facilitated participatory learning and action (PLA) cycles with women's groups is recommended to improve maternal and newborn health, particularly in rural settings with low access to health services. Participatory women's groups represent an opportunity for women to discuss their needs during pregnancy, including barriers to reaching care, and to increase support to pregnant women.
(Context-specific recommendation)

Remarks

- Part of this recommendation was integrated from WHO recommendations on community mobilization through facilitated participatory learning and action cycles with women's groups for maternal and newborn health (2014) *(70)*.
- The pathways of influence of this multifaceted, context-specific intervention on maternal and newborn outcomes are difficult to assess. Women meeting to identify their needs and seek solutions plays an important role; mechanisms related to additional activities that are organized based on the solutions identified at the meetings may also play a role.
- Detailed information and guidance related to the recommendation, including important implementation considerations, can be found in the 2014 WHO recommendations on PLA cycles *(70)*, available at: https://www.who.int/maternal_child_adolescent/documents/community-mobilization-maternal-newborn/en/.

Acceptability

Qualitative evidence suggests that women in a variety of settings and contexts readily engage with interventions designed to increase communication and support, provided they are delivered in a caring and respectful manner (high confidence in the evidence) *(8)*. The use of women's groups is likely to fulfil two key requirements of ANC from a woman's perspective – the opportunity to receive and share relevant information and the opportunity to develop supportive relationships with other women and health-care providers (high confidence in the evidence). Evidence from women and providers in LMICs also highlighted the importance of active community engagement in the design and delivery of informational-based services, especially in communities where traditional beliefs may differ from conventional understandings (moderate confidence in the evidence). Qualitative evidence from providers suggests that there is a willingness to supply pregnancy-related information and offer psychological/emotional support to women provided that resources are available (high confidence in the evidence) and the services are delivered in a coordinated, organized manner with appropriate managerial support (moderate confidence in the evidence) *(8)*.

Feasibility

Qualitative evidence suggests that, where health-care providers are involved in facilitating women's groups, they may need additional training to help with the facilitative components and this may be a barrier in some resource-poor settings (high confidence in the evidence). Similarly, the extra costs associated with home visits in terms of additional staff and extra resources may limit implementation in some LMICs (high confidence in the evidence) *(8)*. It has been suggested that community-based interventions introduced through existing public sector health workers and local health systems may be more feasible and more likely to succeed than project-based interventions *(71)*.

RECOMMENDATION E.4.2: Packages of interventions that include household and community mobilization and antenatal home visits are recommended to improve antenatal care utilization and perinatal health outcomes, particularly in rural settings with low access to health services.
(Context-specific recommendation)

Remarks

- The GDG agreed that the extent to which these packages improve communication and support for pregnant women is not clear.
- As a stand-alone intervention, the evidence does not support the use of antenatal home visits by lay health workers during pregnancy to improve ANC utilization health outcomes. While the quality and effectiveness of communication during home visits, and the extent to which they increase support for women, is not clear, antenatal home visits may be helpful in ensuring continuity of care across the antenatal, intrapartum and postnatal periods and in promoting other healthy behaviour.
- Stakeholders need to be clear that antenatal home visits by lay health workers do not replace ANC visits.
- Stakeholders should implement health system strengthening interventions alongside these community-based interventions.
- Health-care providers need initial and ongoing training in communication with women and their partners. For women's groups and community mobilization, providers also need training on group facilitation, in the convening of public meetings and in other methods of communication.
- Information for women and community members should be provided in languages and formats accessible to them and programme planners need to ensure that health-care providers/facilitators have reliable supplies of appropriate information materials.
- Programme planners should be aware of the potential for additional costs associated with home visits and community mobilization initiatives, including the potential need for extra staff and travel expenses.
- When considering the use of antenatal home visits, women's groups, partner involvement or community mobilization, programme planners need to ensure that these can be implemented in a way that respects and facilitates women's needs for privacy as well as their choices and their autonomy in decision-making. By offering pregnant women a range of opportunities for contact, communication and support, their individual preferences and circumstances should also be addressed.
- Further research is needed on the acceptability and feasibility of mixed-gender communication, the optimal methods for community mobilization, the best model for integration with health systems, continuity elements of home visits, and the mechanisms of effect of these interventions.

Acceptability

See ACCEPTABILITY section for E.4.1.

Feasibility

See FEASIBILITY section for E.4.1.

RECOMMENDATION E.5.1: Task shifting the promotion of health-related behaviours for maternal and newborn health[16] to a broad range of cadres, including lay health workers, auxiliary nurses, nurses, midwives and doctors is recommended. *(Recommended)*

RECOMMENDATION E.5.2: Task shifting the distribution of recommended nutritional supplements and intermittent preventive treatment in pregnancy (IPTp) for malaria prevention to a broad range of cadres, including auxiliary nurses, nurses, midwives and doctors is recommended. *(Recommended)*

Remarks

- Recommendations E.5.1 and E.5.2 have been adapted and integrated from *Optimizing health worker roles to improve access to key maternal and newborn health interventions through task shifting* (OptimizeMNH) (2012) *(72)*.
- The GDG noted that, while task shifting has an important role to play in allowing flexibility in health-care delivery in low-resource settings, policy-makers need to work towards midwife-led care for all women.
- Lay health workers need to be recognized and integrated into the system, and not be working alone, i.e. task shifting needs to occur within a team approach.
- The mandate of all health workers involved in task shifting programmes needs to be clear.
- In a separate guideline on HIV testing services *(43)*, WHO recommends that lay providers who are trained and supervised can independently conduct safe and effective HIV testing using rapid tests (see Recommendation B.1.8).
- The GDG noted that it may be feasible to task shift antenatal ultrasound to midwives with the appropriate training, staffing, mentoring and referral systems in place.
- Further research is needed on the mechanism of effect of MLCC and whether continuity of care can be task shifted.
- Further information on this recommendation can be found in the OptimizeMNH guideline *(72)*, available at: http://www.who.int/reproductivehealth/publications/maternal_perinatal_health/978924504843/en/.

16 Including promotion of the following: care-seeking behaviour and ANC utilization; birth preparedness and complication readiness; sleeping under insecticide-treated bednets; skilled care for childbirth; companionship in labour and childbirth; nutritional advice; nutritional supplements; HIV testing during pregnancy; exclusive breastfeeding; postnatal care and family planning; immunization according to national guidelines.

RECOMMENDATION E.6: Policy-makers should consider educational, regulatory, financial, and personal and professional support interventions to recruit and retain qualified health workers in rural and remote areas. *(Context-specific recommendation)*

Remarks

- Recommendation E.6 has been adapted and integrated for the ANC guideline from the 2010 WHO publication *Increasing access to health workers in remote and rural areas through improved retention: global policy recommendations* (19).
- Strong recommendations (abridged) on recruitment and staff retention from the above guideline include the following.
  - Use targeted admission policies to enrol students with a rural background in education programmes for various health disciplines and/or establish a health-care professional school outside of major cities.
  - Revise undergraduate and postgraduate curricula to include rural health topics and clinical rotations in rural areas so as to enhance the competencies of health-care professionals working in rural areas.
  - Improve living conditions for health workers and their families and invest in infrastructure and services (sanitation, electricity, telecommunications, schools, etc.).
  - Provide a good and safe working environment, including appropriate equipment and supplies, supportive supervision and mentoring.
  - Identify and implement appropriate outreach activities to facilitate cooperation between health workers from better-served areas and those in underserved areas, and, where feasible, use tele-health to provide additional support.
  - Develop and support career development programmes and provide senior posts in rural areas so that health workers can move up the career path as a result of experience, education and training, without necessarily leaving rural areas.
  - Support the development of professional networks, rural health-care professional associations, rural health journals, etc., to improve the morale and status of rural providers and reduce feelings of professional isolation.
  - Adopt public recognition measures such as rural health days, awards and titles at local, national and international levels to lift the profile of working in rural areas.
- Conditional educational, regulatory and financial recommendations from this guideline can be found in the WHO global policy recommendations document *(19)*, available at: http://www.who.int/hrh/retention/guidelines/en/

RECOMMENDATION E.7: Antenatal care models with a minimum of eight contacts are recommended to reduce perinatal mortality and improve women's experience of care. *(Recommended)*

Remarks

- The GDG stresses that the four-visit focused ANC (FANC) model does not offer women adequate contact with health-care practitioners and is no longer recommended. With the FANC model, the first ANC visit occurs before 12 weeks of pregnancy, the second around 26 weeks, the third around 32 weeks, and the fourth between 36 and 38 weeks of gestation. Thereafter, women are advised to return to ANC at 41 weeks of gestation or sooner if they experience danger signs. Each ANC visit involves specific goals aimed at improving triage and timely referral of high-risk women and includes educational components *(10)*. However, up-to-date evidence shows that the FANC model, which was developed in the 1990s, is probably associated with more perinatal deaths than models that comprise at least eight ANC visits. Furthermore, evidence suggests that more ANC visits, irrespective of the resource setting, is probably associated with greater maternal satisfaction than less ANC visits.
- The GDG prefers the word "contact" to "visit", as it implies an active connection between a pregnant woman and a health-care provider that is not implicit with the word "visit". In terms of the operationalization of this recommendation, "contact" can be adapted to local contexts through community outreach programmes and lay health worker involvement.
- The decision regarding the number of contacts with a health system was also influenced by the following:
  - evidence supporting improving safety during pregnancy through increased frequency of maternal and fetal assessment to detect problems
  - evidence supporting improving health system communication and support around pregnancy for women and families
  - evidence from HIC studies indicating no important differences in maternal and perinatal health outcomes between ANC models that included at least eight contacts and ANC models that included more (11–15) contacts *(73)*
  - evidence indicating that more contact between pregnant women and knowledgeable, supportive and respectful health-care practitioners is more likely to lead to a positive pregnancy experience.
- Implementation considerations related to this recommendation and the mapping of guideline recommendations to ANC contacts are presented in the WHO ANC guideline – Chapter 4: Implementation of the ANC guideline and recommendations.

Acceptability

Evidence from high-, medium- and low-resource settings suggests that women do not like reduced visit schedules and would prefer more contact with antenatal services (moderate confidence in the evidence) *(8)*. Women value the opportunity to build supportive relationships during their pregnancy (high confidence in the evidence) and for some women, especially in LMIC settings, the reduced visit schedule may limit their ability to develop these relationships, both with health-care professionals and with other pregnant women (low confidence in the evidence). In some low-income settings where women rely on husbands or partners to financially support their antenatal visits, the reduced visit schedule limits their ability to procure additional finance (low confidence in the evidence). However, the reduced visit schedule may be appreciated by some women in a range of LMIC settings because of the potential for cost savings, e.g. loss of domestic income from extra clinic attendance and/or associated travel costs (low confidence in the evidence). Indirect evidence also suggests that women are much more likely to engage with antenatal services if care is provided by knowledgeable, kind health-care professionals who have the time and resources to deliver genuine woman-centred care, regardless of the number of visits (high confidence in the evidence). Specific evidence from providers relating to reduced visit schedules or the adoption of FANC is sparse and, in some LMICs, highlights concerns around the availability of equipment and resources, staff shortages and inadequate training – issues that are pertinent to all models of ANC delivery in low-resource settings.

Feasibility

Qualitative evidence suggests that some providers in LMICs feel that the reduced visit schedule is a more efficient use of staff time and is less likely to deplete limited supplies of equipment and medicine (moderate confidence in the evidence) *(8)*. Programme reports from Ghana and Kenya stress that inadequate equipment, supplies, infrastructure and training may hamper implementation *(74, 75)*. Providers have also raised concerns about the difficulty of incorporating all of the FANC components into relatively short appointments, especially in LMICs (Burkina Faso, Uganda and the United Republic of Tanzania) where services are already stretched *(76, 77)*.

7. The 2016 WHO ANC model for a positive pregnancy experience: recommendations mapped to eight scheduled ANC contacts

The table below from the WHO recommendations *(1)* (page 108) describes which activities should take place at each contact. These are to be modified in accordance with the country-specific package of ANC interventions. Please note that actions recommended only in a research context should not be included unless there are plans and funds available for that research.

Type of intervention	Type of recommendation	Eight scheduled ANC contacts (weeks of gestation)							
		**1** (12 weeks)	**2** (20 weeks)	**3** (26 weeks)	**4** (30 weeks)	**5** (34 weeks)	**6** (36 weeks)	**7** (38 weeks)	**8** (40 weeks)
**A. Nutritional interventions**									
A.1.1. Nutritional counselling on a healthy diet and physical activity	Recommended	×	×	×	×	×	×	×	×
A.1.2. Nutritional education on increasing daily energy and protein intake	Context-specific recommendation	×	×	×	×	×	×	×	×
A.1.3. Balanced energy and protein supplementation in undernourished populations	Context-specific recommendation	×	×	×	×	×	×	×	×
A.2.1. Daily iron and folic acid supplements	Recommended	×	×	×	×	×	×	×	×
A.2.2. Intermittent iron and folic acid supplements	Context-specific recommendation	×	×	×	×	×	×	×	×
A.3. Calcium supplements	Context-specific recommendation	×	×	×	×	×	×	×	×

Type of intervention	Type of recommendation	Eight scheduled ANC contacts (weeks of gestation)							
		1 (12 weeks)	2 (20 weeks)	3 (26 weeks)	4 (30 weeks)	5 (34 weeks)	6 (36 weeks)	7 (38 weeks)	8 (40 weeks)
A.4. Vitamin A supplements	Context-specific recommendation	×	×	×	×	×	×	×	×
A.5. Zinc supplements	Context-specific recommendation (research)								
A.10. Restricting caffeine intake	Context-specific recommendation	×	×	×	×	×	×	×	×
Essential good clinical practices and other essential counselling (see BAT Recommendations data worksheet for specific activities – rows 50–63)		×	×	×	×	×	×	×	×
**B. Maternal and fetal assessment**									
B.1.1. Anaemia	Context-specific recommendation	×		×			×		
B.1.2. Asymptomatic bacteriuria (ASB)	Context-specific recommendation	×		×		×			
B.1.3. Intimate partner violence (IPV)	Context-specific recommendation	×	×	×	×	×	×	×	×
B.1.4. Gestational diabetes mellitus (GDM)	Recommended	×	×	×	×	×	×	×	×
B.1.5. Tobacco use	Recommended	×	×	×	×	×	×	×	×
B.1.6. Substance use	Recommended	×	×	×	×	×	×	×	×
B.1.7. HIV and syphilis	Recommended	×							
B.1.8. Tuberculosis (TB)	Context-specific recommendation	×							

Type of intervention	Type of recommendation	Eight scheduled ANC contacts (weeks of gestation)							
		**1** (12 weeks)	**2** (20 weeks)	**3** (26 weeks)	**4** (30 weeks)	**5** (34 weeks)	**6** (36 weeks)	**7** (38 weeks)	**8** (40 weeks)
B.2.1. Daily fetal movement counting	Context-specific recommendation (research)								
B.2.2. Symphysis-fundal height (SFH) measurement or abdominal palpation	Context-specific recommendation		×	×	×	×	×	×	×
B.2.4. Ultrasound scan	Recommended	×	×						
**C. Preventive measures**									
C.1. Antibiotics for asymptomatic bacteriuria (ASB)	Recommended	×		×		×			
C.2. Antibiotic prophylaxis to prevent recurrent urinary tract infections	Context-specific recommendation (research)								
C.3. Antenatal anti-D immunoglobulin administration	Context-specific recommendation (research)								
C.4. Preventive anthelminthic treatment	Context-specific recommendation		×						
C.5. Tetanus toxoid vaccination	Recommended	×							
C.6. Malaria prevention: intermittent preventive treatment in pregnancy (IPTp)	Context-specific recommendation	× (13 weeks)	×	×	×		×		×
C.7. Pre-exposure prophylaxis for HIV prevention	Context-specific recommendation	×							

Type of intervention	Type of recommendation	Eight scheduled ANC contacts (weeks of gestation)							
		**1** (12 weeks)	**2** (20 weeks)	**3** (26 weeks)	**4** (30 weeks)	**5** (34 weeks)	**6** (36 weeks)	**7** (38 weeks)	**8** (40 weeks)
**D. Interventions for common physiological symptoms**									
D.1. Nausea and vomiting	Recommended	×	×	×					
D.2–6. Heartburn, leg cramps, low back and pelvic pain, constipation, varicose veins and oedema	Recommended	×	×	×	×	×	×	×	×

8. Draft agenda for stakeholders' meeting

This section aims to help MoHs organize and carry out a stakeholders' meeting. The following draft agenda proposes a possible structure for the first country stakeholders' meeting to review the situational analysis results summary (baseline assessment tool [BAT] worksheet 2) as well as the country-specific package of ANC interventions (BAT worksheet 4). Countries should tailor the agenda to their needs and setting.

National stakeholders' meeting on ANC guideline implementation in Country X
City, Country; Date (DD.MM.YYYY)

Purpose
- To standardize the adaptation of the ANC model to deliver quality pregnancy care in Country X based on the 2016 WHO ANC guideline.

Specific objectives:
1. To share the WHO recommendations on ANC
2. To discuss the preliminary findings of the ANC situational analysis
3. To develop a specific ANC model to deliver quality pregnancy care in Country X
4. To develop a country roadmap for the adoption and implementation of the proposed ANC model.

Expected outcomes:
1. Country ANC situational analysis completed
2. Country ANC model to deliver quality pregnancy care developed
3. Country roadmap for dialogue, adoption and implementation of ANC delivery model developed.

Day 1: Weekday, DD.MM.YYYY
Chair:

Time	Activity	Responsible person
08:30 – 09:00	Registration of participants	
09:00 – 09:15	Welcome and introduction of participants<br>Official opening:<br>Welcome remarks and objectives of the meeting<br>Remarks: WHO and MoH	
09:15 – 09:30	Overview of the meeting objectives and expected outputs<br>Overview of the agenda	
09:30 – 10:30	Women's voices on ANC services*<br>Overview of the 2016 WHO ANC guideline<br>Discussion	
10:30 – 11:00	Coffee break	
11:00 – 12:30	Presentation of the preliminary findings of the ANC situational analysis in Country X.<br>Discussion	
12:30 – 13:30	Lunch	
13:30 – 13:50	Introduction of group work	
13:50 – 15:30	Group work	All
15:30 – 16:00	Coffee break	
16:00 – 17:30	Continue group work	All

National stakeholders' meeting on ANC guideline implementation in Country X<br>City, Country; Date (DD.MM.YYYY)		
**Day 2: Weekday, DD.MM.YYYY**<br>**Chair:**		
**Time**	**Activity**	**Responsible person**
09:00 – 10:30	Group work	All
10:30 – 11:00	Coffee break	
11:00 – 12:00	Group work	All
12:00 – 13:00	Report back from group work:<br>Service delivery model for WHO ANC guideline<br>ANC innovations<br>Strategies for how to implement the ANC model	Facilitators
13:00 – 14:00	Lunch break	
14:00 – 15:00	Plenary discussion on key steps for implementing the WHO ANC guideline	Facilitators
15:00 – 15:30	Summary of strategic elements from the discussion to inform the country roadmap for ANC guideline implementation in Country X	Facilitators
15:30 – 16:00	Discuss selection criteria for implementation region	MoH
16:00 – 16:15	Closing remarks	MoH
16:15 – 16:30	Coffee break	

* The QES Slidedoc® should be presented here at the beginning of the meeting. While the original Slidedoc® contains 55 slides/pages for reading, it can be reduced for presentation purposes.

9. Draft group-work materials for stakeholders' meeting

This section aims to help MoHs organize and carry out the stakeholders' meeting. These materials are meant to be employed during the group-work sessions shown in the draft stakeholder agenda (section 8 of this instruction manual).

The tasks to be completed during the group-work sessions include for each group to:

1. review the integrated country-specific ANC package of interventions (BAT worksheet 4); and
2. provide inputs, comments and suggestions on the following:
   - **WHAT?** The detailed activities planned for each ANC activity to take place during each contact,
   - **BY WHOM?** The cadre who will perform the activity (community health worker, midwife, etc.),
   - **WHERE?** At what health-care delivery/facility level (health centre, etc.), and
   - **WHEN?** The phasing plans for incorporating the activity in the medium to long term and how that will be done, for example with ultrasound.

For stakeholders' meetings of approximately 40 participants, participants can be split into at least two groups (or another even number of groups). To ensure diversity of opinions and experience, each of the groups should have a balanced composition of medical doctors, midwives and nurses, public health officers, and representatives from community health organizations and implementing partners. Group members should select a note-taker and a presenter.

The first (and all odd-numbered) group(s) is then assigned to review: (i) the contents of Contacts 1, 2, 3 (first and second trimester), including essential good practices for each of these contacts; and (ii) the SWOT analysis for any ANC recommendations requiring a new or updated country policy (BAT worksheet 5).

The second (and all even numbered) group(s) is then assigned to review: (i) the contents of Contacts 4, 5, 6, 7, 8 (third trimester), including essential good practices for each of these contacts; and (ii) the SWOT analysis for any ANC recommendations requiring a new or updated country policy (BAT worksheet 5).

Essential practices to be reviewed for every contact include:

Essential good clinical practices
• Maternal weight/height measurement
• Blood pressure measurement
• Fetal heart rate (FHR) auscultation
• Clinical estimate of gestational age
• Abdominal palpation to detect breech presentation (third trimester contacts)
• Urinary dipstick test for proteinuria
• Blood-typing and Rh at first contact

Other essential counselling
• Birth preparedness
• Labour companion
• Family planning counselling
• Lactation counselling
• Management of pain during labour

Groups should be given at least 2 hours for the group work. The chosen presenter may then report the group's consolidated considerations and identified modifications to the package of ANC interventions and the SWOT analysis in the plenary session.

Below, please find printable materials for each of the groups. Please note that the activities column must be tailored to the country setting based on the situational analysis (reflecting Output 1 from the BAT) and which context-specific recommendations apply. If there are no data (e.g. regarding calcium intake), the activity may be maintained on the list and highlighted in yellow for specific discussion by the group, as depicted below.

**Activities (context-specific)**	**Which cadre** (community health worker, midwife, etc.)	**Level** (household, health centre, etc.)	**Phasing, if any** (medium term, long term)
A.1.2. Nutritional education on increasing daily energy and protein intake			
A.1.3. Balanced energy and protein supplementation in undernourished populations			
A.3. Calcium supplements			
A.4. Vitamin A supplements			
A.10. Restricting caffeine intake			
B.1.1. Anaemia			
B.1.2. Asymptomatic bacteriuria (ASB)			
B.1.3. Intimate partner violence (IPV)			
B.1.8. Tuberculosis (TB)			
C.4. Preventive anthelminthic treatment			
C.6. Malaria prevention: intermittent preventive treatment in pregnancy (IPTp)			
C.7. Pre-exposure prophylaxis (PrEP) for HIV prevention			
D.2.–D.6. Heartburn, leg cramps, low back and pelvic pain, constipation, varicose veins and oedema			

GROUP 1 – PART I: Routine package of ANC for eight contacts

First trimester

✔ **Contact 1: (up to 12 weeks)**

**Activities (context-specific)**	**Which cadre** (community health worker, midwife, etc.)	**Level** (household, health centre, etc.)	**Phasing, if any** (medium term, long term)
**Nutritional interventions**			
A.1.1. Nutritional counselling on a healthy diet and physical activity			
A.1.2. Nutritional education on increasing daily energy and protein intake			
A.1.3. Balanced energy and protein supplementation in undernourished populations			
A.2.1. Daily iron and folic acid supplements<br>or<br>A.2.2 Intermittent oral iron and folic acid supplementation (depending on population characteristics)			
A.3. Calcium supplements			
A.4. Vitamin A supplements			
A.10. Restricting caffeine intake			
**Medical assessment**			
B.1.1. Anaemia			
B.1.2. Asymptomatic bacteriuria (ASB)			
B.1.3. Intimate partner violence (IPV)			
B.1.4. Gestational diabetes mellitus (GDM)			
B.1.5. Tobacco use			
B.1.6. Substance use			
B.1.7. Human immunodeficiency virus (HIV) and syphilis			
B.1.8. Tuberculosis (TB)			
B.2.4. Ultrasound scan			
**Preventive measures**			
C.1. Antibiotics for asymptomatic bacteriuria (ASB)			
C.5. Tetanus toxoid vaccination			
C.7 PrEP for HIV prevention			
**Interventions for common physiological symptoms**			
D.1. Nausea and vomiting			
D.2.–D.6. Heartburn, leg cramps, low back and pelvic pain, constipation, varicose veins and oedema			

CONTACT 1	
**List the essential good clinical practices and other essential counselling that are relevant for this contact.**	
**List the activities that the working group did not agree on, if any**	
**List the activities that the working group added, if any**	

Second trimester

✔ **Contact 2: 20 weeks and contact 3: 26 weeks**

**Activities (context-specific)**	**Which cadre** (community health worker, midwife, etc.)	**Level** (household, health centre, etc.)	**Phasing, if any** (medium term, long term)
**Nutritional interventions**			
A.1.1. Nutritional counselling on a healthy diet and physical activity			
A.1.2. Nutritional education on increasing daily energy and protein intake			
A.1.3. Balanced energy and protein supplementation in undernourished populations			
A.2.1. Daily iron and folic acid supplements<br>or<br>A.2.2 Intermittent oral iron and folic acid supplementation (depending on population characteristics)			
A.3. Calcium supplements			
A.4. Vitamin A supplements			
A.10. Restricting caffeine intake			
**Medical assessment**			
B.1.3. Intimate partner violence (IPV)			
B.1.4. Gestational diabetes mellitus (GDM)			
B.1.5. Tobacco use			
B.1.6. Substance use			
B.1.11. Ultrasound scan *(not needed at contact 3)*			
B.1.6. Substance use			
**Preventive measures**			
C.4. Preventive anthelminthic treatment *(not needed at contact 3)*			
C.5. Tetanus toxoid vaccination			
C.6. Malaria prevention: intermittent preventive treatment in pregnancy (IPTp)			
**Interventions for common physiological symptoms**			
D.1. Nausea and vomiting			
D.2.–D.6. Heartburn, leg cramps, low back and pelvic pain, constipation, varicose veins and oedema			

CONTACT 2	
**List the essential good clinical practices and other essential counselling that are relevant for this contact**	
**List the activities that the working group did not agree on, if any**	
**List the activities that the working group added, if any**	

✔ **Additional activities for Contact 3: 26 weeks (unless otherwise stated, all contact 2 activities apply)**

**Activities (context-specific)**	**Which cadre** (community health worker, midwife, etc.)	**Level** (household, health centre, etc.)	**Phasing, if any** (medium term, long term)
**Medical assessment**			
B.1.1. Anaemia			
B.1.2. Asymptomatic bacteriuria (ASB)			
**Preventive measures**			
C.1. Antibiotics for asymptomatic bacteriuria (ASB)			

CONTACT 3	
**List the essential good clinical practices and other essential counselling that are relevant for this contact**	
**List the activities that the working group did not agree on, if any**	
**List the activities that the working group added, if any**	

Essential good clinical practices

- Maternal weight/height measurement
- Blood pressure measurement
- Fetal heart rate (FHR) auscultation
- Clinical estimate of gestational age
- Abdominal palpation to detect breech presentation (third trimester contacts)
- Urinary dipstick test for proteinuria
- Blood-typing and Rh at first contact

Other essential counselling

- Birth preparedness
- Labour companion
- Family planning counselling
- Lactation counselling
- Management of pain during labour

GROUP 2 – PART I: Routine package of ANC interventions for eight contacts

Third trimester (unless otherwise stated, all activities apply to contacts 4–8)

- ✔ **Contact 4: 30 weeks**
- ✔ **Contact 5: 34 weeks**
- ✔ **Contact 6: 36 weeks**
- ✔ **Contact 7: 38 weeks**
- ✔ **Contact 8: 40 weeks**

**Activities (context-specific)**	**Which cadre** (community health worker, midwife, etc.)	**Level** (household, health centre, etc.)	**Phasing, if any** (medium term, long term)
**Nutritional interventions**			
A.1.1. Nutritional counselling on a healthy diet and physical activity			
A.1.2. Nutritional education on increasing daily energy and protein intake			
A.1.3. Balanced energy and protein supplementation in undernourished populations			
A.2.1. Daily iron and folic acid supplements<br>or<br>A.2.2. Intermittent oral iron and folic acid supplementation (depending on population characteristics)			
A.3. Calcium supplements			
A.4. Vitamin A supplements			
A.10. Restricting caffeine intake			
**Medical assessment**			
B.1.1. Anaemia *(only for contact 6)*			
B.1.2. Asymptomatic bacteriuria (ASB) *(only for contact 5)*			
B.1.3. Intimate partner violence (IPV)			
B.1.4. Gestational diabetes mellitus (GDM)			
B.1.5. Tobacco use			
B.1.6. Substance use			
**Preventive measures**			
C.1. Antibiotics for asymptomatic bacteriuria (ASB) *(only for contact 5)*			
C.6. Malaria prevention: intermittent preventive treatment in pregnancy (IPTp)			
**Interventions for common physiological symptoms**			
D.2.–D.6. Heartburn, leg cramps, low back and pelvic pain, constipation, varicose veins and oedema			

CONTACT 4

**List the essential good clinical practices and other essential counselling that are relevant for this contact**	
**List the activities that the working group did not agree on, if any**	
**List the activities that the working group added, if any**	

CONTACT 5

**List the essential good clinical practices and other essential counselling that are relevant for this contact**	
**List the activities that the working group did not agree on, if any**	
**List the activities that the working group added, if any**	

CONTACT 6

**List the essential good clinical practices and other essential counselling that are relevant for this contact**	
**List the activities that the working group did not agree on, if any**	
**List the activities that the working group added, if any**	

CONTACT 7

**List the essential good clinical practices and other essential counselling that are relevant for this contact**	
**List the activities that the working group did not agree on, if any**	
**List the activities that the working group added, if any**	

CONTACT 8

**List the essential good clinical practices and other essential counselling that are relevant for this contact**	
**List the activities that the working group did not agree on, if any**	
**List the activities that the working group added, if any**	

Essential good clinical practices

- Maternal weight/height measurement
- Blood pressure measurement
- Fetal heart rate (FHR) auscultation
- Clinical estimate of gestational age
- Abdominal palpation to detect breech presentation (third trimester contacts)
- Urinary dipstick test for proteinuria
- Blood-typing and Rh at first contact

Other essential counselling

- Birth preparedness
- Labour companion
- Family planning counselling
- Lactation counselling
- Management of pain during labour

10. National ANC guideline table of contents template

Once the country-specific package of ANC interventions (BAT worksheet 4) has been discussed by stakeholders, a national ANC guideline should be drafted based on those results. A possible table of contents for a national ANC guideline is given below. This table of contents template is aimed at guiding countries in their development of an updated ANC policy (after completing the mapping) which should align with the longer-term national reproductive health strategy.

After the table of contents for a national ANC guideline has been drafted, stakeholders should meet again to validate the new or updated guideline, prepare a long- and short-term implementation plan, and discuss the updating of HMIS tools (e.g. facility registers, mothers' cards, data reporting forms).

Training materials are under development by WHO to support health workers to implement the new ANC recommendations.

ANC guideline table of contents

I. Foreword

II. Acknowledgements

III. Acronyms

IV. Background

- a. Situational analysis (written summary of BAT worksheet 2)
- b. Rationale for ANC guidelines
- c. Guiding principles of ANC service delivery
- d. Governance, coordination and organization of ANC health services
- e. Organization of ANC services

V. ANC contact schedule

- a. New schedule of ANC contacts
- b. Package of ANC interventions (section 11 of this instruction manual)

VI. Providing ANC services

- a. History-taking
- b. Maternal assessment
- c. Fetal assessment
- d. Point-of-care tests
- e. Laboratory tests
- f. Preventive measures

VII. Counselling and information provision

- a. Caffeine intake
- b. Smoking, alcohol and substance abuse
- c. Intimate partner violence (IPV)
- d. Birth preparedness
- e. Complication readiness
- f. Counselling on family planning
- g. Routine and follow-up visits

VIII. Nutrition in pregnancy

- a. Weight gain
- b. Nutrition and diet
- c. Physical activity
- d. Iron, folic acid, calcium and other supplementation
- e. Key messages

IX. Standard precautions for infection control and prevention

- a. Infection control and prevention
- b. Hand washing
- c. House keeping
- d. Waste management

X. Community-based ANC service provision

- a. Community awareness and engagement for ANC
- b. Key messages
- c. Community engagement platforms

XI. Monitoring and evaluation of ANC

- a. Data management
- b. Monitoring of ANC programme
- c. Research

XII. References

11. Example of package of ANC interventions

This table gives an example of how an package of ANC interventions may be detailed in the national ANC guideline (section V.b in the table of contents in section 10 of this instruction manual).

Contact	Maternal assessment	Fetal assessment	Counselling	Point-of-care tests	Labs	Radiological tests	Preventive measures	Essential good clinical practices	Treat/ manage	Others
**Initial contact regardless of gestation**	• Full history<br>• Clinical estimation of gestational age<br>• Exhaustive physical examination<br>• Abdominal palpation<br>• Tuberculosis (TB) screening<br>• Screening for intimate partner violence (IPV)	• Refer to the gestational age	• Nutrition, diet, physical activity and rest<br>• HIV and sexually transmitted infection (STI) tests<br>• Adherence to medicines prescribed<br>• Avoid caffeine intake, alcohol, tobacco and substance abuse<br>• Use of long-lasting insecticidal nets (LLINs)<br>• Danger signs and<br>• emergency preparedness<br>• Early stimulation of the baby<br>• Family planning<br>• Importance of attending all eight ANC contacts	• HIV<br>• Syphilis,<br>• Hepatitis B<br>• Urine dipstick<br>• Malaria<br>• Haemoglobin (Hb) estimation<br>• Asymptomatic bacteriuria (Gram stain or culture) (depending on facility)	• Blood group and Rhesus<br>• Full blood count	• Ultrasound	• Iron and folic acid (IFA)<br>• Tetanus toxoid/ tetanus diphtheria vaccine<br>• Treat or advise about relief of common physiological symptoms<br>• Combination prevention: pre-exposure prophylaxis for HIV (PrEp), condom, voluntary medical male circumcision (VMMC) for men	• Maternal weight/height measurement<br>• Mid-upper arm circumference (MUAC)<br>• Blood pressure measurement<br>• Male involvement<br>• STI screening	• Common physiological disorders including STIs<br>• Complications of early pregnancy	• Complete ANC card and give to woman<br>• Fill ANC register/ community management information system (CMIS)

Contact	Maternal assessment	Fetal assessment	Counselling	Point-of-care tests	Labs	Radiological tests	Preventive measures	Essential good clinical practices	Treat/ manage	Others
**Contact 1 at 8–12 weeks**	• Full history<br>• Clinical estimation of gestational age<br>• Exhaustive physical examination<br>• Abdominal palpation<br>• TB screening<br>• Screening for IPV		• Nutrition, diet and physical activity, rest, HIV and STI test<br>• Adherence to medicines prescribed<br>• Avoid caffeine intake, alcohol, tobacco and substance abuse<br>• Use of LLINs<br>• Danger signs<br>• Emergency preparedness<br>• Early stimulation of the baby<br>• Family planning	• HIV<br>• Syphilis<br>• Hepatitis B<br>• Urine dipstick<br>• Malaria<br>• Hb estimation	• Blood group and Rhesus<br>• Full blood count<br>• Check for ASB (Gram stain or culture)	• Ultrasound	• Iron and folic acid<br>• TT/TD<br>• Treat or advise about relief of common physiological symptoms<br>• Combination prevention: PrEp, condom, voluntary medical male circumcision (VMMC) for men<br>• Antibiotics for ASB	• Maternal weight/height measurement<br>• MUAC<br>• Blood pressure measurement<br>• Male involvement<br>• STI screening	• Common physiological disorders including STIs<br>• Complications of early pregnancy	• Complete ANC card and give to woman<br>• Fill in ANC register/CMIS
**Contact 2 at 20 weeks**	• Ask the mother if she has experienced any problems/ changes since last visit<br>• Clinical estimation of gestational age<br>• Physical examination<br>• Symphysis-fundal height measurement/ abdominal palpation<br>• TB screening<br>• Screening for IPV	• FHR/fetal heart sound auscultation after 20 weeks<br>• Ask about fetal movement	• Nutrition, diet, physical activity and rest<br>• HIV and STI test<br>• Adherence to medicines prescribed<br>• Avoid caffeine intake, alcohol, tobacco and substance abuse<br>• Use of LLINs<br>• Danger signs<br>• Emergency preparedness<br>• Early stimulation of the baby<br>• Family planning	• Urine dipstick, HIV, syphilis, retest for those who were negative or not tested in the first contact<br>• Hepatitis B<br>• Malaria		• Ultrasound if not yet done	• Anthelmintic (single-dose mebendazole 500 mg)<br>• IFA<br>• Calcium<br>• Combination prevention: PrEp, condom, VMMC for men	• Maternal weight measurement<br>• MUAC<br>• Blood pressure measurement<br>• Male involvement<br>• STI screening	• Common physiological disorders including STIs<br>• Complications of pregnancy	• Complete ANC card and give to woman<br>• Fill in ANC register/CMIS

Contact	Maternal assessment	Fetal assessment	Counselling	Point-of-care tests	Labs	Radiological tests	Preventive measures	Essential good clinical practices	Treat/ manage	Others
Contact 3 at 26 weeks	• Ask the mother if she has experienced any problems/ changes since last visit<br>• Clinical estimation of gestational age<br>• Physical examination<br>• Symphysis-fundal height measurement/ abdominal palpation<br>• TB screening<br>• Screening for IPV<br>• Rule out premature rupture of membranes (PROM)	• Fetal heart rate (FHR) auscultation<br>• Ask about fetal movements	• Nutrition, diet, physical activity, and rest<br>• HIV and STI test<br>• Adherence to medicines prescribed<br>• Avoid caffeine intake, alcohol, tobacco and substance abuse<br>• Use of LLINs<br>• Danger signs<br>• Emergency preparedness<br>• Early stimulation of the baby<br>• Family planning	• Urine dipstick Malaria	• Hb<br>• ASB	• Ultrasound if indicated	• IFA<br>• calcium<br>• Combination prevention: PrEp, condom VMMC for men	• Maternal weight measurement<br>• MUAC<br>• Blood pressure measurement<br>• Male involvement<br>• STI screening	• Common physiological disorders including STIs<br>• Complications of pregnancy	• Complete ANC card and give to woman<br>• Fill ANC register/CMIS
Contact 4 at 30 weeks	• Ask how the mother is doing<br>• Clinical estimation of gestational age, physical examination<br>• Symphysis-fundal height measurement/ abdominal palpation<br>• TB screening<br>• Screening for IPV<br>• Ask about fetal movement<br>• Rule out PROM	• FHR auscultation<br>• Ask about fetal movements	• Nutrition, diet physical activity, and rest<br>• HIV and STI test<br>• Adherence to medicines prescribed<br>• Avoid caffeine intake, alcohol, tobacco and substance abuse<br>• Use of LLINs<br>• Danger signs<br>• Emergency preparedness<br>• Early stimulation of the baby<br>• Family planning	• Urine dipstick<br>• HIV and syphilis re-test for those who were negative<br>• Malaria		• Ultrasound if indicated	• IFA<br>• Calcium<br>• Combination prevention (PrEp, condom VMMC for men)	• Maternal weight measurement<br>• MUAC<br>• Blood pressure measurement<br>• Male involvement<br>• STI screening	• Common physiological disorders<br>• Manage complications of pregnancy	• Complete ANC card and give to woman<br>• Fill ANC register/CMIS

Contact	Maternal assessment	Fetal assessment	Counselling	Point-of-care tests	Labs	Radiological tests	Preventive measures	Essential good clinical practices	Treat/ manage	Others
**Contact 5 at 34 weeks**	• Ask how the mother is doing<br>• Clinical estimation of gestational age<br>• Physical examination<br>• Symphysis fundal height measurement/ abdominal palpation<br>• TB screening<br>• Screening for IPV<br>• Rule out PROM	• FHR auscultation<br>• Ask about fetal movements	• Nutrition, diet and physical activity, rest, HIV and STI test<br>• Adherence to medicines prescribed,<br>• Avoid caffeine intake, alcohol, tobacco and substance abuse<br>• Use of LLINs<br>• Danger signs<br>• Emergency preparedness<br>• Early stimulation of the baby<br>• Family planning	• Urine dipstick, malaria test	• Check ASB	• Ultrasound if indicated	• IFA, calcium, combination prevention (PrEp, condom VMMC for men)	• Maternal weight measurement, MUAC<br>• Blood pressure measurement,<br>• male involvement, STI screening	• Common physiological disorders<br>• Manage complications of pregnancy	• Complete ANC card and give to woman<br>• Fill ANC register/CMIS
**Contact 6 at 36 weeks**	• Ask how the mother is doing<br>• Clinical estimation of gestational age<br>• Physical examination<br>• Symphysis-fundal height measurement/ abdominal palpation<br>• TB screening<br>• Screening for IPV<br>• Rule out PROM	• FHR auscultation<br>• Ask about fetal movements	• Nutrition, diet, physical activity, and rest<br>• HIV and STI test<br>• Adherence to medicines prescribed<br>• Avoid caffeine intake, alcohol, tobacco and substance abuse<br>• Use of LLINs<br>• Danger signs<br>• Emergency preparedness<br>• Early stimulation of the baby<br>• Family planning	• Urine dipstick<br>• Malaria	• Hb	• Ultrasound if indicated	• IFA<br>• Calcium<br>• Combination prevention: PrEp, condom VMMC for men	• Maternal weight measurement<br>• MUAC<br>• Blood pressure measurement<br>• Male involvement<br>• STI screening	• Common physiological disorders<br>• Manage complications of pregnancy	• Complete ANC card and give to woman<br>• Fill ANC register/CMIS

Contact	Maternal assessment	Fetal assessment	Counselling	Point-of-care tests	Labs	Radiological tests	Preventive measures	Essential good clinical practices	Treat/ manage	Others
**Contact 7 at 38 weeks**	• Ask how the mother is doing<br>• Clinical estimation of gestational age<br>• Physical examination<br>• Symphysis fundal height measurement/ abdominal palpation<br>• TB screening<br>• Screening for IPV<br>• Rule out PROM	• FHR auscultation<br>• Ask about fetal movements	• Nutrition, diet, physical activity, and rest<br>• HIV test<br>• Avoid: caffeine intake, alcohol, tobacco and substance abuse<br>• Use of LLINs<br>• Danger signs<br>• Emergency preparedness<br>• Infant and young child feeding (IYCF)<br>• Breastfeeding<br>• Breast care<br>• Family planning/ Healthy Timing and Spacing of Pregnancy (HTSP)<br>• Birth planning<br>• Early infant male circumcision (EIMC)<br>• Early stimulation of the baby	• Urine dipstick<br>• HIV and Syphilis re-test for those who were negative<br>• Malaria		• Ultrasound if indicated	• IFA<br>• Calcium<br>• Combination prevention: PrEp, condom VMMC for men	• Maternal weight measurement<br>• MUAC<br>• Blood pressure measurement<br>• Male involvement<br>• STI screening	• Common physiological disorders<br>• Manage complications of pregnancy	• Complete ANC card and give to woman<br>• Fill ANC register/CMIS

Contact	Maternal assessment	Fetal assessment	Counselling	Point-of-care tests	Labs	Radiological tests	Preventive measures	Essential good clinical practices	Treat/ manage	Others
**Contact 8 at 40 weeks**	• Ask how the mother is doing since the last contact<br>• Clinical estimation of gestational age<br>• Physical examination<br>• Symphysis fundal height measurement/ abdominal palpation<br>• TB screening<br>• Screening for IPV<br>• Rule out PROM<br>• Ask about symptoms of labour	• FHR auscultation<br>• Ask about fetal movements	• Nutrition, diet, physical activity, and rest<br>• HIV test<br>• Avoid: caffeine intake, alcohol, tobacco and substance abuse<br>• Use of LLINs<br>• Danger signs<br>• Emergency preparedness<br>• IYCF<br>• Breastfeeding<br>• Breast care<br>• Family Planning/ HTSP<br>• Birth planning<br>• EIMC<br>• Early stimulation of the baby<br>• Counsel on admission if no signs of labour at 41 weeks	• Urine dipstick<br>• Malaria		• Ultrasound if indicated	• IFA<br>• Calcium<br>• Combination prevention: PrEP, condom VMMC for men	• Maternal weight measurement<br>• MUAC<br>• Blood pressure measurement<br>• Male involvement<br>• STI screening	• Common physiological disorders<br>• Manage complications of pregnancy	• Complete ANC card and give to woman<br>• Fill ANC register/CMIS
**Cadres to provide the services**	• Midwives, obstetricians, doctors	• Midwives, obstetricians, doctors	• Midwife, nurses, dieticians, nutritionists, health promotion, community health workers, doctors, obstetricians	• Midwives, nurses, doctors	• Lab technician	• Radiology, obstetricians, midwives, sonographers (if any)	• Nurses and midwifes	• Midwives, (blood pressure – BP), obstetricians (BP), doctors (BP); community health workers (weight, MUAC, height)	• Midwives, doctors, obstetricians	• Midwives, obstetricians, doctors

Notes:
- ANC services are offered at health posts and health centers, and district, provincial and referral hospitals.
- All cadres will be expected to provide information, education and communication. All midwives will be expected to provide individual counselling.
- Complete ANC card, enter information into the register, and give the card to the pregnant woman.
- Refer women with complications to the next level of care.
- Health promotion will be done for community engagement.

References

1. World Health Organization. WHO Recommendations on antenatal care for a positive pregnancy experience. Geneva: WHO, 2016. (https://www.who.int/reproductivehealth/publications/maternal_perinatal_health/anc-positive-pregnancy-experience/en/, accessed 28 November 2019).
2. de Masi S, Bucagu M, Tunçalp Ö, Peña-Rosas JP, Lawrie T, Oladapo OT et al. Integrated person-centered health care for all women during pregnancy: implementing World Health Organization recommendations on antenatal care for a positive pregnancy experience. Global Health: Science and Practice. 2017;5(2):197–201. doi:10.9745/GHSP-D-17-00141.
3. Tunçalp Ö, Pena-Rosas JP, Lawrie T, Bucagu M, Oladapo OT, Portela A, et al. WHO recommendations on antenatal care for a positive pregnancy experience—going beyond survival. BJOG: An International Journal of Obstetrics & Gynaecology. 2017;124(6):860–2. doi:10.1111/1471-0528.14599.
4. Fischer F, Lange K, Klose K, Greiner W, Kraemer A. Barriers and strategies in guideline implementation—a scoping review. Healthcare. 2016;4(3). doi:10.3390/healthcare4030036.
5. Barreix et al. (XXX)
6. Monitoring the building blocks of health systems: a handbook of indicators and their measurement strategies. Geneva: World Health Organization; 2010. (https://www.who.int/healthinfo/systems/monitoring/en/; accessed 28 November 2019).
7. Lazarus JV, France T. A new era for the WHO health system building blocks? Health Systems Global; 22 August 2014 (https://www.healthsystemsglobal.org/blog/9/A-new-era-for-the-WHO-health-system-building-blocks-.html; accessed 28 November 2019).
8. Downe S, Finlayson K, Tunçalp Ö, Gülmezoglu AM. Provision and uptake of routine antenatal services: a qualitative evidence synthesis. Cochrane Database Syst Rev. 2019(6):CD012392.pub2.
9. Health statistics and information systems: Service availability and readiness assessment (SARA). Geneva: World Health Organization; 2019. (https://www.who.int/healthinfo/systems/sara_introduction/en/; accessed 28 November 2019).
10. WHO antenatal care randomized trial: manual for the implementation of the new model. Geneva: World Health Organization; 2002. (http://www.who.int/reproductivehealth/publications/maternal_perinatal_health/RHR_01_30/en/, accessed 28 November 2019).
11. Downe S, Finlayson K, Lawrie T, Lewin S, Glenton C, Rosenbaum S, et al. Using qualitative evidence synthesis to inform guideline scope and develop qualitative findings statements. Health Research Policy and Systems. 2019;17(1):76. doi:10.1186/s12961-019-0467-5.
12. Guidelines for the identification and management of substance use and substance use disorders in pregnancy. Geneva: World Health Organization; 2014 (https://www.who.int/substance_abuse/publications/pregnancy_guidelines/en/, accessed 28 November 2019).
13. Systematic screening for active tuberculosis: principles and recommendations. Geneva: World Health Organization; 2013 (https://www.who.int/tb/tbscreening/en/, accessed 28 November 2019).
14. Manual of diagnostic ultrasound, second edition. Geneva: World Health Organization; 2013 (https://www.who.int/medical_devices/publications/manual_ultrasound_pack1-2/en/, accessed 28 November 2019).
15. AIUM-ACR-ACOG-SMFM-SRU practice parameter for the performance of obstetric ultrasound examinations. Laurel (MD): American Institute of Ultrasound in Medicine (AIUM); 2013 (https://www.aium.org/resources/guidelines/obstetric.pdf, accessed 28 November 2019).
16. Maternal immunization against tetanus: integrated management of pregnancy and childbirth (IMPAC). Standards for maternal and neonatal care 1.1. Geneva: Department of Making Pregnancy Safer, World Health Organization;

2006 (https://www.who.int/reproductivehealth/publications/maternal_perinatal_health/immunization_tetanus.pdf, accessed 28 November 2019).

17. Guidelines for the treatment of malaria, third edition. Geneva: World Health Organization; 2015 (https://www.who.int/malaria/publications/atoz/9789241549127/en/, accessed 28 November 2019).
18. WHO recommendations: optimizing health worker roles to improve access to key maternal and newborn health interventions through task shifting. Geneva: World Health Organization; 2012 (https://www.who.int/reproductivehealth/publications/maternal_perinatal_health/978924504843/en/, accessed 28 November 2019).
19. Increasing access to health workers in remote and rural areas through improved retention: global policy recommendations. Geneva: World Health Organization; 2010 (https://www.who.int/hrh/retention/guidelines/en/, accessed 28 November 2019).
20. Healthy diet. Fact sheet no. 394. Geneva: World Health Organization; 2015 (https://www.who.int/nutrition/publications/nutrientrequirements/healthydiet_factsheet394.pdf/, accessed 28 November 2019).
21. UK Chief Medical Officers' Physical Activity Guidelines. 7 September 2019 (https://assets.publishing.service.gov.uk/government/uploads/system/uploads/attachment_data/file/832868/uk-chief-medical-officers-physical-activity-guidelines.pdf/, accessed 11 December 2019).
22. Rasmussen KM, Yaktine AL, editors; Weight gain during pregnancy: re-examining the guidelines. Washington (DC): The National Academies Press; 2009.
23. Physical status: the use and interpretation of anthropometry: report of a WHO Expert Committee. Technical Report Series no. 854. Geneva: World Health Organization; 1995 (https://apps.who.int/iris/bitstream/handle/10665/37003/WHO_TRS_854.pdf?sequence=1, accessed 28 November 2019).
24. Tang AM, Chung M, Dong K, Terrin N, Edmonds A, Assefa N et al. Determining a global mid-upper arm circumference cutoff to assess malnutrition in pregnant women. Washington (DC): FHI 360/Food and Nutrition Technical Assistance III Project (FANTA); 2016 (https://www.fantaproject.org/sites/default/files/resources/FANTA-MUAC-cutoffs-pregnant-women-June2016.pdf, accessed 28 November 2019).
25. Guideline: daily iron and folic acid supplementation in pregnant women. Geneva: World Health Organization; 2012 (https://www.who.int/nutrition/publications/micronutrients/guidelines/daily_ifa_supp_pregnant_women/en/, accessed 28 November 2019).
26. The clinical use of blood in general medicine, obstetrics, paediatrics, surgery & anaesthesia, trauma & burns. Geneva: World Health Organization; 1998 (https://www.who.int/bloodsafety/clinical_use/en/Manual_EN.pdf, accessed 28 November 2019).
27. WHO; de Benoist B, McLean E, Egli I, Cogswell M, editors. Worldwide prevalence of anaemia 1993–2005. WHO global database on anaemia. Geneva: World Health Organization (WHO); 2008 (https://apps.who.int/iris/bitstream/10665/43894/1/9789241596657_eng.pdf, accessed 28 November 2019.
28. Iron and folate supplementation: integrated management of pregnancy and childbirth (IMPAC). Standards for maternal and neonatal care 1.8. Geneva: Department of Making Pregnancy Safer, World Health Organization; 2006 (https://www.who.int/reproductivehealth/publications/maternal_perinatal_health/iron_folate_supplementation.pdf, accessed 28 November 2019).
29. The international pharmacopoeia, 9th edition. Geneva: World Health Organization; 2019 (https://apps.who.int/phint/en/p/about, accessed 28 November 2019).
30. The WHO Expert Committee on Specifications for Pharmaceutical Preparations. Quality assurance of pharmaceuticals: meeting a major public health challenge. Geneva: World Health Organization; 2007 (WHO/PSM/QSM/2007.5; https://www.who.int/medicines/publications/brochure_pharma.pdf, accessed 28 November 2019).
31. Guideline: intermittent iron and folic acid supplementation in non-anaemic pregnant women. Geneva: World Health Organization; 2012 (https://apps.who.int/iris/bitstream/10665/75335/1/9789241502016_eng.pdf, accessed 28 November 2019).

32. The global prevalence of anaemia in 2011. Geneva: World Health Organization; 2015 (https://apps.who.int/iris/bitstream/10665/177094/1/9789241564960_eng.pdf, accessed 28 November 2019).

33. WHO recommendations for prevention and treatment of pre-eclampsia and eclampsia. Geneva: World Health Organization; 2011 (https://apps.who.int/iris/bitstream/10665/44703/1/9789241548335_eng.pdf, accessed 28 November 2019).

34. Guideline: calcium supplementation in pregnant women. Geneva: World Health Organization; 2013 (https://apps.who.int/iris/bitstream/10665/85120/1/9789241505376_eng.pdf, accessed 28 November 2019).

35. Hofmeyr GJ, Lawrie TA, Atallah ÁN, Duley L, Torloni MR. Calcium supplementation during pregnancy for preventing hypertensive disorders and related problems. Cochrane Database Syst Rev. 2014;(6):CD001059.

36. Guideline: vitamin A supplementation in pregnant women. Geneva: World Health Organization; 2011 (https://apps.who.int/iris/bitstream/10665/44625/1/9789241501781_eng.pdf, accessed 28 November 2019).

37. Sommer A, Davidson FR. Assessment and control of vitamin A deficiency: the Annecy Accords. J Nutr. 2002;132(9):2845S–50S. doi:10.1093/jn/132.9.2845S.

38. Safe vitamin A dosage during pregnancy and lactation. Recommendations and report of a consultation. Micronutrient series. Geneva: World Health Organization; 1998 (WHO/NUT/98.4; https://apps.who.int/iris/bitstream/10665/62838/1/WHO_NUT_98.4_eng.pdf, accessed 28 November 2019).

39. Responding to intimate partner violence and sexual violence against women: WHO clinical and policy guidelines. Geneva: World Health Organization; 2013 (https://www.who.int/reproductivehealth/publications/violence/9789241548595/en/, accessed 28 November 2019).

40. Diagnostic criteria and classification of hyperglycaemia first detected in pregnancy. Geneva: World Health Organization; 2013 (WHO/NMH/MND/13.2; https://www.who.int/diabetes/publications/Hyperglycaemia_In_Pregnancy/en/, accessed 28 November 2019).

41. Antenatal care for uncomplicated pregnancies: clinical guideline [CG62]. UK: National Institute for Health and Clinical Excellence; 2008 (updated 2019) (https://www.nice.org.uk/guidance/cg62, accessed 28 November 2019).

42. WHO recommendations for the prevention and management of tobacco use and second-hand smoke exposure in pregnancy. Geneva: World Health Organization; 2013 (https://www.who.int/tobacco/publications/pregnancy/guidelinestobaccosmokeexposure/en/, accessed 28 November 2019).

43. Consolidated guidelines on HIV testing services. Geneva: World Health Organization; 2015 (https://www.who.int/hiv/pub/guidelines/hiv-testing-services/en/, accessed 28 November 2019).

44. Guideline on when to start antiretroviral therapy and on pre-exposure prophylaxis for HIV. Geneva: World Health Organization; 2015 (https://www.who.int/hiv/pub/guidelines/earlyrelease-arv/en/, accessed 28 November 2019).

45. Prevention of mother-to-child transmission of syphilis: integrated management of pregnancy and childbirth (IMPAC). Standards for maternal and neonatal care 1.3. Geneva: Department of Making Pregnancy Safer, World Health Organization; 2006 (https://www.who.int/reproductivehealth/publications/maternal_perinatal_health/prevention_mtct_syphilis.pdf, accessed 28 November 2019).

46. WHO guidelines for the treatment of *Chlamydia trachomatis*. Geneva: World Health Organization; 2016 (https://www.who.int/reproductivehealth/publications/rtis/chlamydia-treatment-guidelines/en/, accessed 28 November 2019).

47. WHO guidelines for the treatment of *Neisseria gonorrhoeae*. Geneva: World Health Organization; 2016 (https://www.who.int/reproductivehealth/publications/rtis/gonorrhoea-treatment-guidelines/en/, accessed 28 November 2019).

48. WHO guidelines for the treatment of *Treponema pallidum* (syphilis). Geneva: World Health Organization; 2016 (https://www.who.int/reproductivehealth/publications/rtis/syphilis-treatment-guidelines/en/, accessed 28 November 2019).

49. Prevention of sexual transmission of Zika virus: interim guidance. Geneva: World Health Organization; 2016 (https://www.who.int/csr/resources/publications/zika/sexual-transmission-prevention/en/, accessed 28 November 2019).
50. Delaram M, Shams S. The effect of foetal movement counting on maternal anxiety: a randomised, controlled trial. J Obstet Gynaecol. 2015:39–43. doi:10.3109/01443615.2015.1025726.
51. Pearson JF, Weaver JB. Fetal activity and fetal wellbeing: an evaluation. Br Med J. 1976;1(6021):1305–7. doi:10.1136/bmj.1.6021.1305.
52. McClure EM, Nathan RO, Saleem S, Esamai F, Garces A, Chomba E et al. First look: a cluster-randomized trial of ultrasound to improve pregnancy outcomes in low income country settings. BMC Pregnancy Childbirth. 2014;14:73. doi:10.1186/1471-2393-14-73.
53. Manual of diagnostic ultrasound, second edition. Geneva: World Health Organization; 2013 (https://www.who.int/medical_devices/publications/manual_ultrasound_pack1-2/en/, accessed 28 November 2019).
54. Oluoch DA, Mwangome N, Kemp B, Seale AC, Koech AC, Papagrotghiou AT et al. "You cannot know if it's a baby or not a baby": uptake, provision and perceptions of antenatal care and routine ultrasound scanning in rural Kenya. BMC Pregnancy Childbirth. 2015;15:127. doi:10.1186/s12884-015-0565-5.
55. Heyman B, Hundt G, Sandall J, Spencer K, Williams C, Grellier R, Pitson L. On being at higher risk: a qualitative study of prenatal screening for chromosomal anomalies. Soc Sci Med. 2006(60);2359–72. doi:10.1016/j.socscimed.2005.10.018.
56. WHO recommendations for prevention and treatment of maternal peripartum infections. Geneva: World Health Organization; 2015 (https://apps.who.int/iris/bitstream/10665/186171/1/9789241549363_eng.pdf, accessed 28 November 2019).
57. Crowther C, Middleton P. Anti-D administration after childbirth for preventing rhesus alloimmunization. Cochrane Database Syst Rev. 1997;(2):CD000021.
58. Guideline: preventive chemotherapy to control soil-transmitted helminth infections in at-risk groups. Geneva: World Health Organization; 2016 (https://www.who.int/nutrition/publications/guidelines/deworming/en/, accessed 28 November 2019).
59. Preventive chemotherapy in human helminthiasis: coordinated use of antihelminthic drugs in control interventions: a manual for health professionals and programme managers. Geneva: World Health Organization; 2006 (https://apps.who.int/iris/bitstream/10665/43545/1/9241547103_eng.pdf, accessed 28 November 2019).
62. Salam RA, Haider BA, Humayun Q, Bhutta ZA. Effect of administration of antihelminthics for soil-transmitted helminths during pregnancy. Cochrane Database Syst Rev. 2015;(6):CD005547.
61. Mondadori E, Ehrhardt A, Le Anh T, Tran Cong D, Sepe G, Van Huyen N et al. Appreciation of school deworming program by parents in Ha Giang Province (Vietnam). Southeast Asian J Trop Med Public Health. 2006;37:1095–8.
62. Maternal immunization against tetanus: integrated management of pregnancy and childbirth (IMPAC). Standards for maternal and neonatal care 1.1. Geneva: Department of Making Pregnancy Safer, World Health Organization; 2006 (https://www.who.int/reproductivehealth/publications/maternal_perinatal_health/immunization_tetanus.pdf, accessed 28 November 2019).
63. Thwaites CL, Loan HT. Eradication of tetanus. Br Med Bull. 2015;116(1):69–77. doi:10.1093/bmb/ldv044.
64. Kayentao K, Garner P, van Eijk AM, Naidoo I, Roper C, Mulokozi A et al. Intermittent preventive therapy for malaria during pregnancy using 2 vs 3 or more doses of sulfadoxine–pyrimethamine and risk of low birth weight in Africa: systematic review and meta-analysis. JAMA. 2013;309:594–604. doi:10.1001/jama.2012.216231.
65. Roll Back Malaria Partnership Malaria in Pregnancy Working Group. Consensus statement on folic acid supplementation during pregnancy. Geneva; 2015 (https://www.pmi.gov/docs/default-source/default-document-library/tools-curricula/consensus-statement-folic-acid-supplementation-during-pregnancy.pdf, accessed 28 November 2019).

66. Joint Formulary Committee. Disorders of gastric acid and ulceration. Chapter 1: Gastro-intestinal system. In: British National Formulary (BNF) 72. London: BMJ Publishing Group Ltd and Royal Pharmaceutical Society; 2016.

67. Hawley G, Janamian T, Jackson C, Wilkinson SA. In a maternity shared-care environment, what do we know about the paper hand-held and electronic health record: a systematic literature review. BMC Pregnancy Childbirth. 2014;14:52. doi:10.1186/1471-2393-14-52.

68. Patil CL, Abrams ET, Klima C, Kaponda CP, Leshabari SC, Vonderheid SC et al. CenteringPregnancy-Africa: a pilot of group antenatal care to address Millennium Development Goals. Midwifery. 2013;29(10): 1190–8. doi:10.1016/j.midw.2013.05.008.

69. Jafari F, Eftekhar H, Fotouhi A, Mohammad K, Hantoushzadeh S. Comparison of maternal and neonatal outcomes of group versus individual prenatal care: a new experience in Iran. Health Care for Women Int. 2010;31(7):571–84. doi: 10.1080/07399331003646323.

70. WHO recommendation on community mobilization through facilitated participatory learning and action cycles with women's groups for maternal and newborn health. Geneva: World Health Organization; 2014 (https://www.who.int/maternal_child_adolescent/documents/community-mobilization-maternal-newborn/en/, accessed 28 November 2019).

71. Memon ZA, Khan GN, Soofi SB, Baig IY, Bhutta ZA. Impact of a community-based perinatal and newborn preventive care package on perinatal and neonatal mortality in a remote mountainous district in Northern Pakistan. BMC Pregnancy Childbirth. 2015;15:106. doi:10.1186/s12884-015-0538-8.

72. WHO recommendations: optimizing health worker roles to improve access to key maternal and newborn health interventions through task shifting. Geneva: World Health Organization; 2012 (https://www.who.int/reproductivehealth/publications/maternal_perinatal_health/978924504843/en/, accessed 28 November 2019).

73. Dowswell T, Carroli G, Duley L, Gates S, Gülmezoglu AM, Khan-Neelofur D, Piaggio G. Alternative versus standard packages of antenatal care for low-risk pregnancy. Cochrane Database Syst Rev. 2015;(7):CD000934.

74. Nyarko P, Birungi H, Armar-Klamesu M, Arhinful D, Degarnus S, Odoi-Agyarko H, Brew G. Acceptability and feasibility of introducing the WHO focused antenatal care package in Ghana. FRONTIERS final report. Washington (DC): Population Council; 2006 (https://citeseerx.ist.psu.edu/viewdoc/download?rep=rep1&type=pdf&doi=10.1.1.175.8828, accessed 28 November 2019).

75. Birungi H, Onyango-Ouma W. Acceptability and sustainability of the WHO focused antenatal care package in Kenya. Frontiers in Reproductive Health Program, Population Council; 2006 (http://citeseerx.ist.psu.edu/viewdoc/download?doi=10.1.1.175.8488&rep=rep1&type=pdf, accessed 28 November 2019).

76. Conrad P, Schmid G, Tientrebeogo J, Moses A, Kirenga S, Neuhann F et al. Compliance with focused antenatal care services: do health workers in rural Burkina Faso, Uganda and Tanzania perform all ANC procedures? Trop Med Int Health. 2012;17:300–7. doi:10.1111/j.1365-3156.2011.02923.x.

77. Von Both C, Fleßa S, Makuwani A, Mpembeni R, Albrecht J. How much time do health services spend on antenatal care? Implications for the introduction of the focused antenatal care model in Tanzania. BMC Pregnancy Childbirth. 2006;6:22. doi: 10.1186/1471-2393-6-22.

For more information, please contact:

Department of Reproductive Health and Research
World Health Organization
20 Avenue Appia, 1211 Geneva 27
Switzerland
Fax: +41 22 791 4171
Email: reproductivehealth@who.int
Website: www.who.int/reproductivehealth

Department of Maternal, Newborn, Child and Adolescent Health
World Health Organization
20 Avenue Appia, 1211 Geneva 27
Switzerland
Tel. +41 22 791 3281
Fax: +41 22 791 4853
Email: mncah@who.int
Website: www.who.int/maternal_child_adolescent

Department of Nutrition for Health and Development
World Health Organization
20 Avenue Appia, 1211 Geneva 27
Switzerland
Fax: +41 22 791 4156
Email: nutrition@who.int

World Health
Organization
